# Rheum4Games: A Game-Based Board Review to Enhance Confidence and Knowledge in Rheumatology for Internal Medicine Residents

**DOI:** 10.15766/mep_2374-8265.11597

**Published:** 2026-05-01

**Authors:** Megan Schermerhorn, Sarah Donohue, Christine Sharkey, Sonam Kiwalkar

**Affiliations:** 1 Internal Medicine Resident, Department of Medicine, Providence Portland Medical Center; 2 Assistant Professor, Division of Rheumatology, Department of Medicine, University of Wisconsin School of Medicine and Public Health; 3 Clinical Associate Professor, Division of Rheumatology, Department of Medicine, University of Wisconsin School of Medicine and Public Health; 4 Clinical Assistant Professor, Department of Medicine, Washington State University Elson S. Floyd College of Medicine

**Keywords:** Games, Internal Medicine, Rheumatology, Team-Based Learning, Game-based Learning

## Abstract

**Introduction:**

Internal Medicine (IM) residents have typically scored low on the rheumatology component of the In-Training Exam (ITE). While structured rheumatology electives can improve outcomes, these experiences are not always required. Interest in the role of gamification is growing, though application in the rheumatology component of the ITE remains underexplored. We developed a gamified rheumatology board review session to assess whether incorporating gamification into existing didactic curricula could improve ITE scores and enhance resident satisfaction.

**Methods:**

We developed Rheum4Games (R4G) based on preexisting rules of the Snakes and Ladders board game. In total, 121 residents across 5 IM residencies participated over 12 sessions. Questions were developed using the American Board of Internal Medicine (ABIM) blueprint. We measured resident engagement, satisfaction, and confidence using pre- and postsession surveys. Rheumatology ITE scores were compared pre- and postsession utilizing Mann-Whitney U tests.

**Results:**

R4G improved residents’ (*n* = 117) engagement and satisfaction, with significant gains in preparedness for the rheumatology content of the ITE/ABIM Certification Exam. Among residents with matched ITE data (*n* = 106), the median ITE score change was not significantly different between R4G participants and nonparticipants. However, score improvements were significantly greater in community-based programs than in university settings (9% vs 8%).

**Discussion:**

While overall ITE score changes were not statistically significant, community-based programs showed statistically significant improvements compared to university programs. These findings suggest gamification can enhance rheumatology education and may serve as a valuable supplement to traditional didactics, especially in settings where traditional rheumatology teaching resources are limited.

## Educational Objectives

By the end of this activity, learners will be able to:

1. Identify and review high-importance rheumatology topics emphasized in the American College of Physicians Internal Medicine In-Training Exam (ITE) and American Board of Internal Medicine Certification Exam (ABIM-CE).
2. Analyze a board game–style question stem to extract pertinent clinical details.
3. Increase self-reported confidence in their preparedness for the rheumatology component of the ITE/ABIM-CE through gamified learning.
4. Enhance satisfaction and engagement with rheumatology board review by participating in a team-based gamified educational activity.

## Introduction

Rheumatology education encompasses a broad and complex range of topics that can be challenging for Internal Medicine (IM) residents to learn. IM residents have historically scored low within the rheumatology content area of the American College of Physicians Internal Medicine In-Training Examination (ITE).^1^ Studies have shown ITE scores, a surrogate marker for the American Board of Internal Medicine Certification Exam (ABIM-CE), can be improved with a structured rheumatology elective experience.^2^ However, such a rheumatology experience is not part of the core ACGME training requirements; therefore, the amount of time each resident spends training in Rheumatology varies.

Rheumatology concepts, as with many GME topics, have traditionally been taught in lecture format. Interest in the role of gamification in GME is growing, largely due to its positive impact on learner engagement and overall satisfaction.^3–6^ Gamification is an educational strategy that can encompass many different learning elements. In general, gamification is defined as “the use of game design elements in non-game contexts”^7^ or “the craft of deriving all the fun and addicting elements found in games and applying them to real-world or productive activities.”^8^ Games promote decision-making and active learning, which can enhance long-term retention of material.^9^ Using games as educational tools fosters open dialogue among learners and improves their ability to articulate their thoughts and reasoning with peers.^10^ While many card and board games exist for medical education, none specifically target rheumatology ITE or ABIM-CE content preparation within GME. Additionally, while other gamification activities have been published in *MedEdPORTAL*, most are associated with undergraduate medical education.^6,11,12^ Existing GME programs primarily use simulations or workshops rather than board game–style formats to teach medical topics.^13–15^

Therefore, we designed and implemented a gamified rheumatology board review session within our IM residency's existing didactic curricula as an attempt to improve resident engagement, satisfaction, confidence, and ITE scores. We developed Rheum4Games (R4G), a board game based on the popular board game Snakes and Ladders, where residents were divided into 4 teams and answered questions of varying degrees of difficulty in order to advance across the board. Different colored tokens and answering bells were utilized to encourage active participation and engagement. We suspected that residents would exhibit generally positive perceptions regarding gamification as a learning tool and that these perceptions would translate to improved performance on the annual rheumatology ITE scores. Furthermore, we hypothesized that residents would report higher levels of engagement, satisfaction, and confidence with gamification compared to traditional didactics.

## Methods

### Activity Design and Development

The initial concept for Rheum4Games emerged following a needs assessment that revealed low scores within the rheumatology section of the ITE and a desire to revamp existing rheumatology curriculum. To develop our PowerPoint question slide decks, we utilized the American Board of Internal Medicine (ABIM) blueprint to ensure that our game questions were based on high-yield board material.^16^ In the ABIM blueprint, Rheumatology and Orthopedics are combined into a single medical content category, comprising 10% of the assessment. We reviewed the 15 subcategories within the Rheumatology and Orthopedics medical content section. Understanding that there are too many categories to cover in our allotted time, we elected to exclude the subcategories of “regional musculoskeletal syndromes” and “sports injuries and trauma.” Additionally, we excluded “metabolic bone disease,” as these questions were covered under the Endocrinology medical content section. This resulted in 12 subcategories of questions. Further, the ABIM has designated individual topics into 5 categories (Diagnosis, Testing, Treatment/Care Decisions, Risk Assessment/Prognosis/Epidemiology, and Pathophysiology/Basic Science). These categories are designated as high, medium, or low importance. We reviewed all questions to ensure coverage of all categories designated as high importance and at least 70% of those categorized as medium importance. We divided our questions into 2 PowerPoint question decks, one containing easier questions and the other containing more challenging questions. Our question decks (Appendix A and Appendix B) were extensively reviewed by 2 general medicine educators, 1 rheumatology fellow in-training, and 3 rheumatologists in academic/community practice. Edits were made based on feedback. A pilot session was conducted prior to completing the formal sessions. Ten residents and 2 medical educators participated in the pilot session. Based on their feedback, minor revisions were made to the game mechanics and pre- and postsession surveys.

### Curricular Context and Learner Preparation

We collaborated with 5 IM residency programs to implement our learning activity during designated didactic sessions. Of the 5 programs, 4 were community-based programs and 1 was a university academic program. A total of 12 game sessions were conducted across 5 residency programs, with a total of 8–12 residents participating in each session. In total, 121 individual residents took part in the sessions.

### Implementation

Each implementation was conducted over 2 hours. No preparation was required by residents or facilitators prior to the activity. At the beginning of each session, all residents were given a presession survey consisting of 5 questions (Appendix C). Following completion of the survey, game instructions were displayed utilizing the PowerPoint slide deck (Appendix D). The facilitator, typically a rheumatology clinical educator, would clarify rules and answer any questions. Residents were then divided into 4 teams, typically with 2–4 residents per team. Each team would designate a team leader who would provide the team's answer. Each team was assigned a different color game token (yellow, red, blue, or green) that was used to progress along the game board (Appendix E). Each team was also given a tap bell with the same color corresponding to their assigned token color, which was to be tapped when they were ready to answer. The tap bells and game board were purchased from a third party for the activity.

One team would volunteer to answer a question first, followed by the subsequent teams taking their turns in a clockwise order. On each team's turn, the PowerPoint slide deck containing easier questions was displayed on the screen for the team and all residents in the room to review. The question would be read by the facilitator aloud to the room. The designated team would have 10 seconds after the question was completely read aloud to discuss as a team and come up with an answer. Once an answer was decided, the team would ring their assigned bell and state the answer. If the answer was incorrect, the team would stay in place. If the answer was correct, the team would roll the dice and move forward on the game board. If the team landed on a space that was neither a snake nor a ladder, they would be considered safe, and no additional question would be provided. If the team landed on a ladder, they would be given an additional question from the PowerPoint slide deck that contained more challenging questions. If the team landed on a ladder, and if the question was answered correctly, the team would climb up the ladder to progress further along the board game; if the question was answered incorrectly, the team would stay in place. If the team landed on a snake, they would be similarly provided an additional question from the challenging question bank. This time, if a team answered correctly, they would remain in their current position. If they answered incorrectly, they would slide down the snake and move backward on the board.

The facilitator provided further explanations and learning points for each question after the answers were revealed. The team that either reached the end of the board game first or advanced furthest from the starting point at the end of the allotted time would win the game. If any questions were left over in the PowerPoint deck after the game was complete, residents were encouraged to review the material on their own. Of note, the PowerPoint slide deck with answers was sent to all residents following completion of the sessions.

### Evaluation

Following completion of the session, we requested that all residents fill out a postsession survey (Appendix C). Surveys were adapted from previously published gamification and medical education evaluation tools, with additional items developed de novo to address constructs specific to rheumatology board preparation and the R4G game mechanics.^3,4,17^ Utilizing 5-point Likert scales, our survey measured participants’ levels of engagement, satisfaction, and confidence with gamification as compared to traditional didactics (Kirkpatrick Level 1).^3,4^ We also compared presession aggregate rheumatology ITE scores (obtained in 2023) to postsession aggregate rheumatology ITE scores (obtained in 2024) (Kirkpatrick Level 2).

De-identified scores were entered into Microsoft Excel. Mann-Whitney U tests were run to determine whether there were statistically significant differences in median ITE scores (percentage of participants with correct rheumatology ITE 2024 scores) between those who attended R4G and those who did not attend, as well as to compare pre- and postsession survey responses. *P* values less than or equal to .05 were considered statistically significant.

## Results

A total of 121 IM residents participated in the R4G sessions conducted across 5 residency programs. Of these, 118 (97.5%) completed the presession survey, and 117 (96.7%) completed the postsession survey, providing robust data on learner perceptions.

Pre- and postsession surveys included brief, targeted items assessing resident confidence, engagement, and satisfaction with the gamified format. The presession survey contained 5 items, including an item gauging residents’ confidence in their performance on the rheumatology section of the ITE/ABIM-CE. Responses were rated on a 5-point Likert scale (1 = *Not at all confident*; 5 = *Extremely confident*). The postsession survey was expanded to 17 items and assessed participants’ reactions to the session (rated on a 5-point Likert scale; 1 = *Strongly disagree*; 5 = *Strongly agree*), including reactions indicating that the game helped them identify gaps in their rheumatology knowledge, and that they preferred the R4G game format over traditional lectures. The postsession survey also included participants’ ratings of their postsession confidence in their performance on the rheumatology section of the ITE/ABIM-CE (using the same 5-point Likert scale).

Residents (*n* = 117) reported a significant increase in self-reported confidence in the rheumatology section of the ITE and the ABIM-CE following the session. Prior to the session, only 3% of respondents reported feeling *quite confident*, and 13% reported feeling *moderately confident*. Postsession, the percentage of respondents reporting these confidence levels rose to 24% and 50%, respectively (*P* < .0001) (Figure). Postsession survey responses reflected high levels of learner satisfaction (Table). On a 5-point Likert scale, participants rated the activity highly for enjoyment, usefulness of the debriefing, and overall engagement. Most respondents agreed that the game helped identify knowledge gaps, enhanced retention of rheumatology information, and supported skill development in collaboration and communication. The activity was not perceived as stressful, and most participants preferred the game format over traditional lectures.

**Figure.**
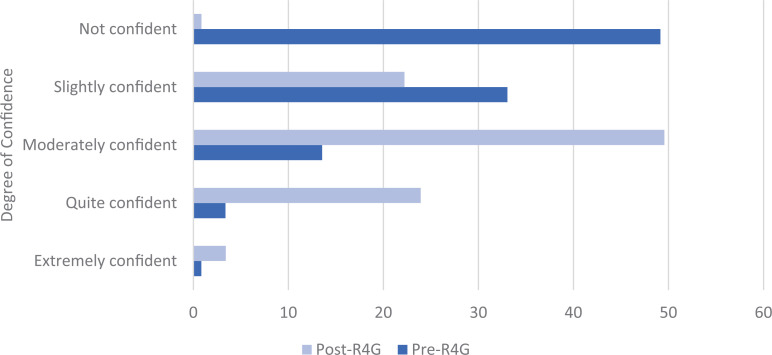
Percentage of residents (*N* = 118) who reported their confidence levels (rated on a 5-point Likert scale; 1 = *Not at all confident*; 5 = *Extremely confident*) in their performance on the rheumatology section of the American College of Physicians Internal Medicine In-Training Exam (ITE) and American Board of Internal Medicine Certification Exam (ABIM-CE) before (Pre-R4G) and after (post-R4G) the Rheum4Games (R4G) activity. Comparisons were performed using Mann-Whitney U test (level of significance, *P* ≤ .05).

**Table.**
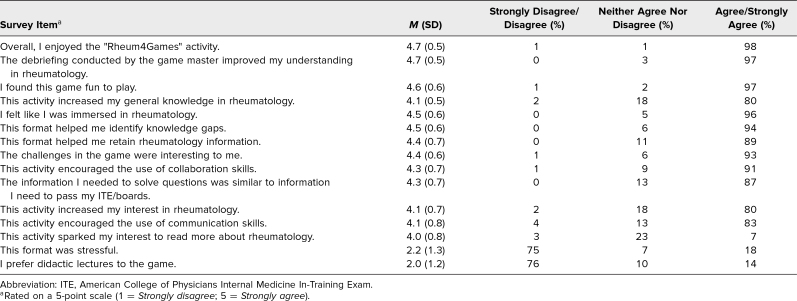
Participant Feedback From Rheum4Games Postactivity Survey (*N* = 117)

The open-ended survey responses further illustrated the participants’ overwhelmingly positive reception of the R4G sessions. Many residents emphasized the engaging and interactive nature of the activity, with comments such as “I had so much fun learning in this format! I wish all lectures were like this” and “What a wonderful and engaging session.” Several participants appreciated the unique learning approach, noting that the session was both “very fun and informative” and a “great way to review rheumatology in a fun and engaging way.” In addition to its enjoyable format, residents highlighted its educational effectiveness and noted that it combined “helpful bullet points and instructor reinforcement of pertinent information.” Constructive feedback included suggestions for logistical improvements, such as shortening the session duration, highlighted by the comment “I think it can be limited to 90 minutes. It was super fun, but I started forgetting questions towards the end,” and enhancing participation dynamics by allowing broader group discussions before answers were revealed, as indicated by the comment “When one group gets it wrong, open it up to the larger group before revealing the answer.”

Separately, 106 residents across these programs had available ITE scores for both 2023 and 2024. Of the 106 residents, 68 (64.2%) participated in an R4G session, while the remaining 38 (35.8%) did not participate in an R4G session. No statistically significant difference in the postsession median change in rheumatology ITE score was observed between the participant group and the nonparticipant group (median score change 9% for each; *P* = .63, by Mann-Whitney U test). Additionally, there was no significant difference in median score improvement between the participant group and nonparticipant group at either the PGY-2 level (*n* = 30; *P* = .32) or the PGY-3 level (*n* = 76; *P* = .93). When performance by program type was compared, R4G participants from community programs (*n* = 45) had a significantly greater median improvement in rheumatology ITE score as compared with those from academic programs (*n* = 23) (9% vs. 8%; *P* < .05).

## Discussion

R4G is a novel approach to prepare IM residents for the rheumatology component of the ITE/ABIM-CE. While other gamification activities exist, R4G is the first of its kind aimed at improving rheumatology knowledge specifically. Additionally, our project was multicentered and encompassed a large sample size (121 residents) across both academic and community programs.

R4G was well received by the residents across programs. Our activity aligns with other gamification methods in the literature, which typically find significant improvement in resident engagement, satisfaction, and challenge from these methods of learning.^6,17–19^ Most importantly, our residents reported feeling more prepared for the rheumatology section of the ITE and expressed greater confidence in identifying their knowledge gaps.

Although residents reported significant gains in confidence and satisfaction, the overall change in ITE performance between participants and nonparticipants did not reach statistical significance. This finding is consistent with limitations commonly described in GME assessment research, including small comparison group size, variability in baseline knowledge, and the wide time interval between the educational intervention and the ITE administration.^13,17^ Additionally, ITE performance is influenced by multiple concurrent curricular experiences, making it difficult to isolate the effect of a single didactic modality such as gamification.

Interestingly, residents from community-based programs demonstrated significantly greater improvement in rheumatology ITE scores when compared with residents from university programs. Several factors may contribute to this difference. Community programs often have smaller cohort sizes, which may create a more intimate and interactive learning environment during gamified sessions. Residents in these settings may also have more limited exposure to rheumatology faculty or fewer structured learning opportunities, making the gamified session a higher-yield supplement to their usual curriculum. Furthermore, community programs may show greater enthusiasm or novelty effect with interactive, team-based learning formats, which may enhance engagement and retention.

We completed a small pilot initially, which allowed us to make several modifications to the game mechanics. After our pilot, we learned there was a need to develop learning points for each question to provide additional education and reference material for residents. Additionally, for our initial pilot, the game was structured where the residents rolled the dice, and a question was only provided if they landed on either a snake or a ladder. This resulted in very few questions being provided over the course of the hour-long pilot. In modifying the game, we were able to complete the majority of the 2 question decks during the 90-minute sessions. Further, our initial model contained only 1 question deck, rather than 2 question decks (an easier deck and a more challenging deck). Through creating 2 decks we could still incorporate the snakes and ladders on the board mat, while keeping the question difficulty distributed fairly. We learned through our pilot that 90 minutes seemed to be the optimal amount of time to complete the game and make it through the majority of the questions, and it would be challenging to complete in less time. However, the activity is easily transferable across residency programs of all sizes and can be implemented without a rheumatologist present, as key learning points are built into the game.

While other gamification activities have been published in *MedEdPORTAL*, our activity is the first board game–style learning intervention targeting GME, as well as the first activity with a focus on rheumatology. In the GME literature on *MedEdPORTAL*, most gamification activities are simulations. Among activities with strong game-like elements, most have focused on inpatient billing, coding, or patient error reporting.^13,14^ For example, a card deck with a point system was used to teach inpatient billing, while an escape room scenario was employed to teach patient event reporting. Similar to our project, these were single-session interventions, with one conducted at a single site and the other across 2 residencies. However, neither used board game mechanics and neither included as many sites as ours. Of the remaining GME studies on medical topics, there was 1 activity targeting improved obstetrics and gynecologic emergencies using a Kahoot! style game, though it did not incorporate board game design and only reached Kirkpatrick Level 1.^15^ Three board game–style learning activities have been published on *MedEdPORTAL*, though all were designed for medical students and conducted at single institutions.^6,11,12^ Similar to our intervention, these studies used pre- and postintervention Likert-scale surveys to assess satisfaction, and all reported positive reception among learners. Our activity is unique by being multicentered and reaching Kirpatrick Level 2 by assessing knowledge retention and application through analysis of pre- and postsession rheumatology ITE scores, including comparison to a control group of participants who did not receive the intervention.

Given the positive feedback and enjoyment experienced by residents completing R4G, overall, this activity seemed to be a fun way to educate residents on rheumatology. Beyond its application to rheumatology, the R4G design offers a flexible and scalable framework that can be readily adapted to a wide range of medical education needs. The board game mechanics, structured question banks, tiered difficulty levels, and team-based decision-making can be easily modified to address other subspecialties such as Endocrinology, Cardiology, Infectious Diseases, or Pulmonary Medicine. Similarly, the format is adaptable to different training levels: simple knowledge-based questions could support early interns or medical students, while more complex clinical reasoning scenarios could challenge senior residents or fellows. Programs could also tailor game content to local curricular gaps, institutional priorities, or standardized exam blueprints. Because the game does not rely on specialty-specific facilitators and includes built-in learning points, it can be delivered by generalist faculty or chief residents, expanding its reach across diverse GME settings. This adaptability suggests that the gamified approach used in R4G may serve as a model for developing interactive, low-cost, and highly engaging educational tools across multiple domains of GME.

## Appendices


Question Bank - Easier.pptxQuestion Bank - Challenging.pptxSurvey.docxGame Rules.pptxBoard Game.docx
*All appendices are peer reviewed as integral parts of the Original Publication.*
