# Supplementary material for: Rheum4Games: A Game-Based Board Review to Enhance Confidence and Knowledge in Rheumatology for Internal Medicine Residents
Source: MedEdPORTAL. 2026 May 1;22:11597. doi: 10.15766/mep_2374-8265.11597 (PMC13133093; doi:10.15766/mep_2374-8265.11597)
Supplement: Supplementary file 1 — Question Bank - Easier.pptxQuestion Bank - Challenging.pptxSurvey.docxGame Rules.pptxBoard Game.docx [file mep_2374-8265.11597-s001.zip › A. Question Bank - Easier.pptx]

## Slide 1
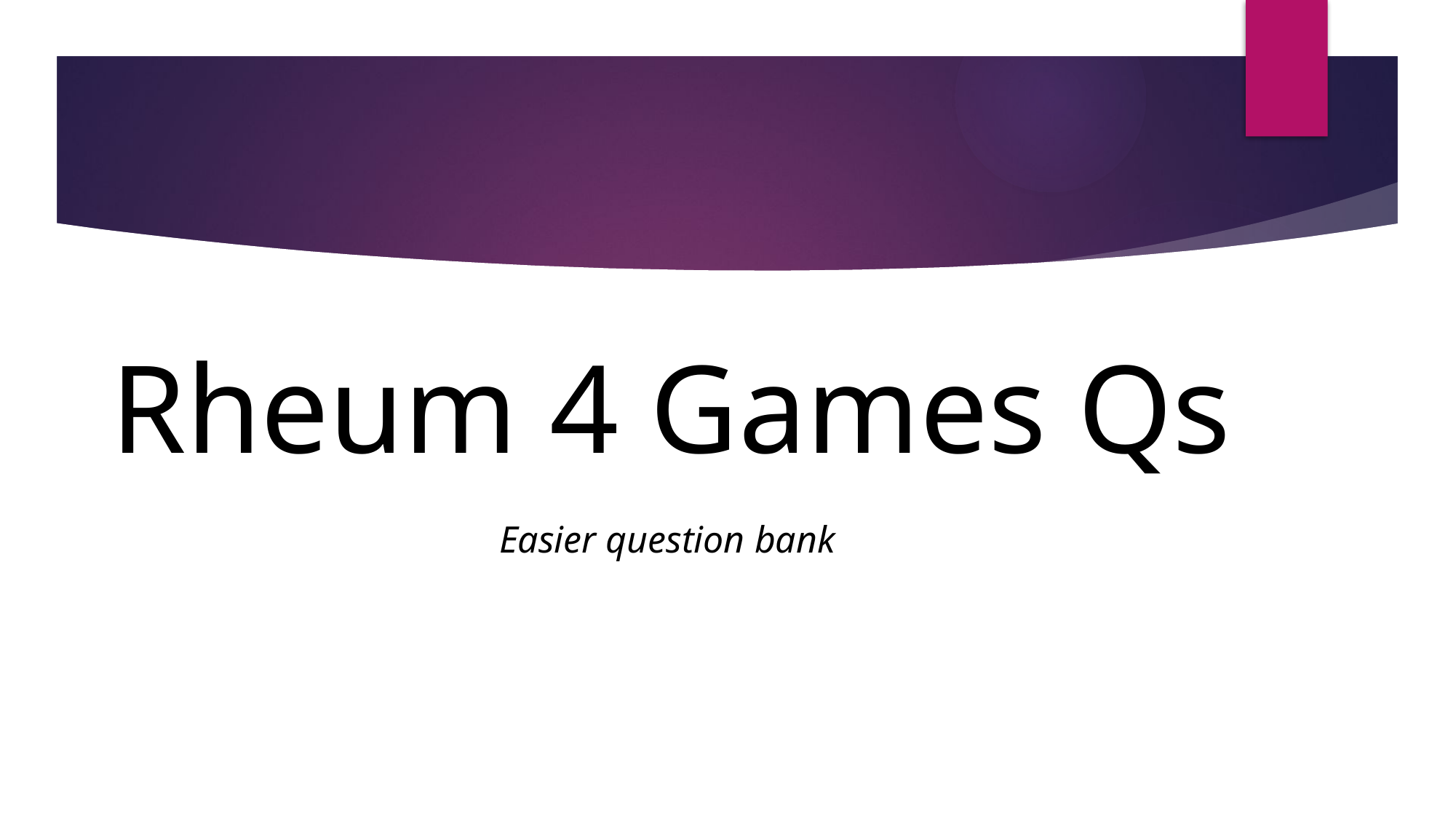

# Rheum 4 Games Qs
Easier question bank

## Slide 2
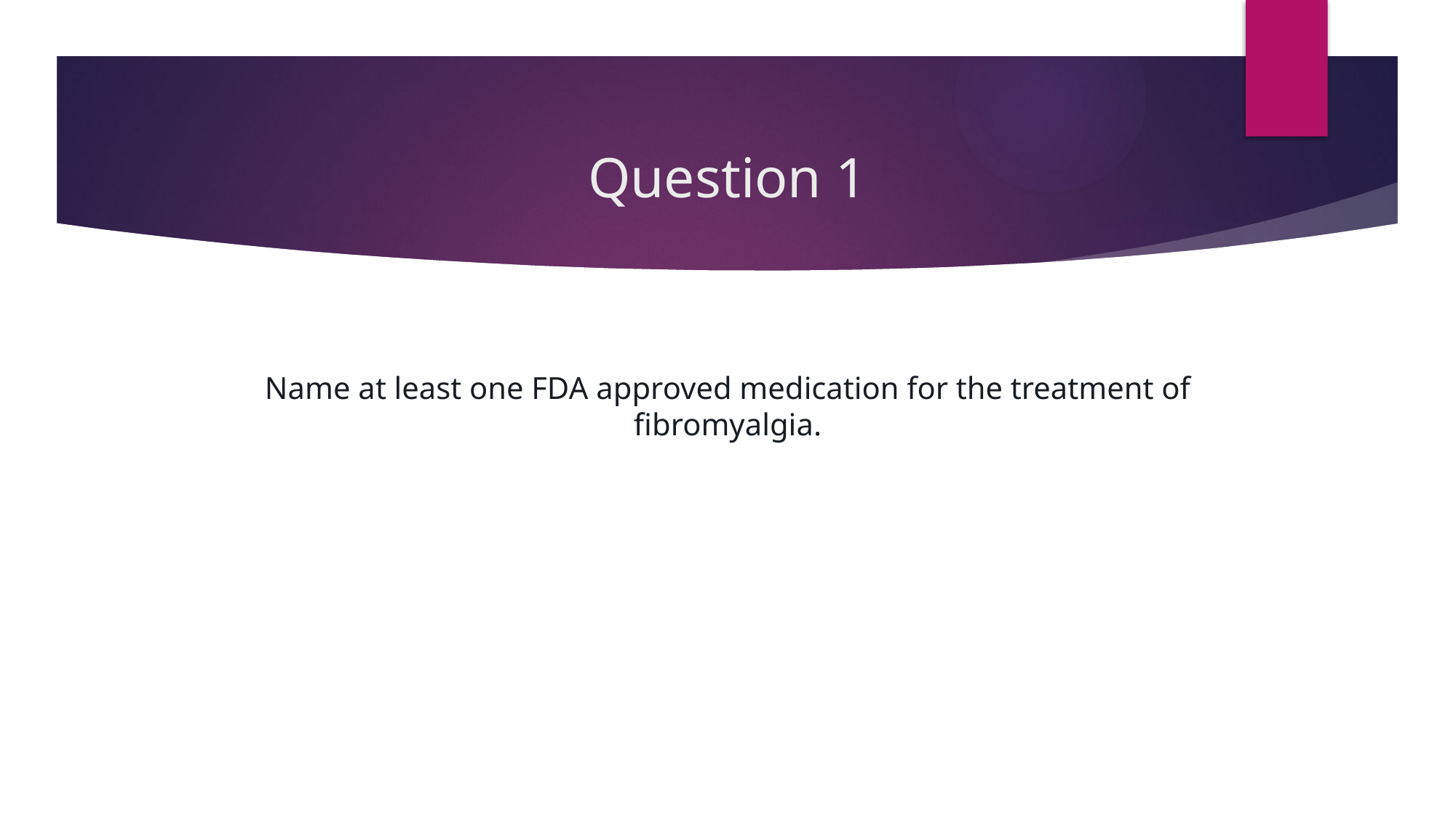

# Question 1
Name at least one FDA approved medication for the treatment of fibromyalgia.

## Slide 3
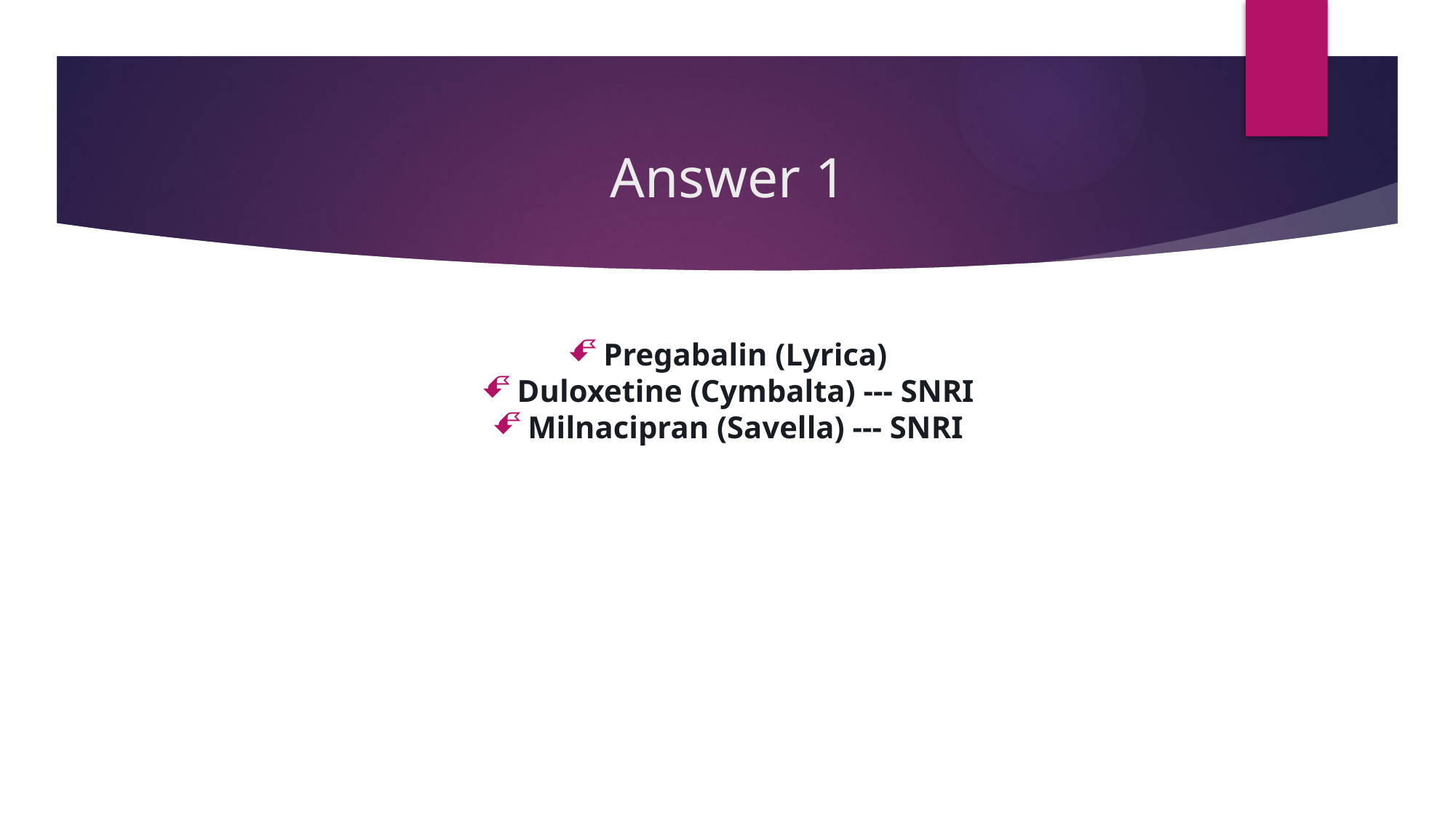

# Answer 1
Pregabalin (Lyrica)
Duloxetine (Cymbalta) --- SNRI
Milnacipran (Savella) --- SNRI

## Slide 4
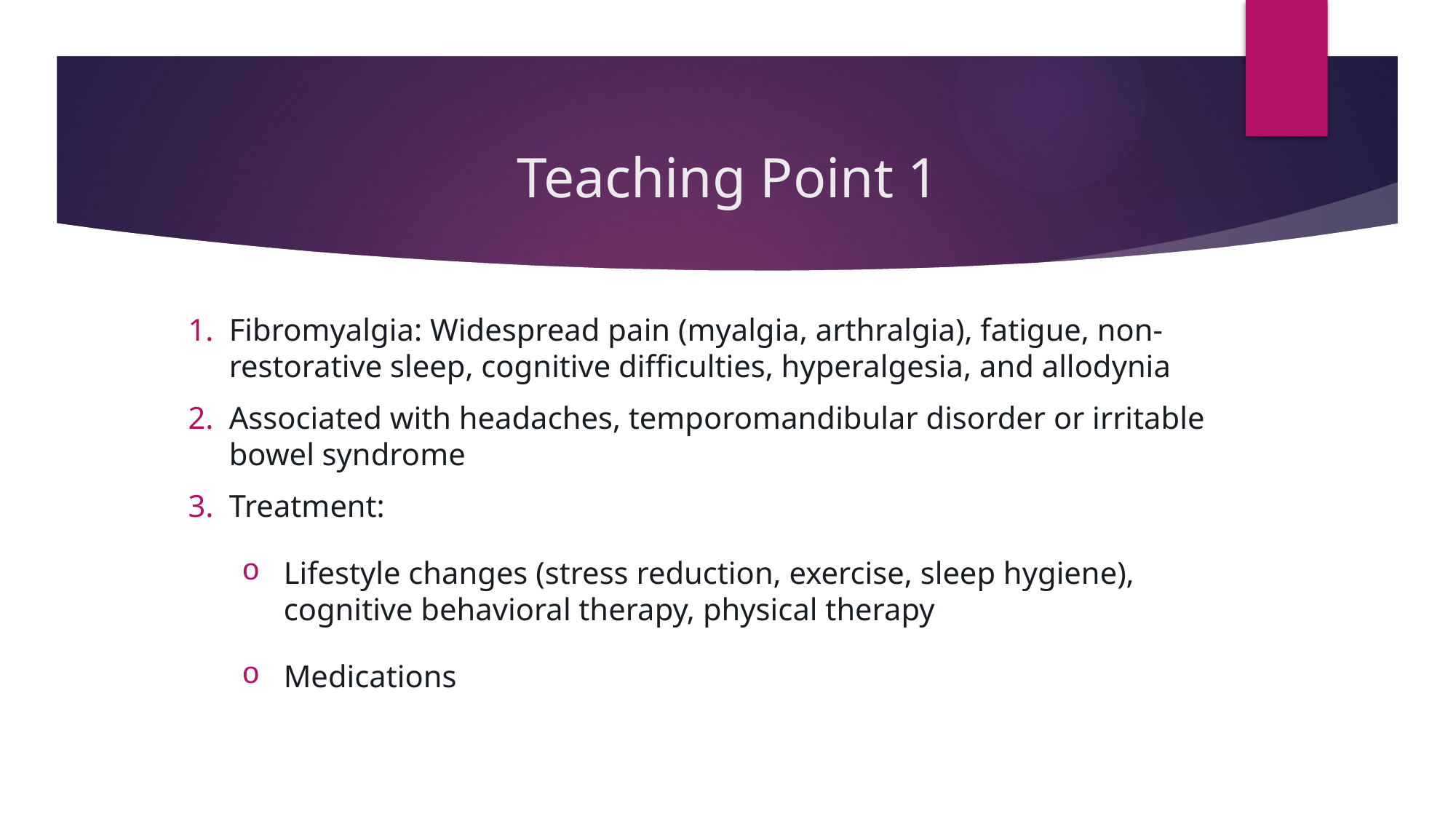

# Teaching Point 1
Fibromyalgia: Widespread pain (myalgia, arthralgia), fatigue, non-restorative sleep, cognitive difficulties, hyperalgesia, and allodynia
Associated with headaches, temporomandibular disorder or irritable bowel syndrome
Treatment:
Lifestyle changes (stress reduction, exercise, sleep hygiene), cognitive behavioral therapy, physical therapy
Medications

## Slide 5
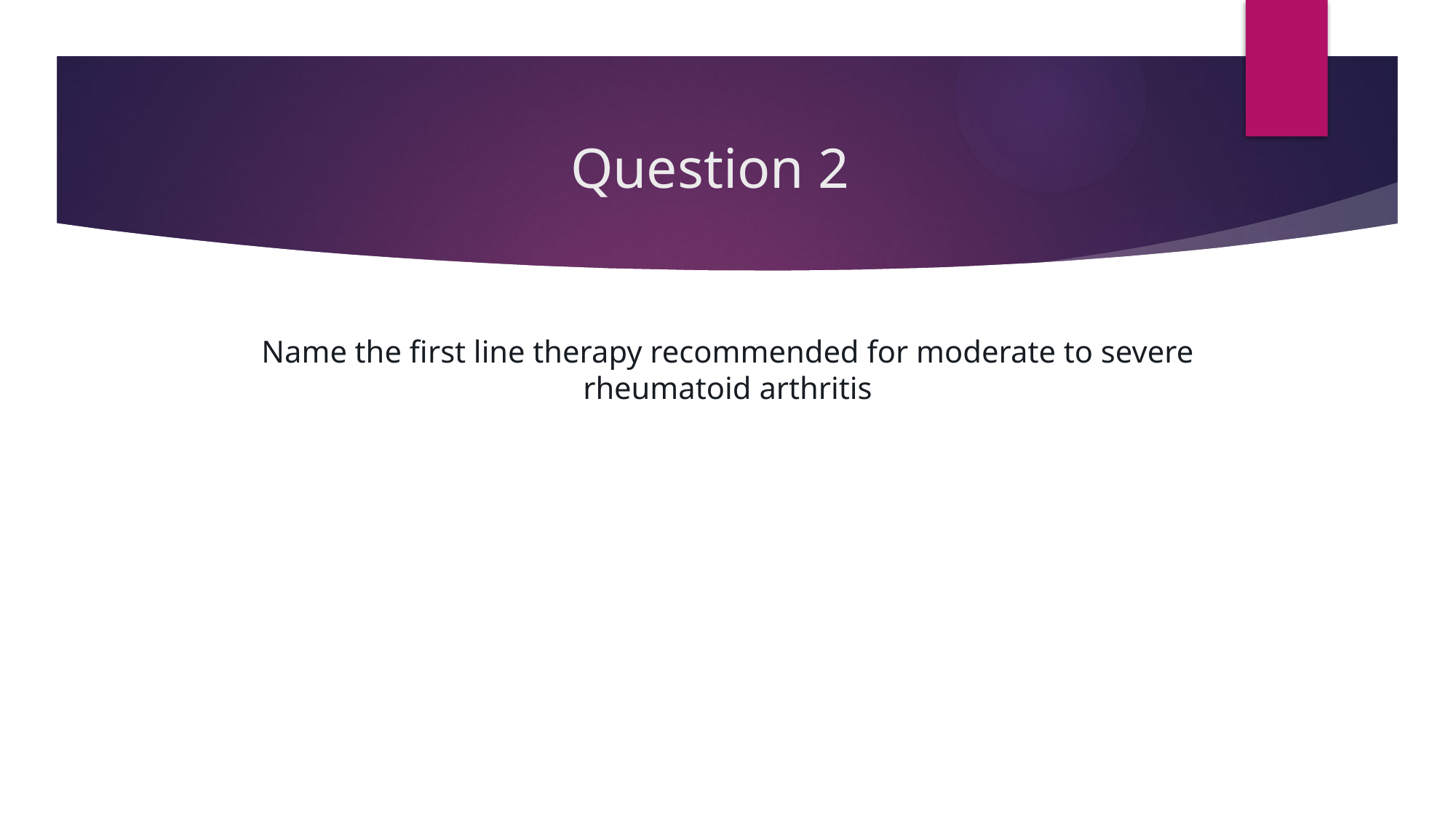

# Question 2
Name the first line therapy recommended for moderate to severe rheumatoid arthritis

## Slide 6
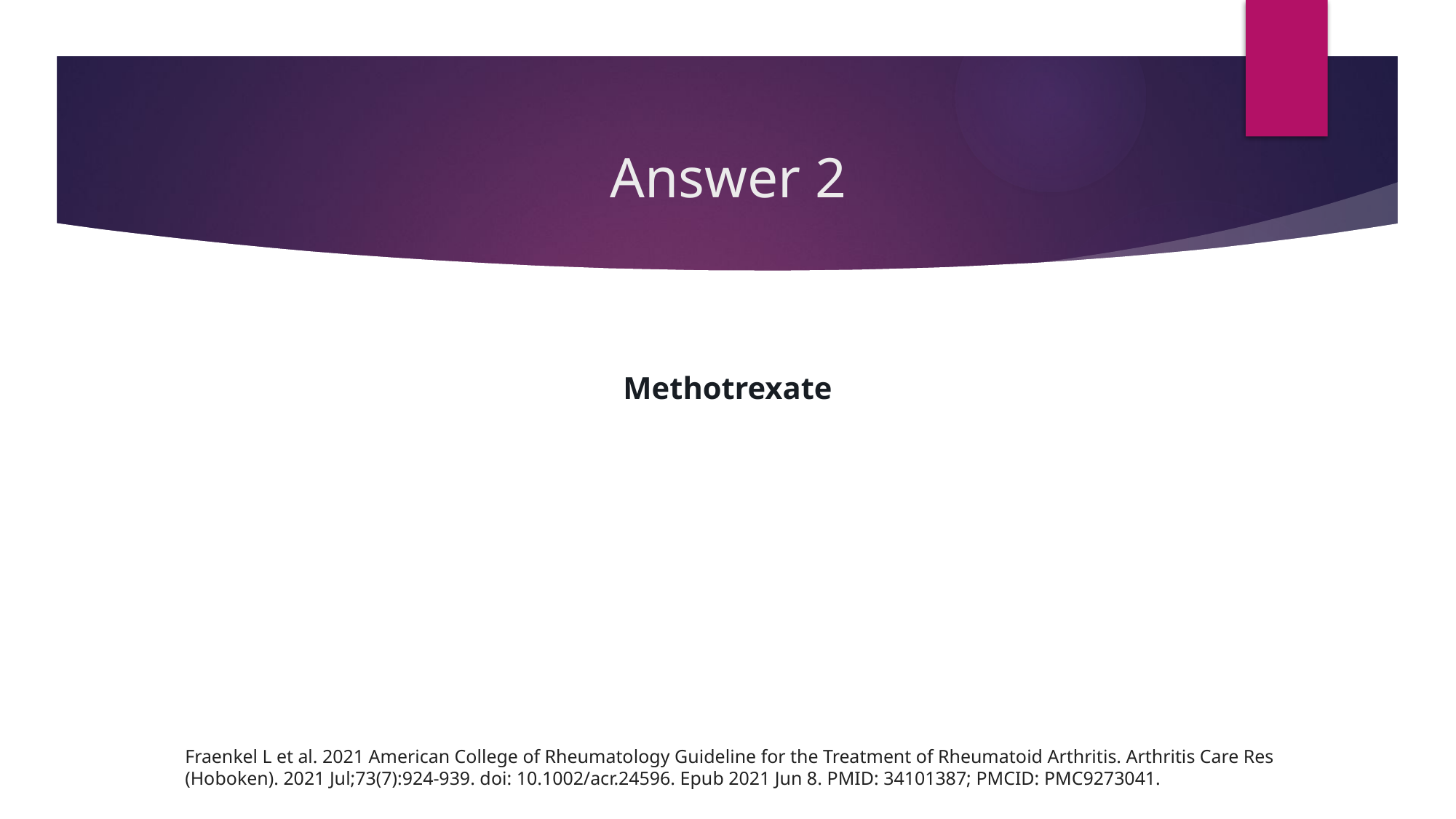

# Answer 2
Methotrexate
Fraenkel L et al. 2021 American College of Rheumatology Guideline for the Treatment of Rheumatoid Arthritis. Arthritis Care Res (Hoboken). 2021 Jul;73(7):924-939. doi: 10.1002/acr.24596. Epub 2021 Jun 8. PMID: 34101387; PMCID: PMC9273041.

## Slide 7
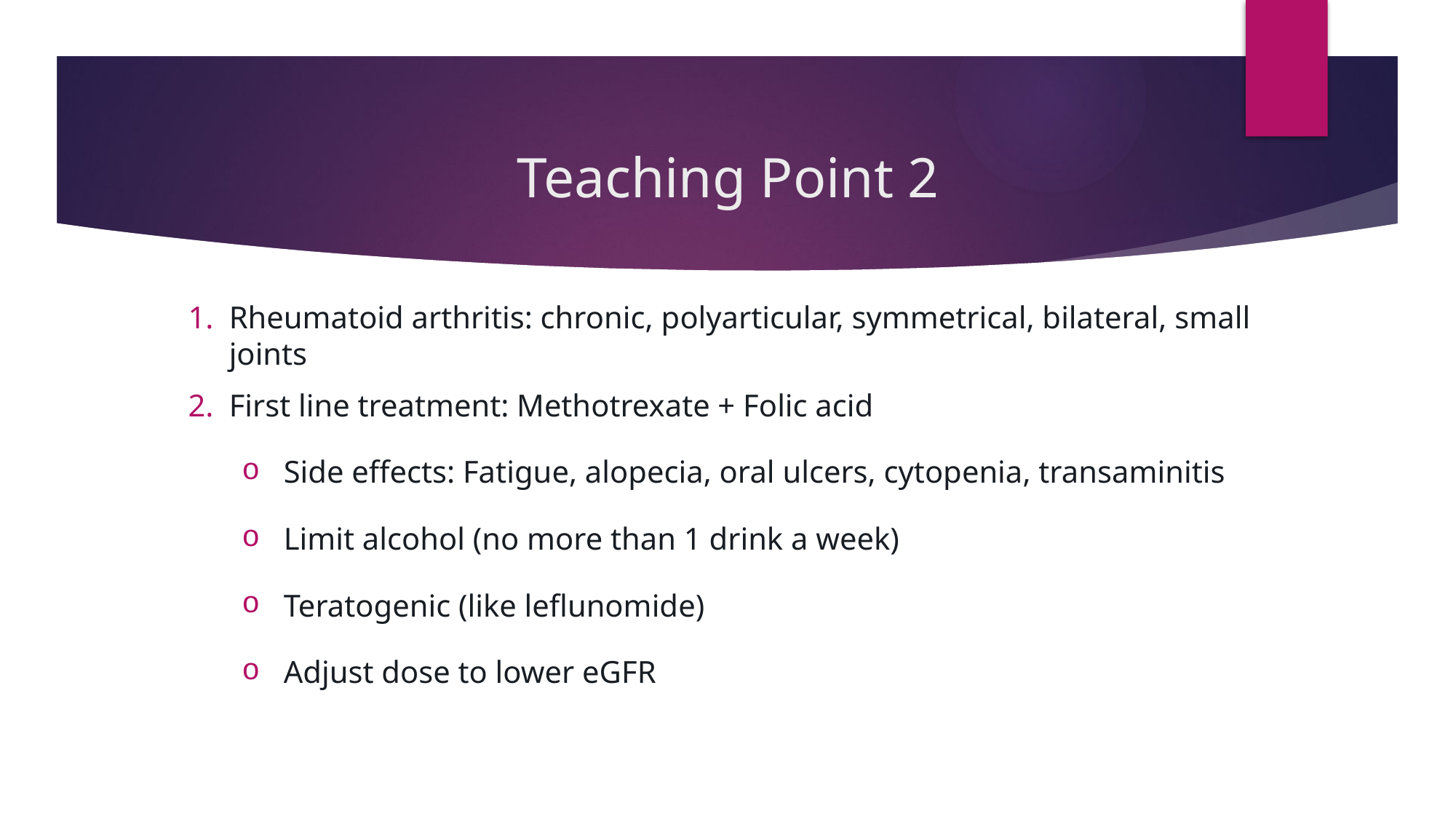

# Teaching Point 2
Rheumatoid arthritis: chronic, polyarticular, symmetrical, bilateral, small joints
First line treatment: Methotrexate + Folic acid
Side effects: Fatigue, alopecia, oral ulcers, cytopenia, transaminitis
Limit alcohol (no more than 1 drink a week)
Teratogenic (like leflunomide)
Adjust dose to lower eGFR

## Slide 8
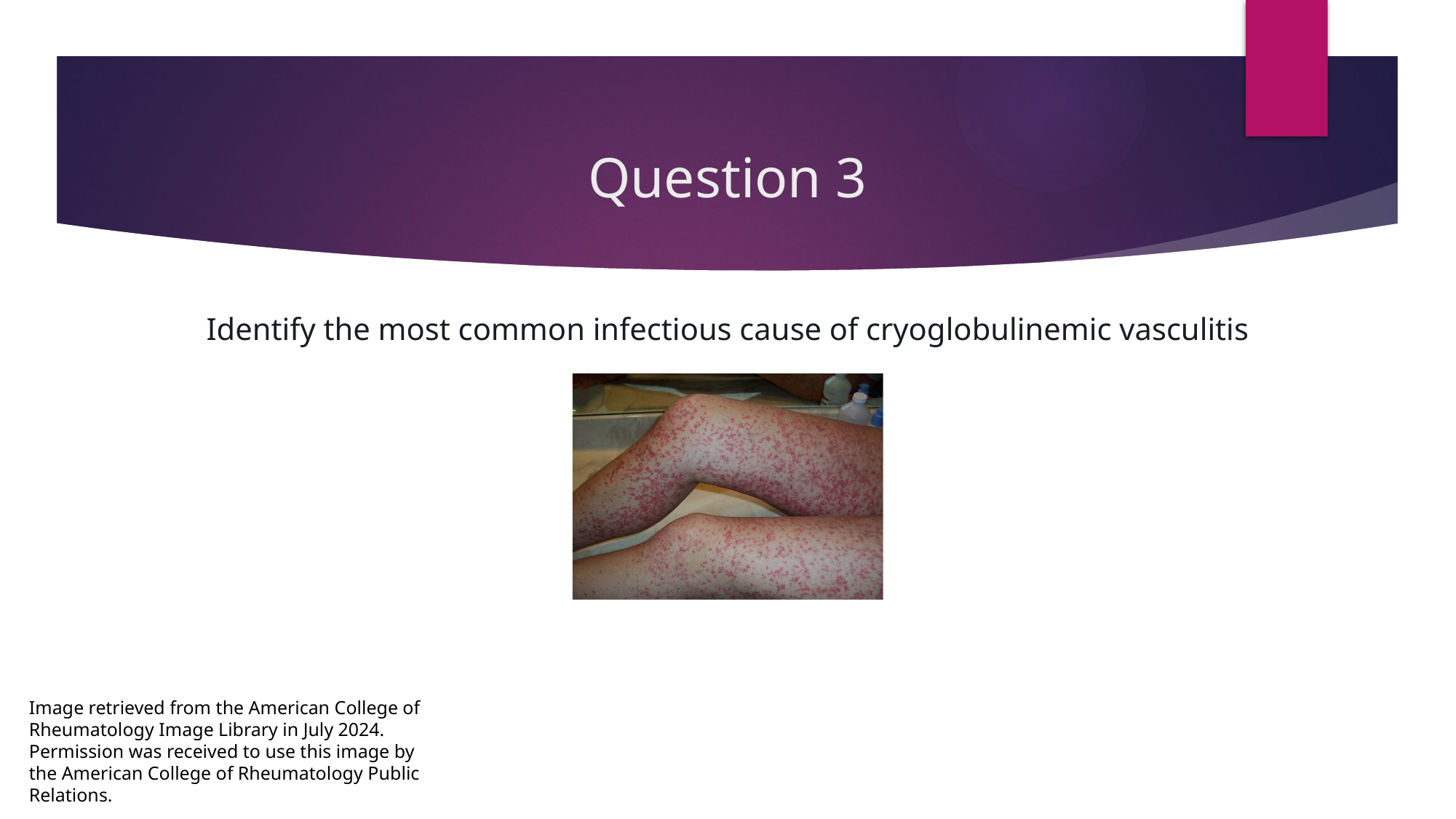

# Question 3
Identify the most common infectious cause of cryoglobulinemic vasculitis
Image retrieved from the American College of Rheumatology Image Library in July 2024. Permission was received to use this image by the American College of Rheumatology Public Relations.

## Slide 9
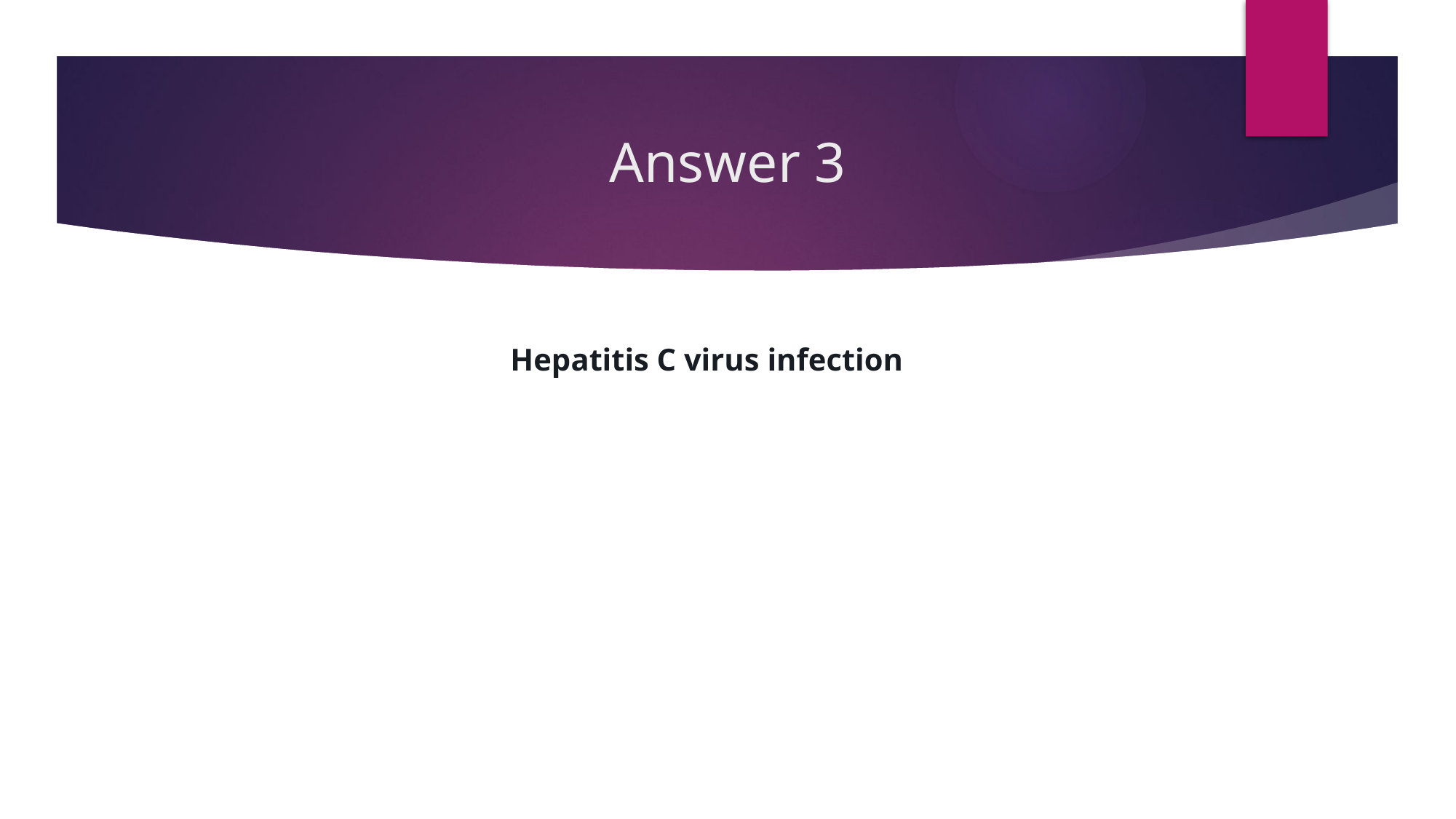

# Answer 3
Hepatitis C virus infection

## Slide 10
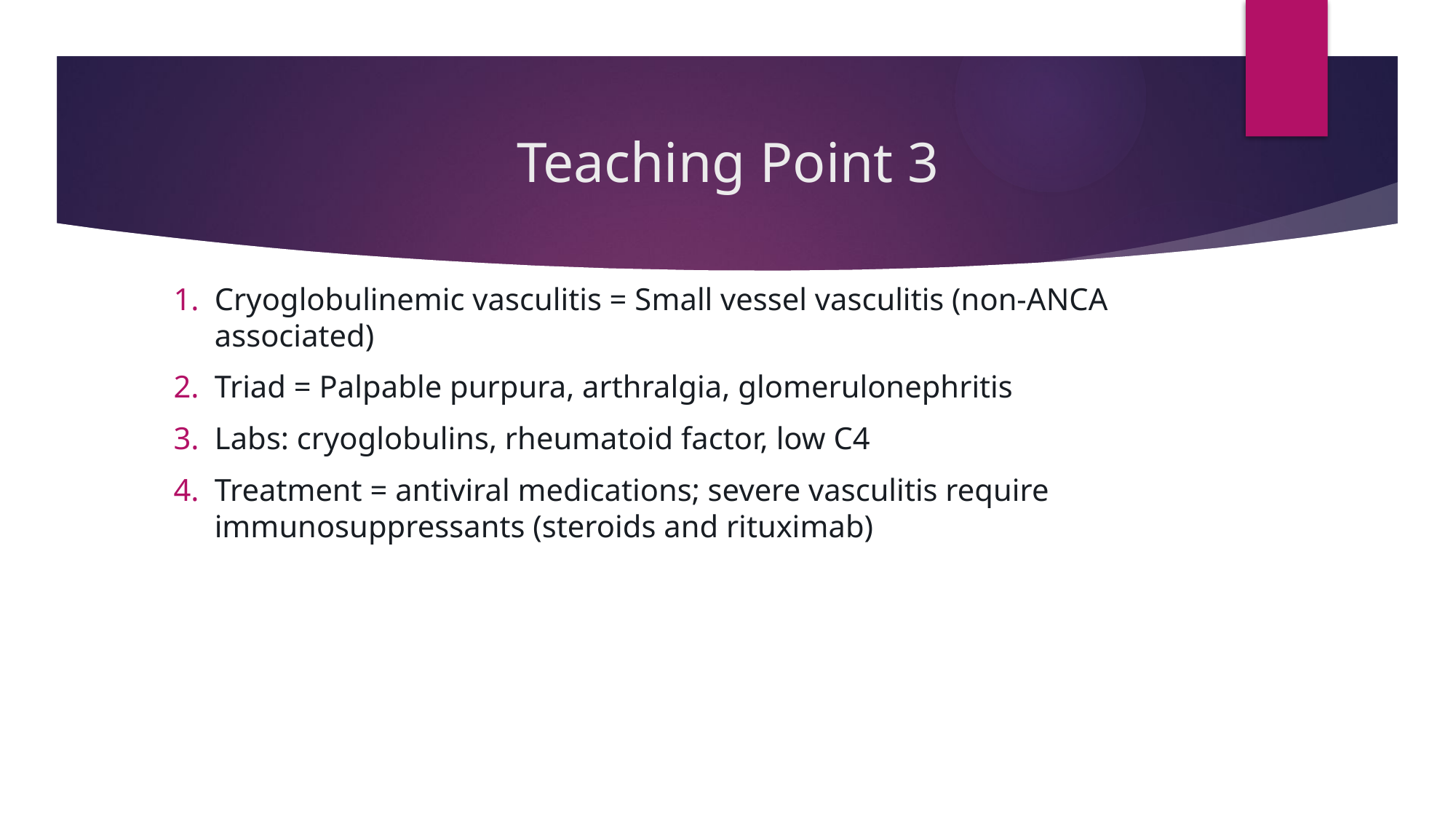

# Teaching Point 3
Cryoglobulinemic vasculitis = Small vessel vasculitis (non-ANCA associated)
Triad = Palpable purpura, arthralgia, glomerulonephritis
Labs: cryoglobulins, rheumatoid factor, low C4
Treatment = antiviral medications; severe vasculitis require immunosuppressants (steroids and rituximab)

## Slide 11
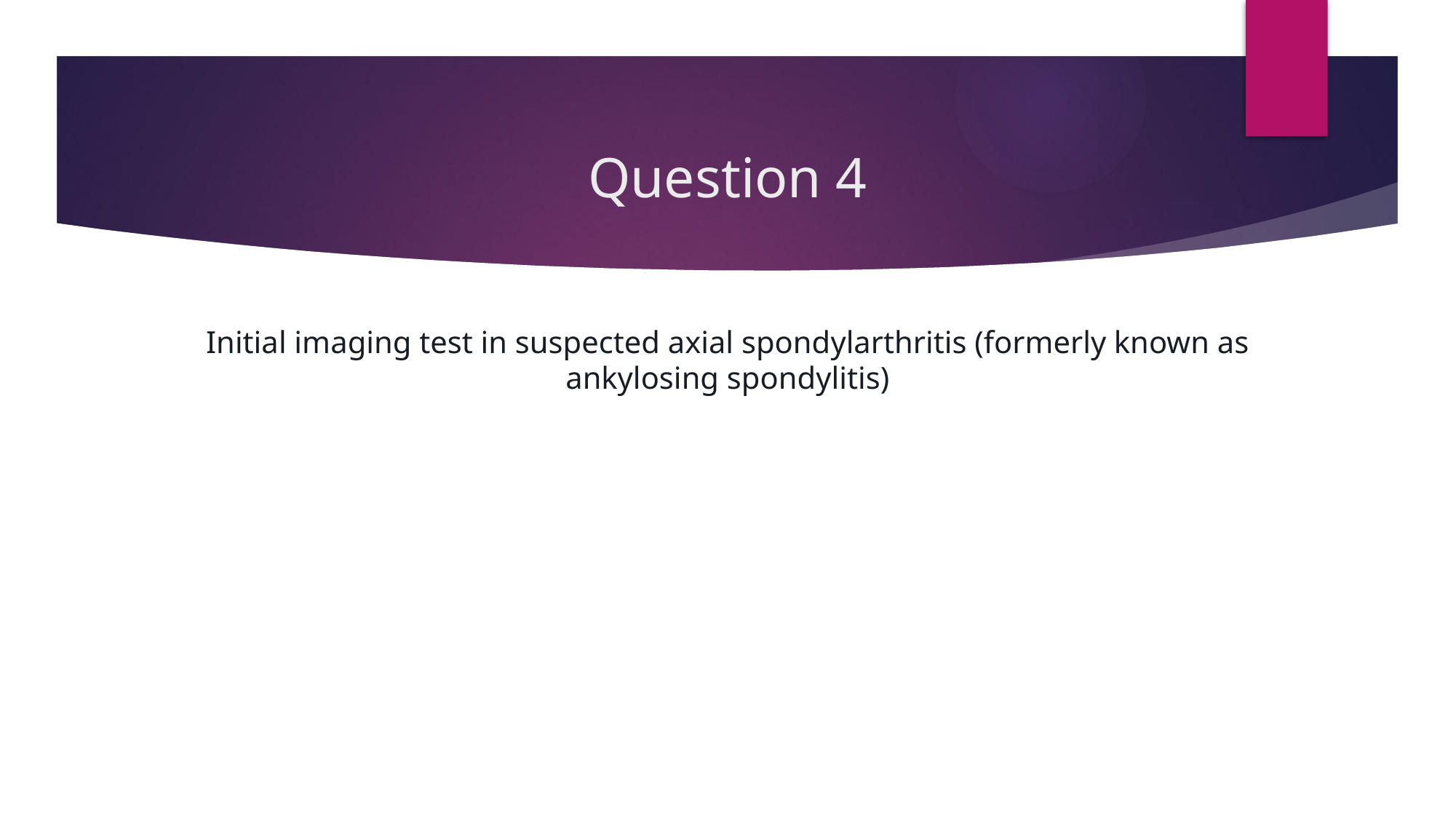

# Question 4
Initial imaging test in suspected axial spondylarthritis (formerly known as ankylosing spondylitis)

## Slide 12
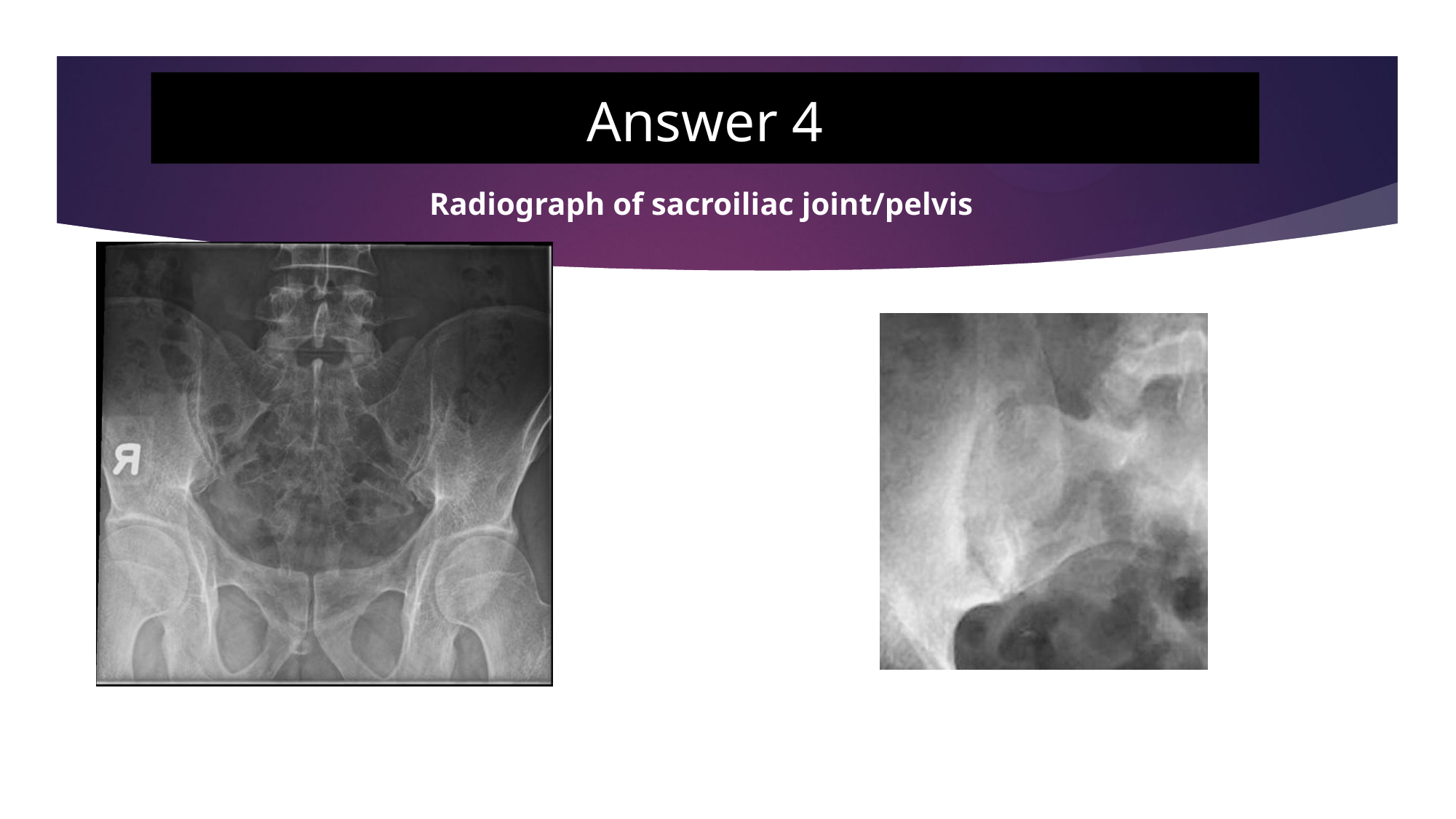

Radiograph of sacroiliac joint/pelvis
# Answer 4
Sacroiliitis
Normal SI joint Radiograph
Image by Frank Gaillard, retrieved from https://radiopaedia.org/cases/sacroiliitis-grade-iii-2?lang=us on July 24 2024. Creative Commons License associated: https://radiopaedia.org/licence?lang=us
	Image by Gni Liew, retrieved from https://radiopaedia.org/cases/normal-sacroiliac-joints-series?lang=us on July 24 2024. Creative Commons License associated: https://radiopaedia.org/licence?lang=us

## Slide 13
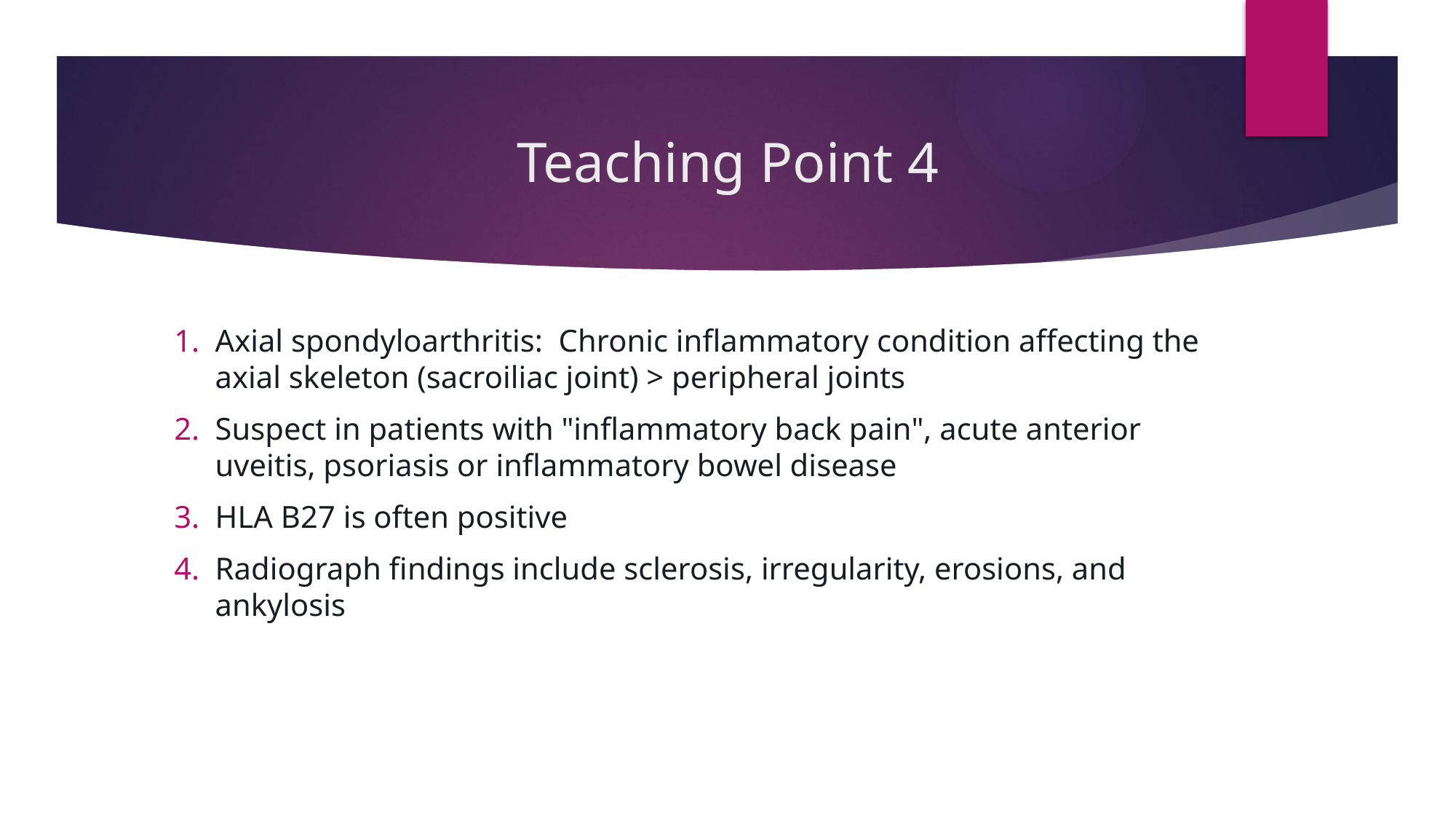

# Teaching Point 4
Axial spondyloarthritis:  Chronic inflammatory condition affecting the axial skeleton (sacroiliac joint) > peripheral joints
Suspect in patients with "inflammatory back pain", acute anterior uveitis, psoriasis or inflammatory bowel disease
HLA B27 is often positive
Radiograph findings include sclerosis, irregularity, erosions, and ankylosis

## Slide 14
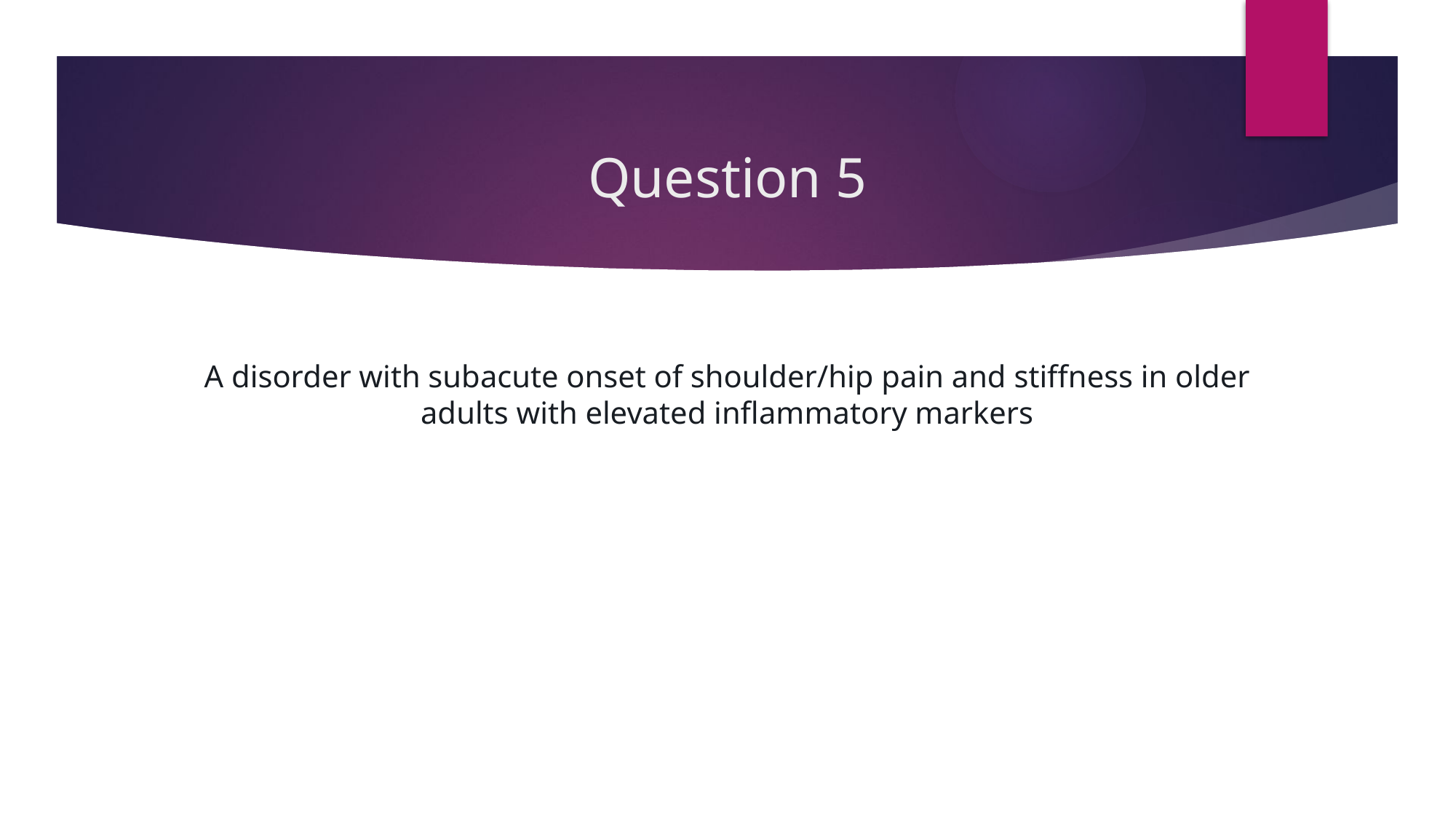

# Question 5
A disorder with subacute onset of shoulder/hip pain and stiffness in older adults with elevated inflammatory markers

## Slide 15
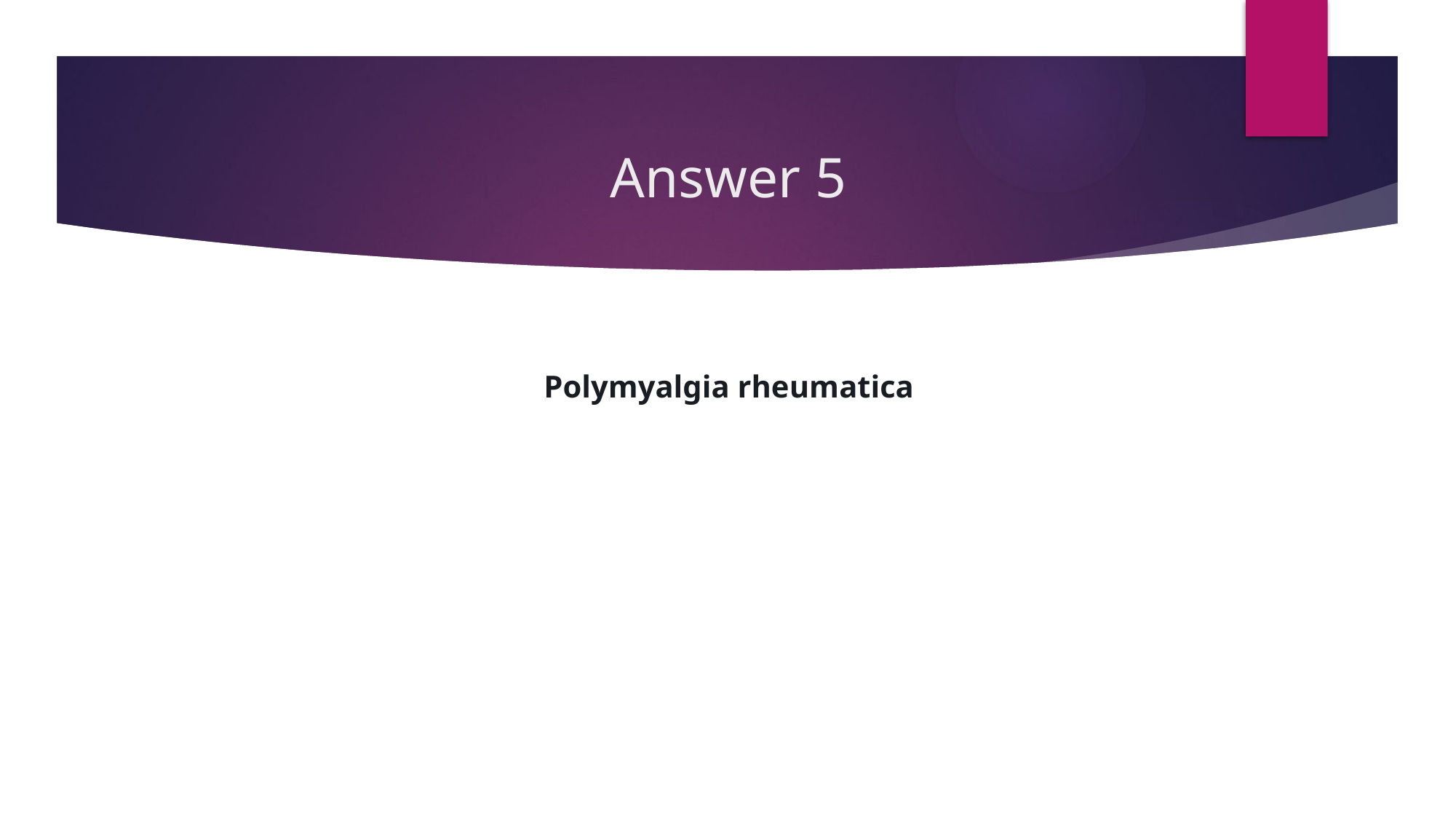

# Answer 5
Polymyalgia rheumatica

## Slide 16
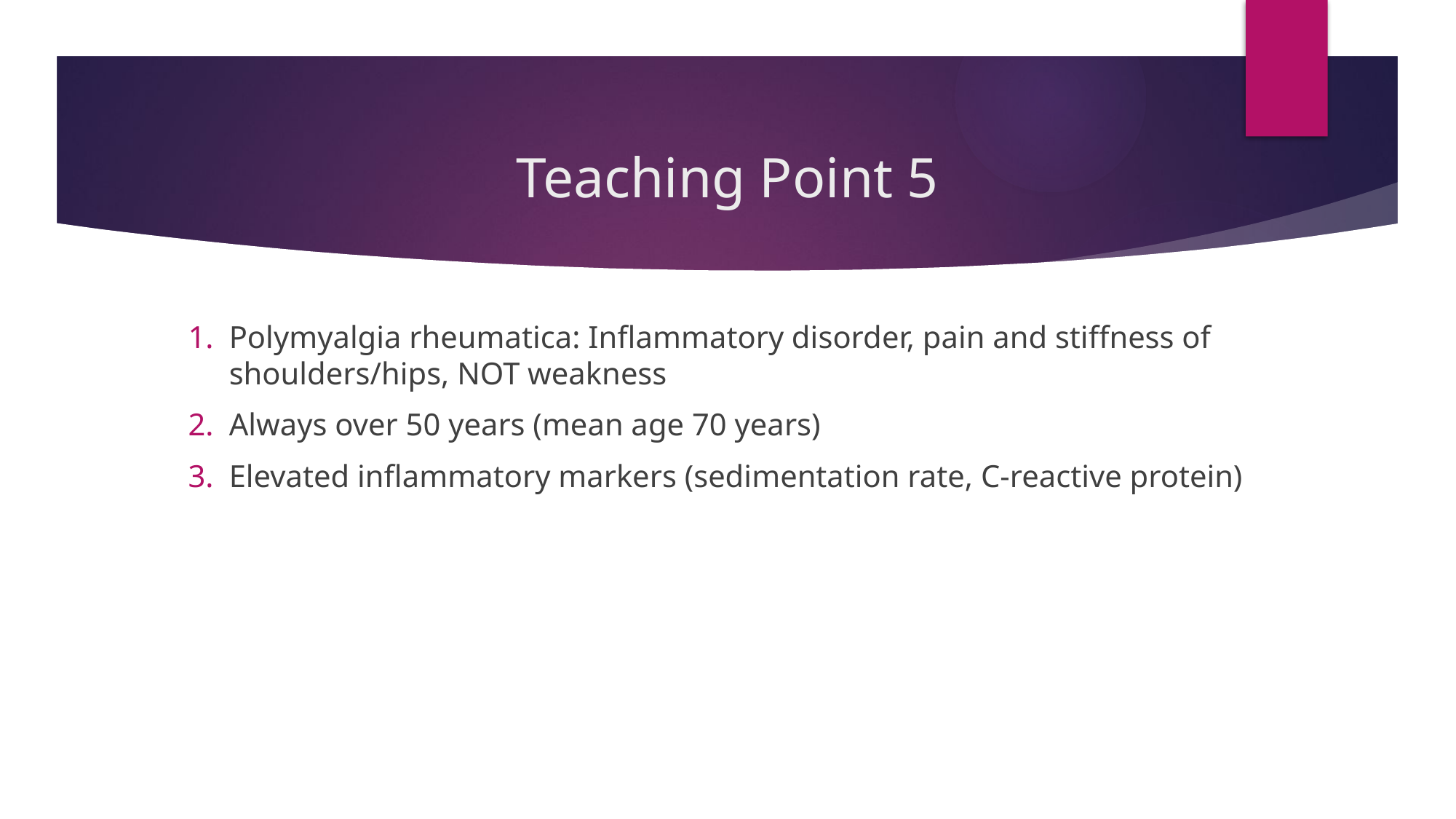

# Teaching Point 5
Polymyalgia rheumatica: Inflammatory disorder, pain and stiffness of shoulders/hips, NOT weakness
Always over 50 years (mean age 70 years)
Elevated inflammatory markers (sedimentation rate, C-reactive protein)

## Slide 17
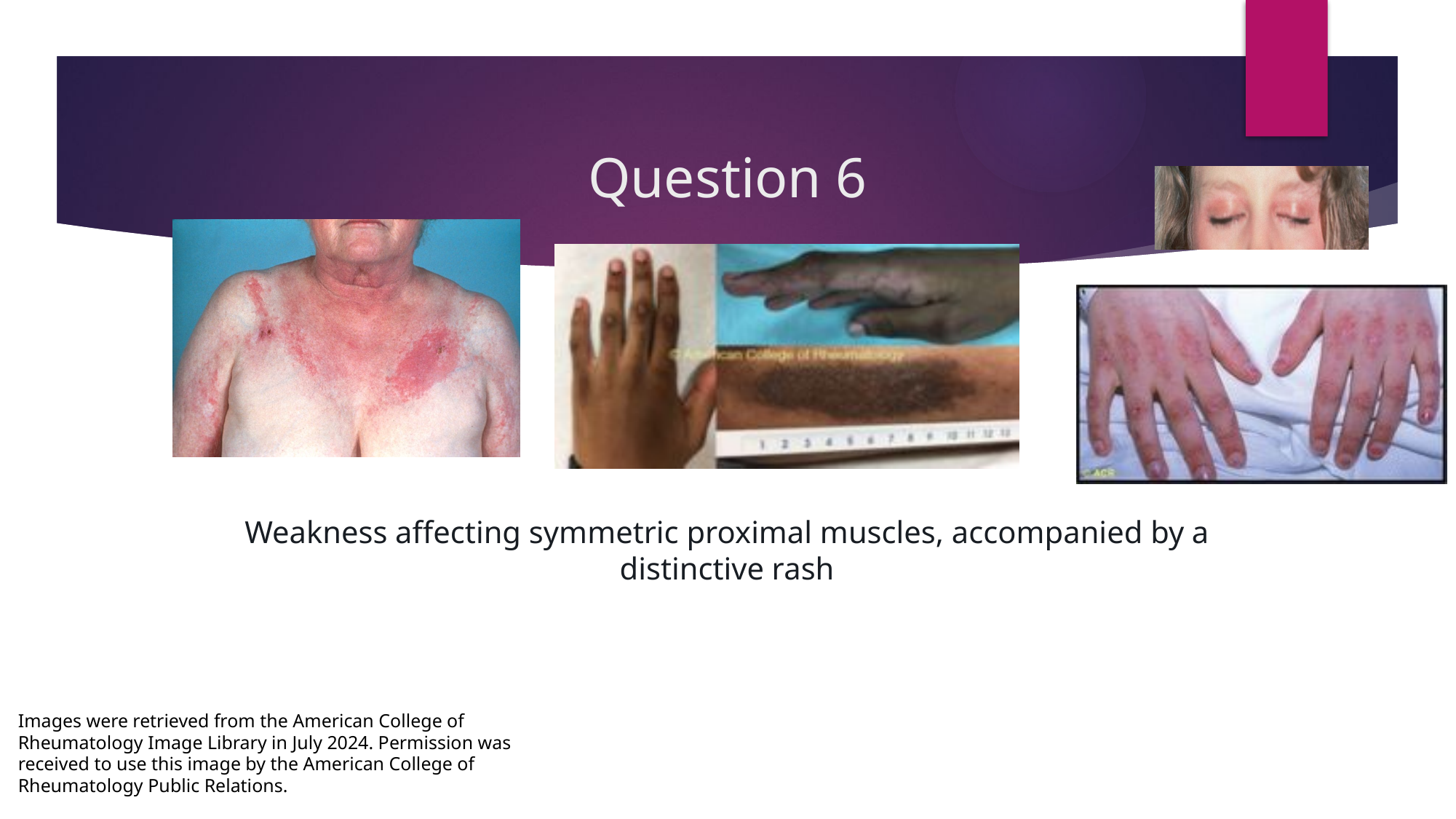

# Question 6
Weakness affecting symmetric proximal muscles, accompanied by a distinctive rash
Images were retrieved from the American College of Rheumatology Image Library in July 2024. Permission was received to use this image by the American College of Rheumatology Public Relations.

## Slide 18
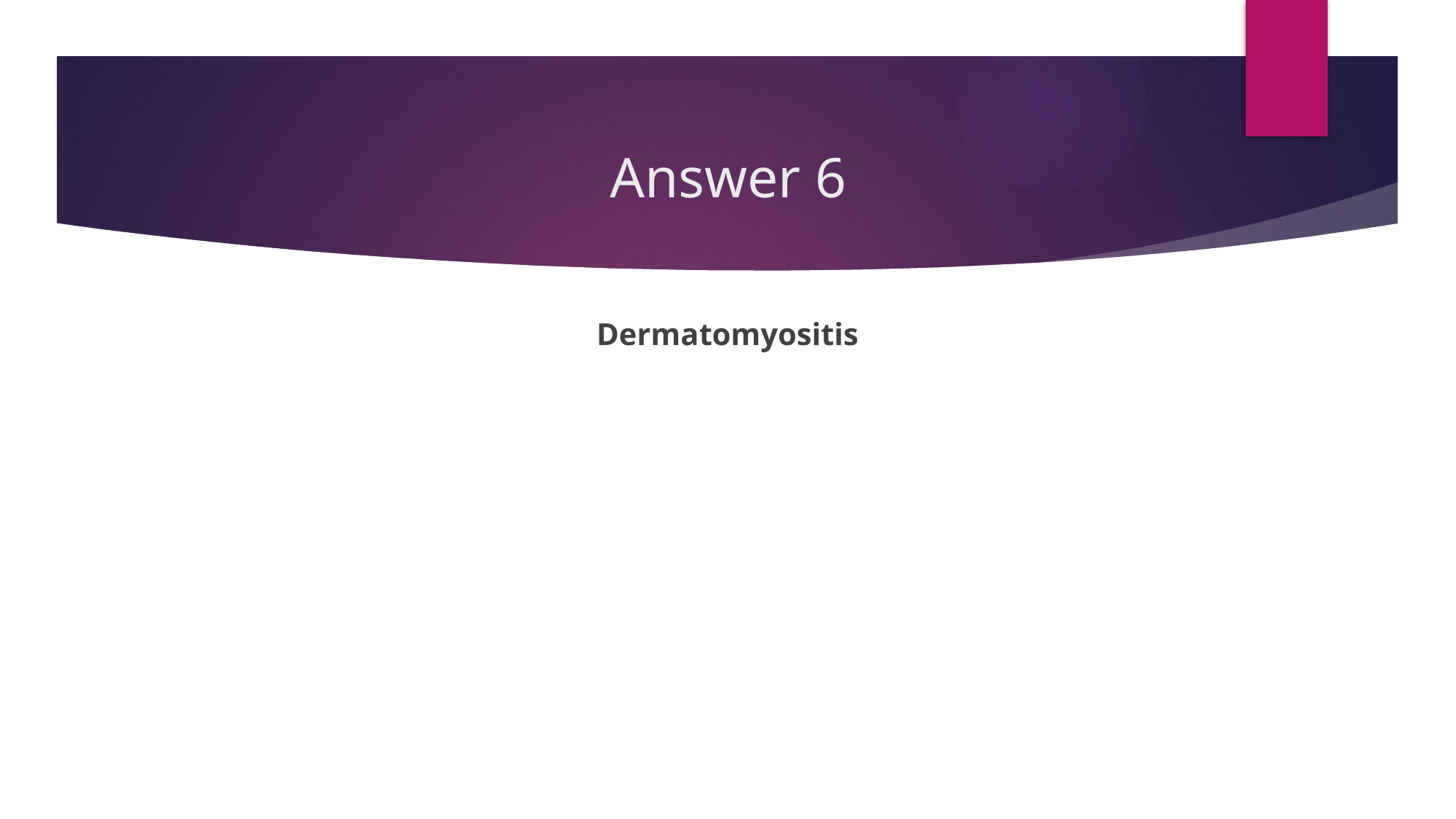

# Answer 6
Dermatomyositis

## Slide 19
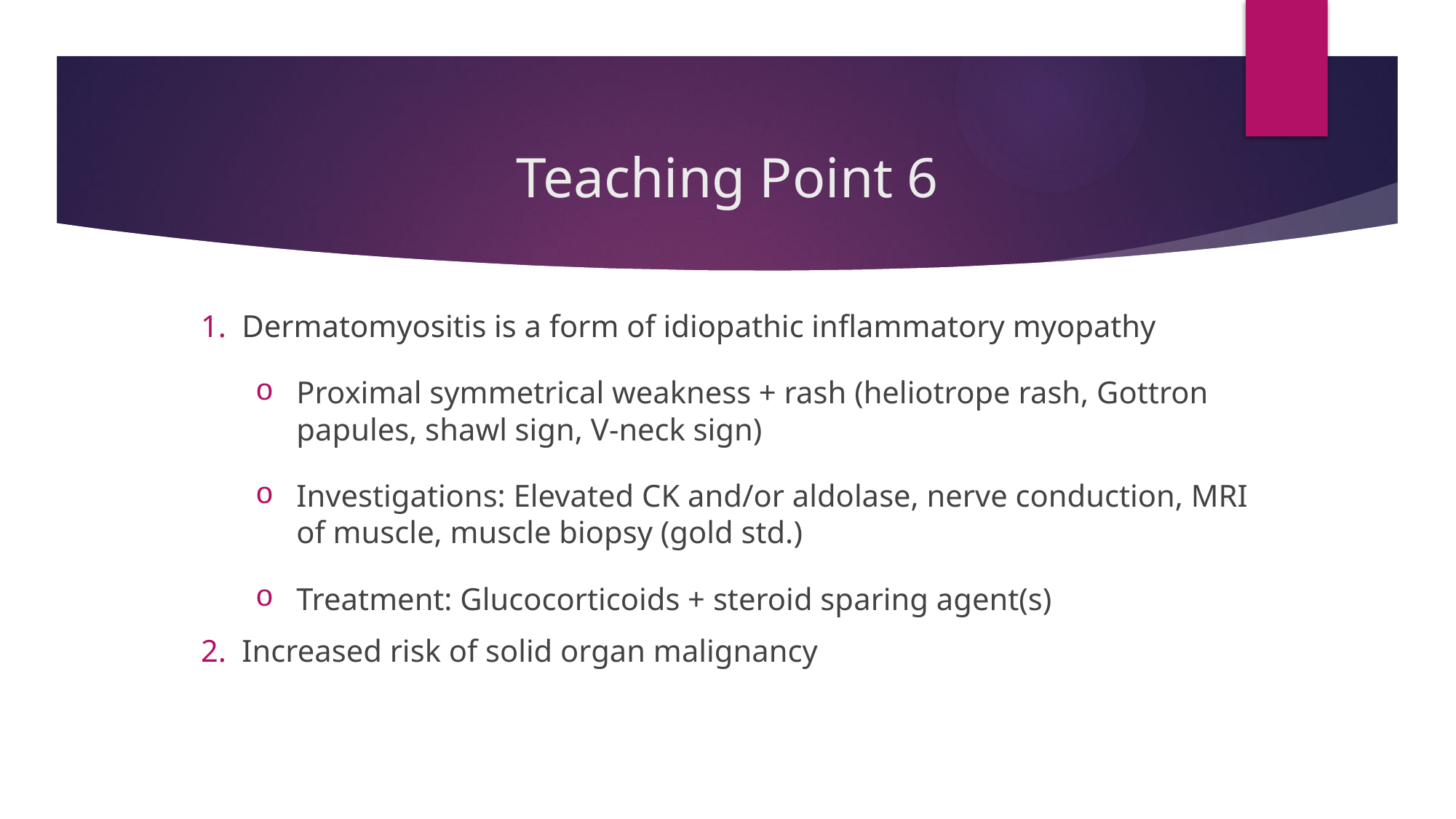

# Teaching Point 6
Dermatomyositis is a form of idiopathic inflammatory myopathy
Proximal symmetrical weakness + rash (heliotrope rash, Gottron papules, shawl sign, V-neck sign)
Investigations: Elevated CK and/or aldolase, nerve conduction, MRI of muscle, muscle biopsy (gold std.)
Treatment: Glucocorticoids + steroid sparing agent(s)
Increased risk of solid organ malignancy

## Slide 20
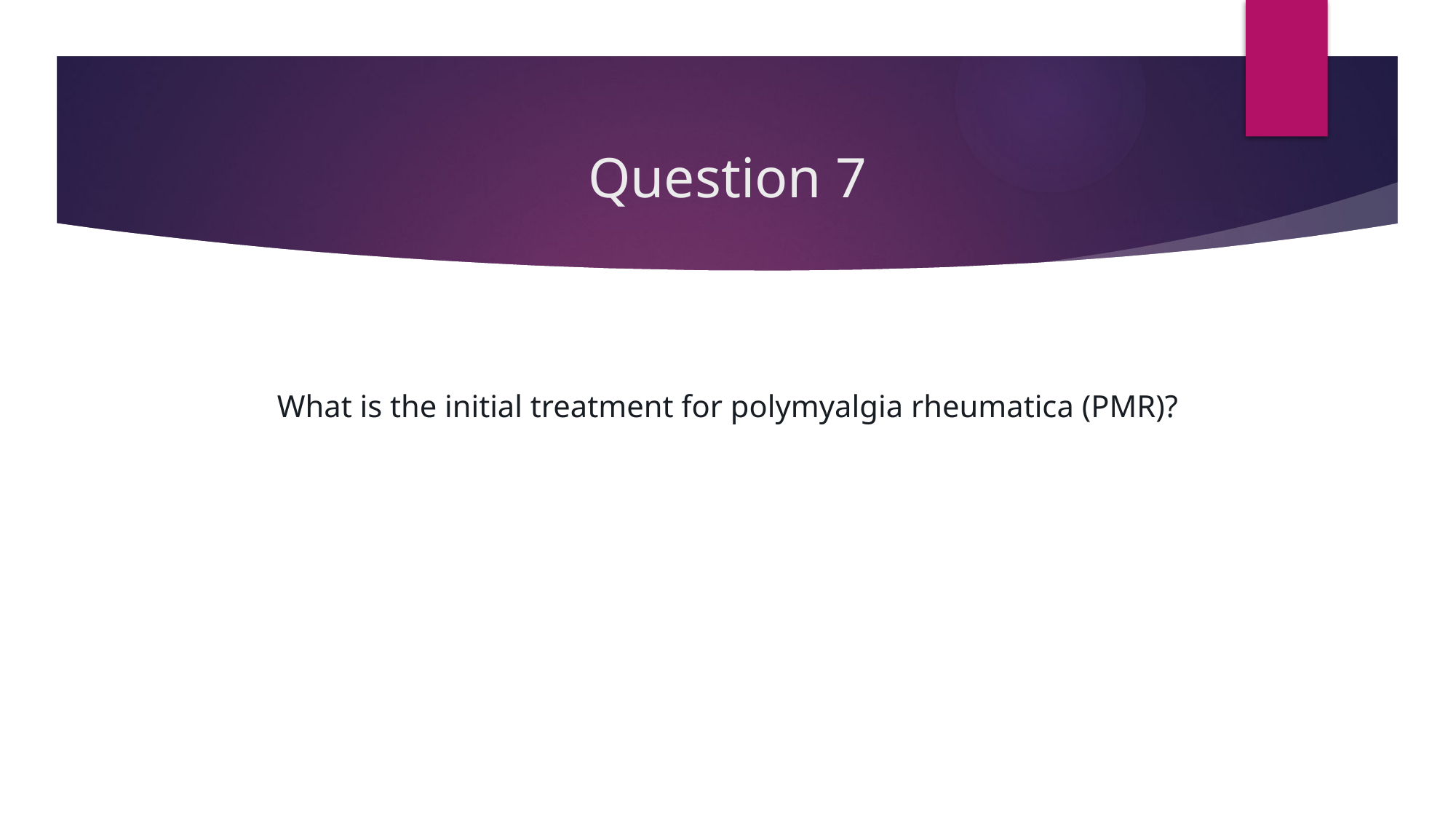

# Question 7
What is the initial treatment for polymyalgia rheumatica (PMR)?

## Slide 21
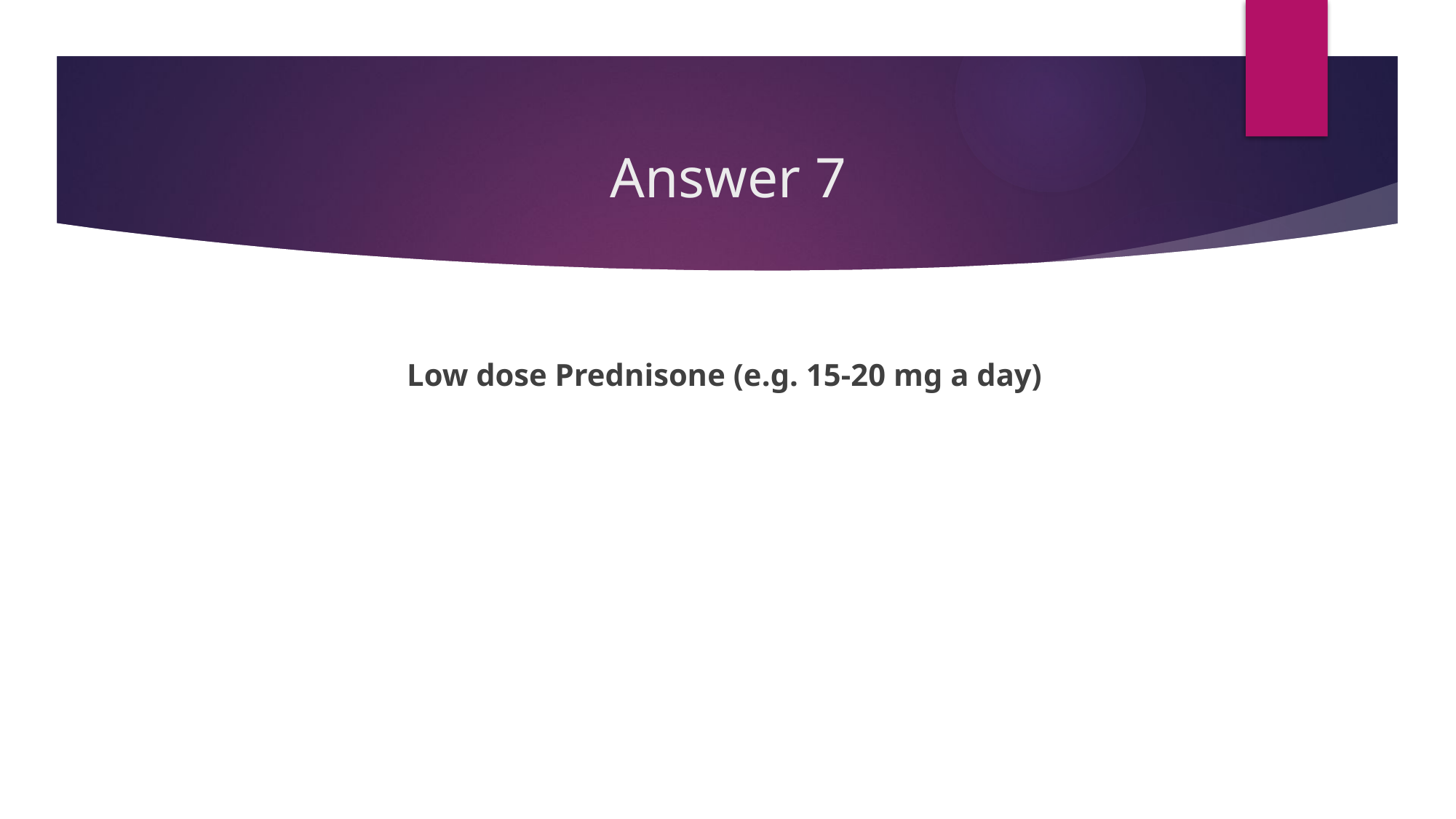

# Answer 7
Low dose Prednisone (e.g. 15-20 mg a day)

## Slide 22
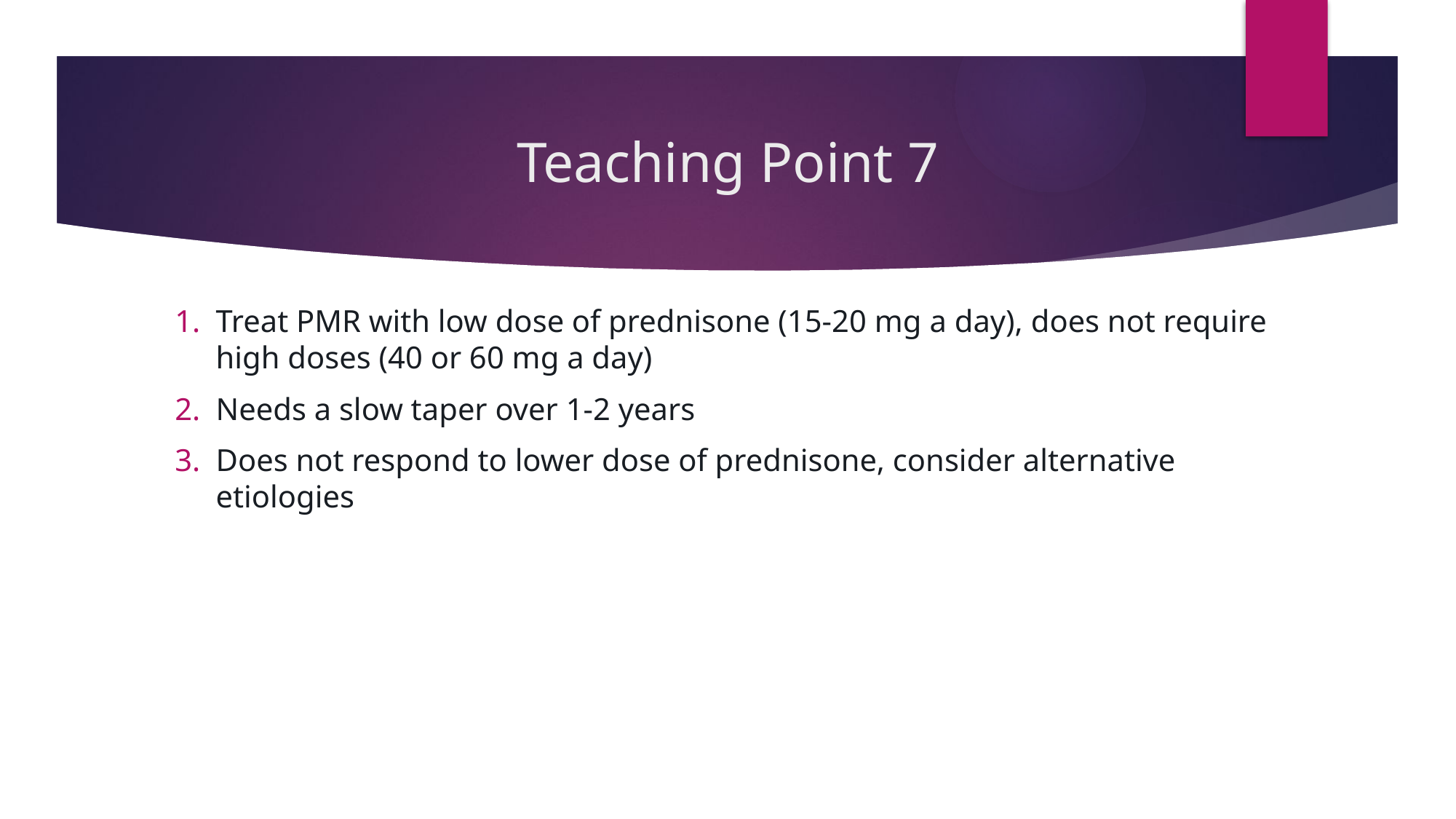

# Teaching Point 7
Treat PMR with low dose of prednisone (15-20 mg a day), does not require high doses (40 or 60 mg a day)
Needs a slow taper over 1-2 years
Does not respond to lower dose of prednisone, consider alternative etiologies

## Slide 23
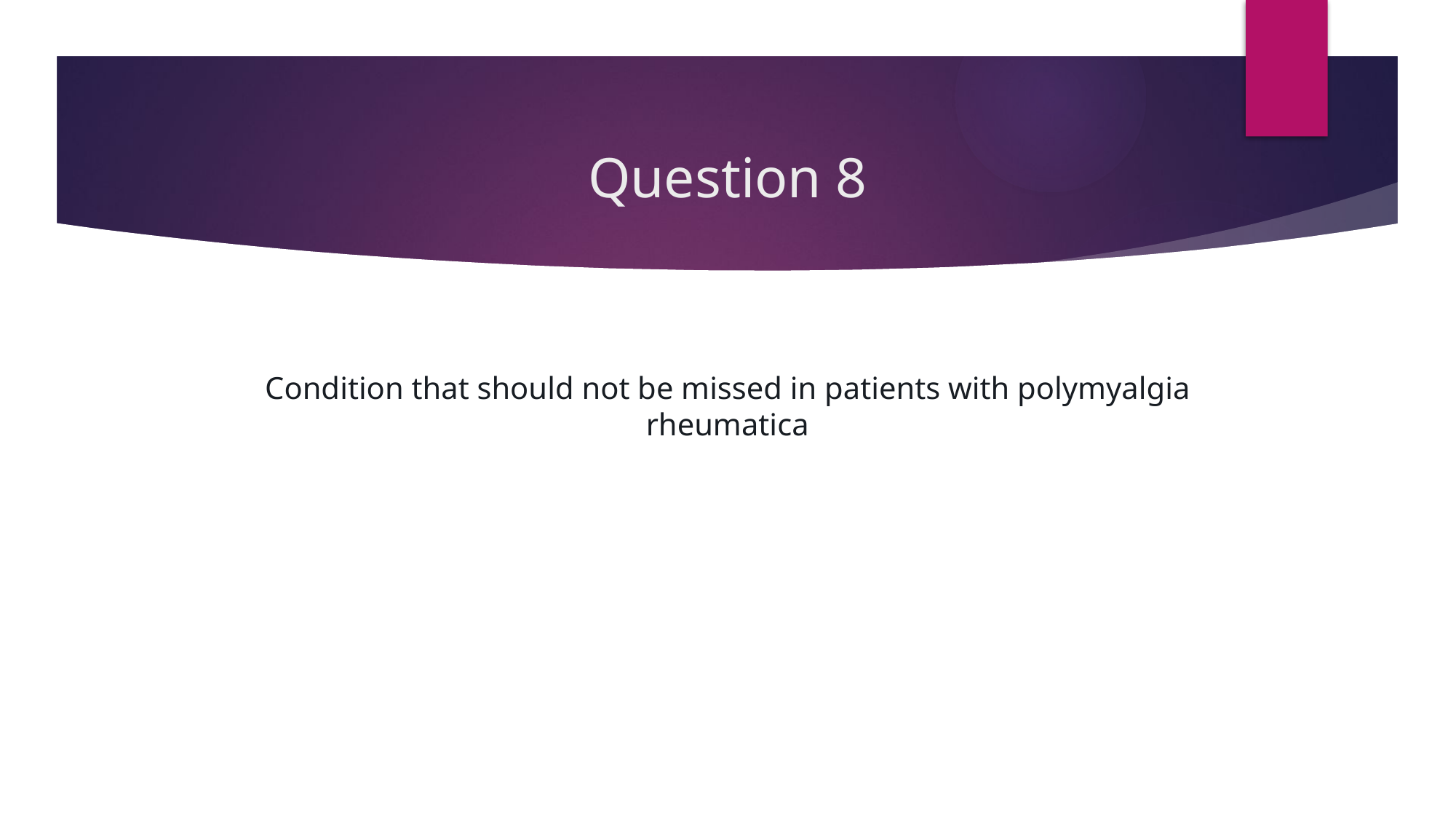

# Question 8
Condition that should not be missed in patients with polymyalgia rheumatica

## Slide 24
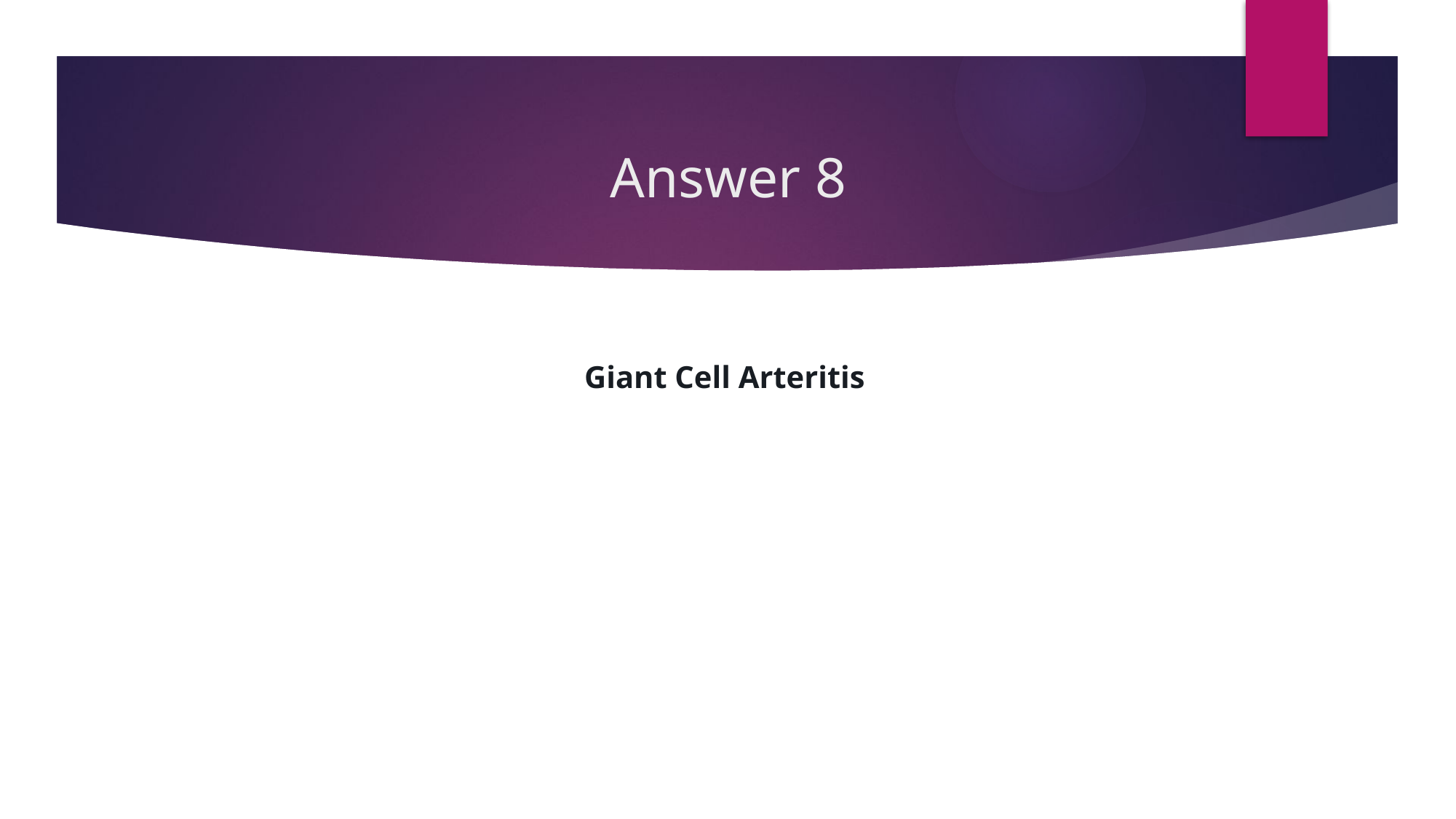

# Answer 8
Giant Cell Arteritis

## Slide 25
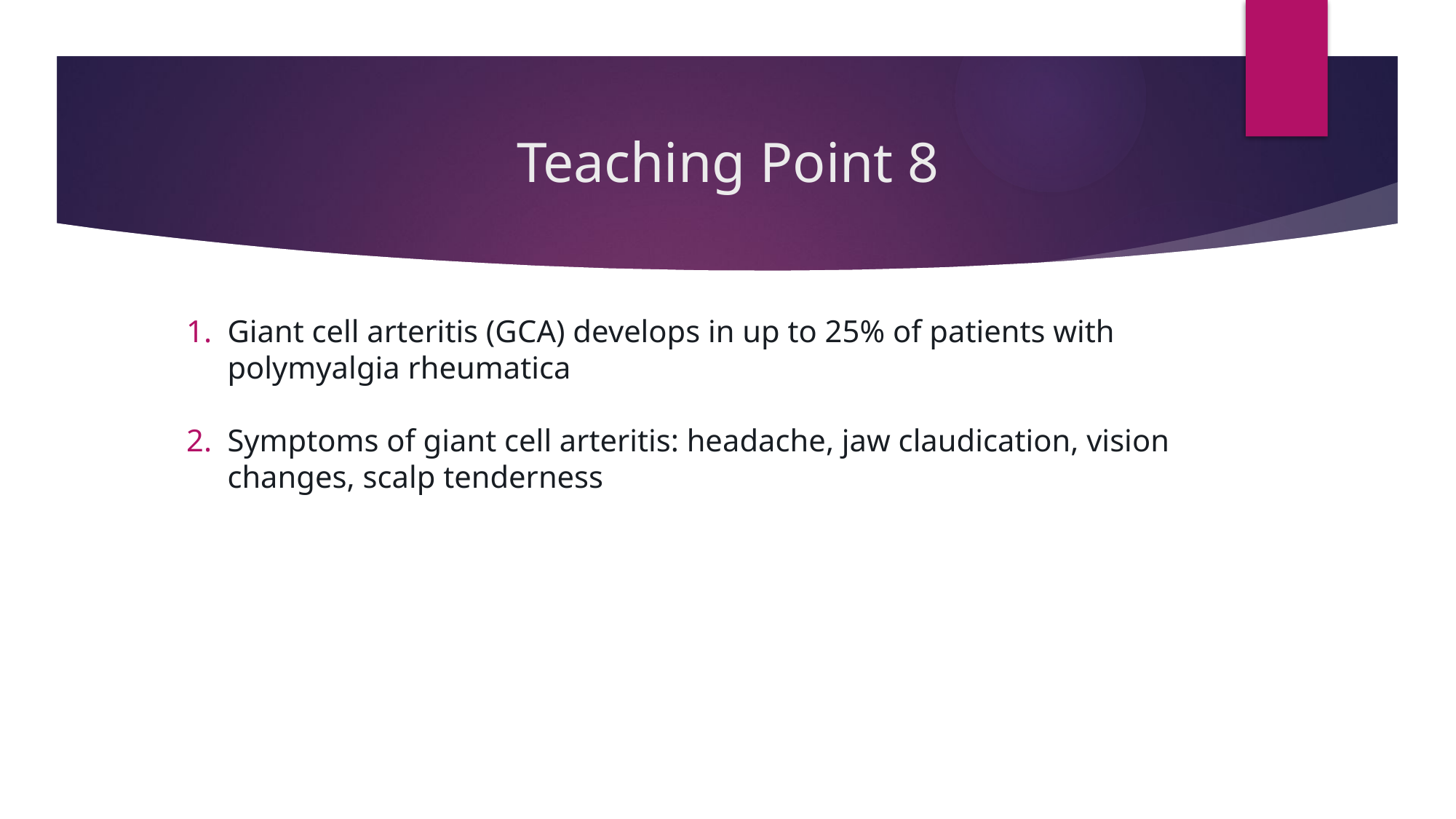

# Teaching Point 8
Giant cell arteritis (GCA) develops in up to 25% of patients with polymyalgia rheumatica
Symptoms of giant cell arteritis: headache, jaw claudication, vision changes, scalp tenderness

## Slide 26
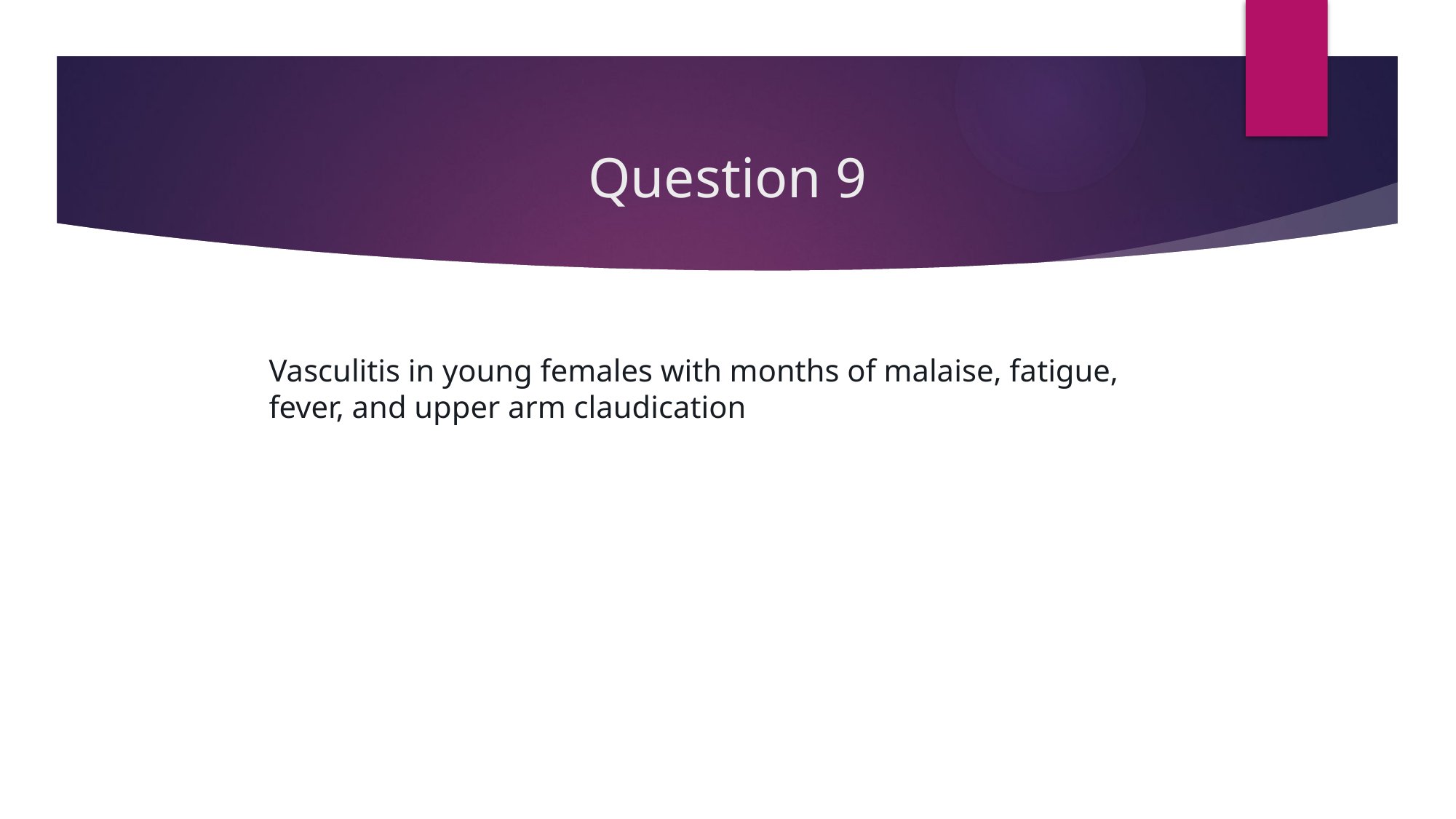

# Question 9
Vasculitis in young females with months of malaise, fatigue, fever, and upper arm claudication

## Slide 27
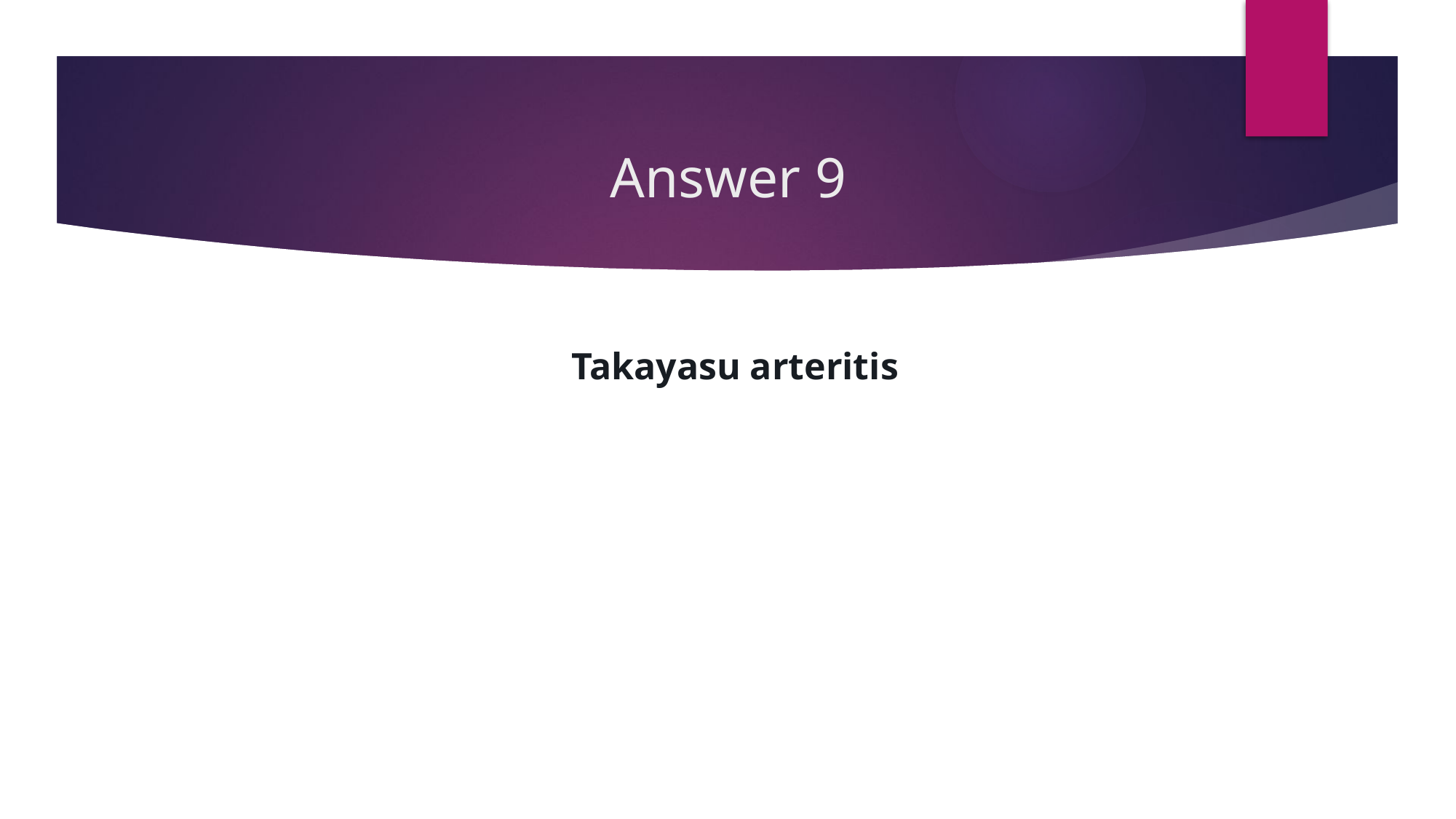

# Answer 9
Takayasu arteritis

## Slide 28
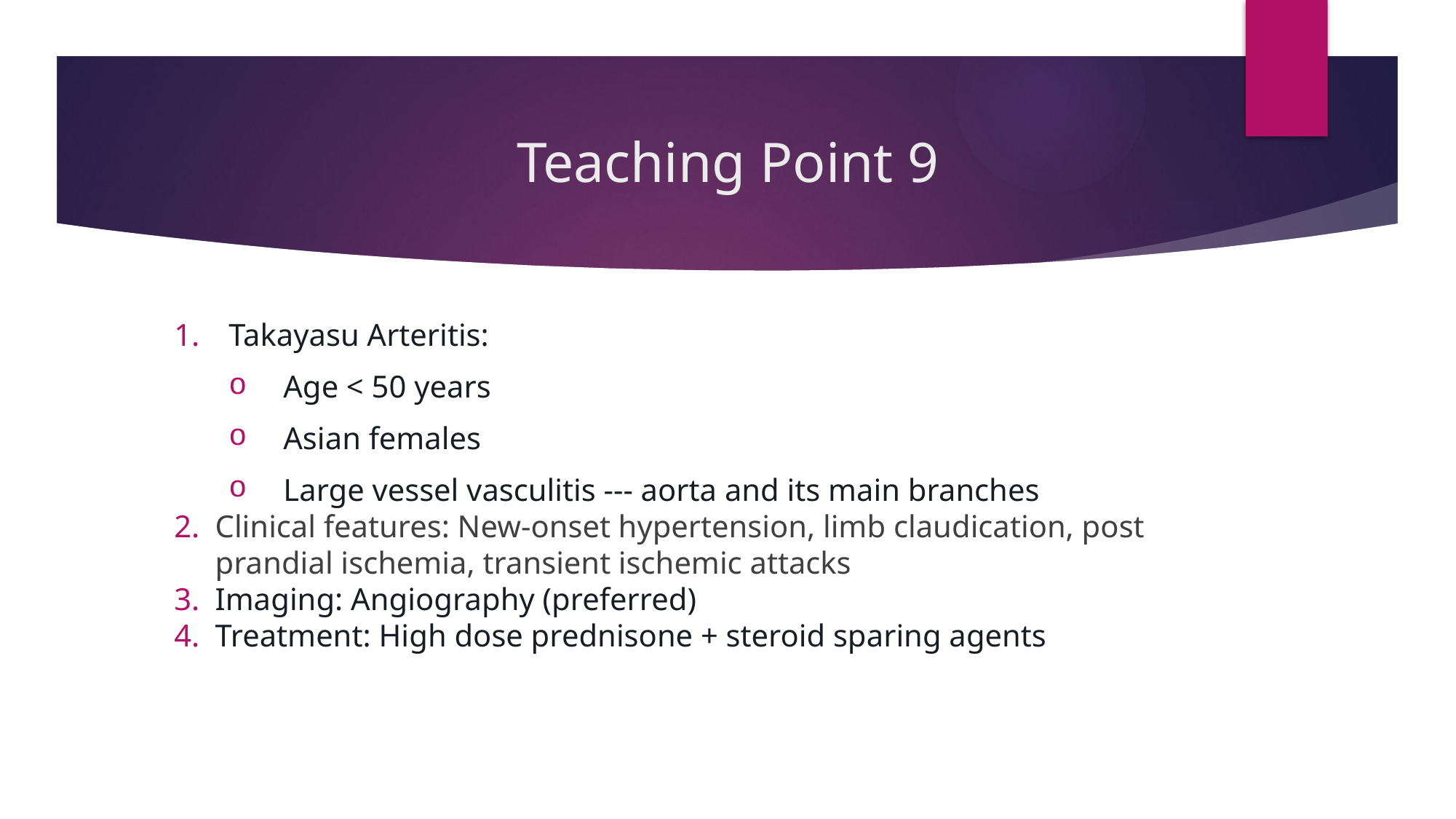

# Teaching Point 9
Takayasu Arteritis:
Age < 50 years
Asian females
Large vessel vasculitis --- aorta and its main branches
Clinical features: New-onset hypertension, limb claudication, post prandial ischemia, transient ischemic attacks
Imaging: Angiography (preferred)
Treatment: High dose prednisone + steroid sparing agents

## Slide 29
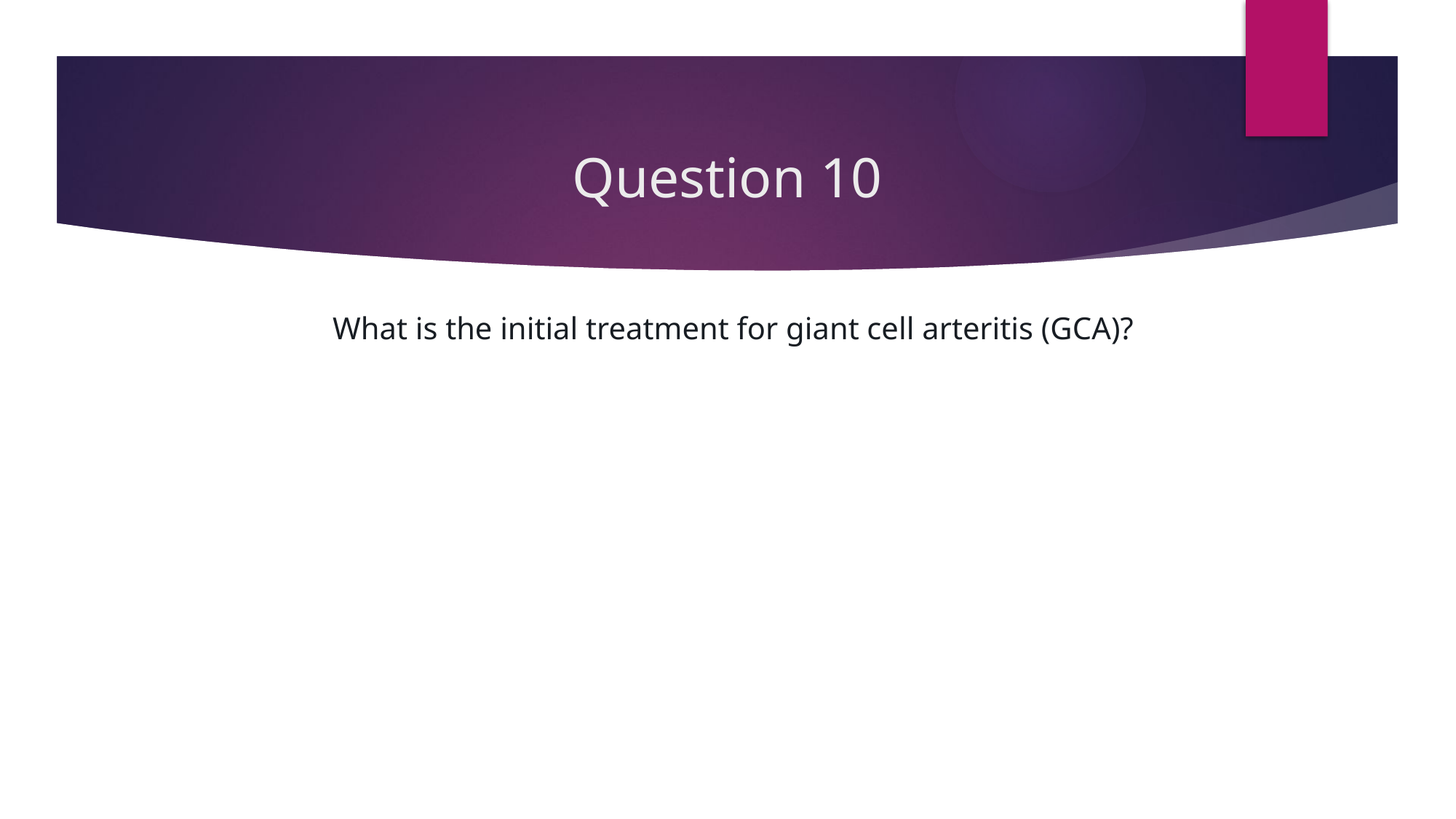

# Question 10
What is the initial treatment for giant cell arteritis (GCA)?

## Slide 30
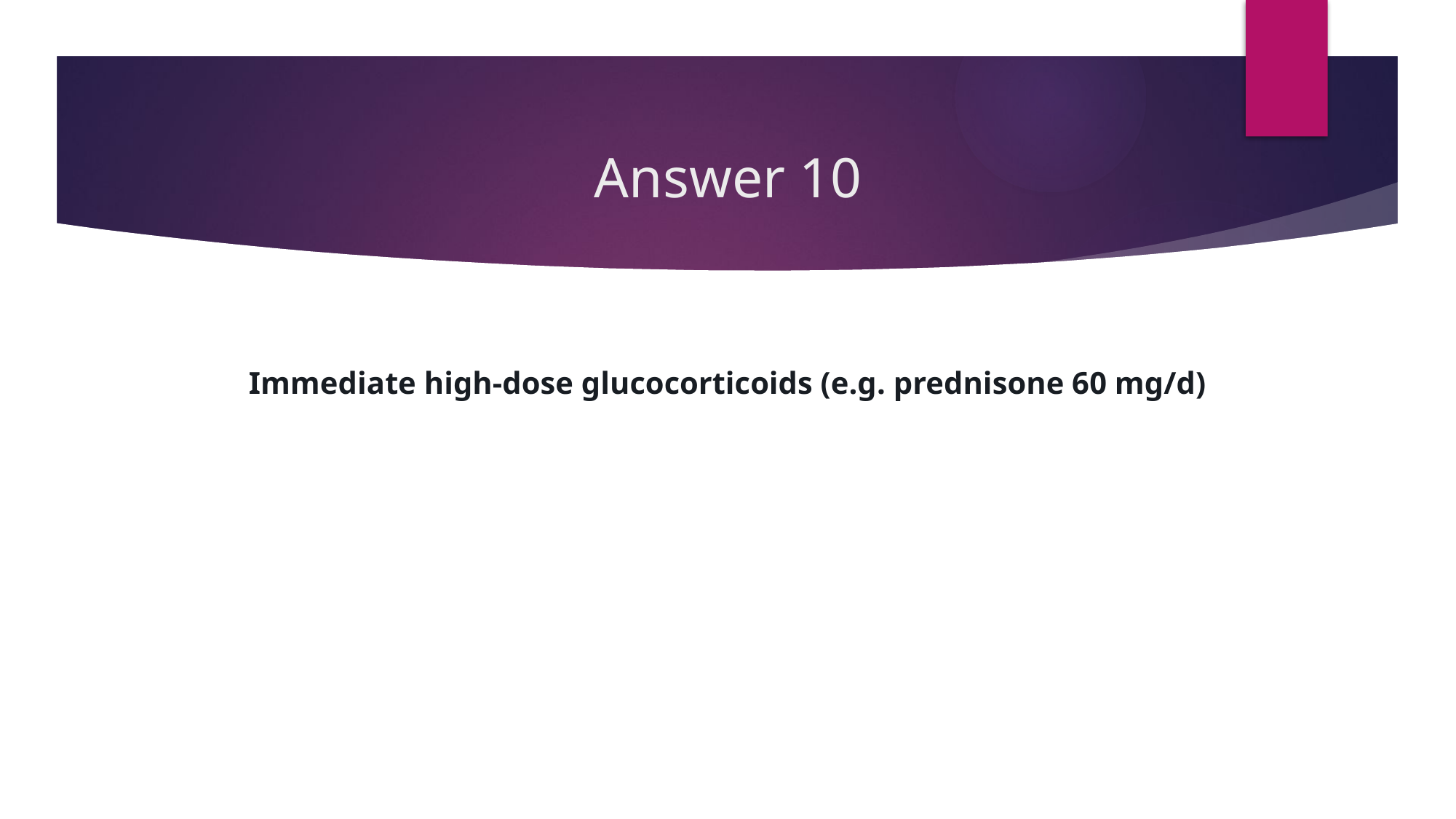

# Answer 10
Immediate high-dose glucocorticoids (e.g. prednisone 60 mg/d)

## Slide 31
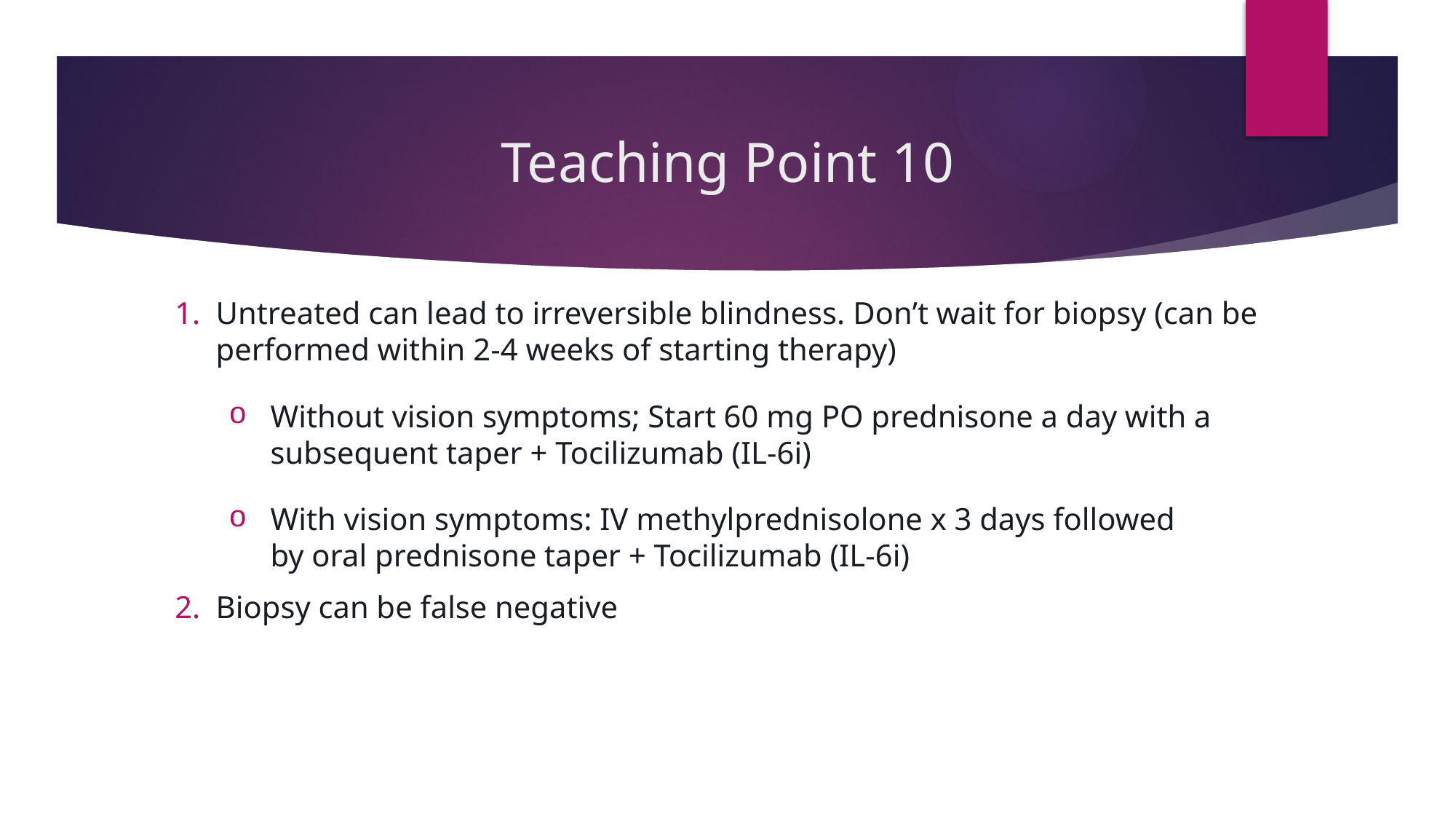

# Teaching Point 10
Untreated can lead to irreversible blindness. Don’t wait for biopsy (can be performed within 2-4 weeks of starting therapy)
Without vision symptoms; Start 60 mg PO prednisone a day with a subsequent taper + Tocilizumab (IL-6i)
With vision symptoms: IV methylprednisolone x 3 days followed by oral prednisone taper + Tocilizumab (IL-6i)
Biopsy can be false negative

## Slide 32
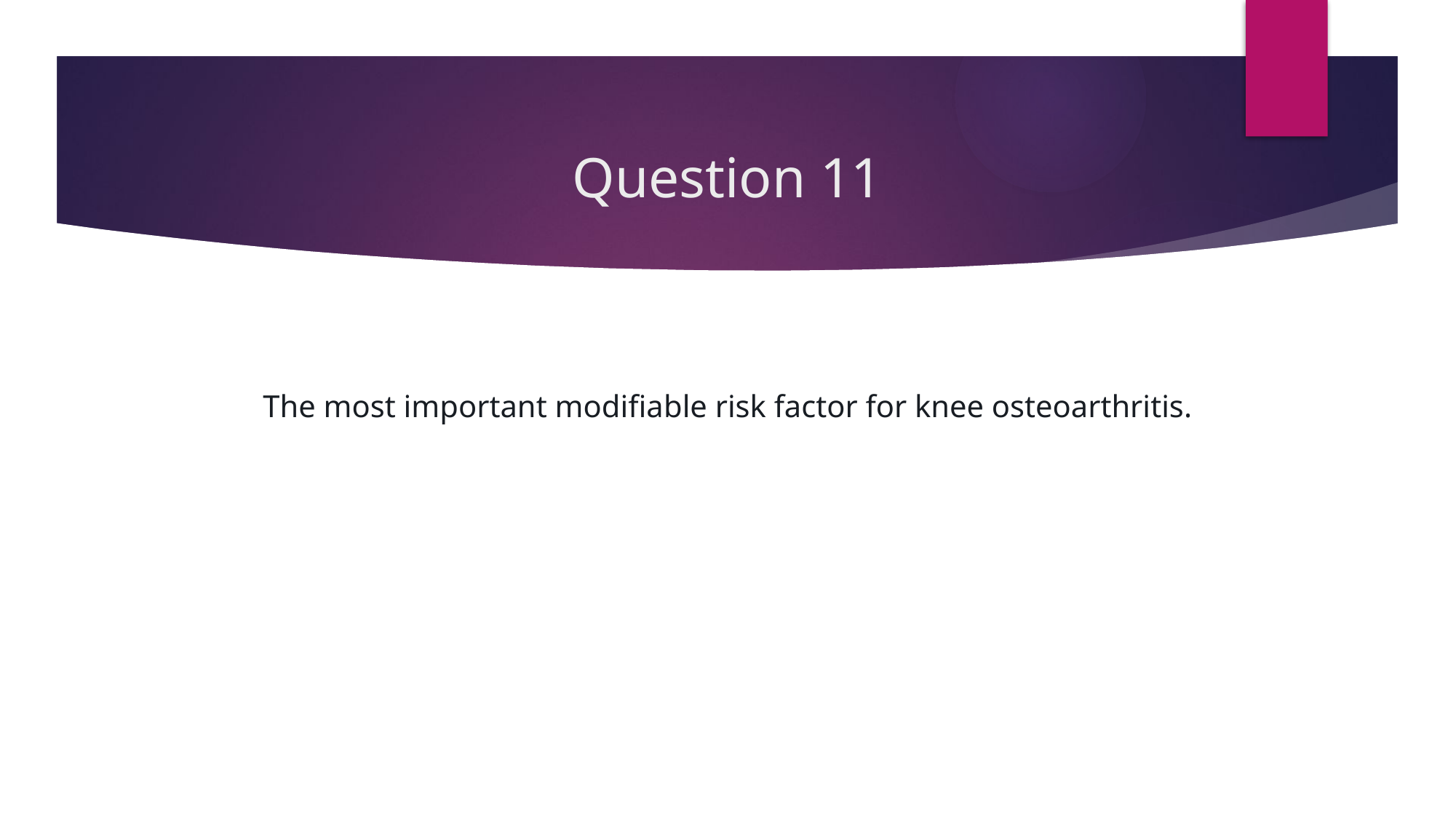

# Question 11
The most important modifiable risk factor for knee osteoarthritis.

## Slide 33
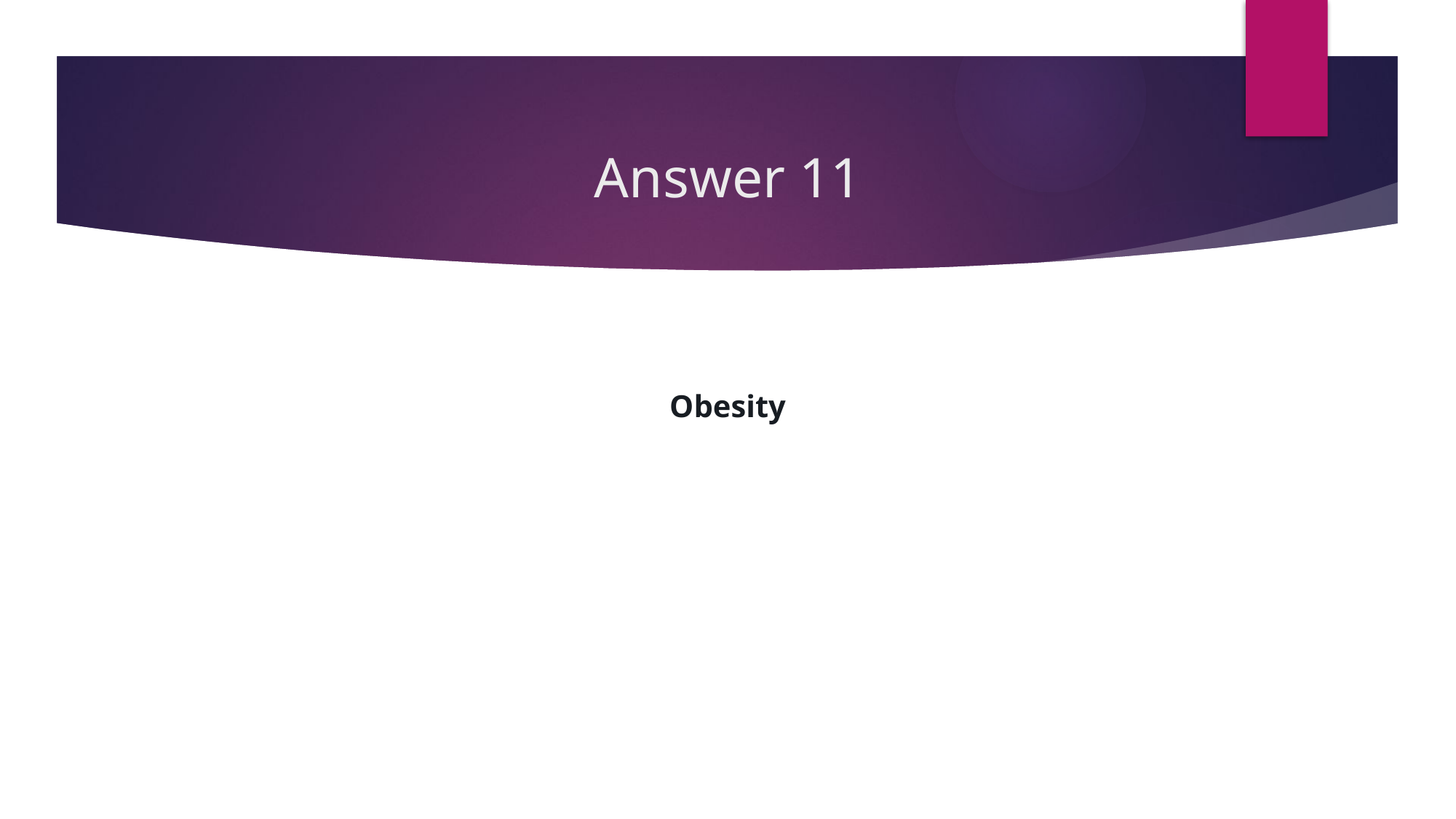

# Answer 11
Obesity

## Slide 34
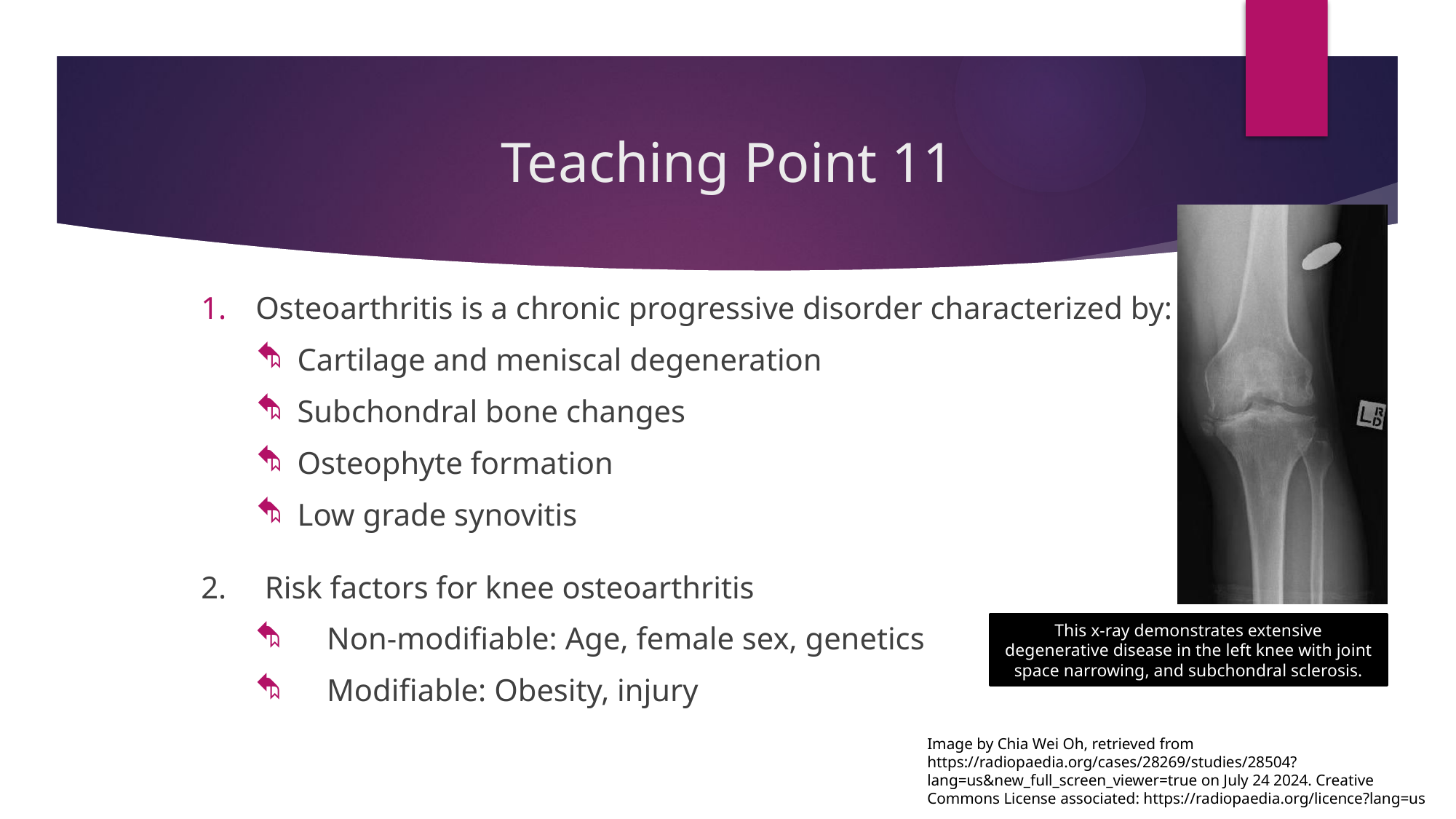

# Teaching Point 11
Osteoarthritis is a chronic progressive disorder characterized by:
Cartilage and meniscal degeneration
Subchondral bone changes
Osteophyte formation
Low grade synovitis
2.  Risk factors for knee osteoarthritis
 Non-modifiable: Age, female sex, genetics
 Modifiable: Obesity, injury
This x-ray demonstrates extensive degenerative disease in the left knee with joint space narrowing, and subchondral sclerosis.
Image by Chia Wei Oh, retrieved from https://radiopaedia.org/cases/28269/studies/28504?lang=us&new_full_screen_viewer=true on July 24 2024. Creative Commons License associated: https://radiopaedia.org/licence?lang=us

## Slide 35
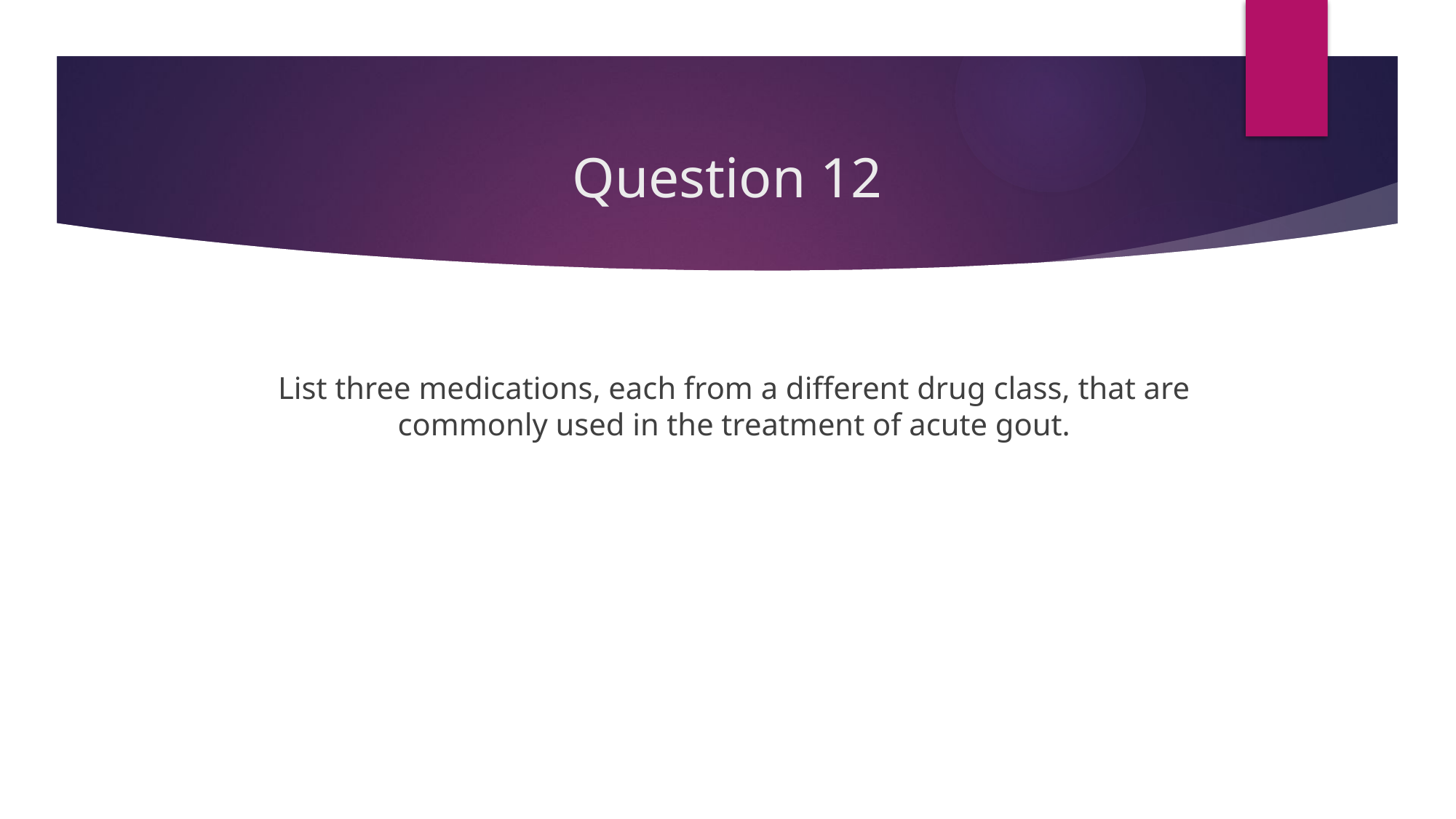

# Question 12
List three medications, each from a different drug class, that are commonly used in the treatment of acute gout.

## Slide 36
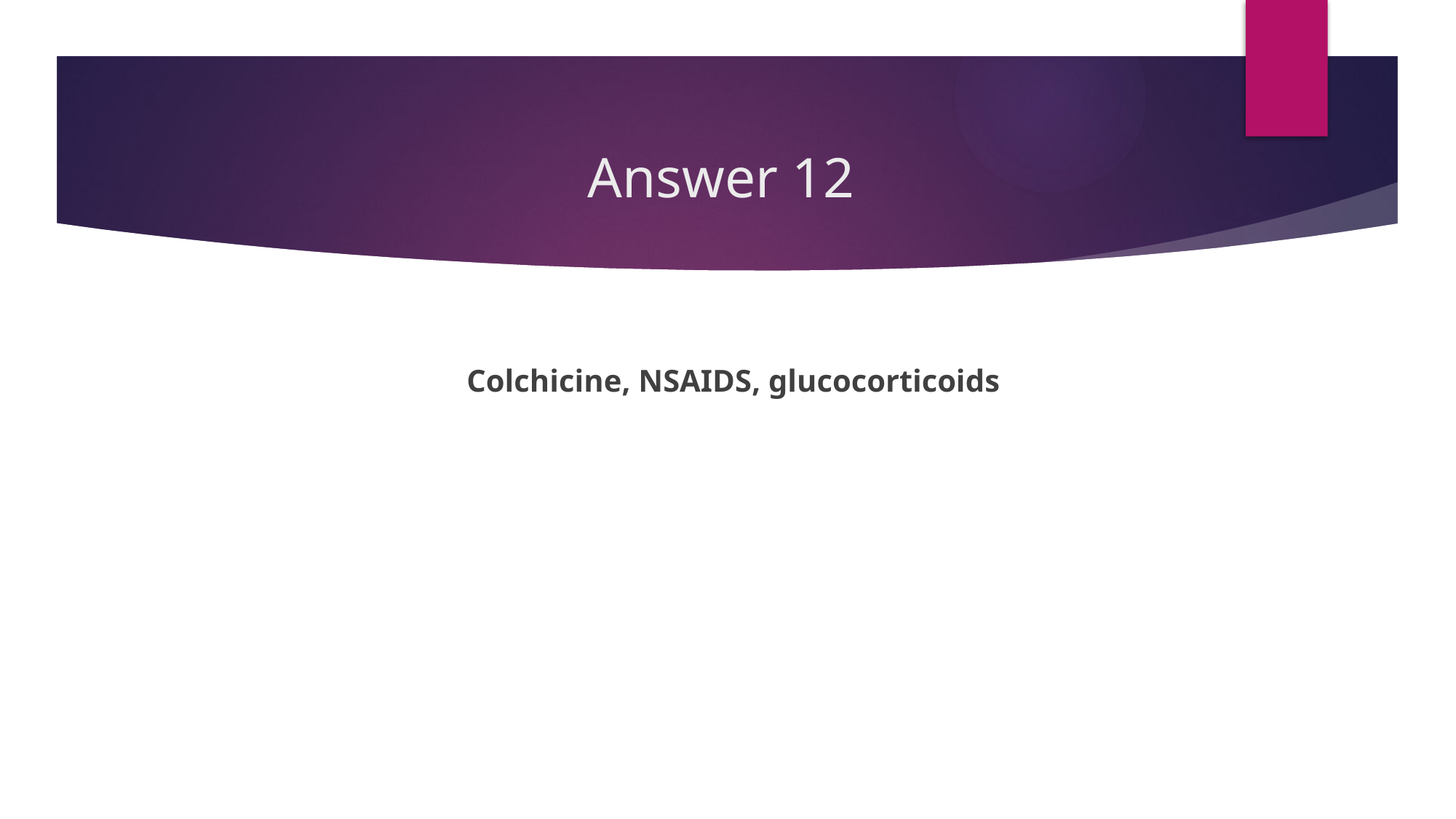

# Answer 12
Colchicine, NSAIDS, glucocorticoids

## Slide 37
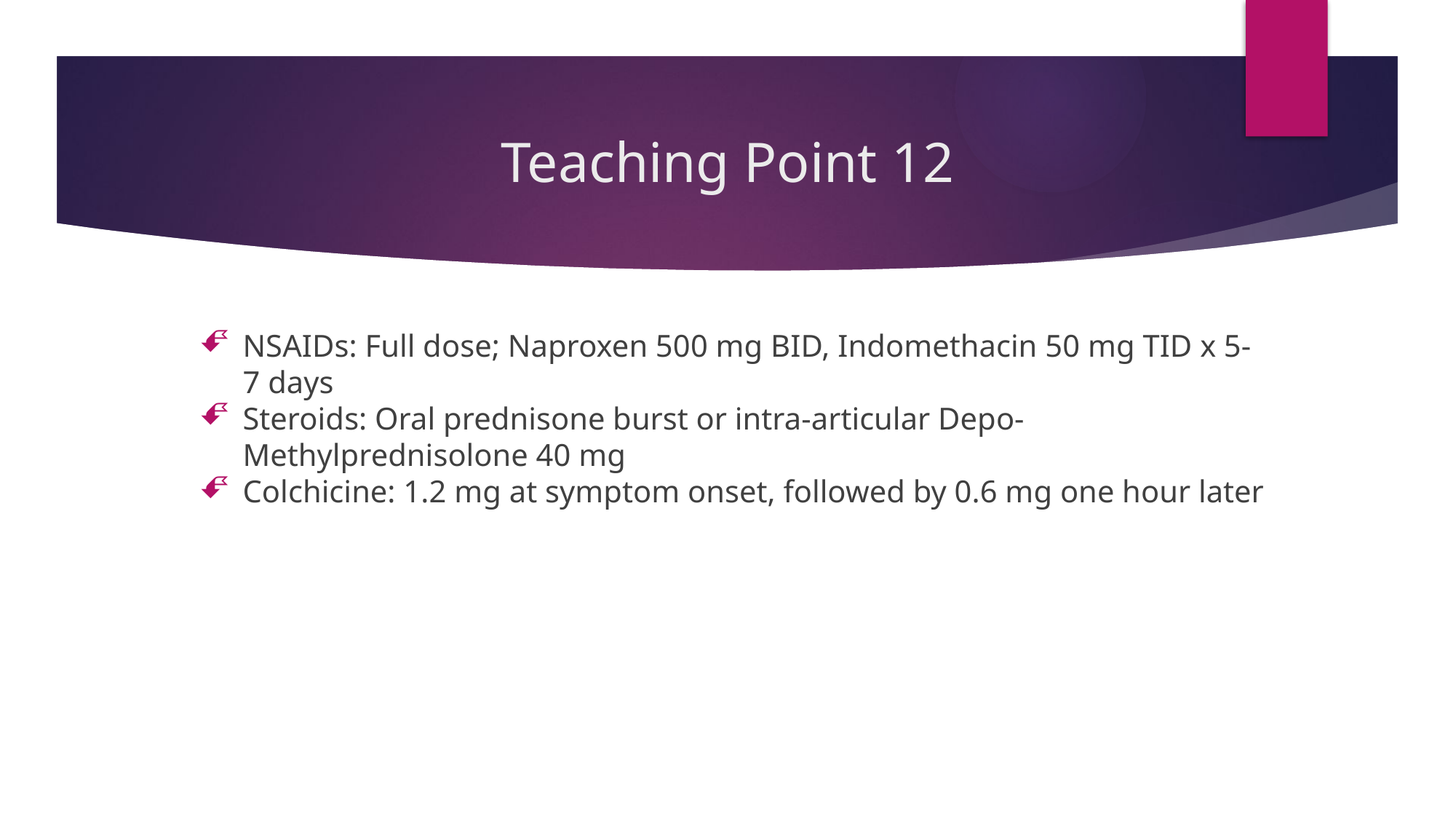

# Teaching Point 12
NSAIDs: Full dose; Naproxen 500 mg BID, Indomethacin 50 mg TID x 5-7 days
Steroids: Oral prednisone burst or intra-articular Depo-Methylprednisolone 40 mg
Colchicine: 1.2 mg at symptom onset, followed by 0.6 mg one hour later

## Slide 38
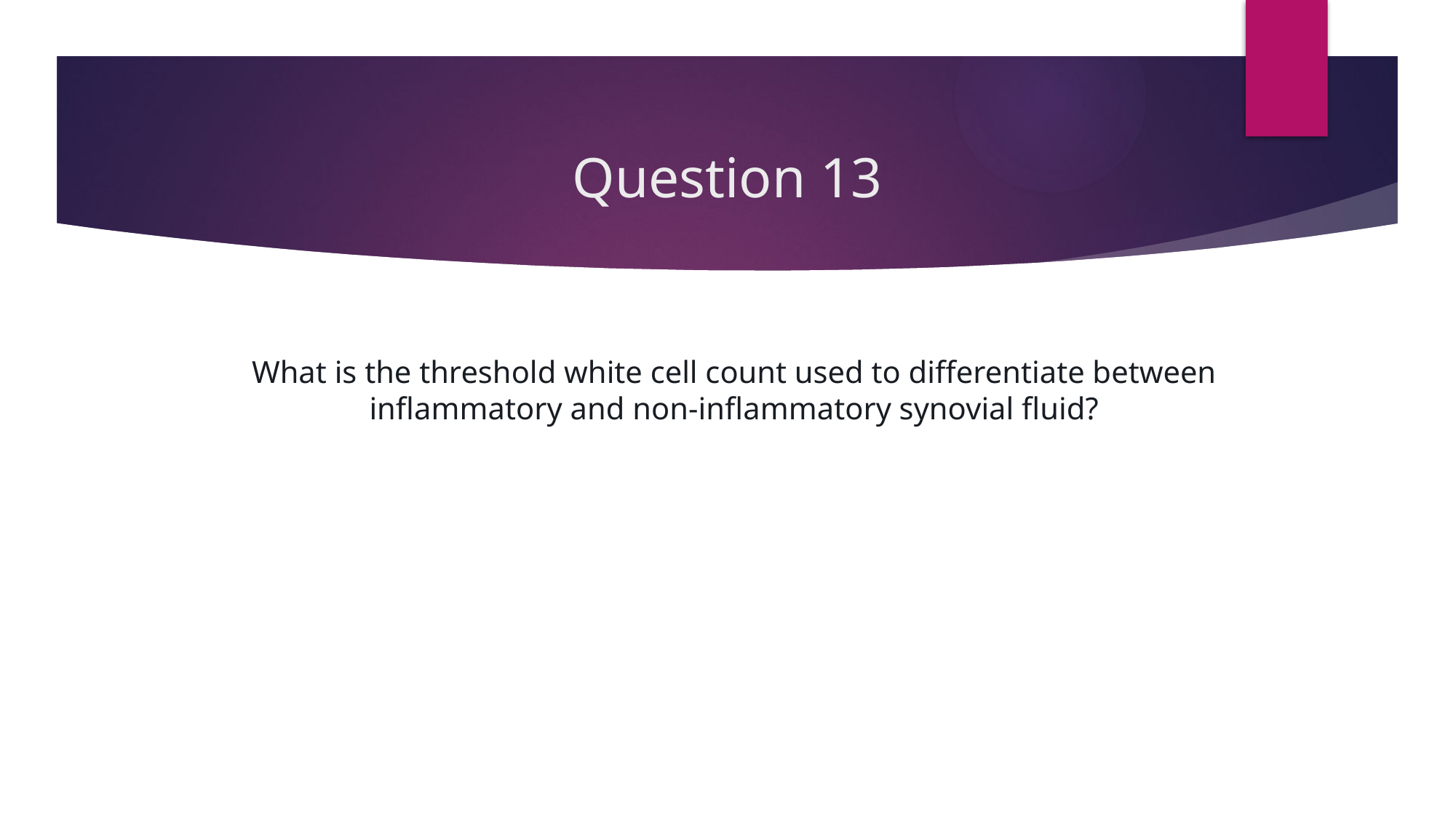

# Question 13
What is the threshold white cell count used to differentiate between inflammatory and non-inflammatory synovial fluid?

## Slide 39
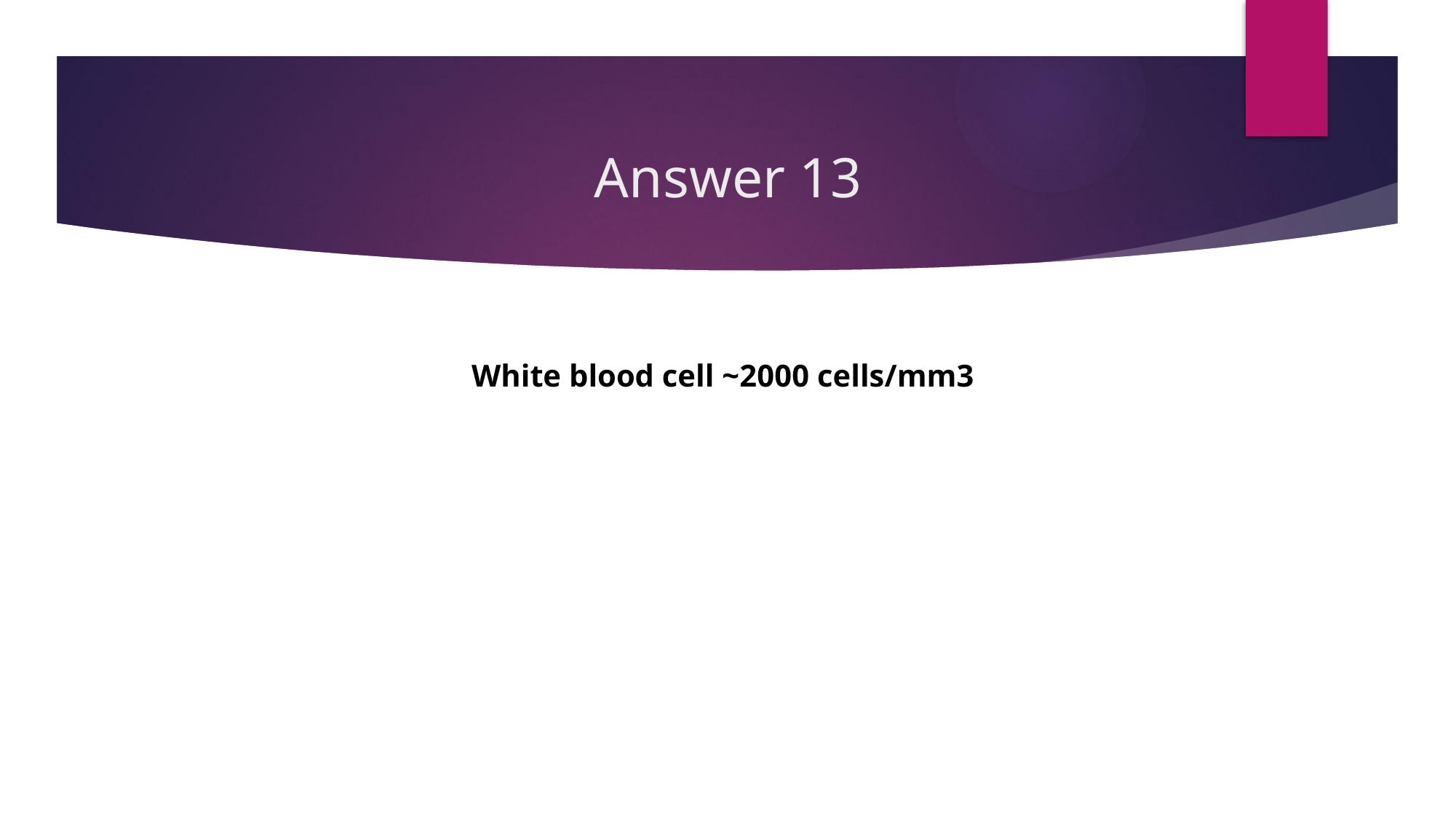

# Answer 13
White blood cell ~2000 cells/mm3

## Slide 40
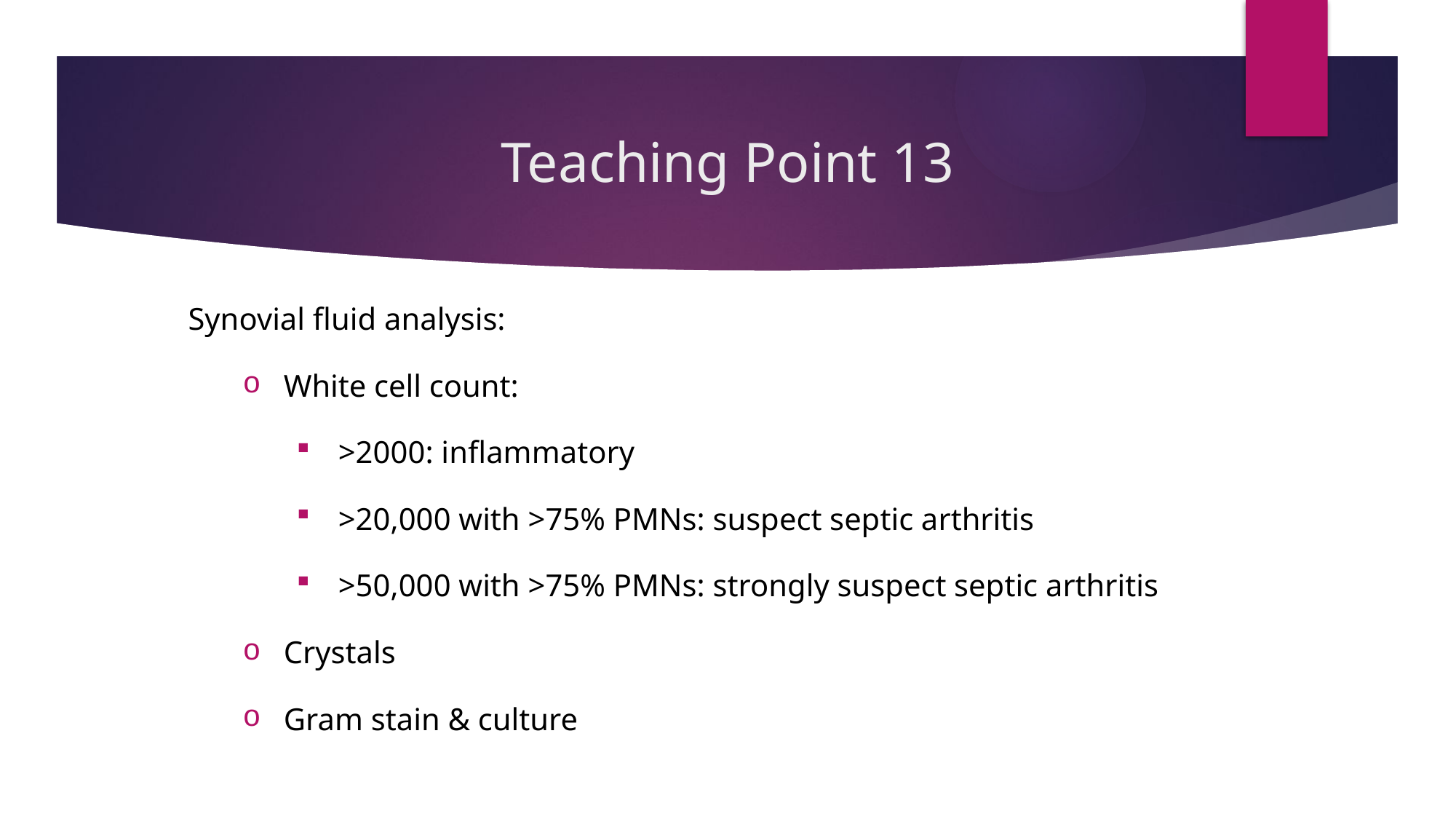

# Teaching Point 13
Synovial fluid analysis:
White cell count:
>2000: inflammatory
>20,000 with >75% PMNs: suspect septic arthritis
>50,000 with >75% PMNs: strongly suspect septic arthritis
Crystals
Gram stain & culture

## Slide 41
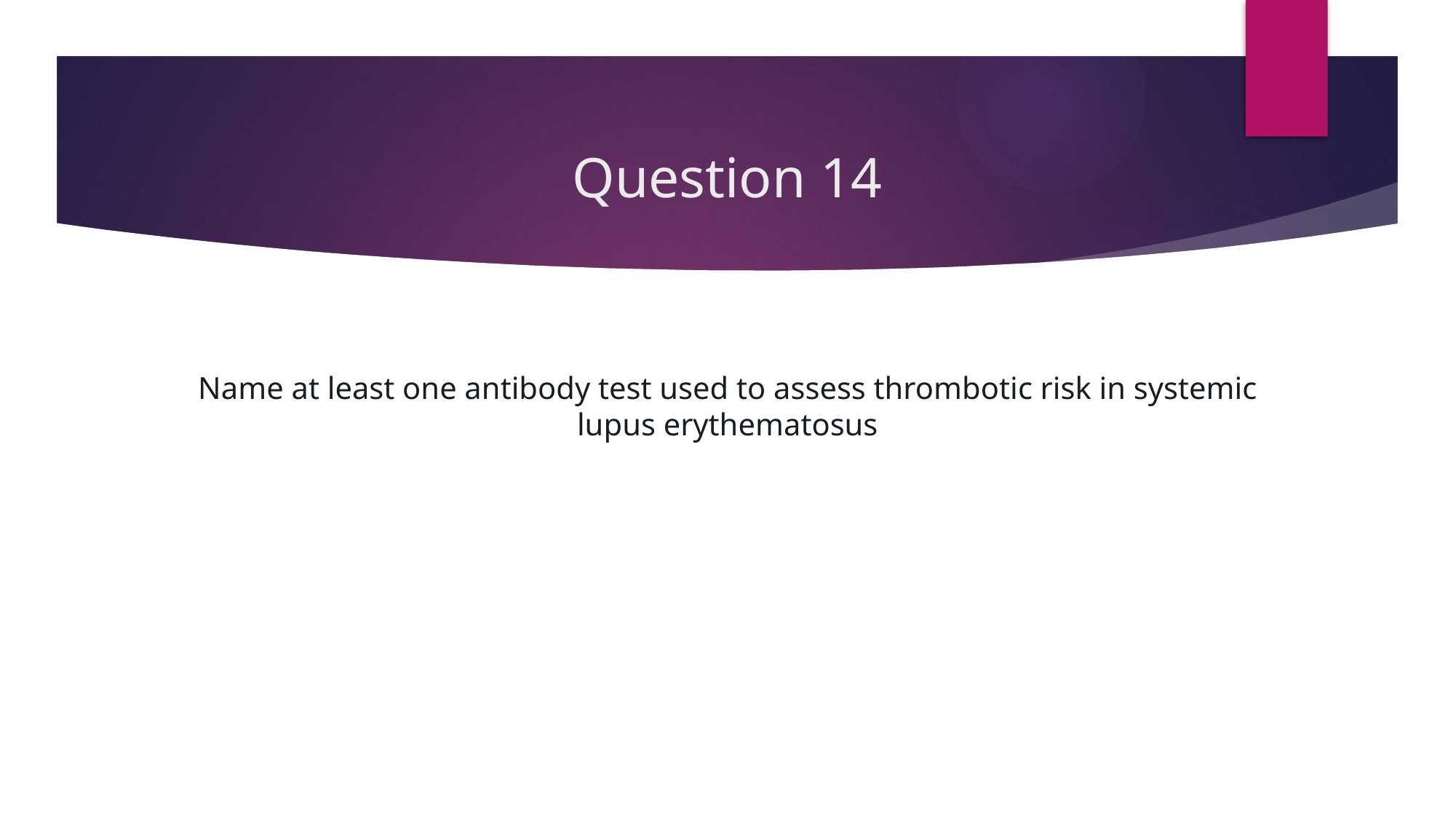

# Question 14
Name at least one antibody test used to assess thrombotic risk in systemic lupus erythematosus

## Slide 42
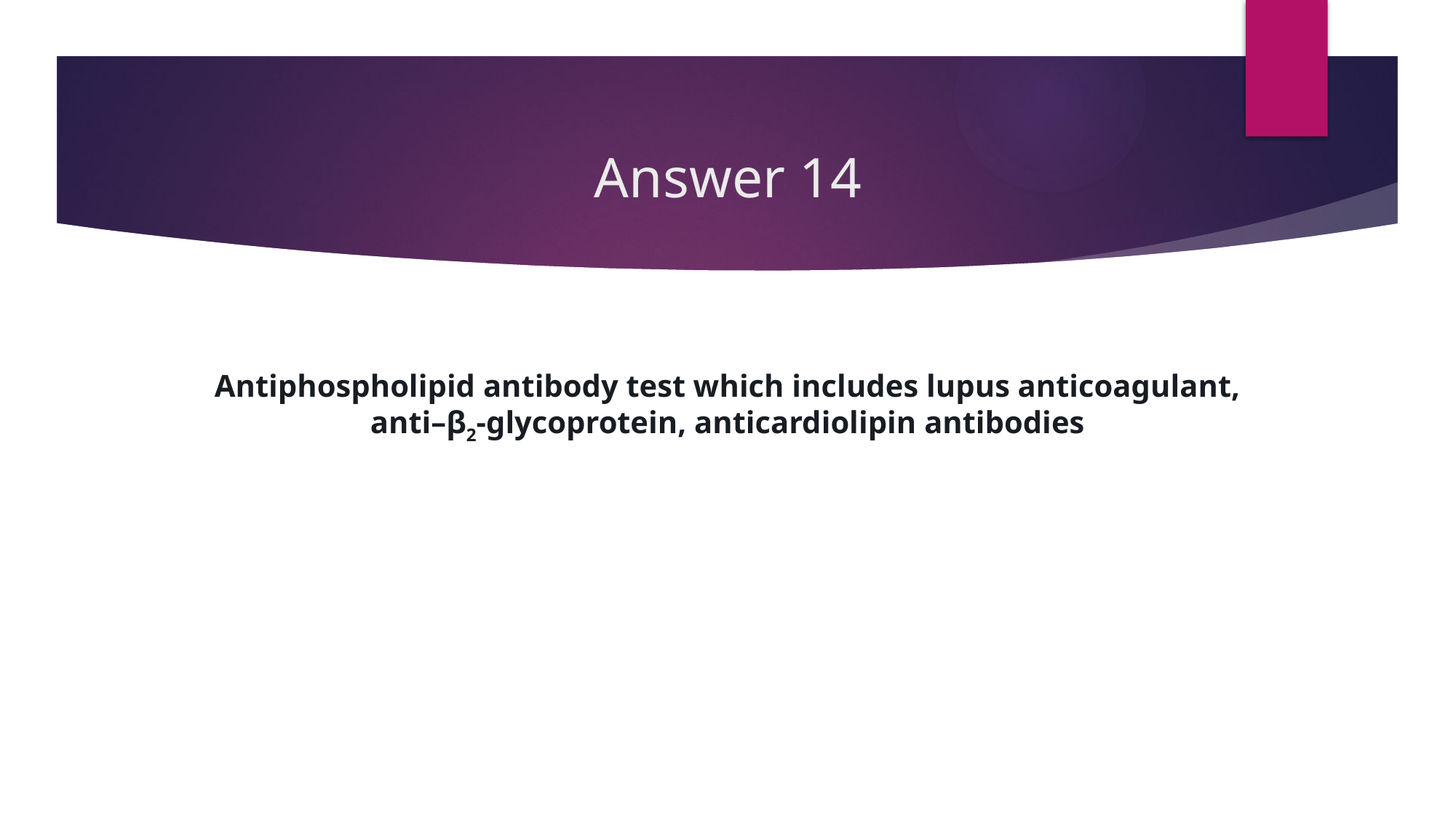

# Answer 14
Antiphospholipid antibody test which includes lupus anticoagulant, anti–β2-glycoprotein, anticardiolipin antibodies

## Slide 43
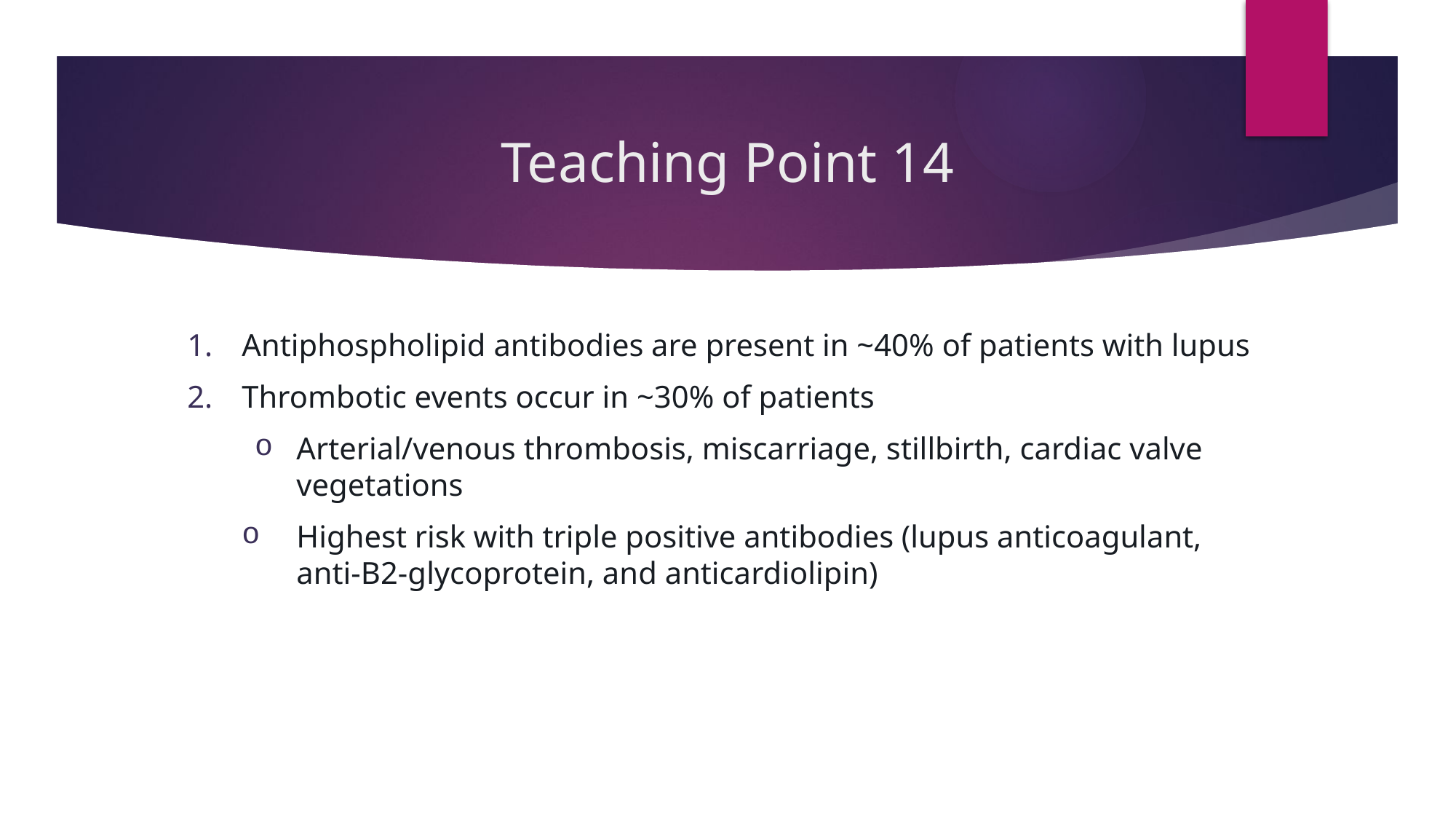

# Teaching Point 14
Antiphospholipid antibodies are present in ~40% of patients with lupus
Thrombotic events occur in ~30% of patients
Arterial/venous thrombosis, miscarriage, stillbirth, cardiac valve vegetations
Highest risk with triple positive antibodies (lupus anticoagulant, anti-B2-glycoprotein, and anticardiolipin)

## Slide 44
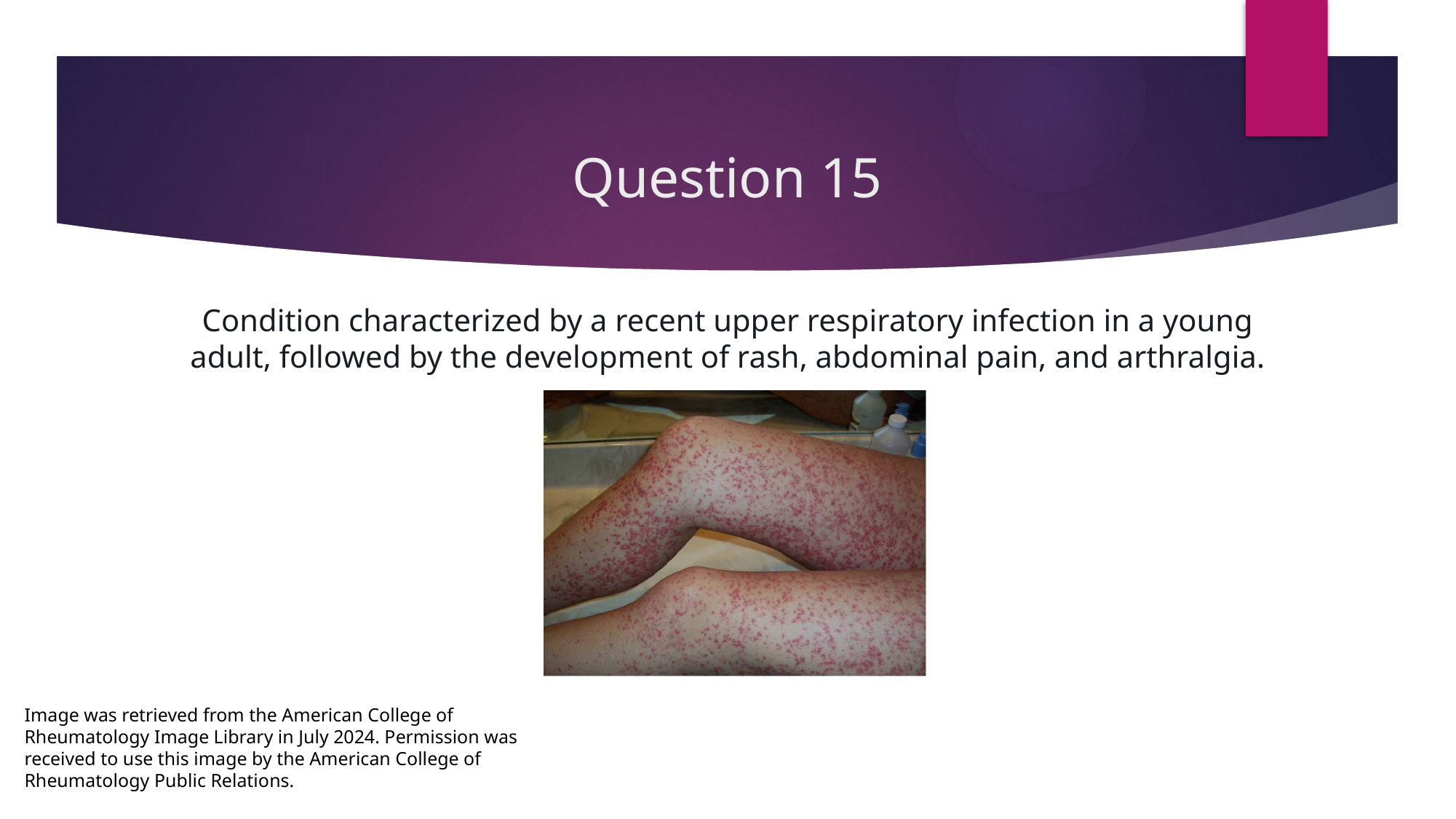

# Question 15
Condition characterized by a recent upper respiratory infection in a young adult, followed by the development of rash, abdominal pain, and arthralgia.
Image was retrieved from the American College of Rheumatology Image Library in July 2024. Permission was received to use this image by the American College of Rheumatology Public Relations.

## Slide 45
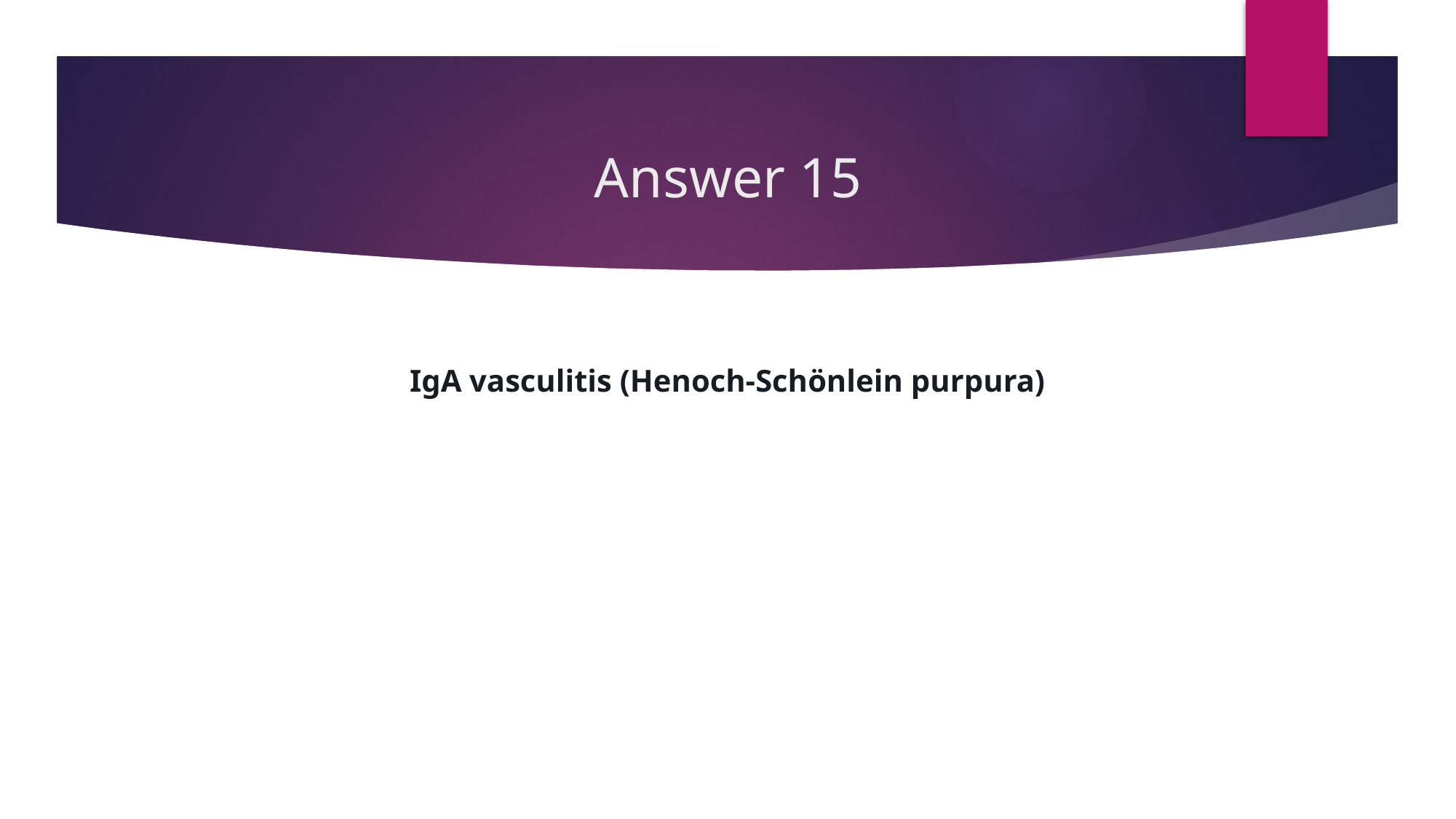

# Answer 15
IgA vasculitis (Henoch-Schönlein purpura)

## Slide 46
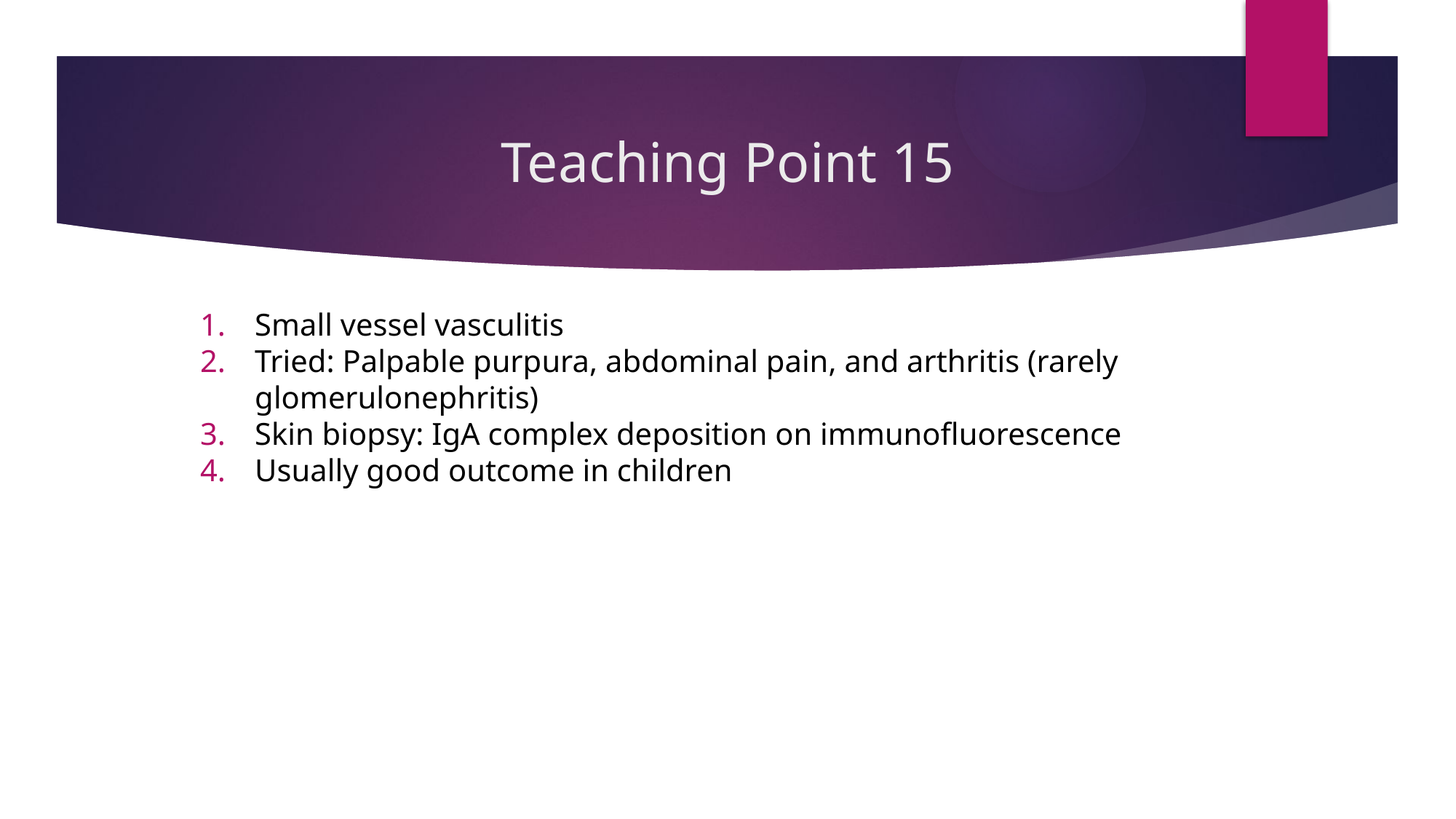

# Teaching Point 15
Small vessel vasculitis
Tried: Palpable purpura, abdominal pain, and arthritis (rarely glomerulonephritis)
Skin biopsy: IgA complex deposition on immunofluorescence
Usually good outcome in children

## Slide 47
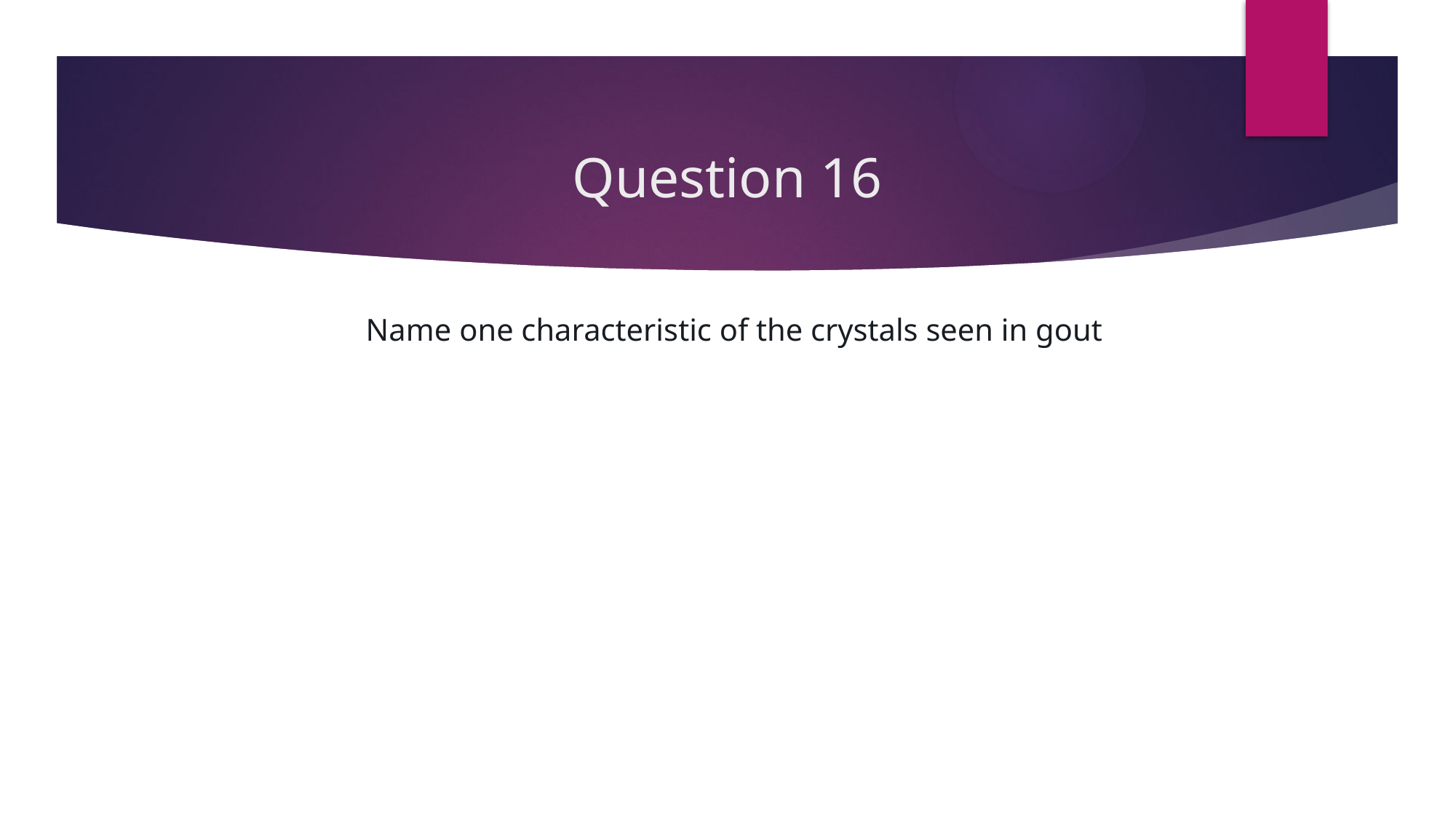

# Question 16
Name one characteristic of the crystals seen in gout

## Slide 48
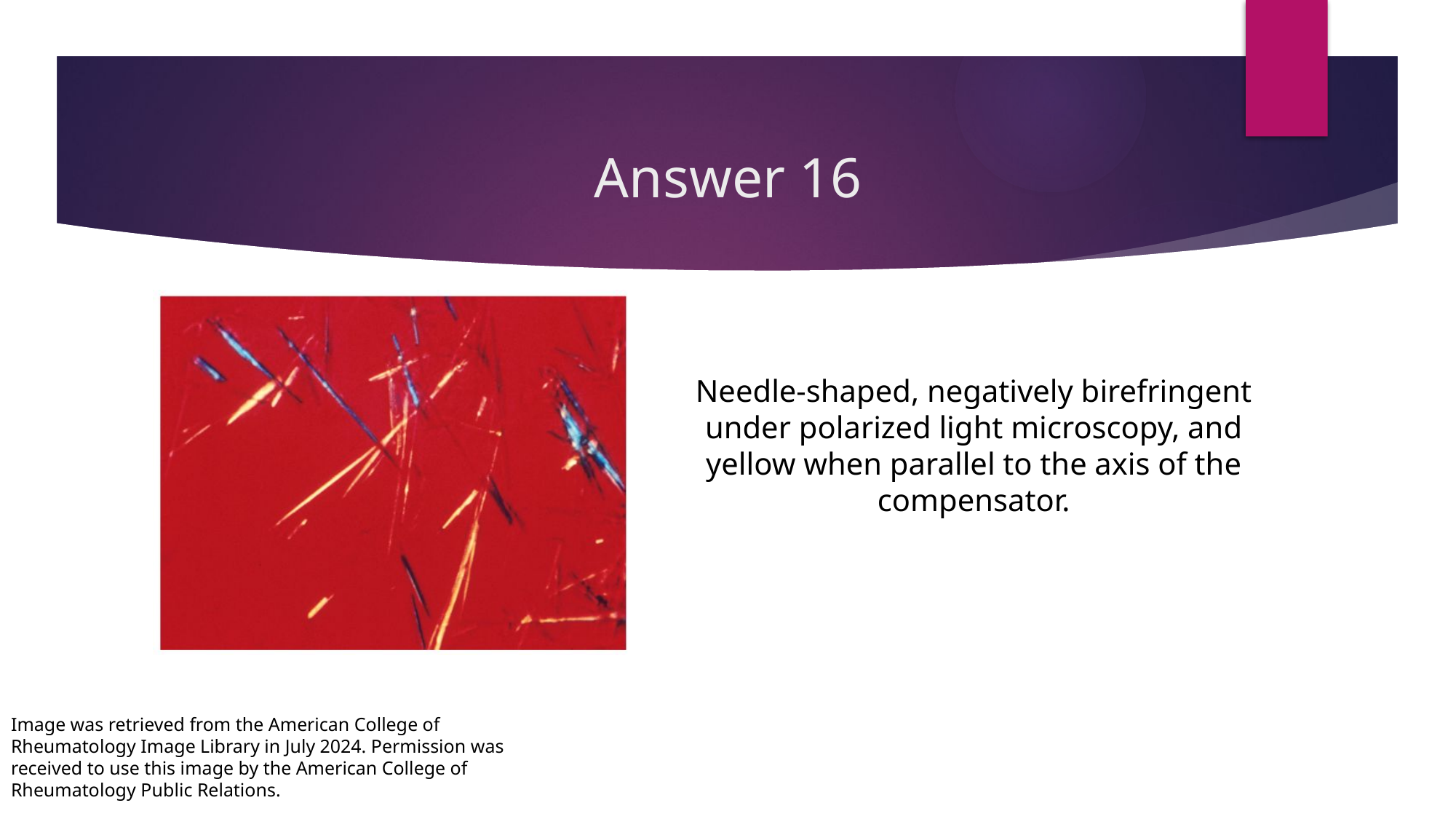

# Answer 16
Needle-shaped, negatively birefringent under polarized light microscopy, and yellow when parallel to the axis of the compensator.
Image was retrieved from the American College of Rheumatology Image Library in July 2024. Permission was received to use this image by the American College of Rheumatology Public Relations.

## Slide 49
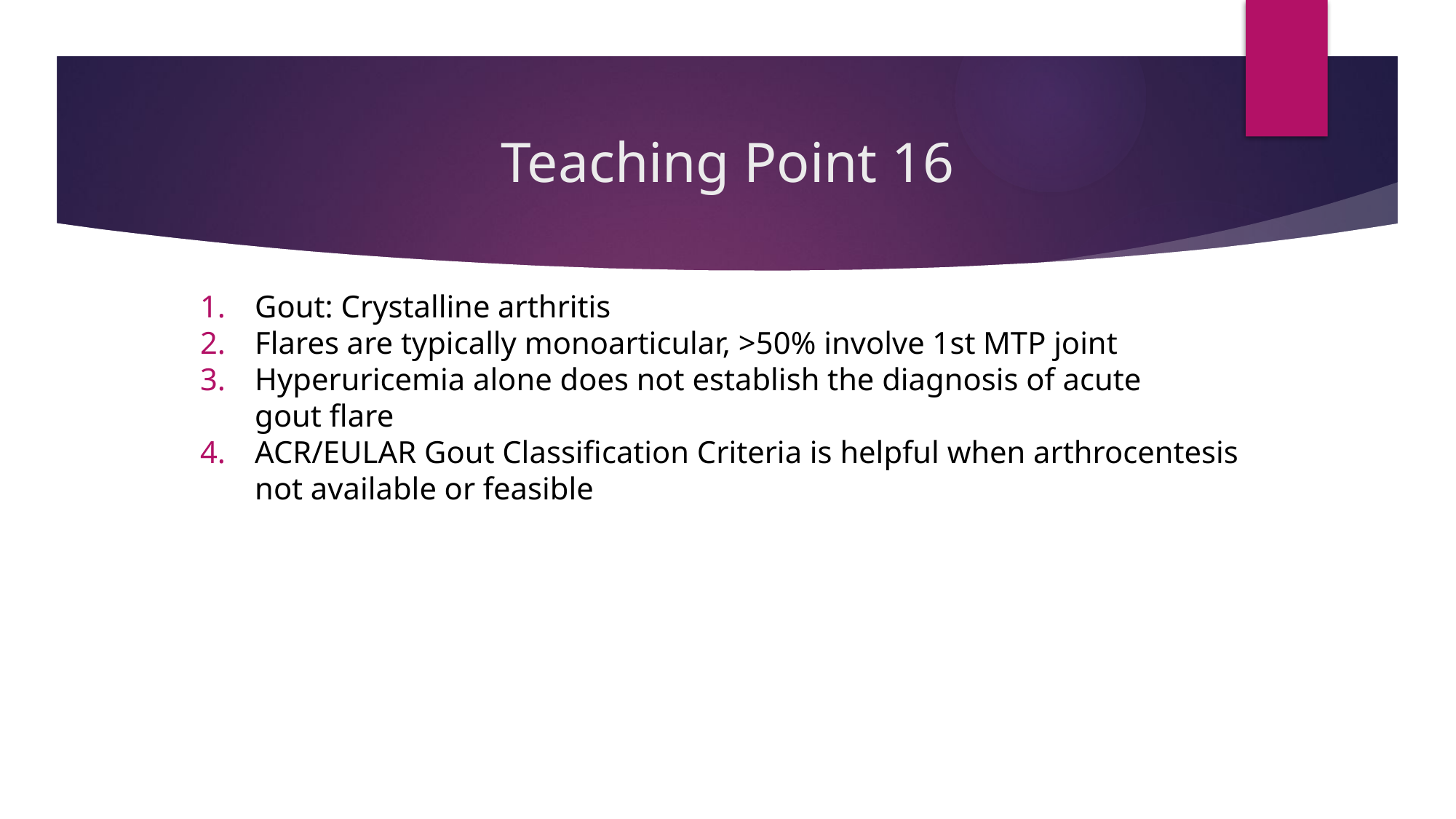

# Teaching Point 16
Gout: Crystalline arthritis
Flares are typically monoarticular, >50% involve 1st MTP joint
Hyperuricemia alone does not establish the diagnosis of acute gout flare
ACR/EULAR Gout Classification Criteria is helpful when arthrocentesis not available or feasible

## Slide 50
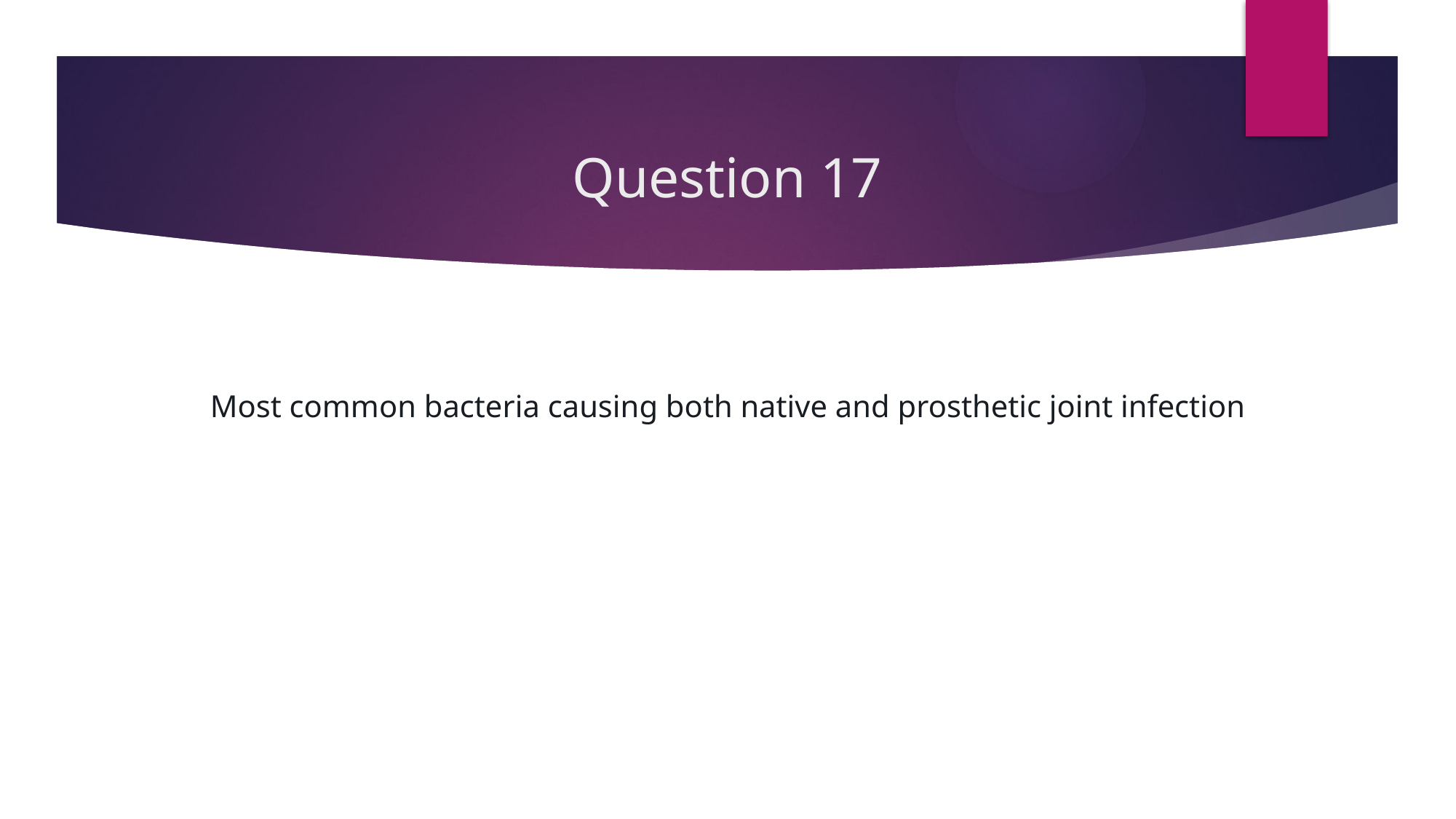

# Question 17
Most common bacteria causing both native and prosthetic joint infection

## Slide 51
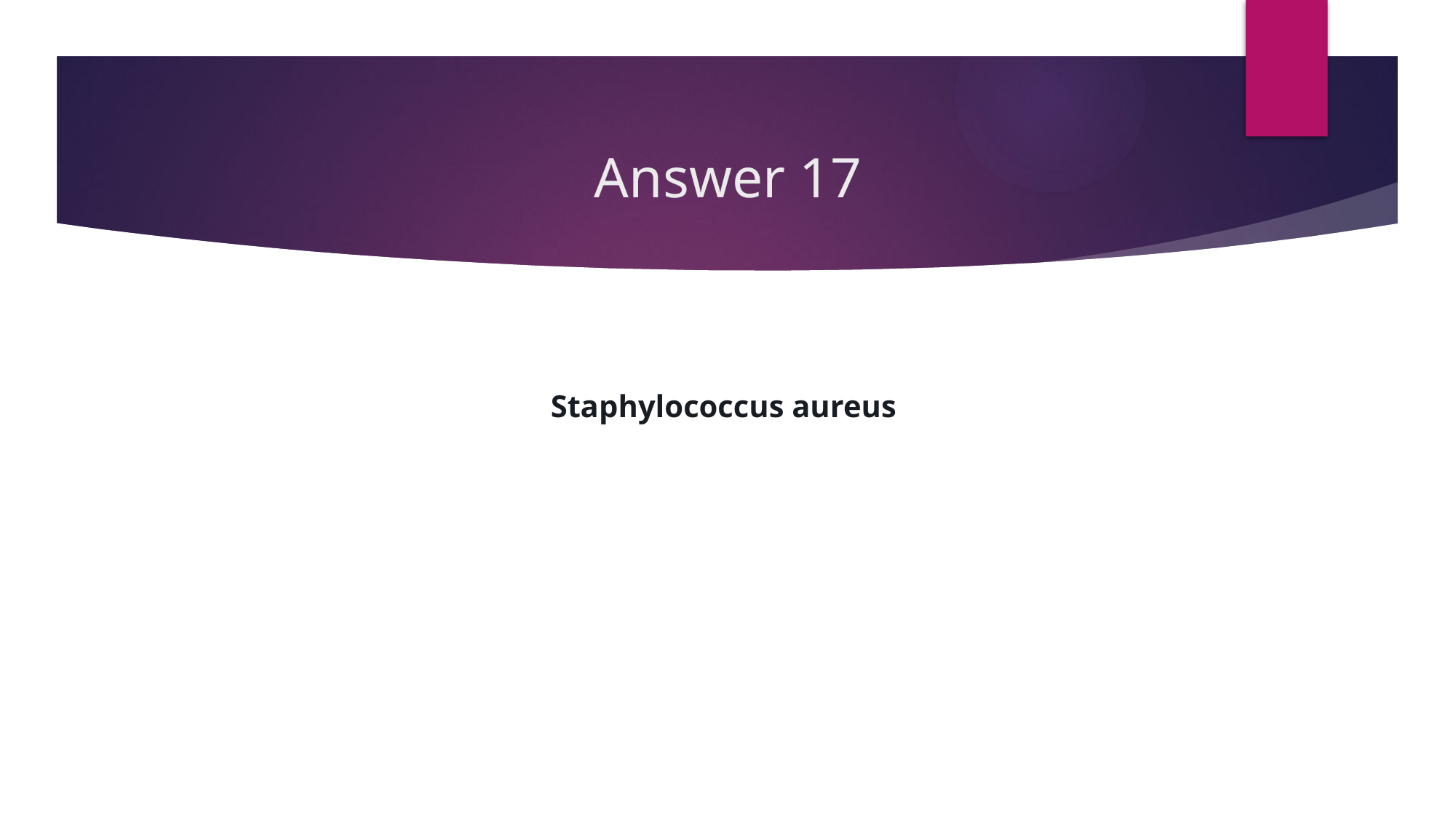

# Answer 17
Staphylococcus aureus

## Slide 52
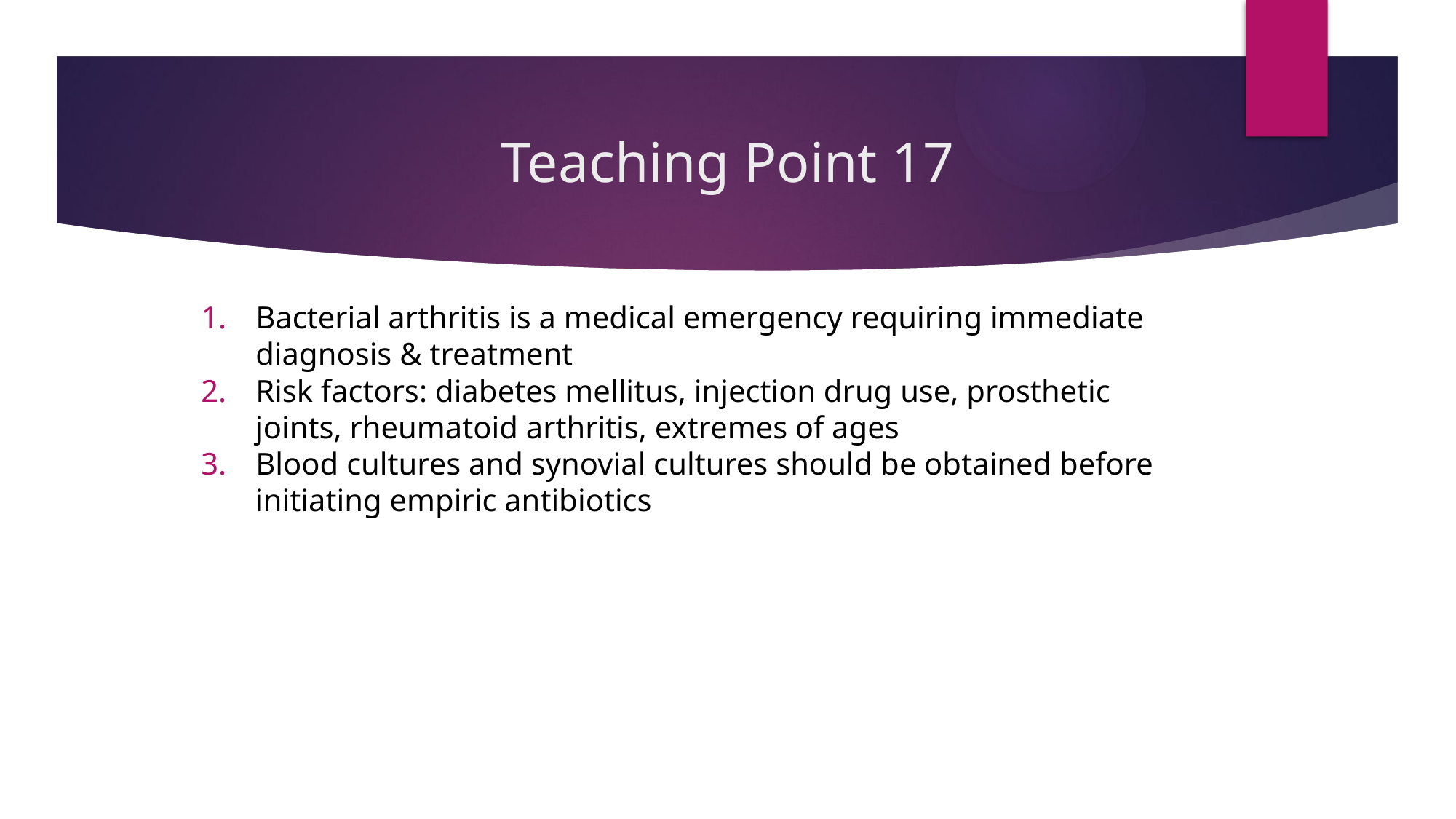

# Teaching Point 17
Bacterial arthritis is a medical emergency requiring immediate diagnosis & treatment
Risk factors: diabetes mellitus, injection drug use, prosthetic joints, rheumatoid arthritis, extremes of ages
Blood cultures and synovial cultures should be obtained before initiating empiric antibiotics

## Slide 53
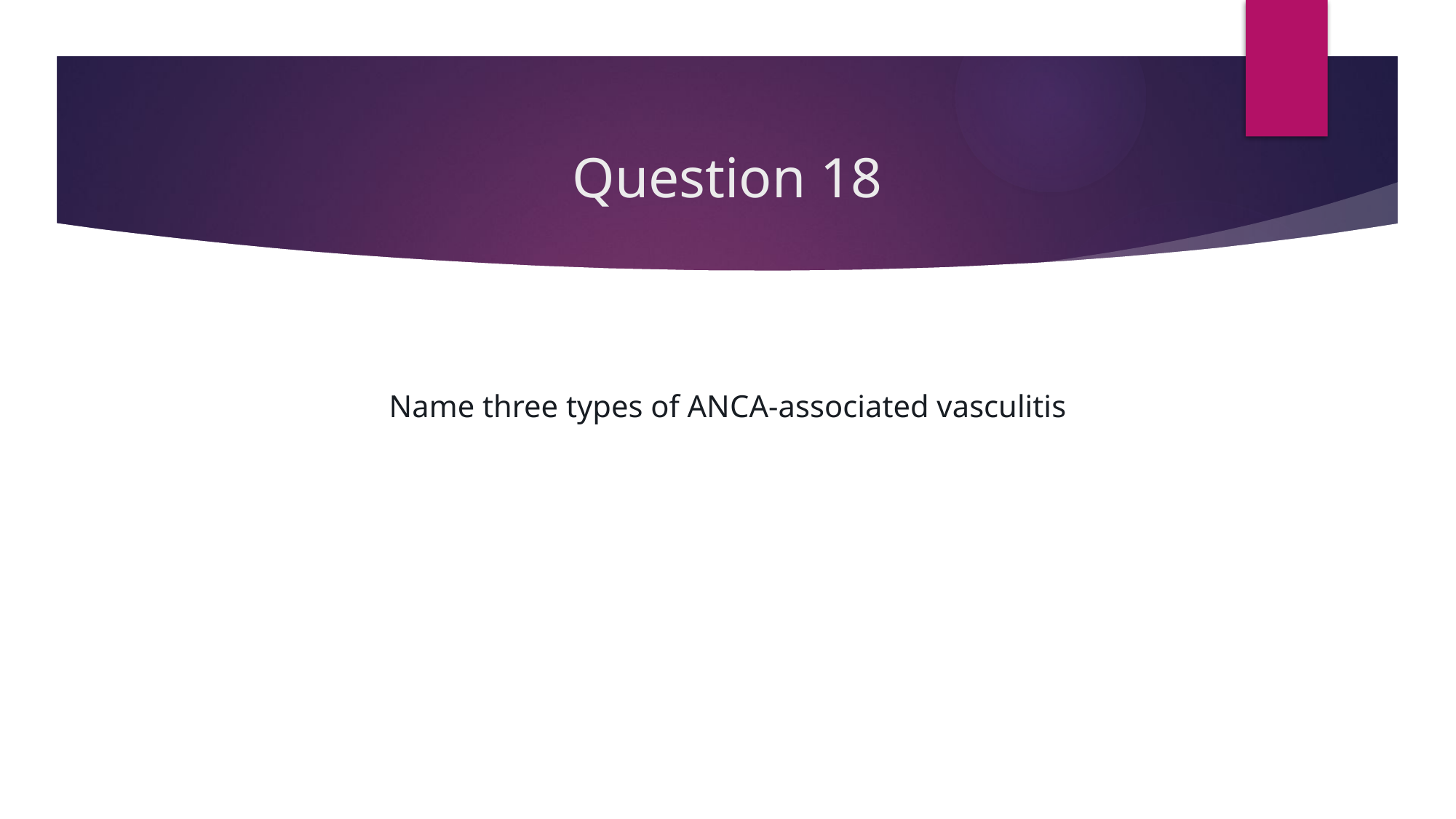

# Question 18
Name three types of ANCA-associated vasculitis

## Slide 54
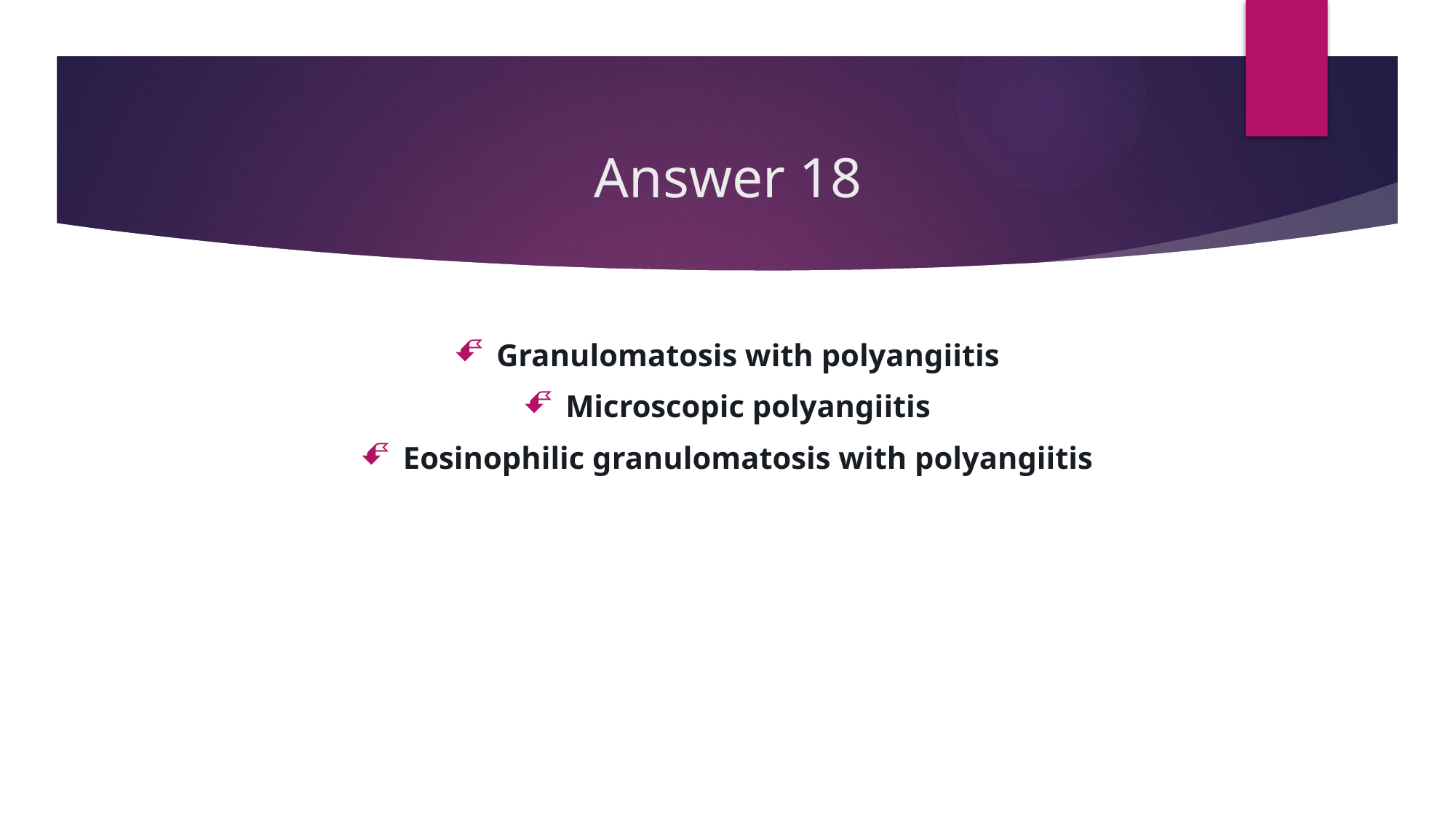

# Answer 18
Granulomatosis with polyangiitis
Microscopic polyangiitis
Eosinophilic granulomatosis with polyangiitis

## Slide 55
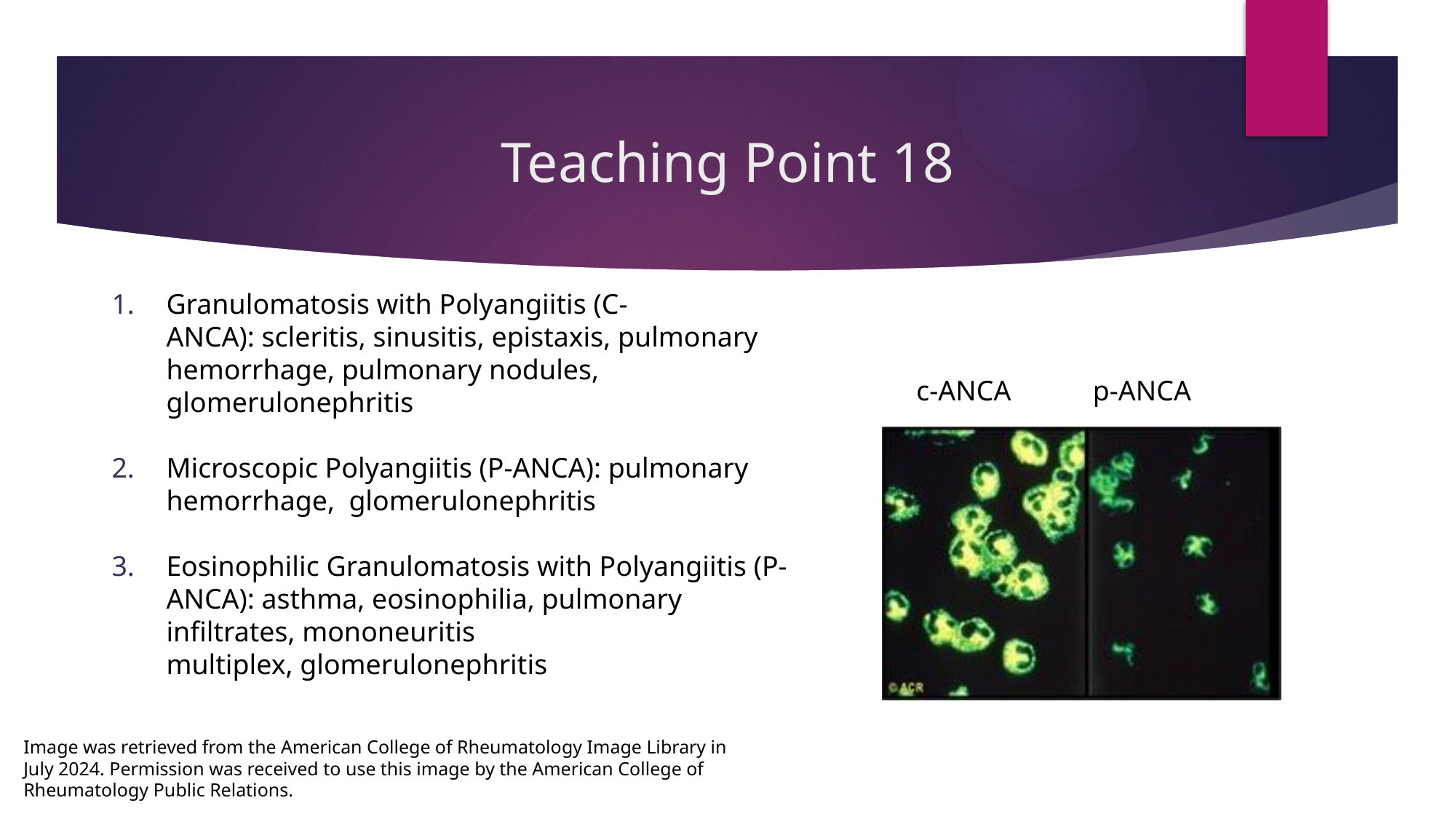

# Teaching Point 18
Granulomatosis with Polyangiitis (C-ANCA): scleritis, sinusitis, epistaxis, pulmonary hemorrhage, pulmonary nodules, glomerulonephritis
Microscopic Polyangiitis (P-ANCA): pulmonary hemorrhage,  glomerulonephritis
Eosinophilic Granulomatosis with Polyangiitis (P-ANCA): asthma, eosinophilia, pulmonary infiltrates, mononeuritis multiplex, glomerulonephritis
c-ANCA   p-ANCA
Image was retrieved from the American College of Rheumatology Image Library in July 2024. Permission was received to use this image by the American College of Rheumatology Public Relations.

## Slide 56
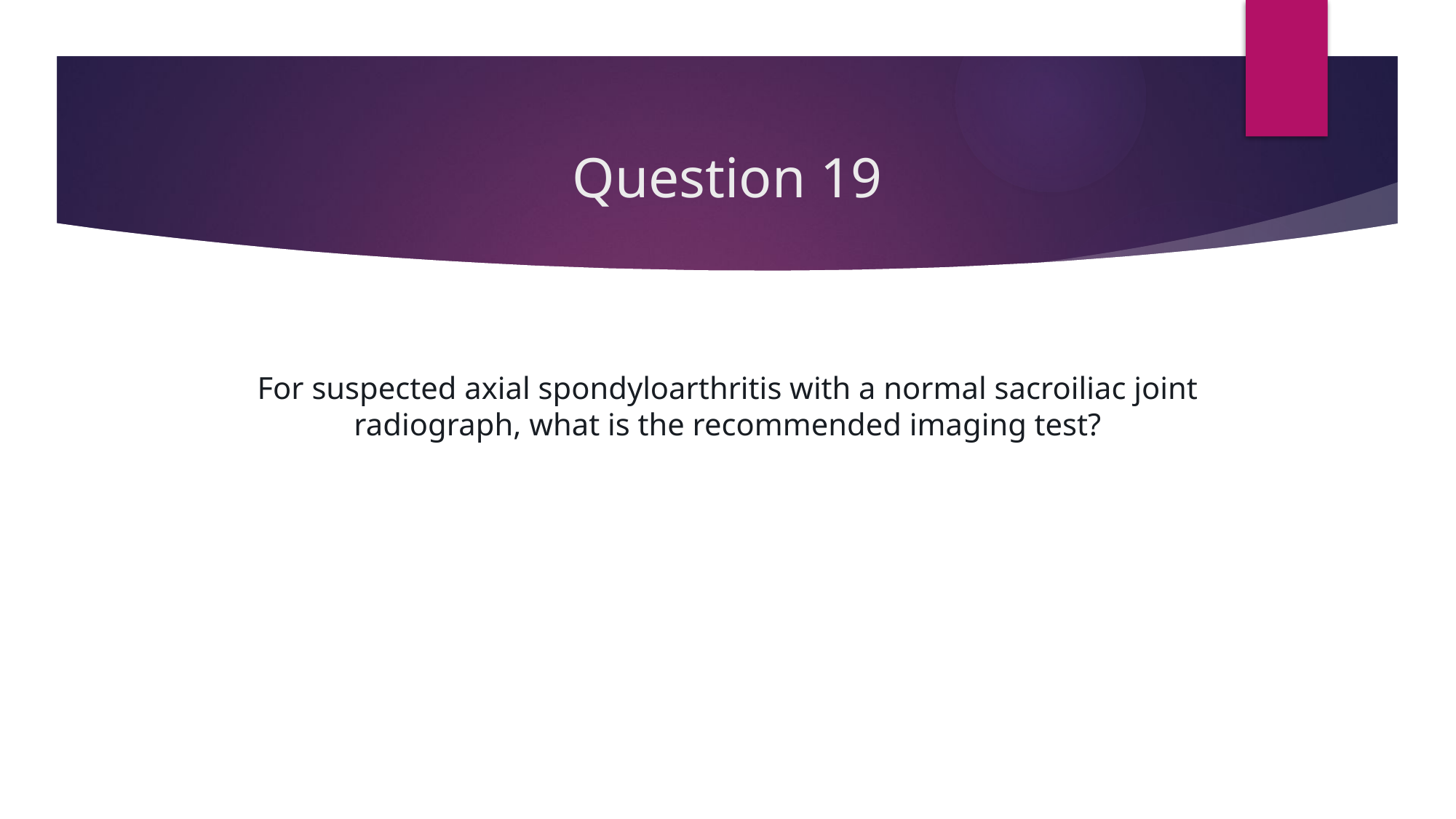

# Question 19
For suspected axial spondyloarthritis with a normal sacroiliac joint radiograph, what is the recommended imaging test?

## Slide 57
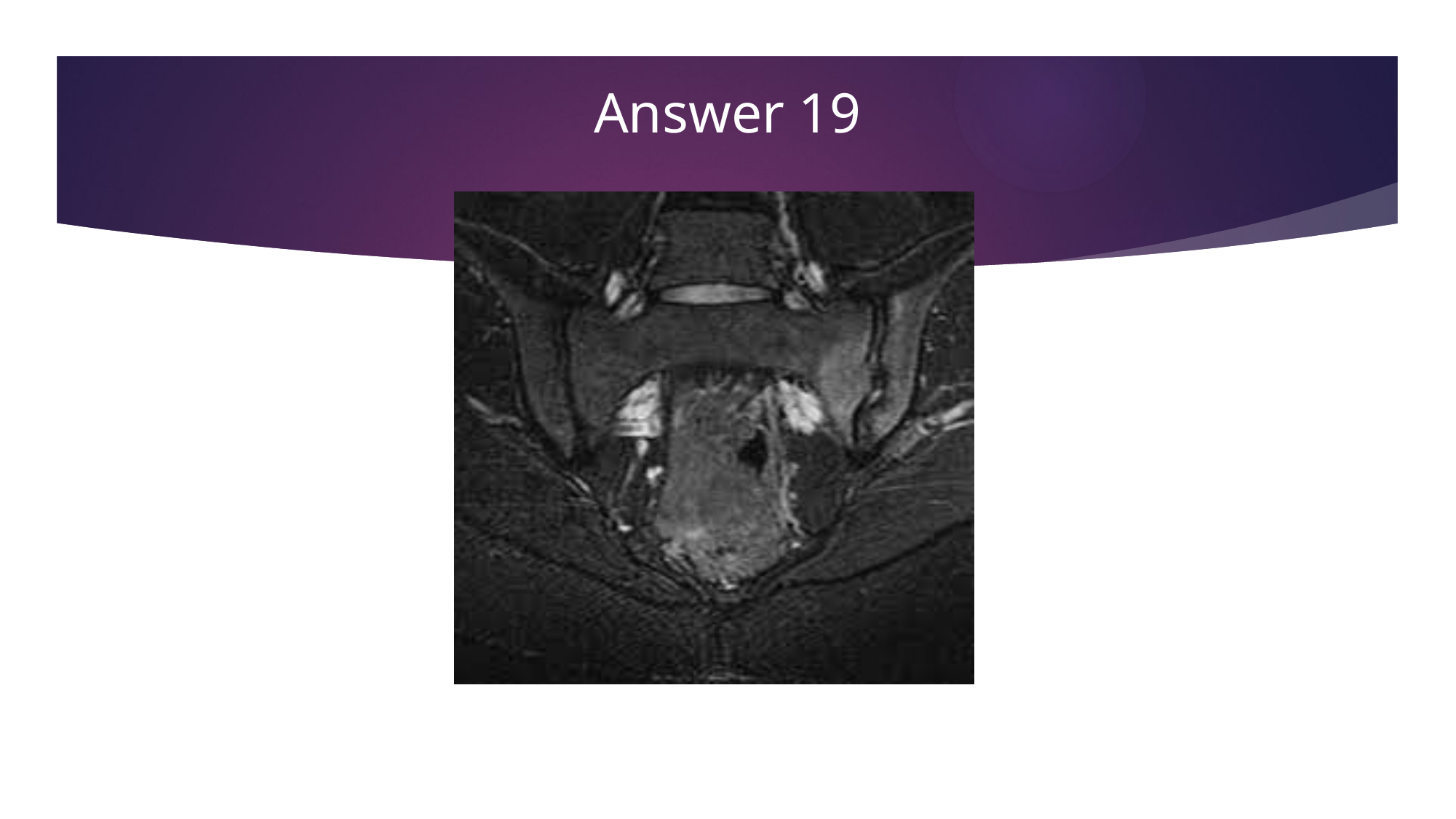

# Answer 19
Marrow edema and post contrast enhancement across the left sacroiliac joint reflecting sacroiliitis.
MRI of sacroiliac joint
Image by Roberto Schubert, retrieved from https://radiopaedia.org/cases/sacroiliitis-9?lang=us on July 16 2024. Creative Commons License associated: https://radiopaedia.org/licence?lang=us

## Slide 58
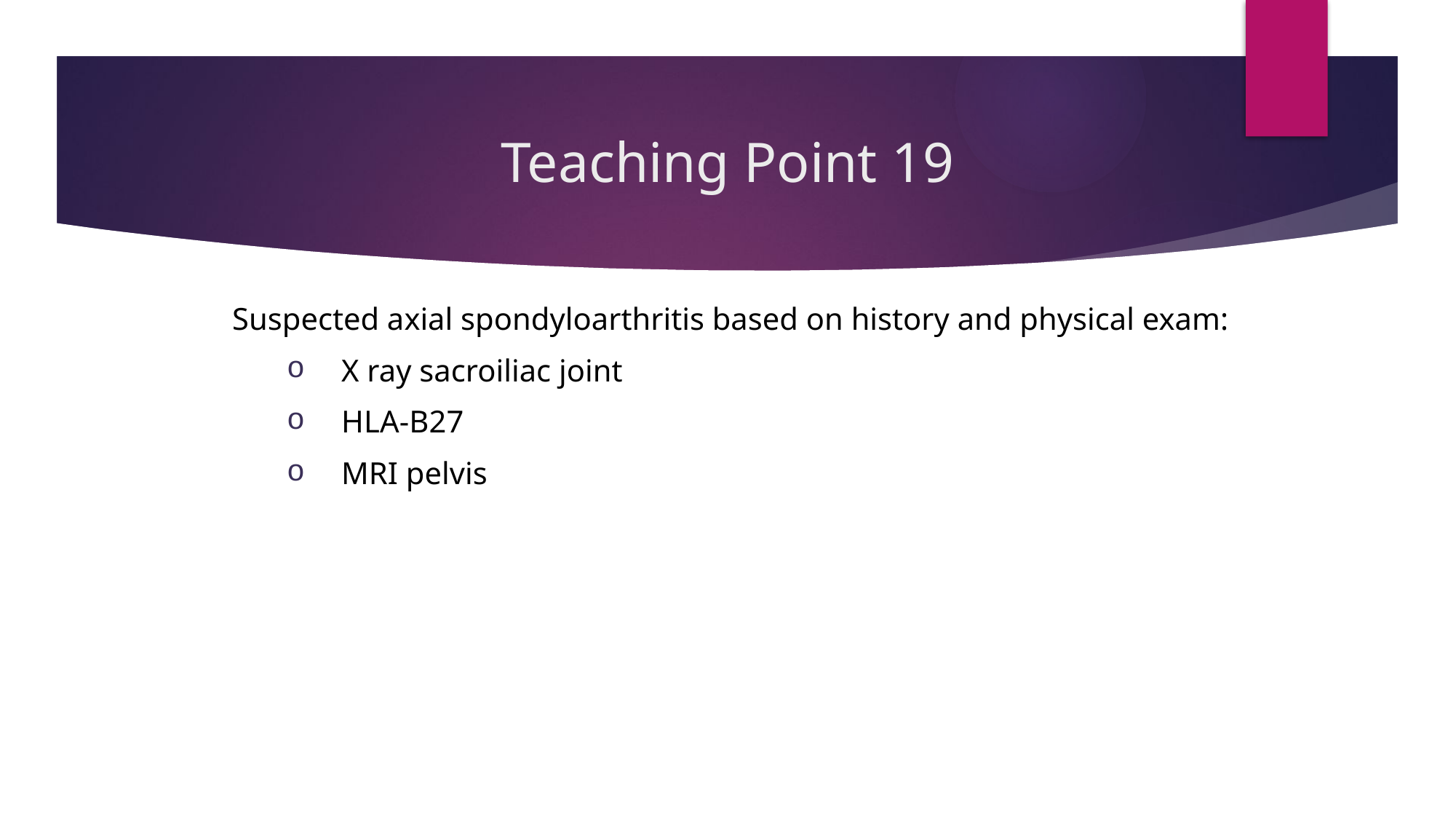

# Teaching Point 19
Suspected axial spondyloarthritis based on history and physical exam:
X ray sacroiliac joint
HLA-B27
MRI pelvis

## Slide 59
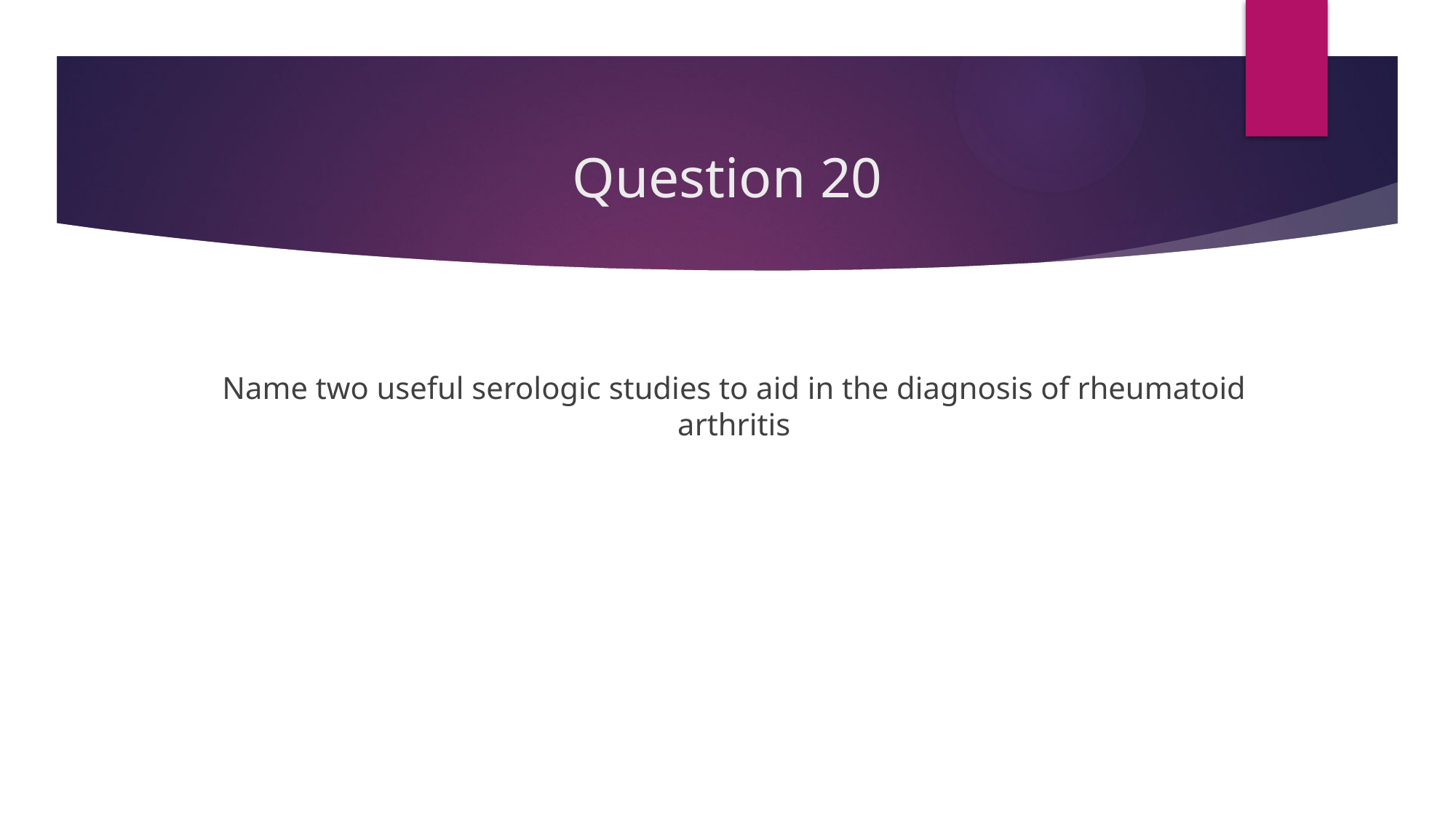

# Question 20
Name two useful serologic studies to aid in the diagnosis of rheumatoid arthritis

## Slide 60
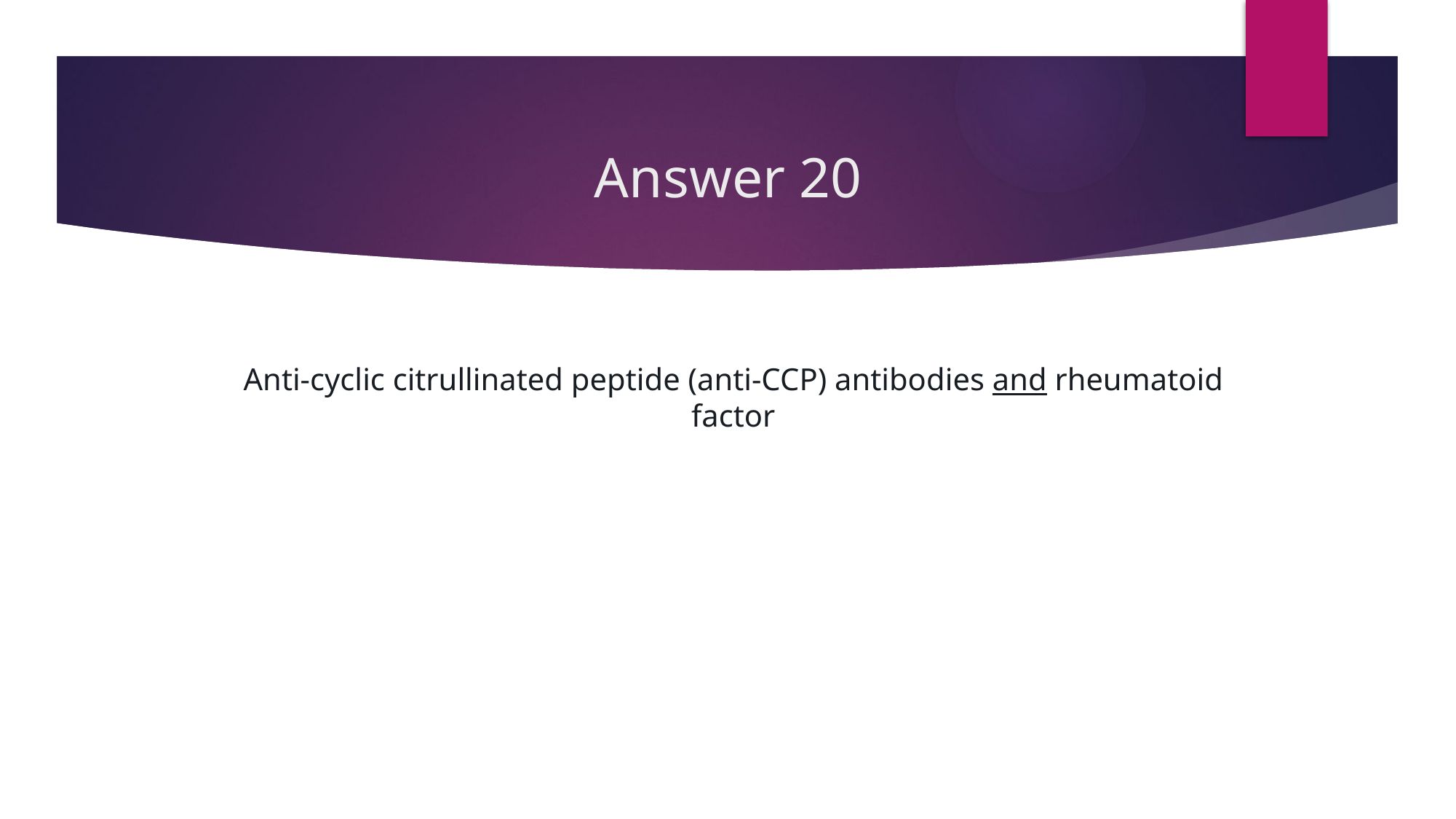

# Answer 20
Anti-cyclic citrullinated peptide (anti-CCP) antibodies and rheumatoid factor

## Slide 61
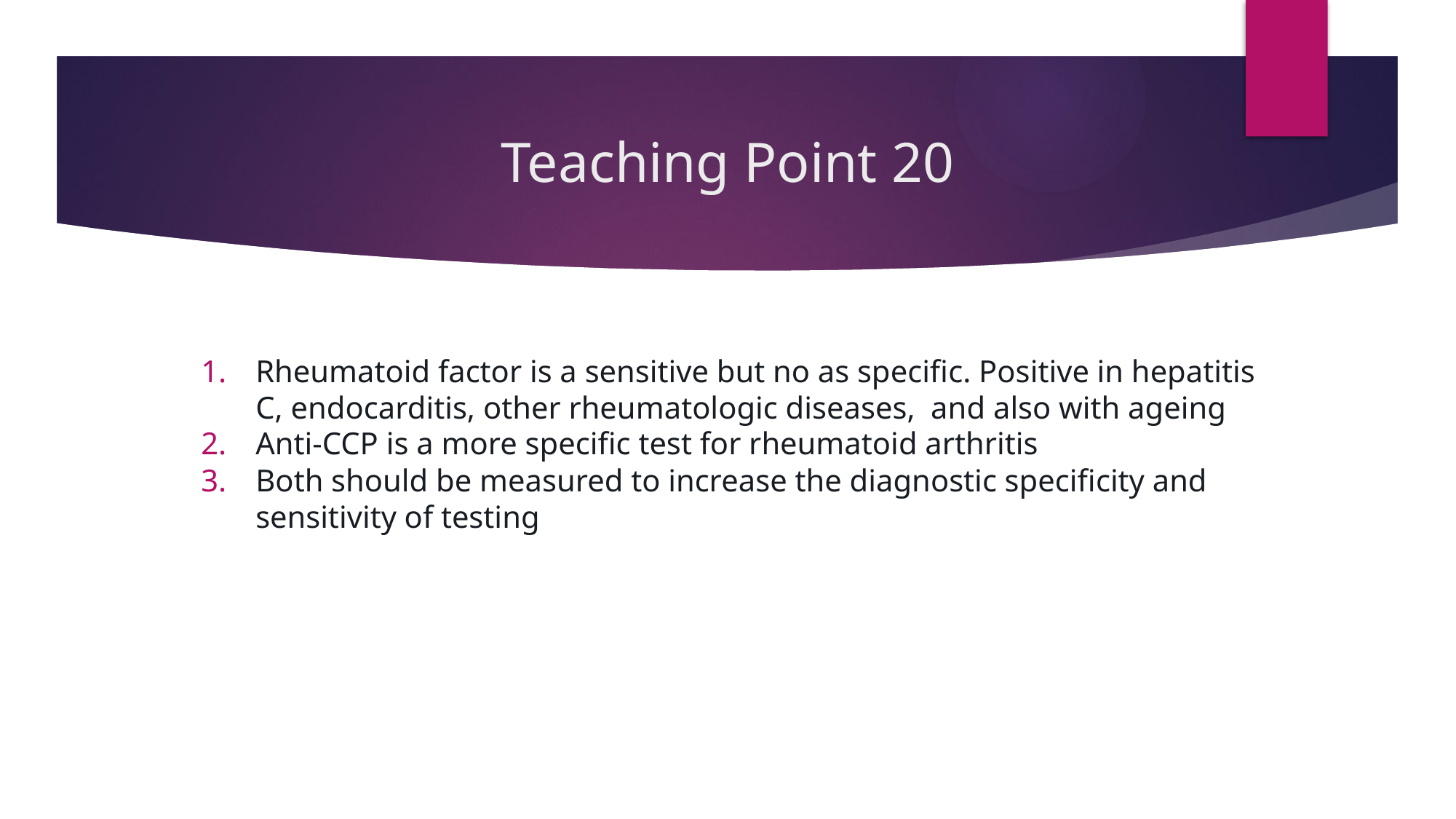

# Teaching Point 20
​
Rheumatoid factor is a sensitive but no as specific. Positive in hepatitis C, endocarditis, other rheumatologic diseases,  and also with ageing
Anti-CCP is a more specific test for rheumatoid arthritis
Both should be measured to increase the diagnostic specificity and sensitivity of testing

## Slide 62
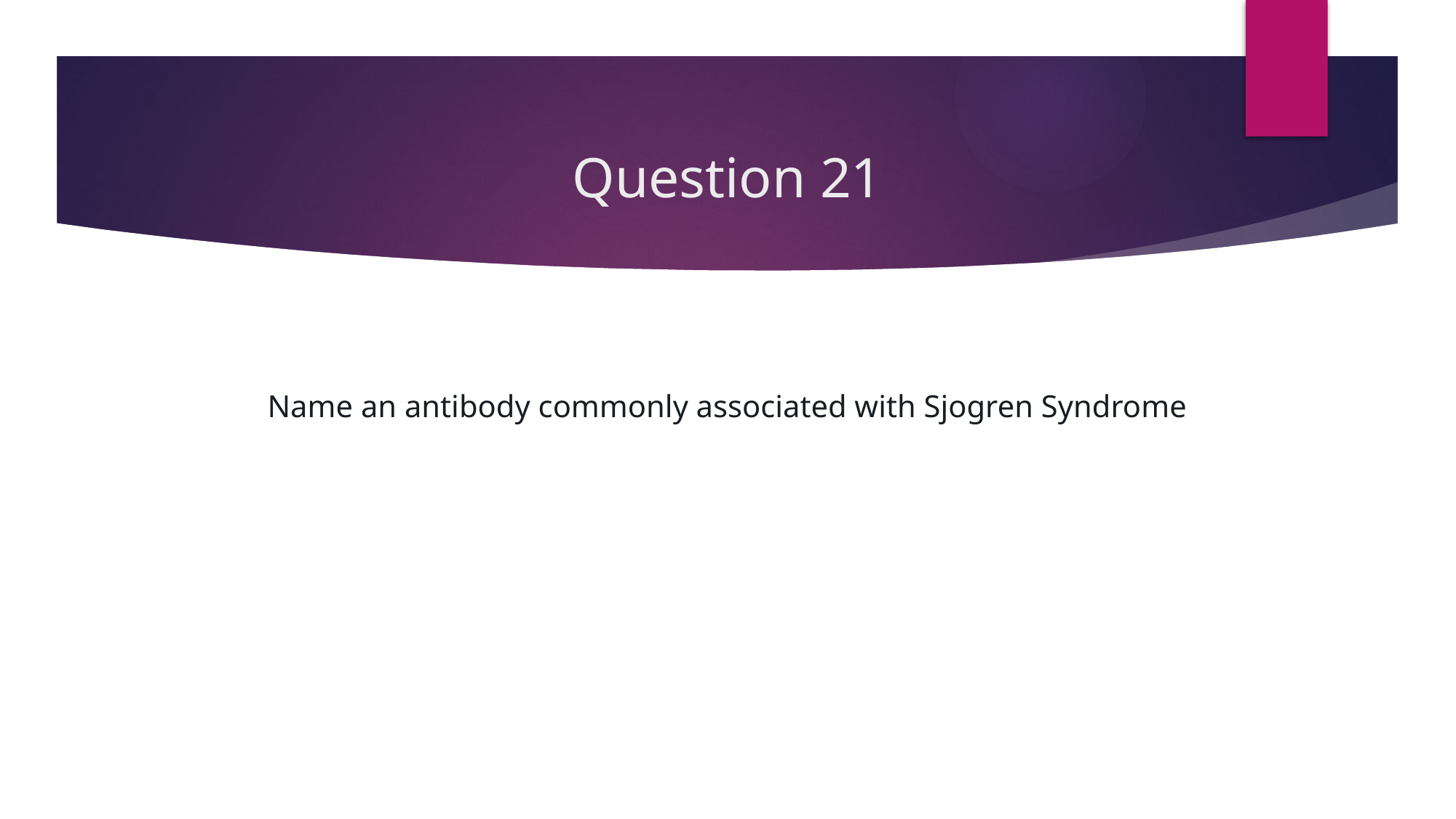

# Question 21
Name an antibody commonly associated with Sjogren Syndrome

## Slide 63
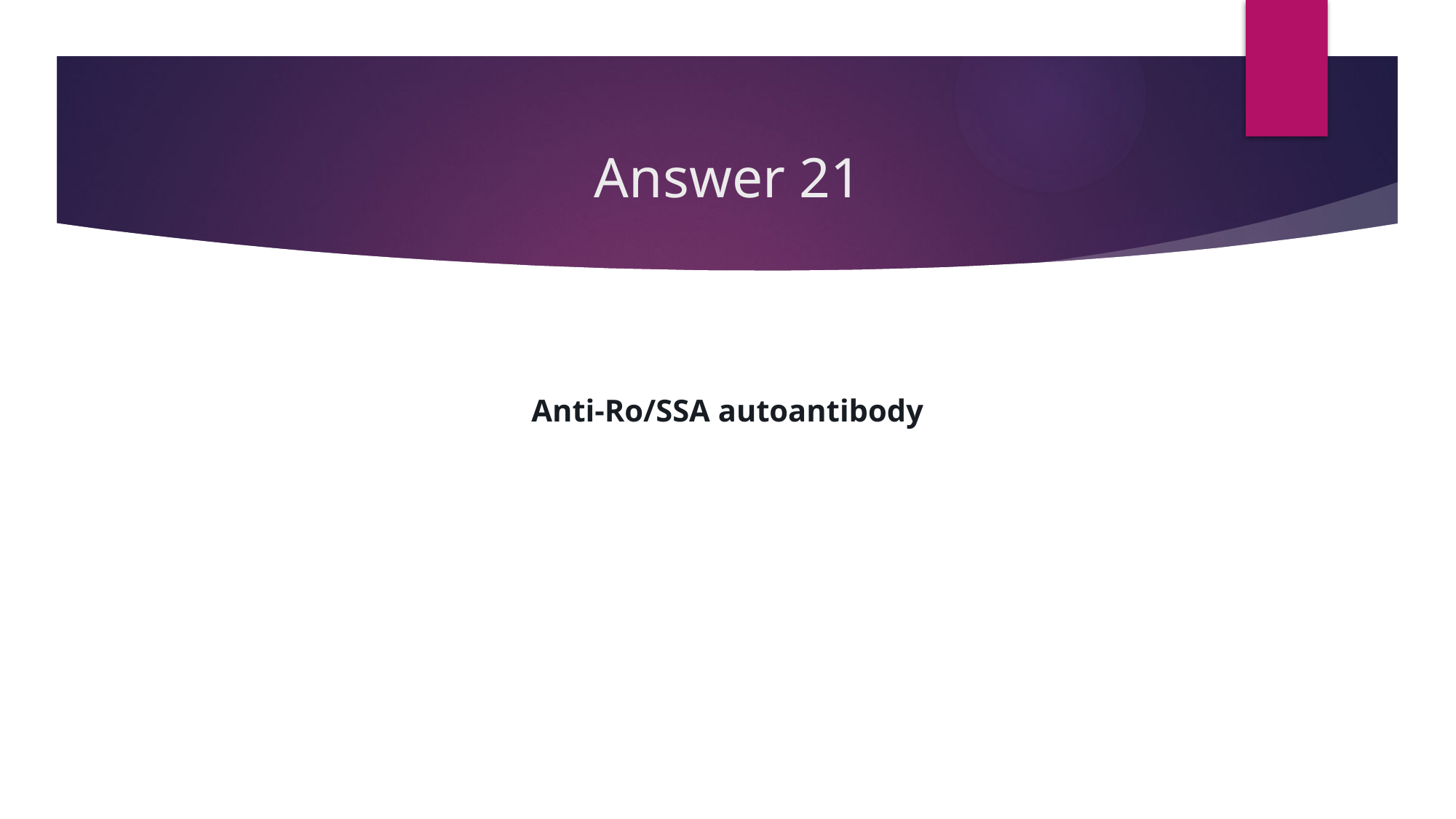

# Answer 21
Anti-Ro/SSA autoantibody

## Slide 64
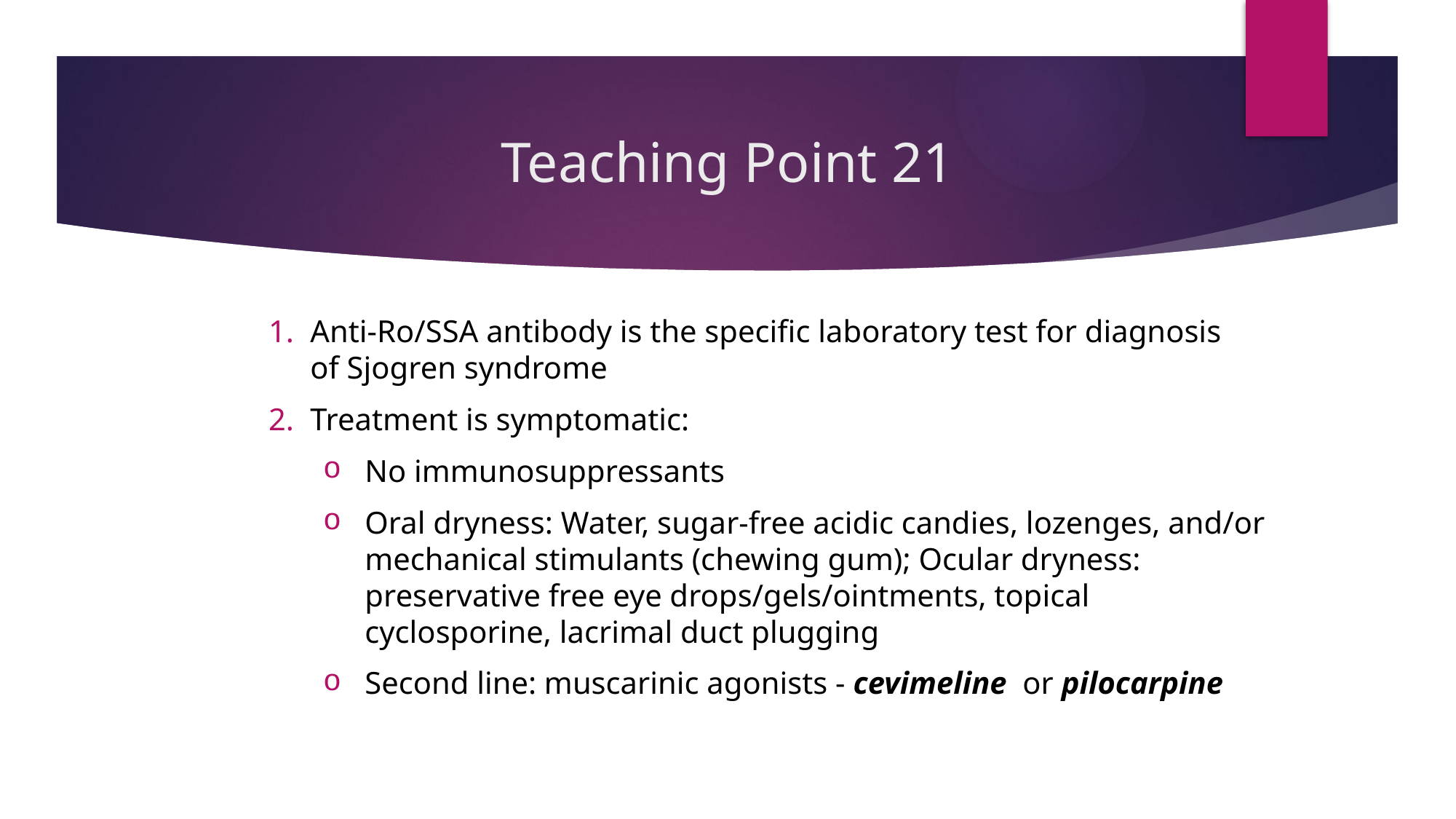

# Teaching Point 21
​
Anti-Ro/SSA antibody is the specific laboratory test for diagnosis of Sjogren syndrome
Treatment is symptomatic:
No immunosuppressants
Oral dryness: Water, sugar-free acidic candies, lozenges, and/or mechanical stimulants (chewing gum); Ocular dryness: preservative free eye drops/gels/ointments, topical cyclosporine, lacrimal duct plugging
Second line: muscarinic agonists - cevimeline  or pilocarpine

## Slide 65
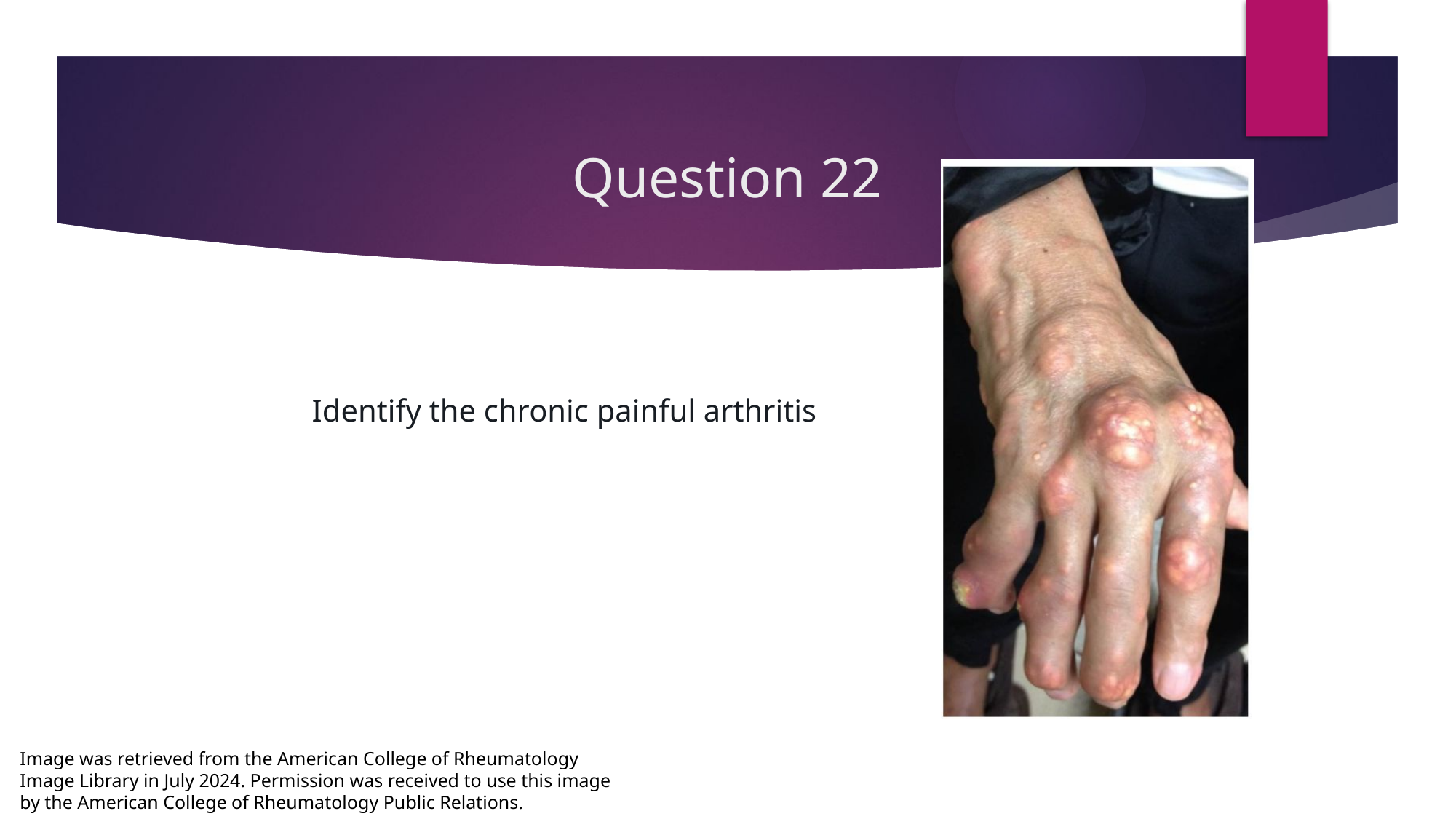

# Question 22
Identify the chronic painful arthritis
Image was retrieved from the American College of Rheumatology Image Library in July 2024. Permission was received to use this image by the American College of Rheumatology Public Relations.

## Slide 66
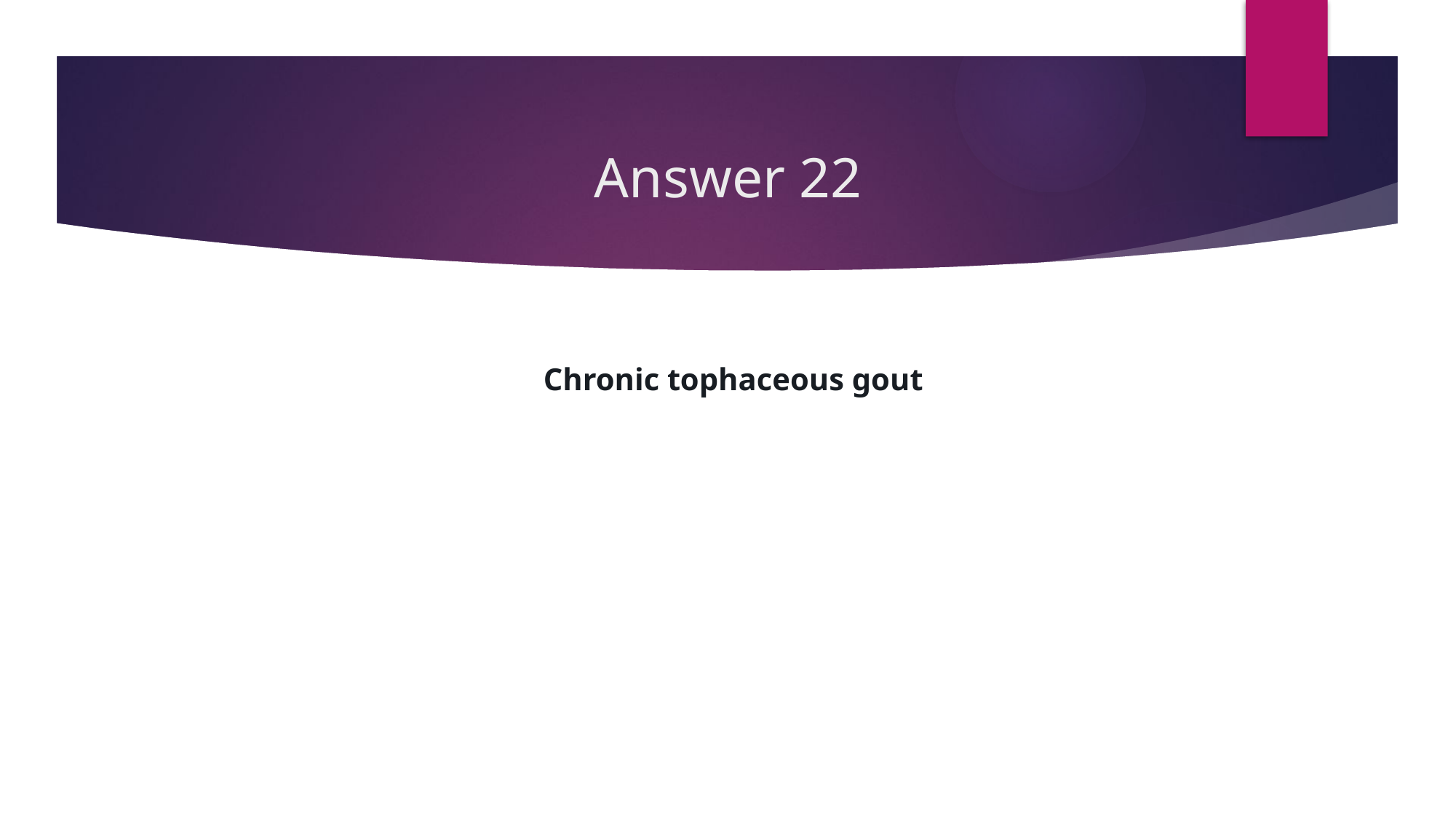

# Answer 22
Chronic tophaceous gout

## Slide 67
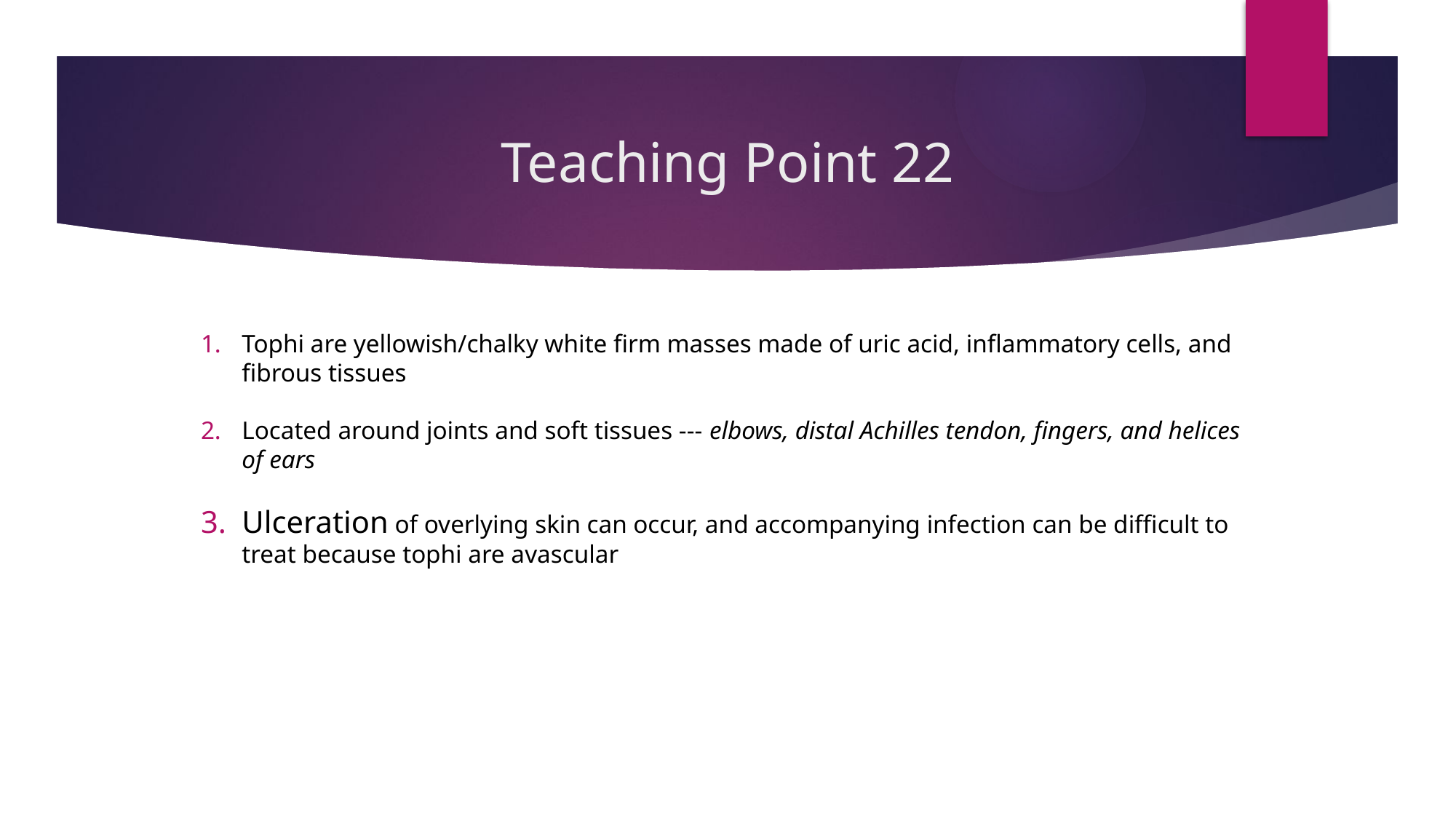

# Teaching Point 22
​
Tophi are yellowish/chalky white firm masses made of uric acid, inflammatory cells, and fibrous tissues
Located around joints and soft tissues --- elbows, distal Achilles tendon, fingers, and helices of ears
Ulceration of overlying skin can occur, and accompanying infection can be difficult to treat because tophi are avascular

## Slide 68
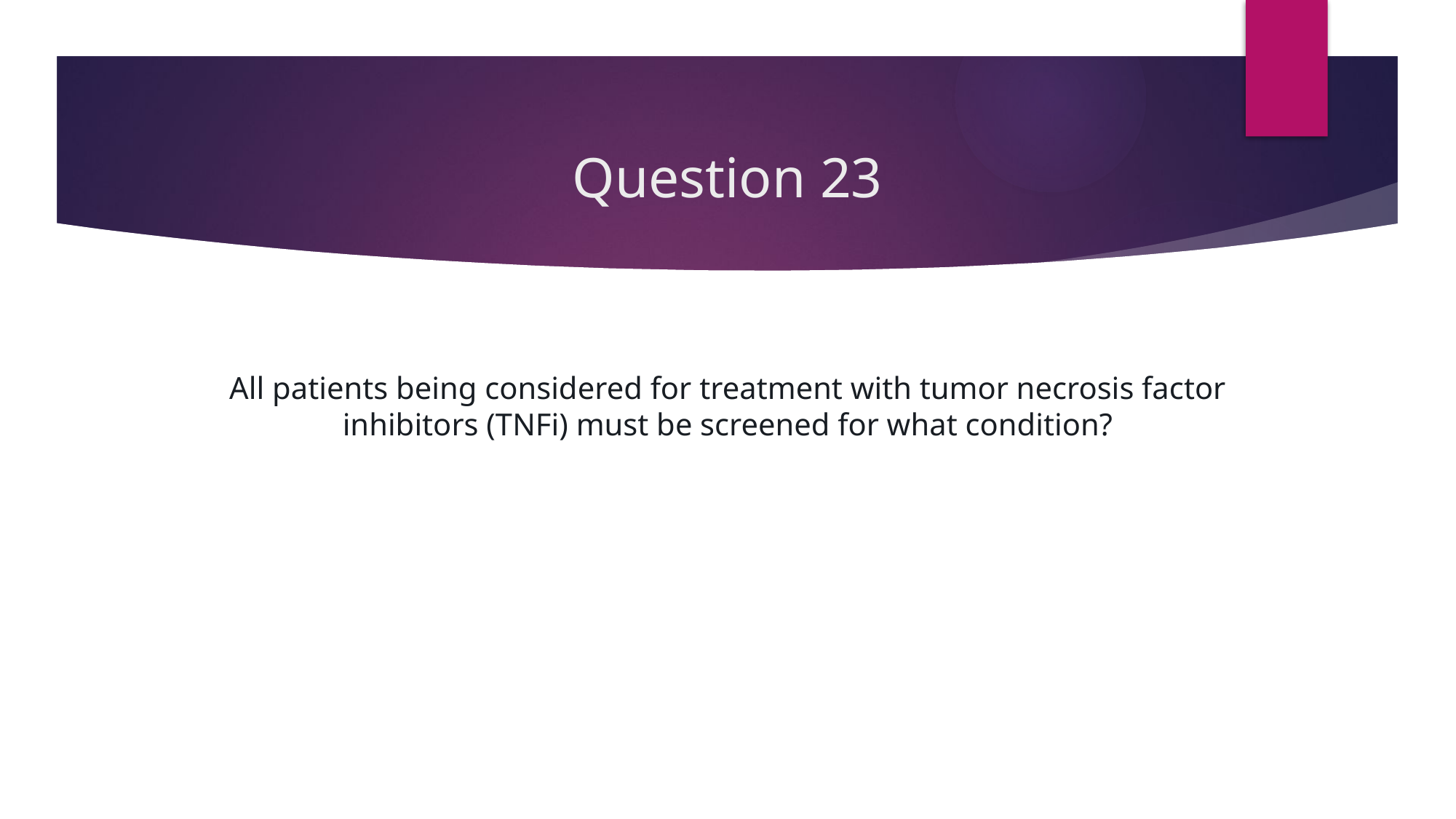

# Question 23
All patients being considered for treatment with tumor necrosis factor inhibitors (TNFi) must be screened for what condition?

## Slide 69
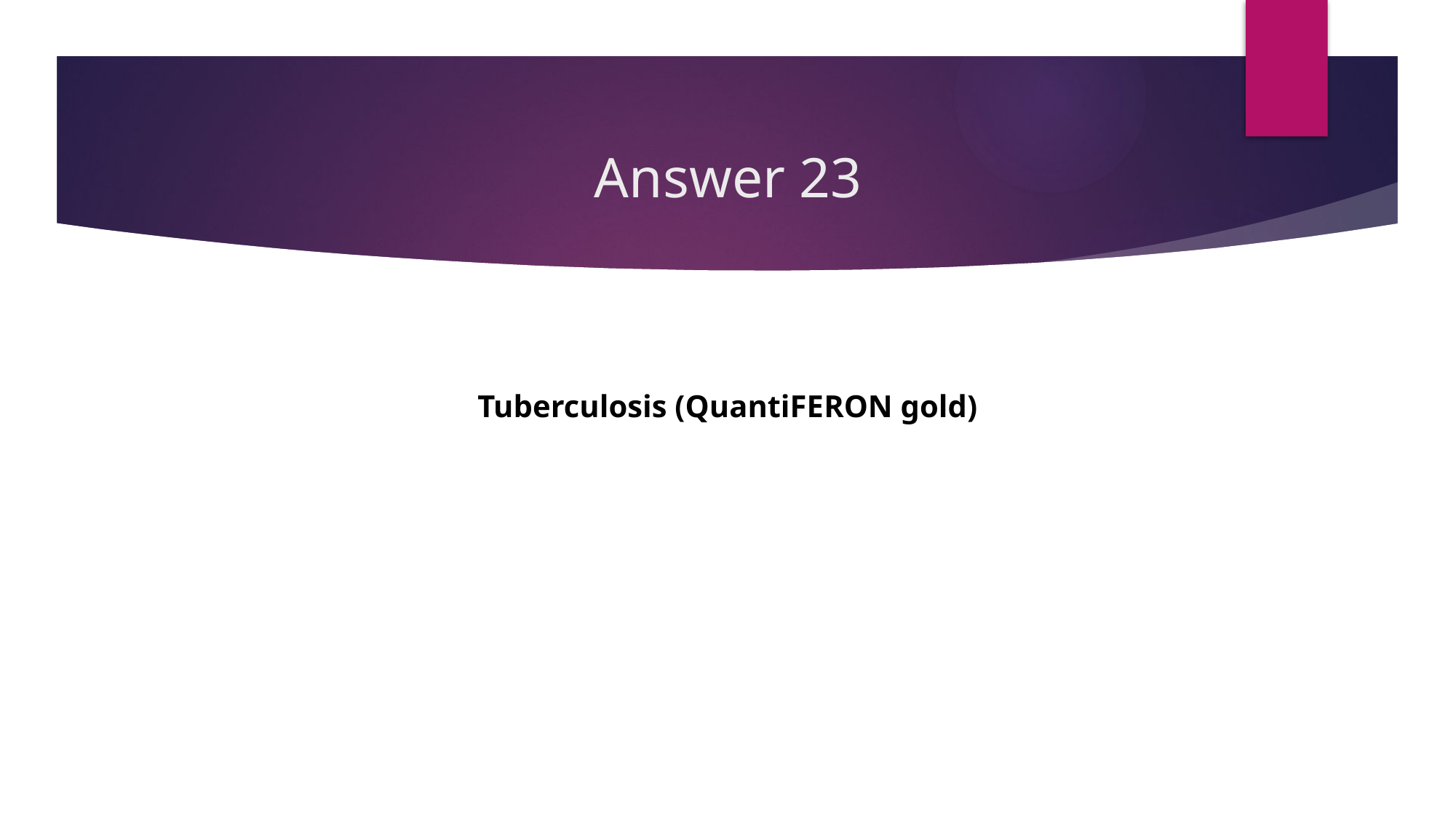

# Answer 23
Tuberculosis (QuantiFERON gold)

## Slide 70
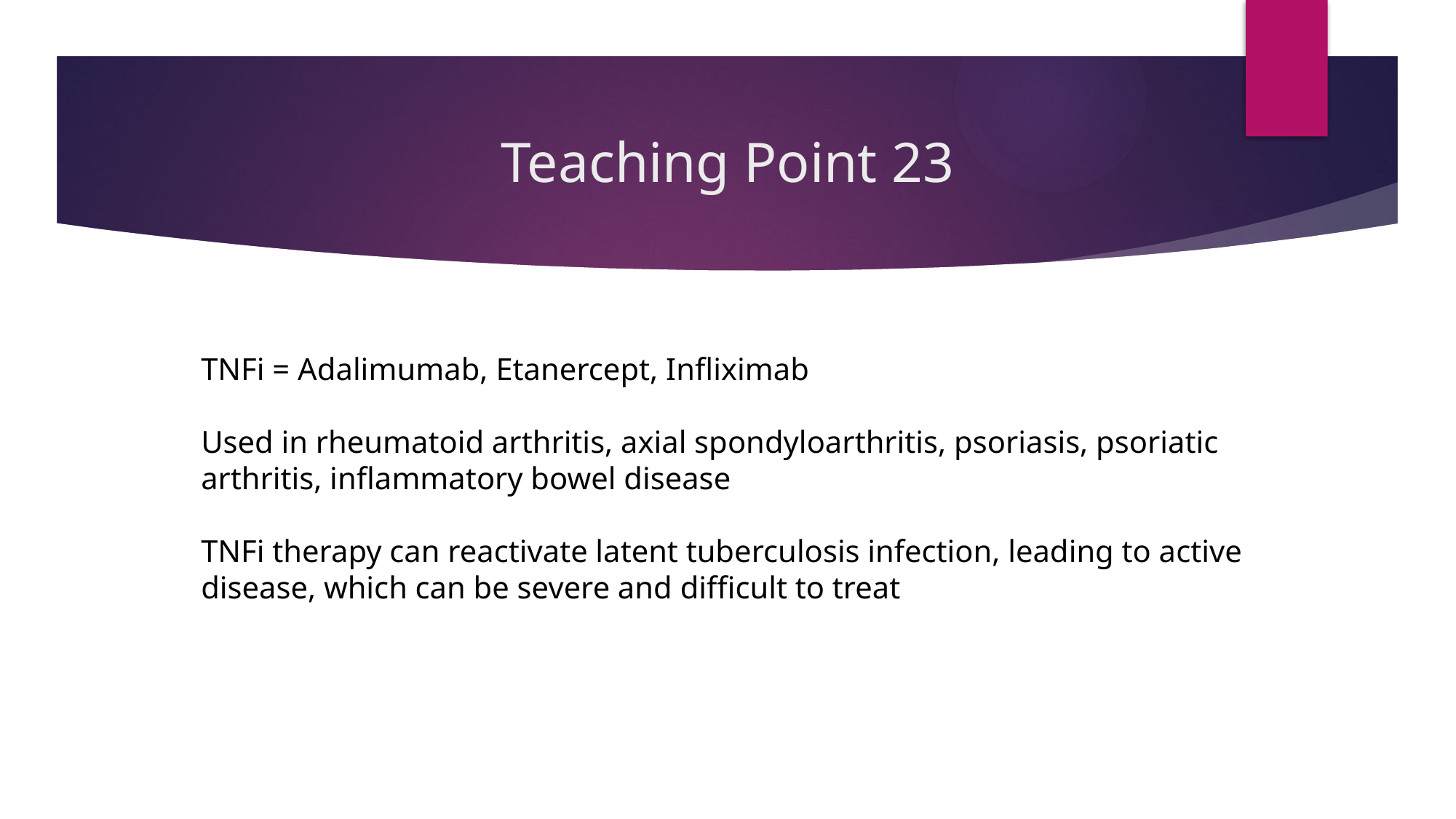

# Teaching Point 23
​
TNFi = Adalimumab, Etanercept, Infliximab
Used in rheumatoid arthritis, axial spondyloarthritis, psoriasis, psoriatic arthritis, inflammatory bowel disease
TNFi therapy can reactivate latent tuberculosis infection, leading to active disease, which can be severe and difficult to treat

## Slide 71
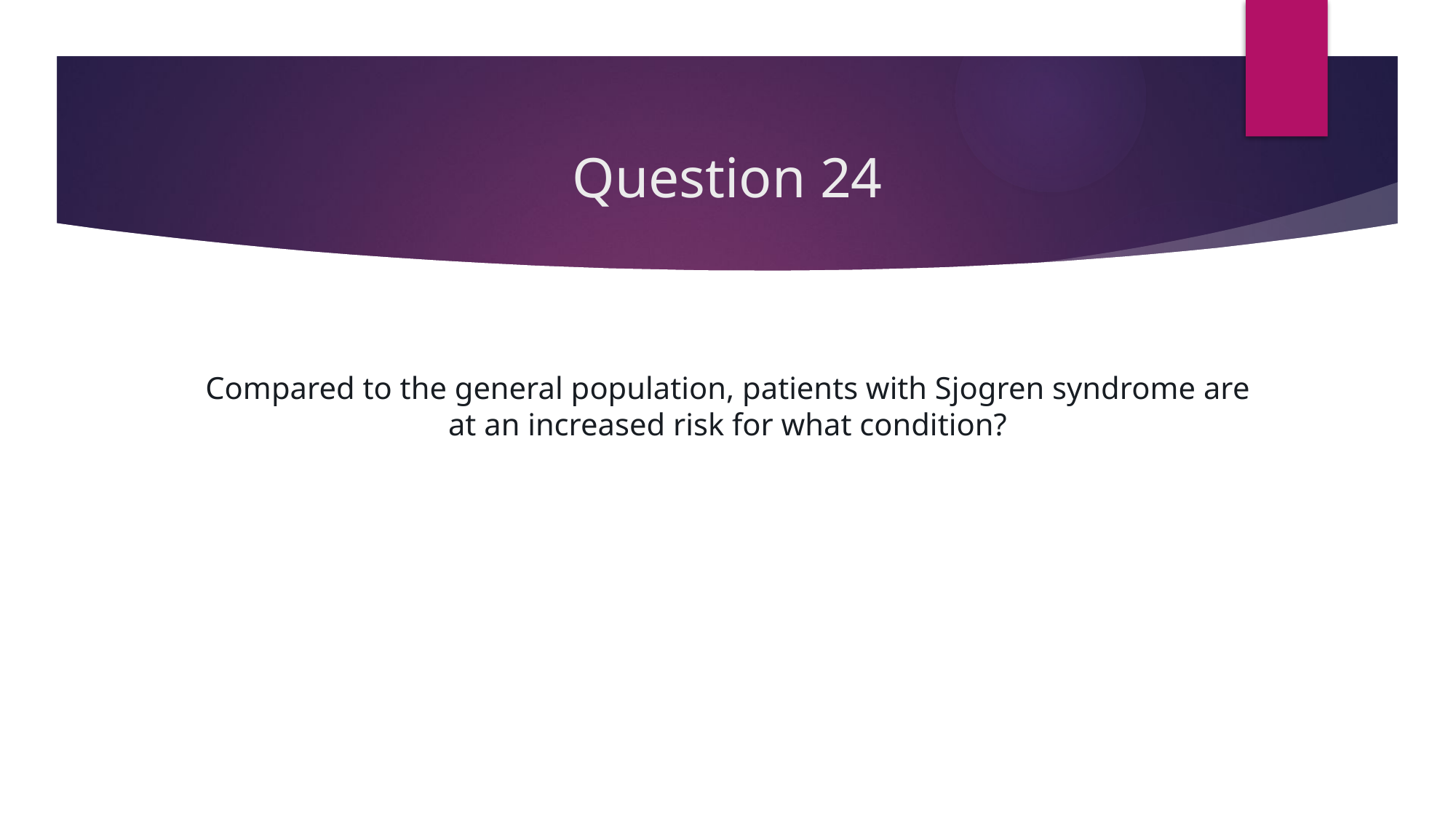

# Question 24
Compared to the general population, patients with Sjogren syndrome are at an increased risk for what condition?

## Slide 72
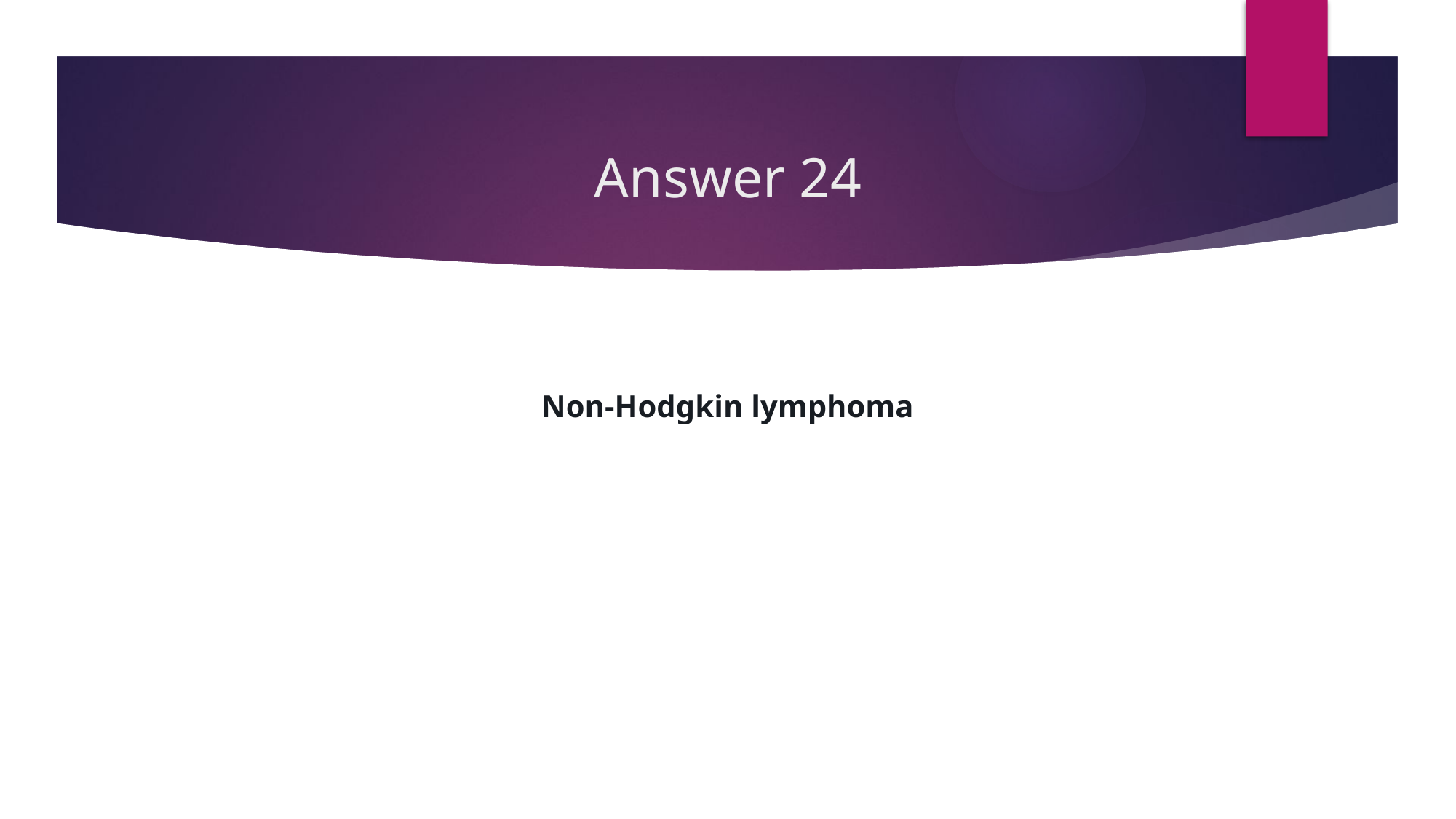

# Answer 24
Non-Hodgkin lymphoma

## Slide 73
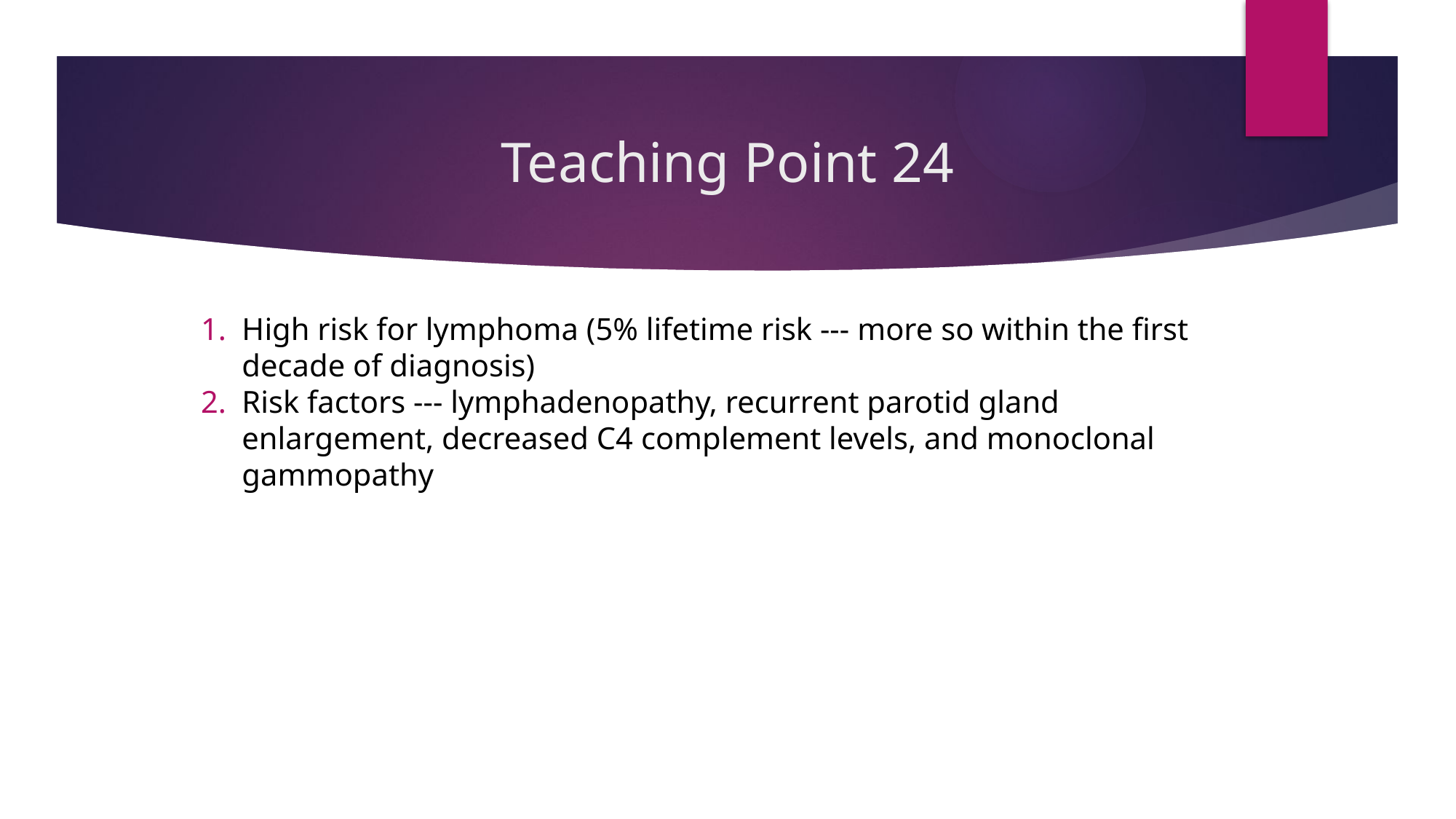

# Teaching Point 24
​
High risk for lymphoma (5% lifetime risk --- more so within the first decade of diagnosis)
Risk factors --- lymphadenopathy, recurrent parotid gland enlargement, decreased C4 complement levels, and monoclonal gammopathy

## Slide 74
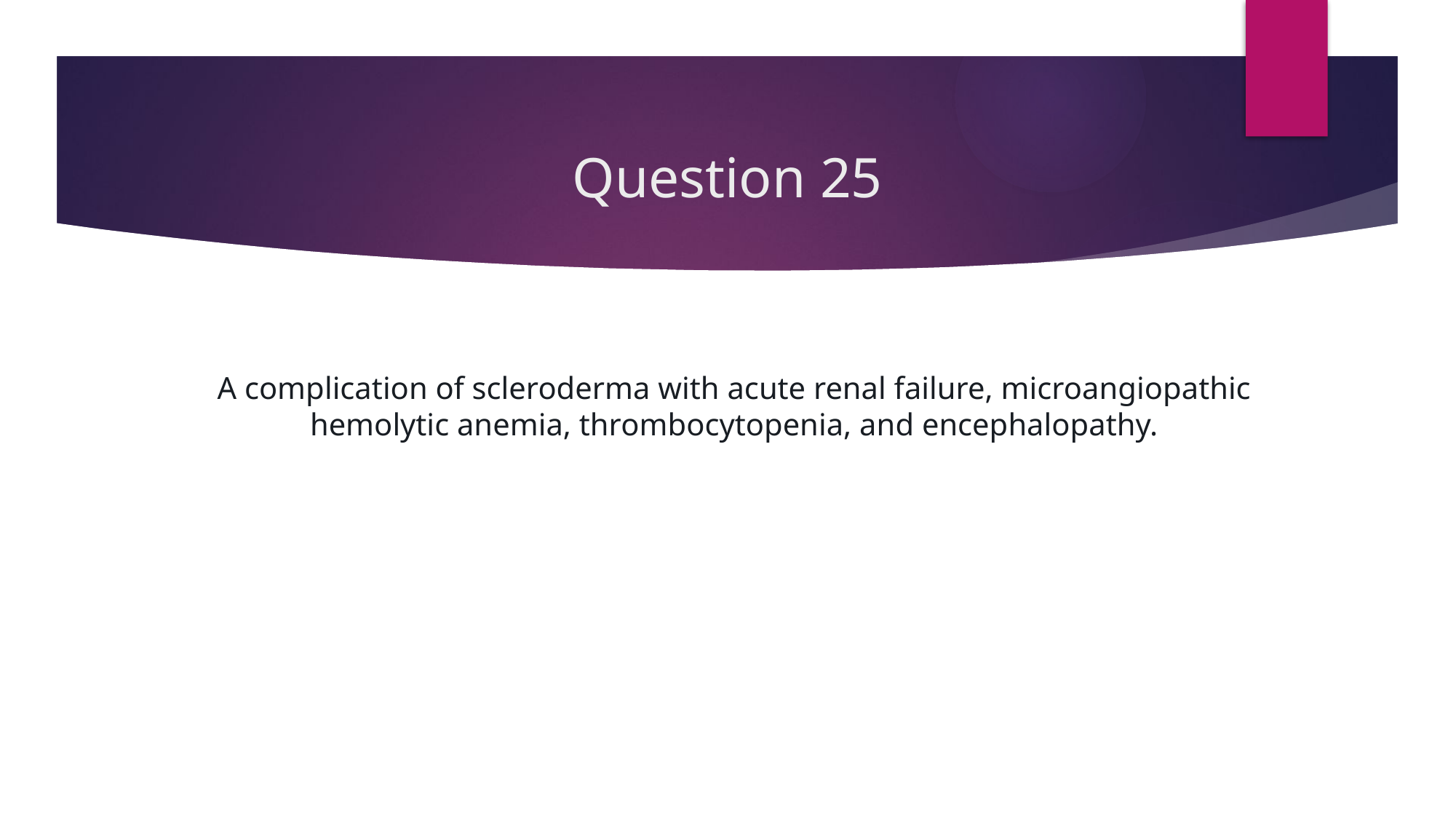

# Question 25
A complication of scleroderma with acute renal failure, microangiopathic hemolytic anemia, thrombocytopenia, and encephalopathy.

## Slide 75
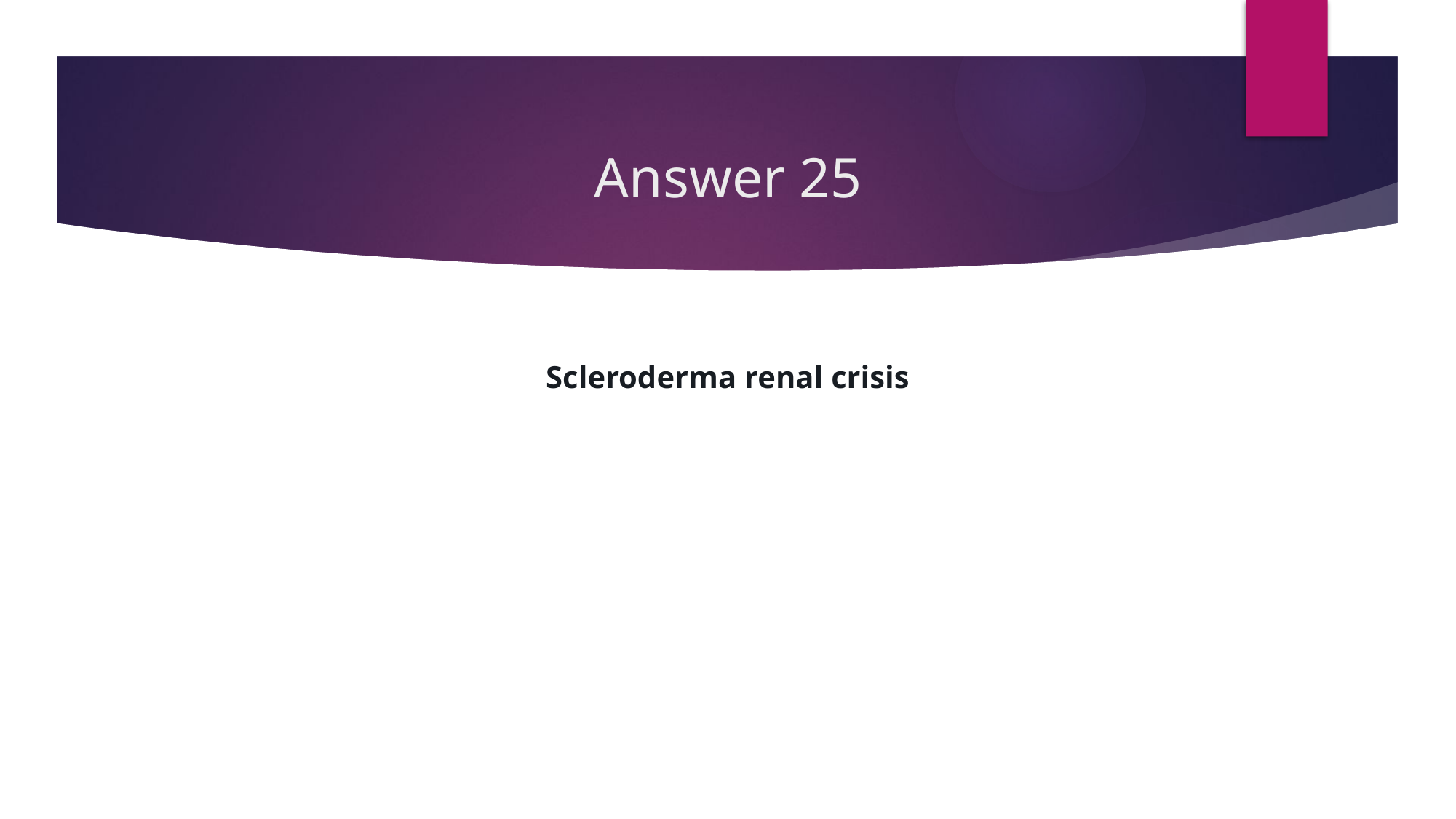

# Answer 25
Scleroderma renal crisis

## Slide 76
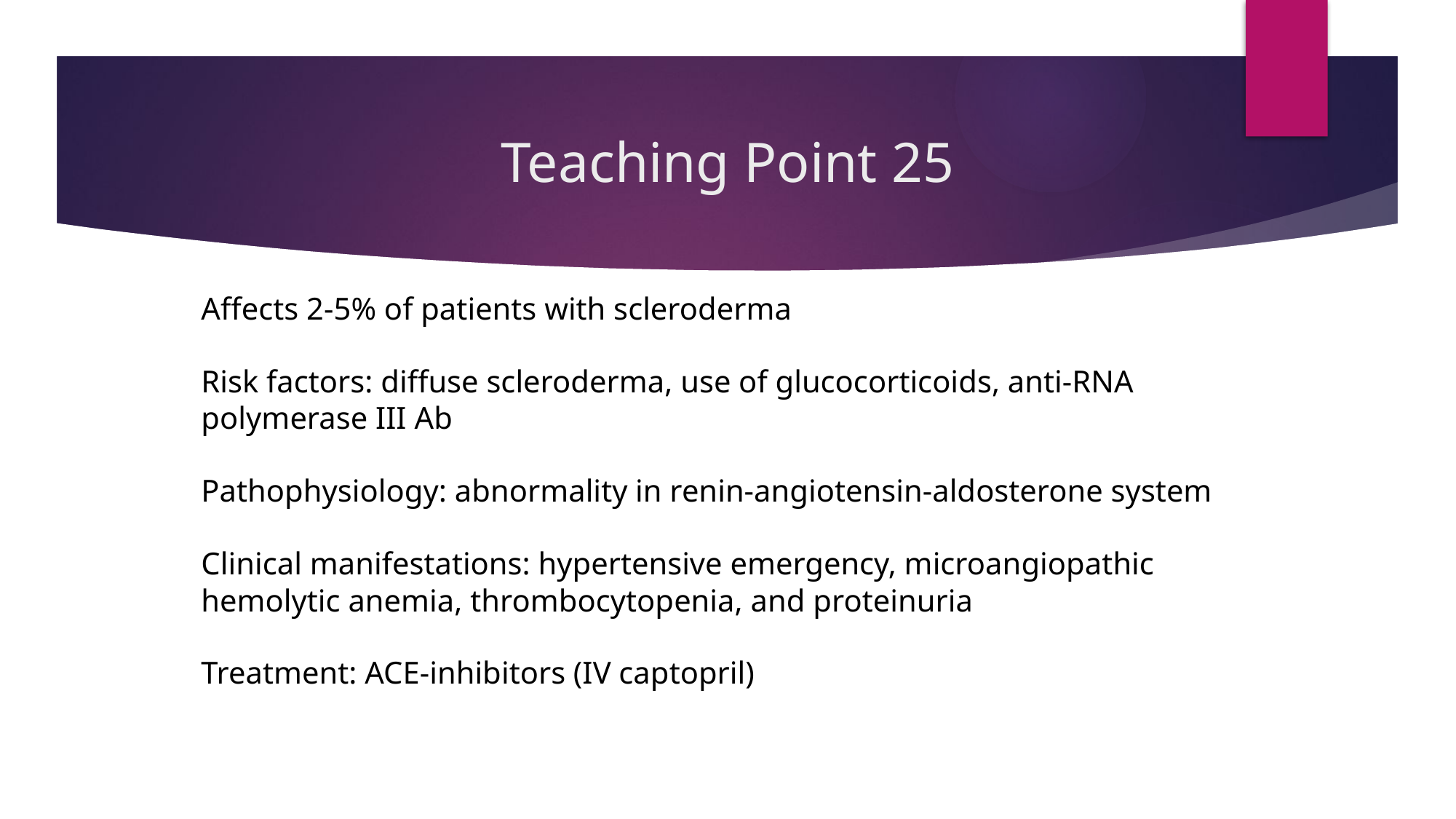

# Teaching Point 25
Affects 2-5% of patients with scleroderma
Risk factors: diffuse scleroderma, use of glucocorticoids, anti-RNA polymerase III Ab
Pathophysiology: abnormality in renin-angiotensin-aldosterone system
Clinical manifestations: hypertensive emergency, microangiopathic hemolytic anemia, thrombocytopenia, and proteinuria
Treatment: ACE-inhibitors (IV captopril)

## Slide 77
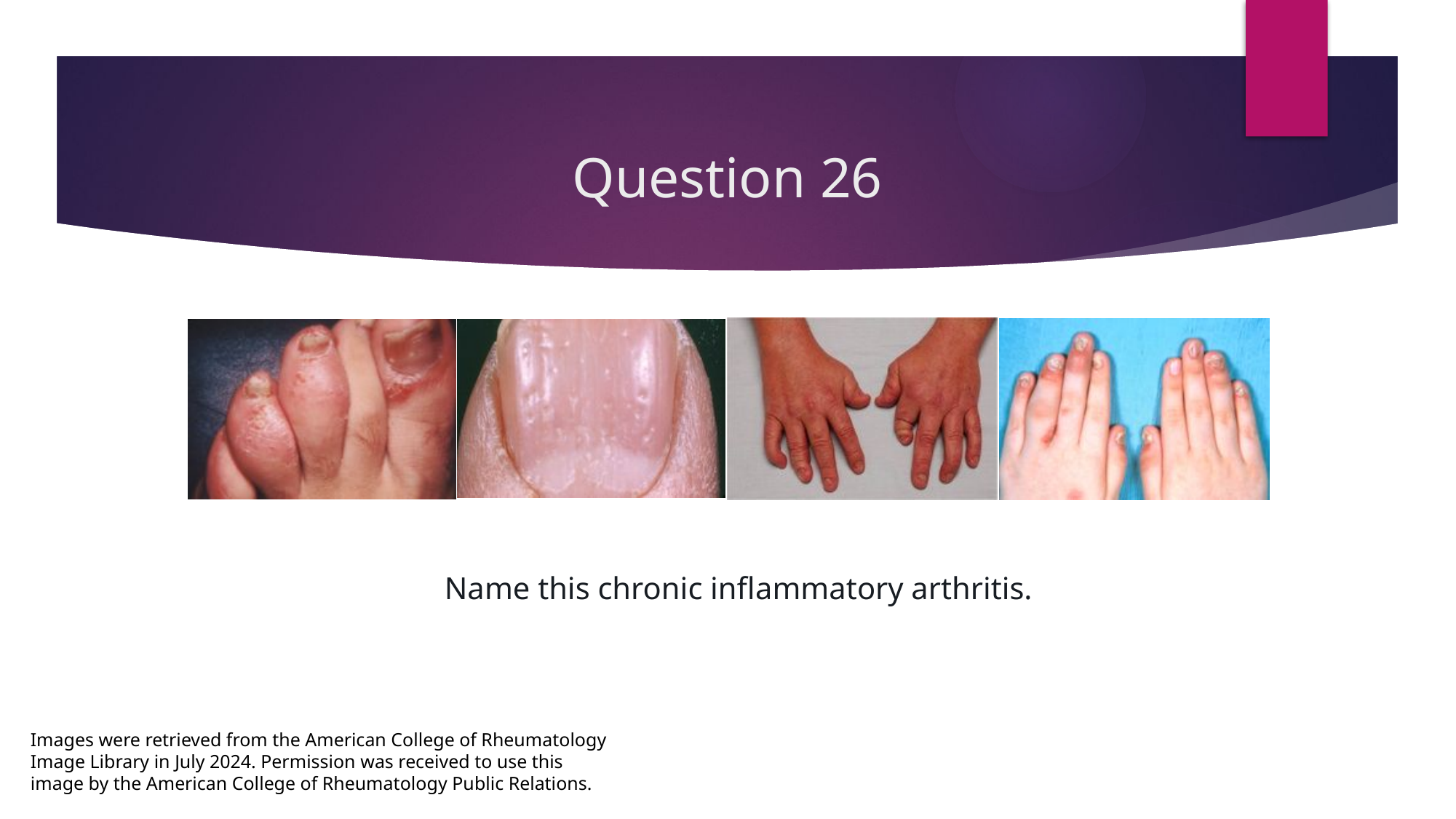

# Question 26
Name this chronic inflammatory arthritis.
Images were retrieved from the American College of Rheumatology Image Library in July 2024. Permission was received to use this image by the American College of Rheumatology Public Relations.

## Slide 78
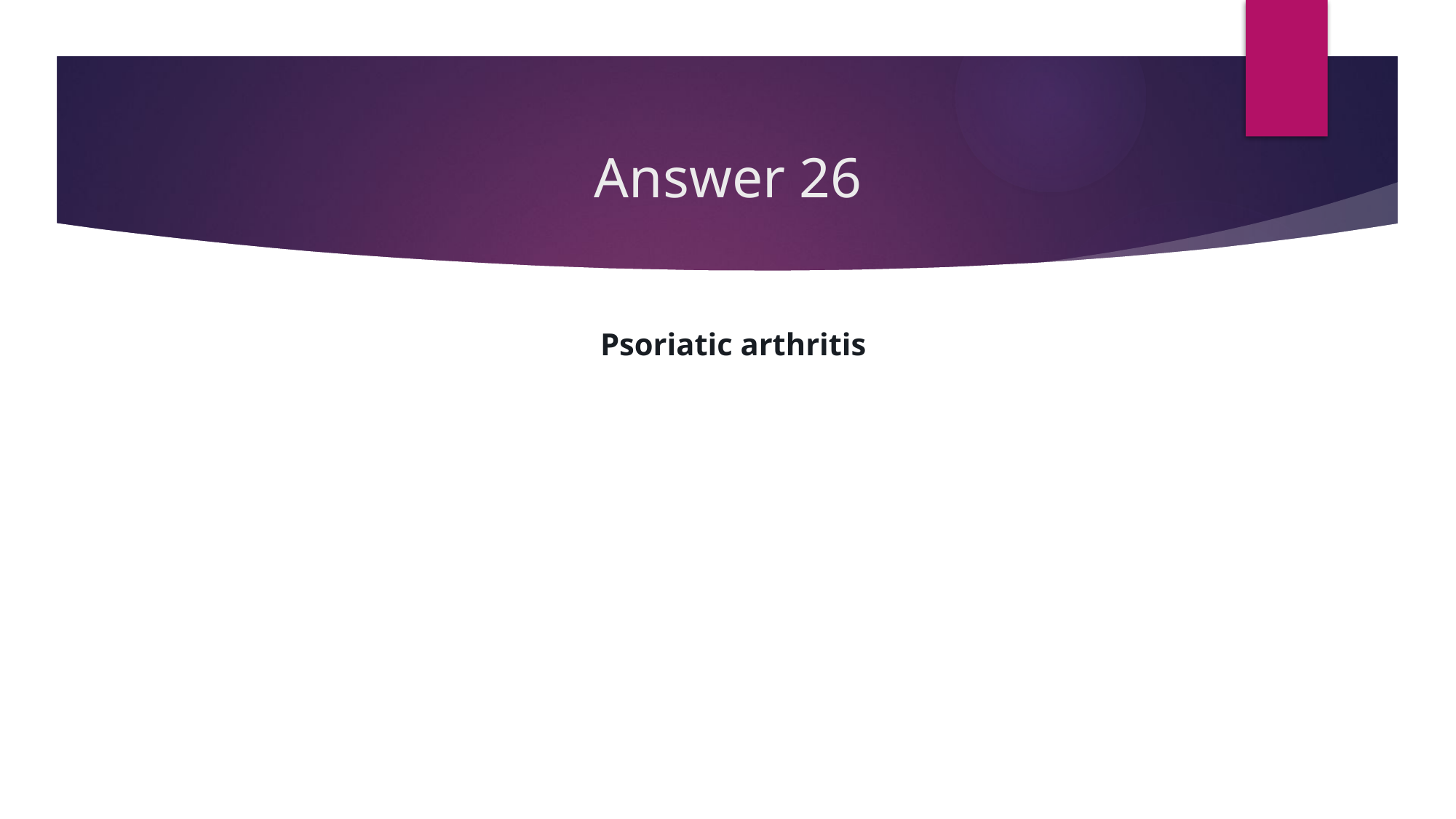

# Answer 26
Psoriatic arthritis

## Slide 79
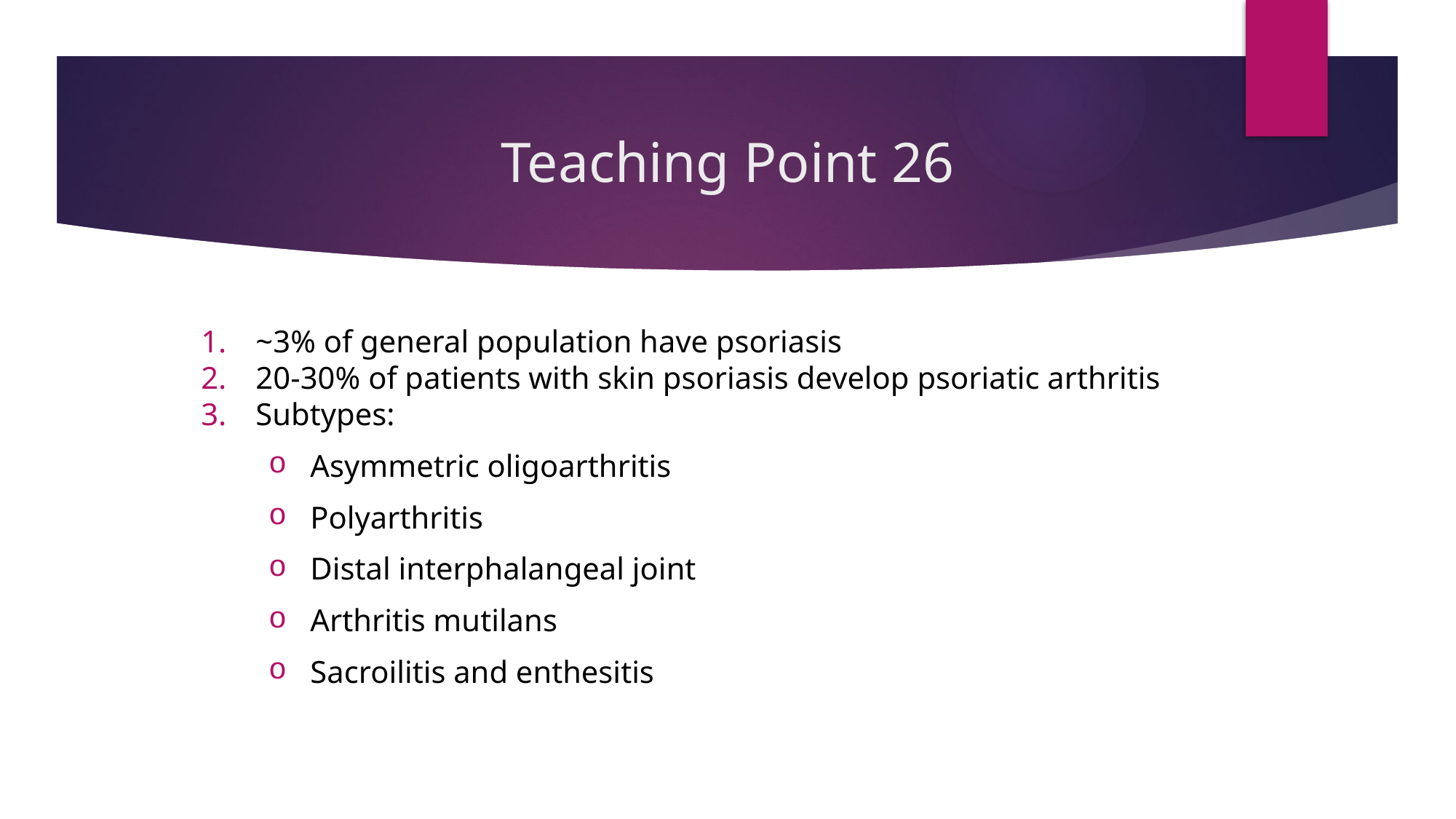

# Teaching Point 26
​
~3% of general population have psoriasis
20-30% of patients with skin psoriasis develop psoriatic arthritis
Subtypes:
Asymmetric oligoarthritis
Polyarthritis
Distal interphalangeal joint
Arthritis mutilans
Sacroilitis and enthesitis

## Slide 80
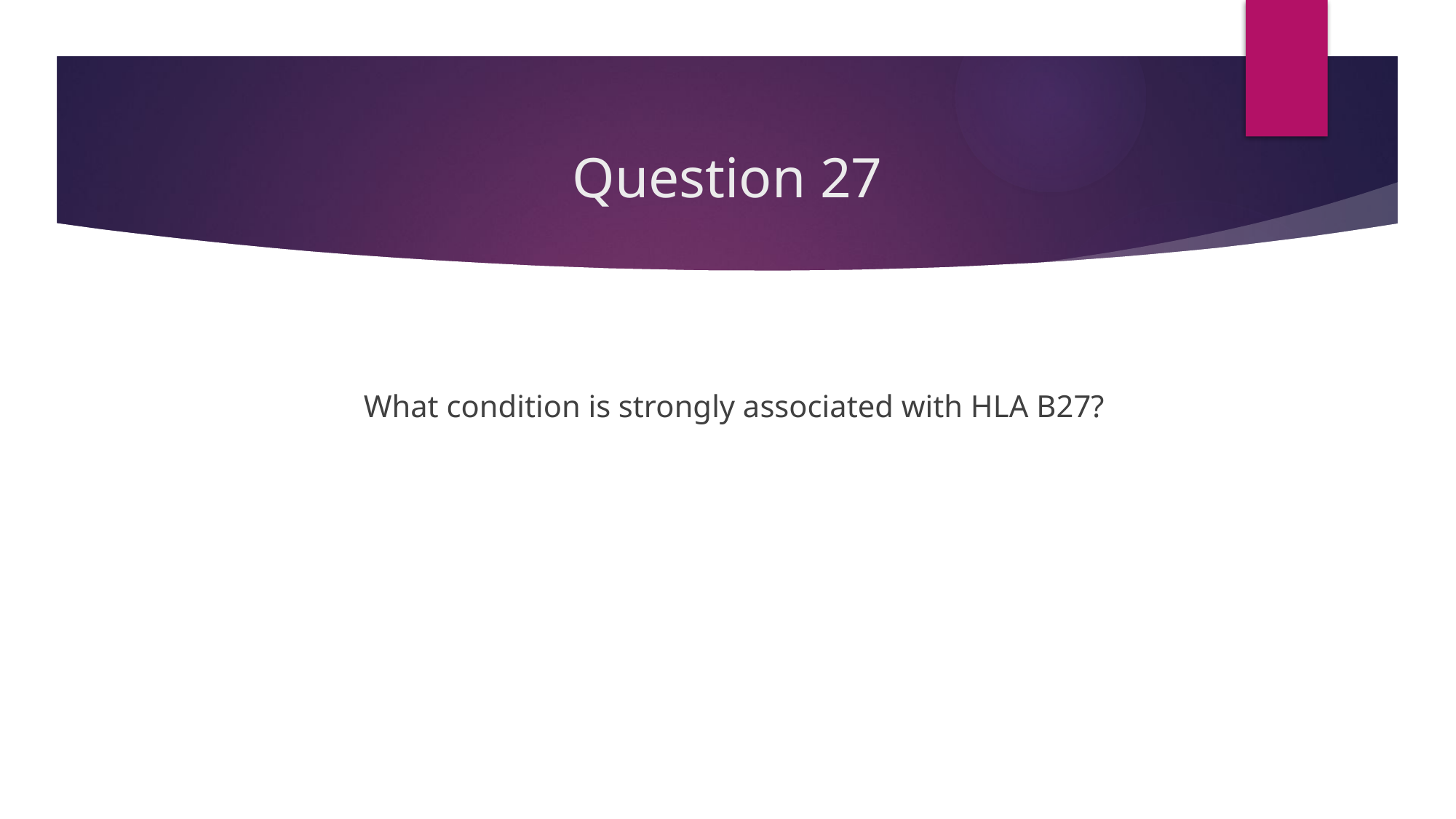

# Question 27
What condition is strongly associated with HLA B27?

## Slide 81
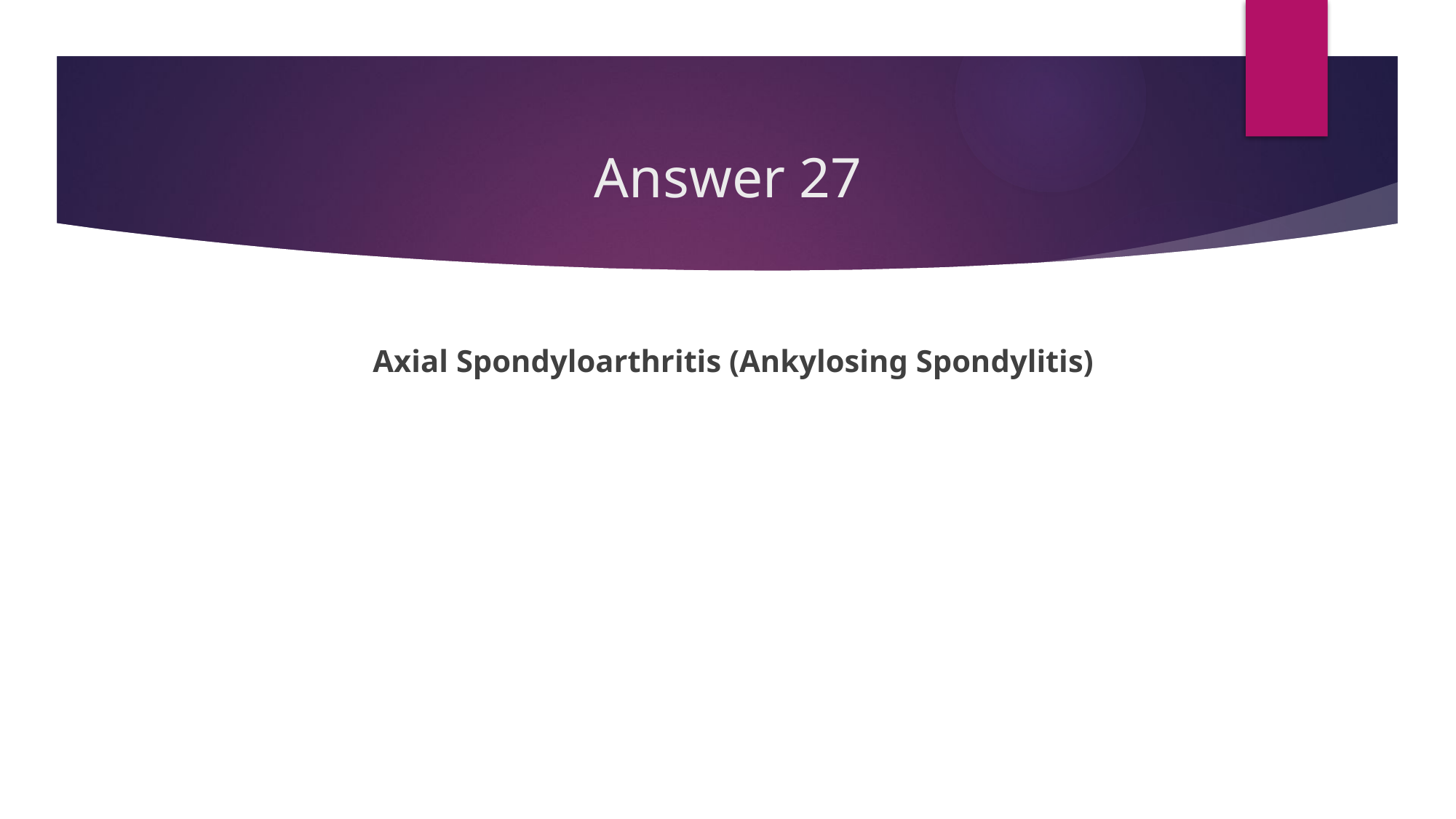

# Answer 27
Axial Spondyloarthritis (Ankylosing Spondylitis)

## Slide 82
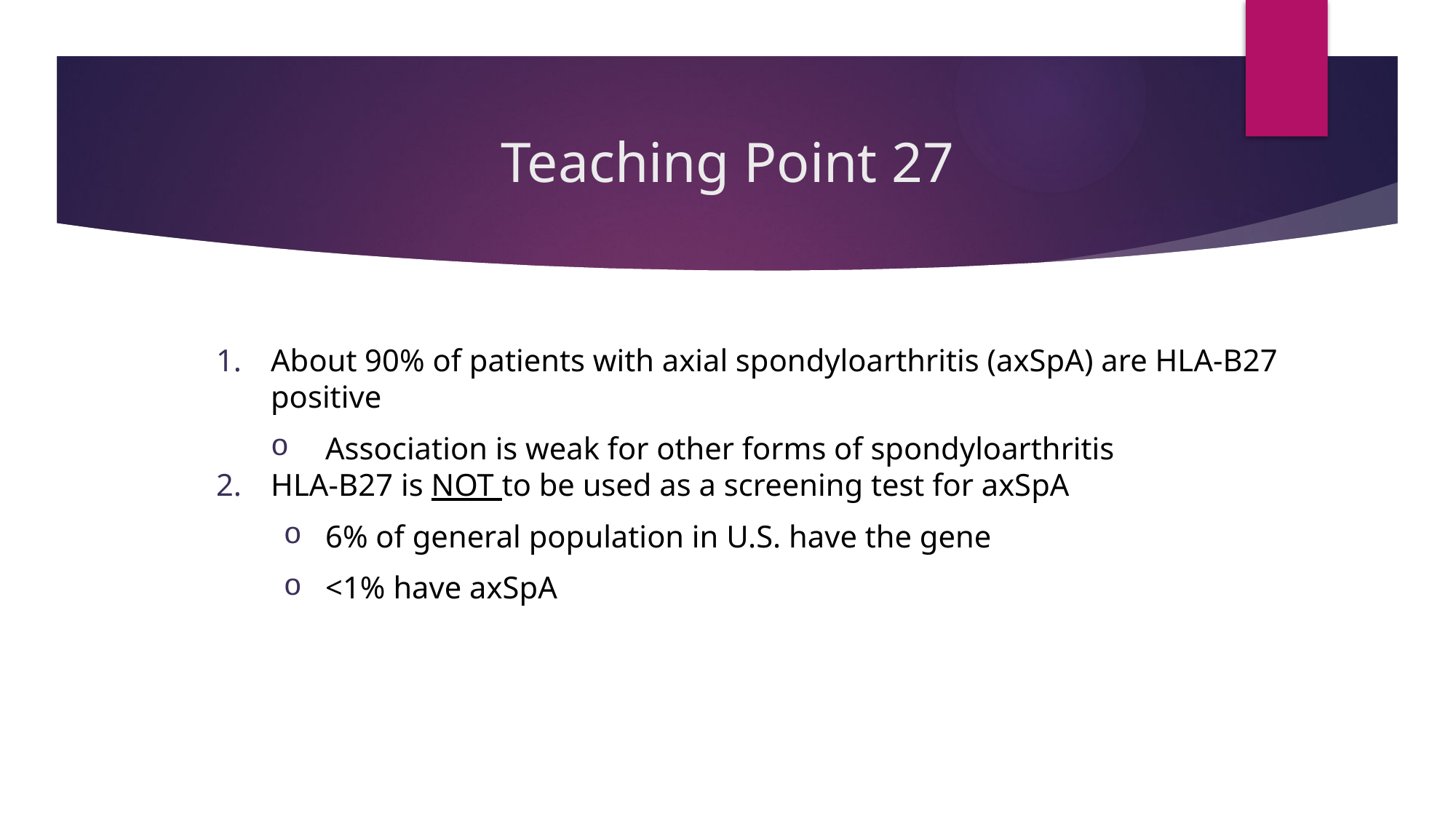

# Teaching Point 27
​
About 90% of patients with axial spondyloarthritis (axSpA) are HLA-B27 positive
Association is weak for other forms of spondyloarthritis
HLA-B27 is NOT to be used as a screening test for axSpA
6% of general population in U.S. have the gene
<1% have axSpA

## Slide 83
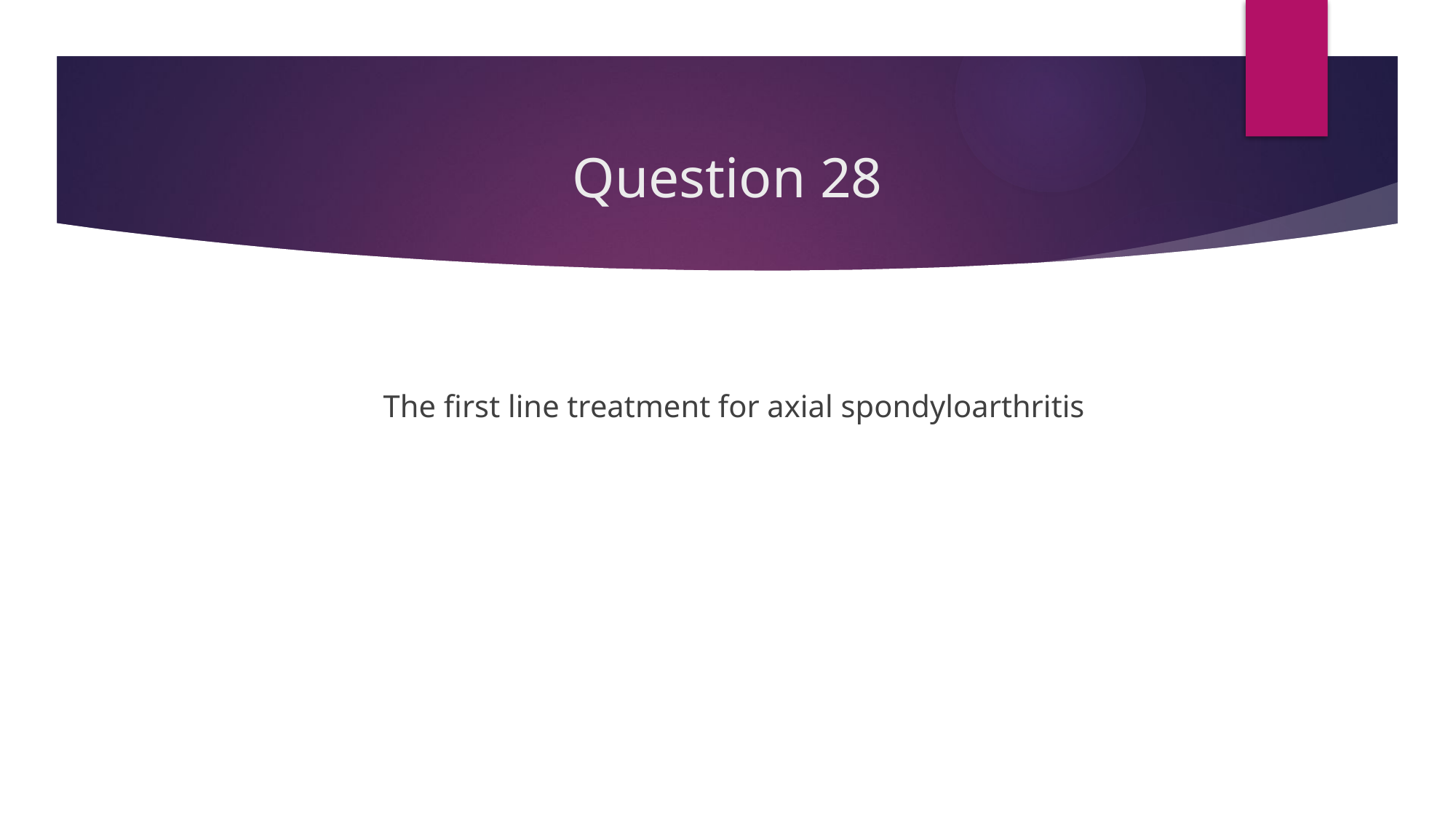

# Question 28
The first line treatment for axial spondyloarthritis

## Slide 84
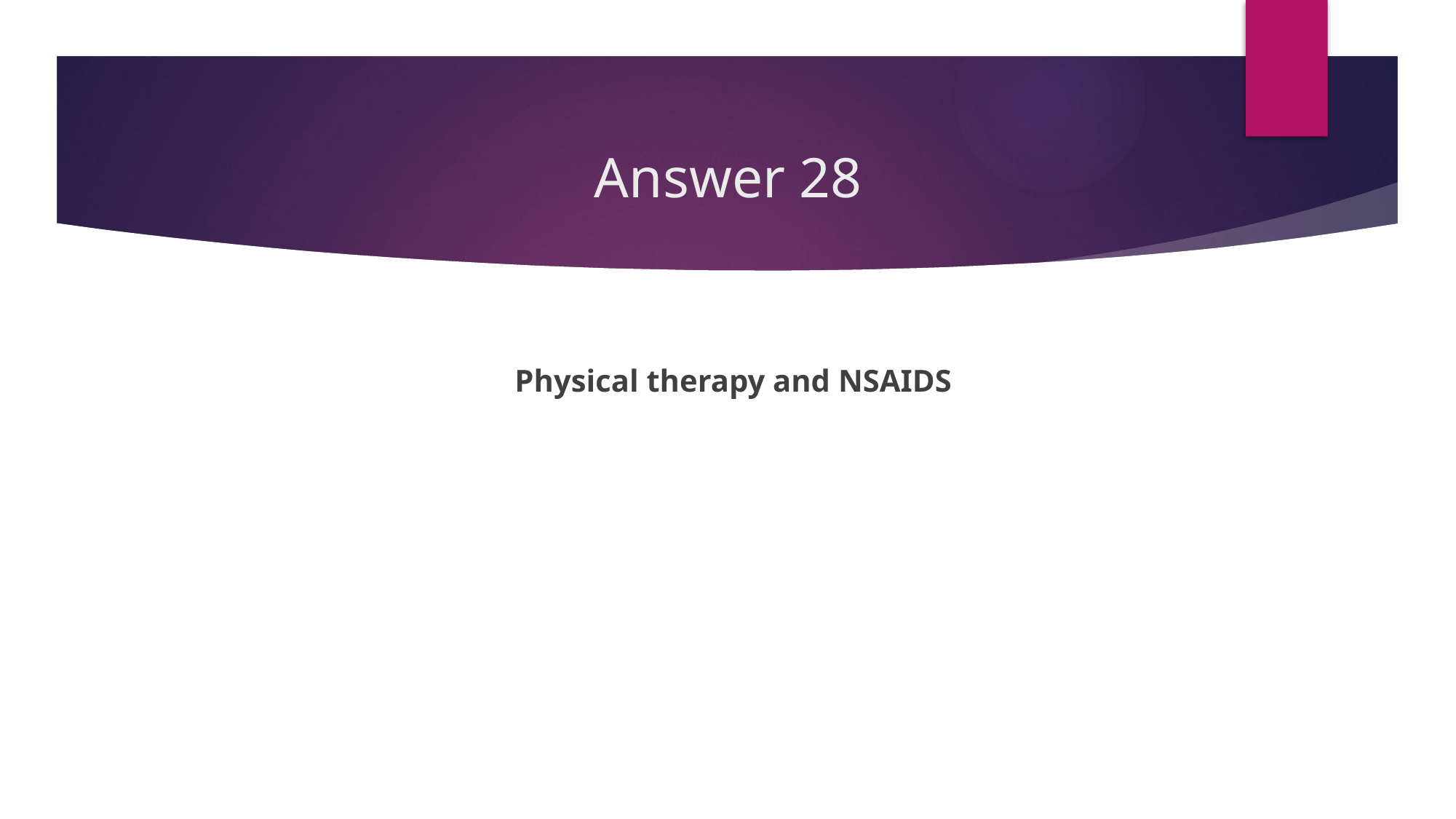

# Answer 28
Physical therapy and NSAIDS

## Slide 85
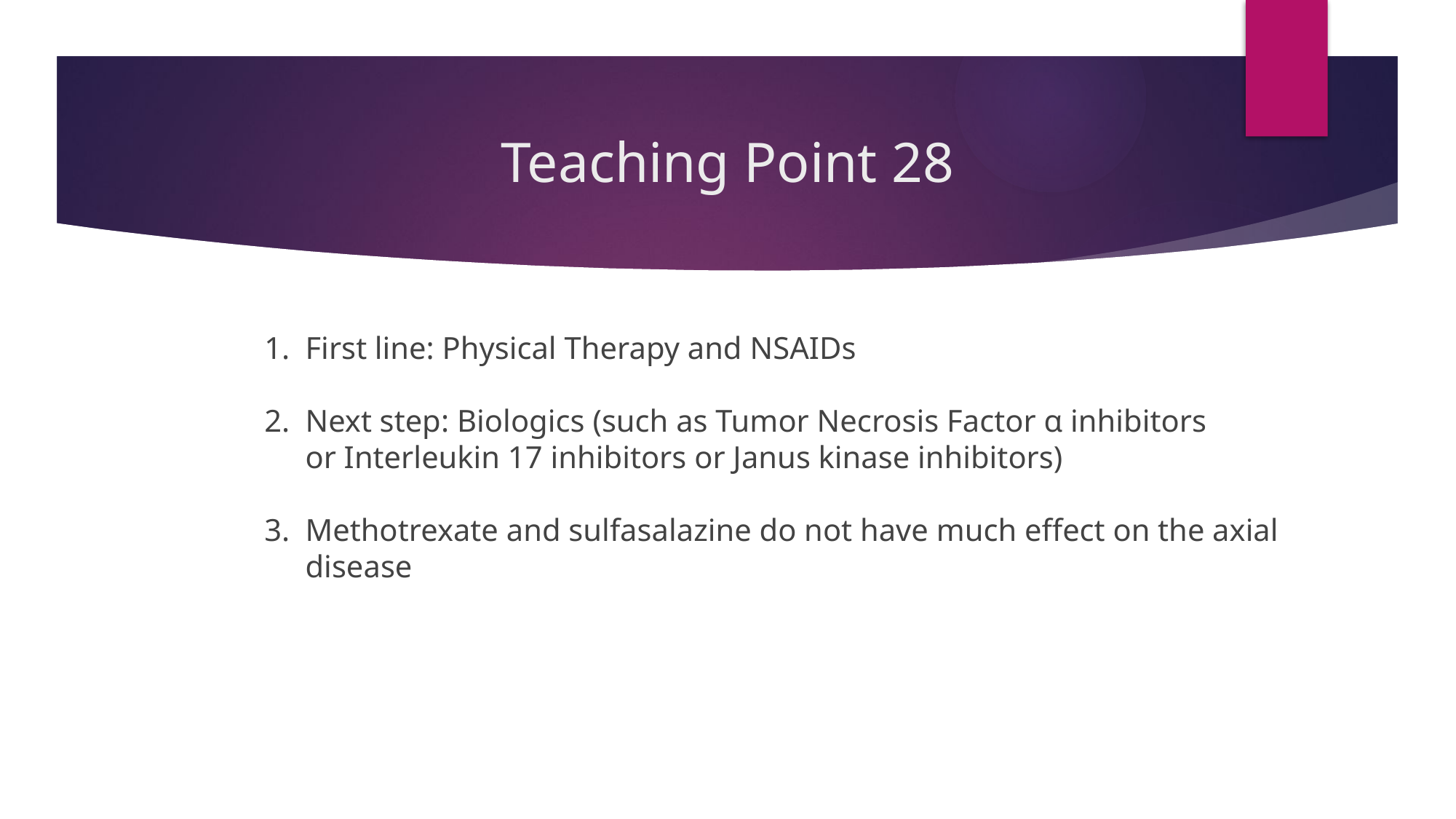

# Teaching Point 28
​
First line: Physical Therapy and NSAIDs
Next step: Biologics (such as Tumor Necrosis Factor α inhibitors or Interleukin 17 inhibitors or Janus kinase inhibitors)
Methotrexate and sulfasalazine do not have much effect on the axial disease

## Slide 86
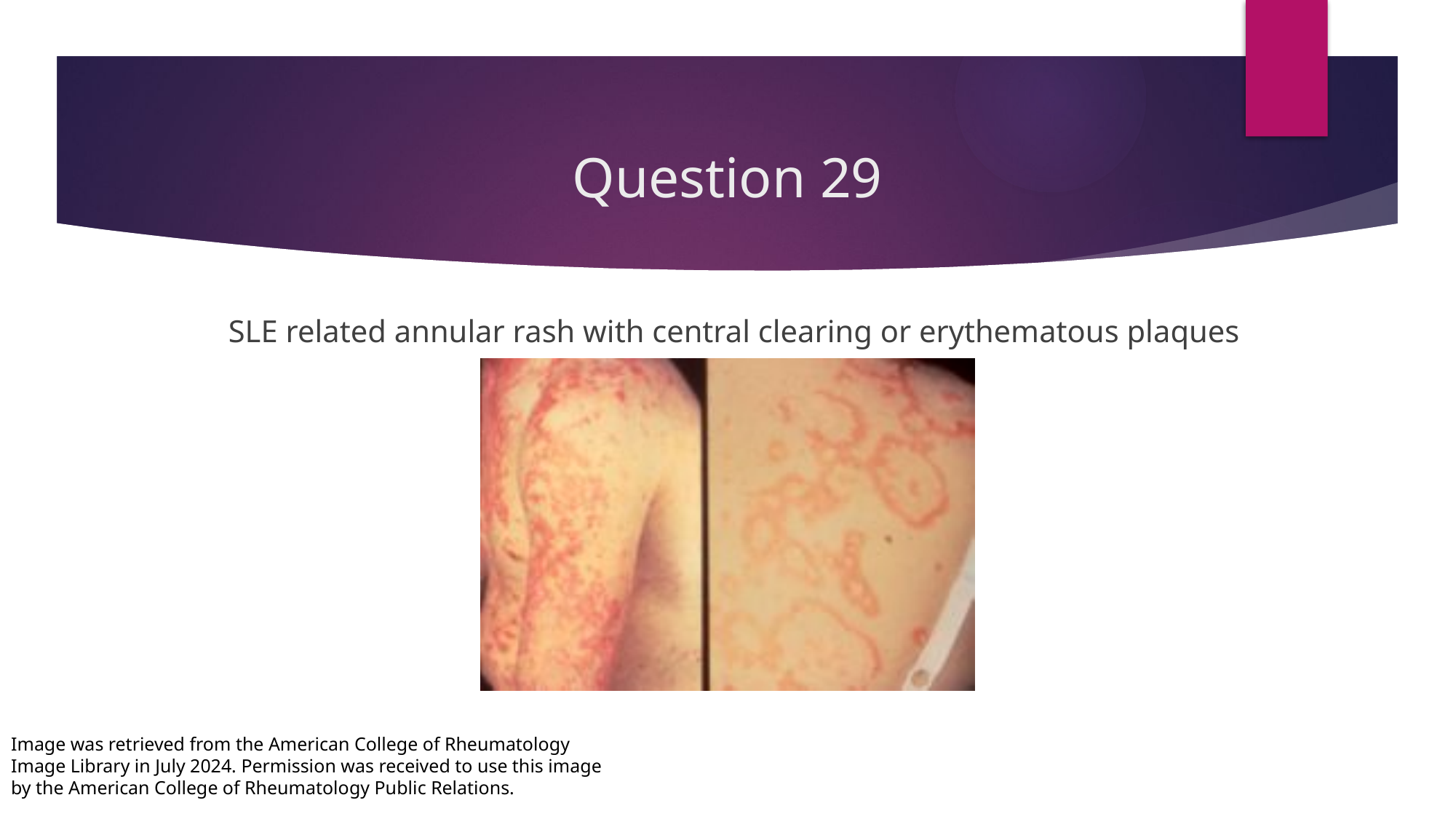

# Question 29
SLE related annular rash with central clearing or erythematous plaques
Image was retrieved from the American College of Rheumatology Image Library in July 2024. Permission was received to use this image by the American College of Rheumatology Public Relations.

## Slide 87
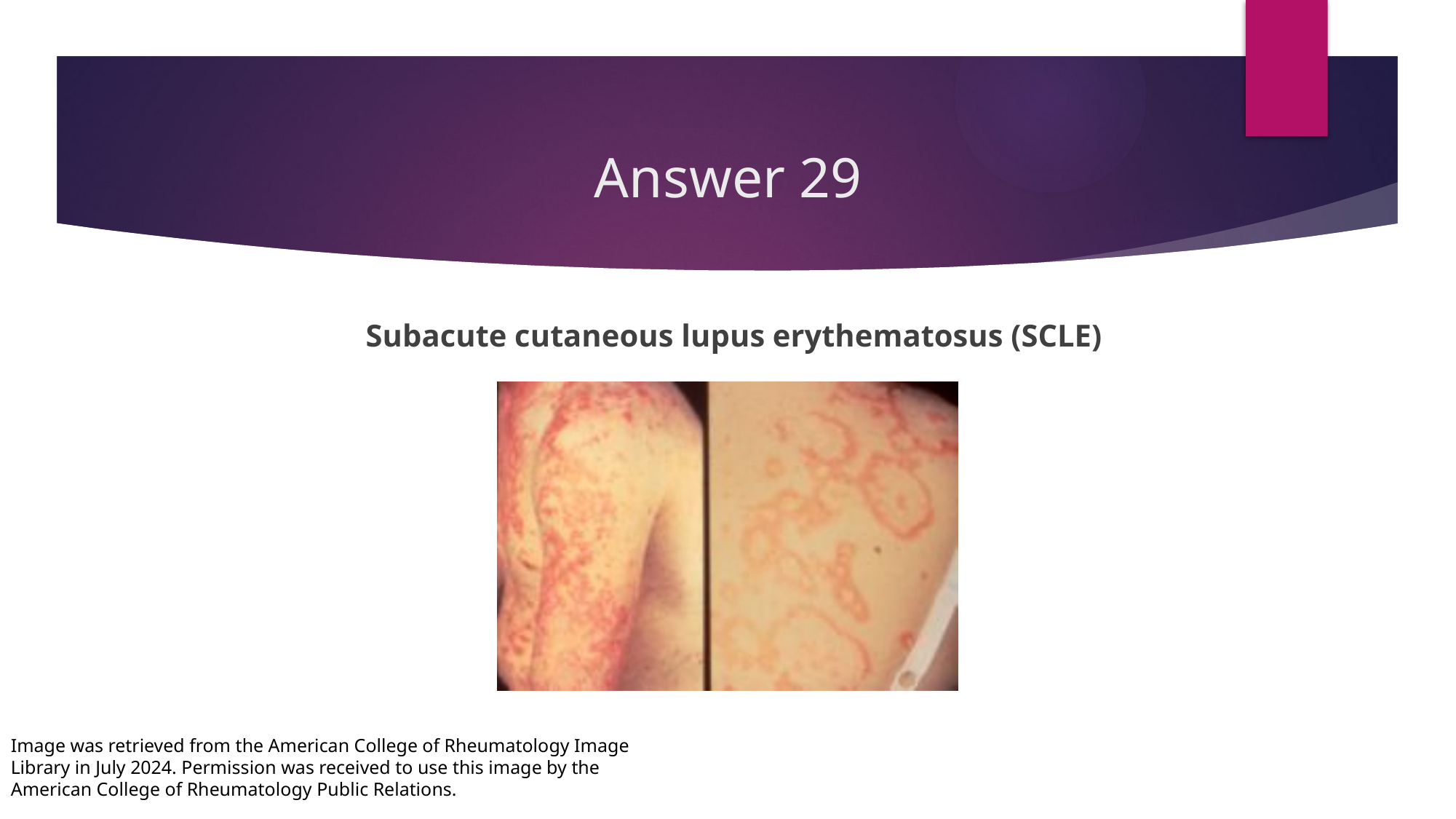

# Answer 29
Subacute cutaneous lupus erythematosus (SCLE)
Image was retrieved from the American College of Rheumatology Image Library in July 2024. Permission was received to use this image by the American College of Rheumatology Public Relations.

## Slide 88
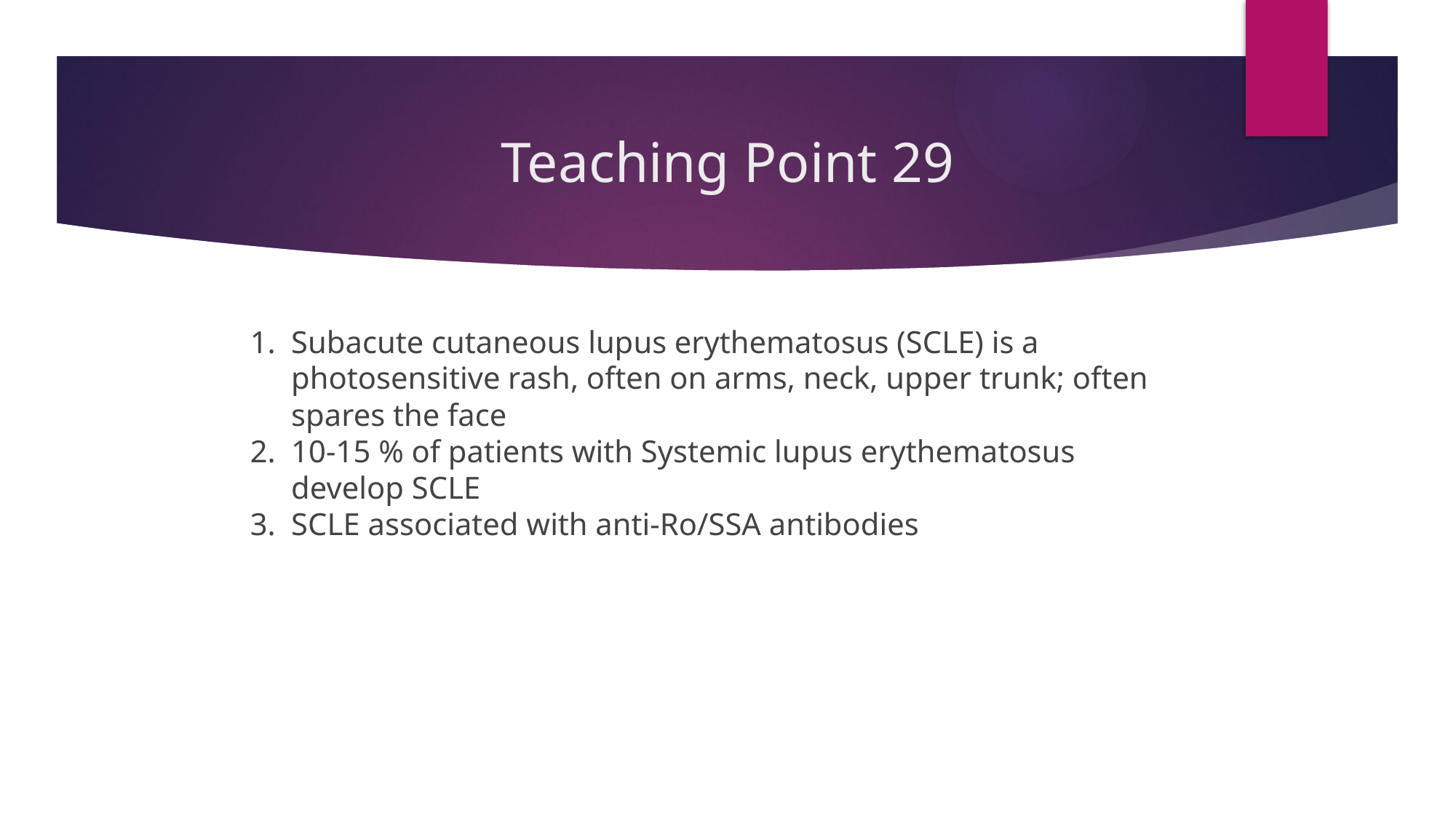

# Teaching Point 29
​
Subacute cutaneous lupus erythematosus (SCLE) is a photosensitive rash, often on arms, neck, upper trunk; often spares the face
10-15 % of patients with Systemic lupus erythematosus develop SCLE​
SCLE associated with anti-Ro/SSA antibodies

## Slide 89
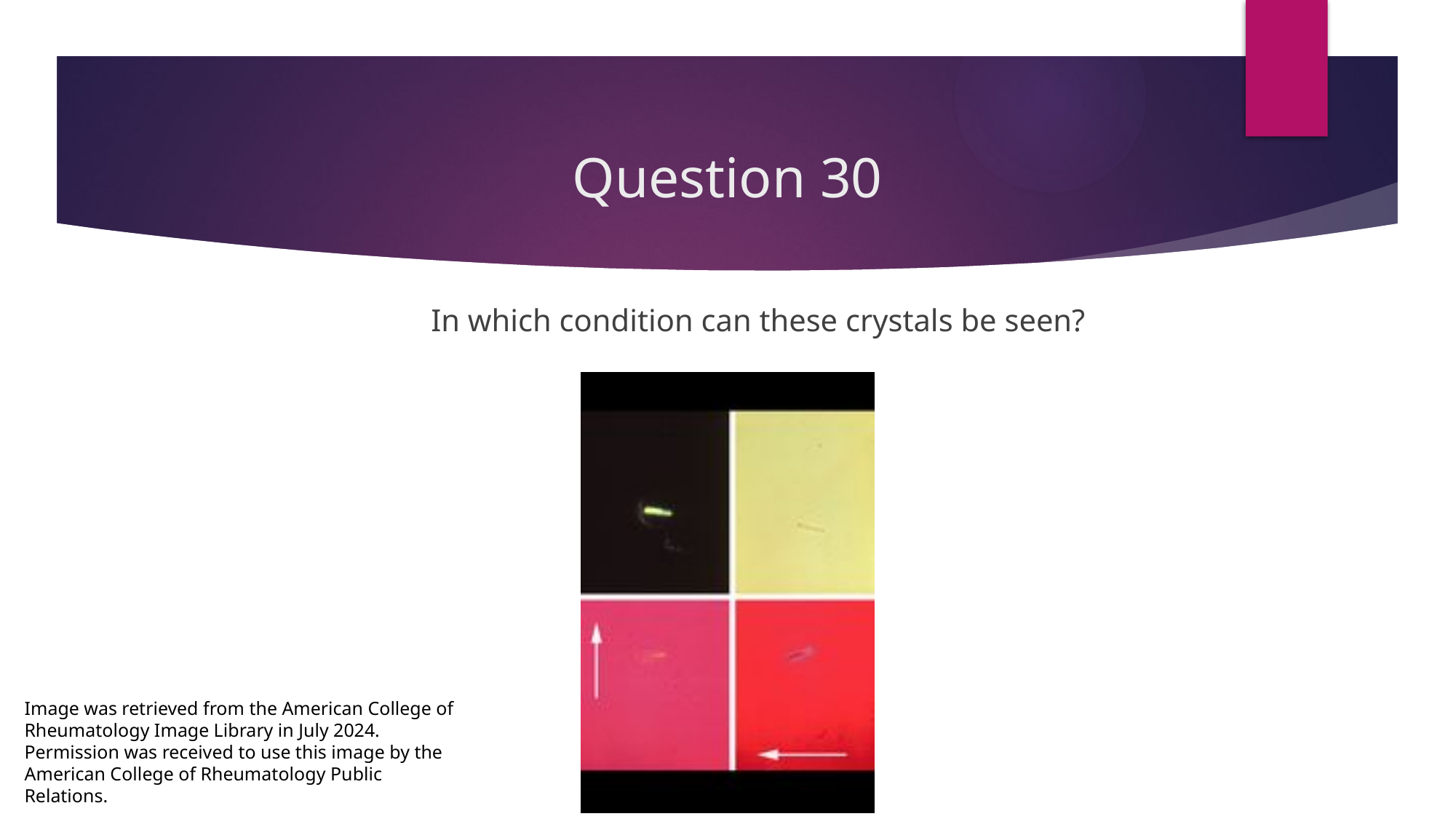

# Question 30
In which condition can these crystals be seen?
Image was retrieved from the American College of Rheumatology Image Library in July 2024. Permission was received to use this image by the American College of Rheumatology Public Relations.

## Slide 90
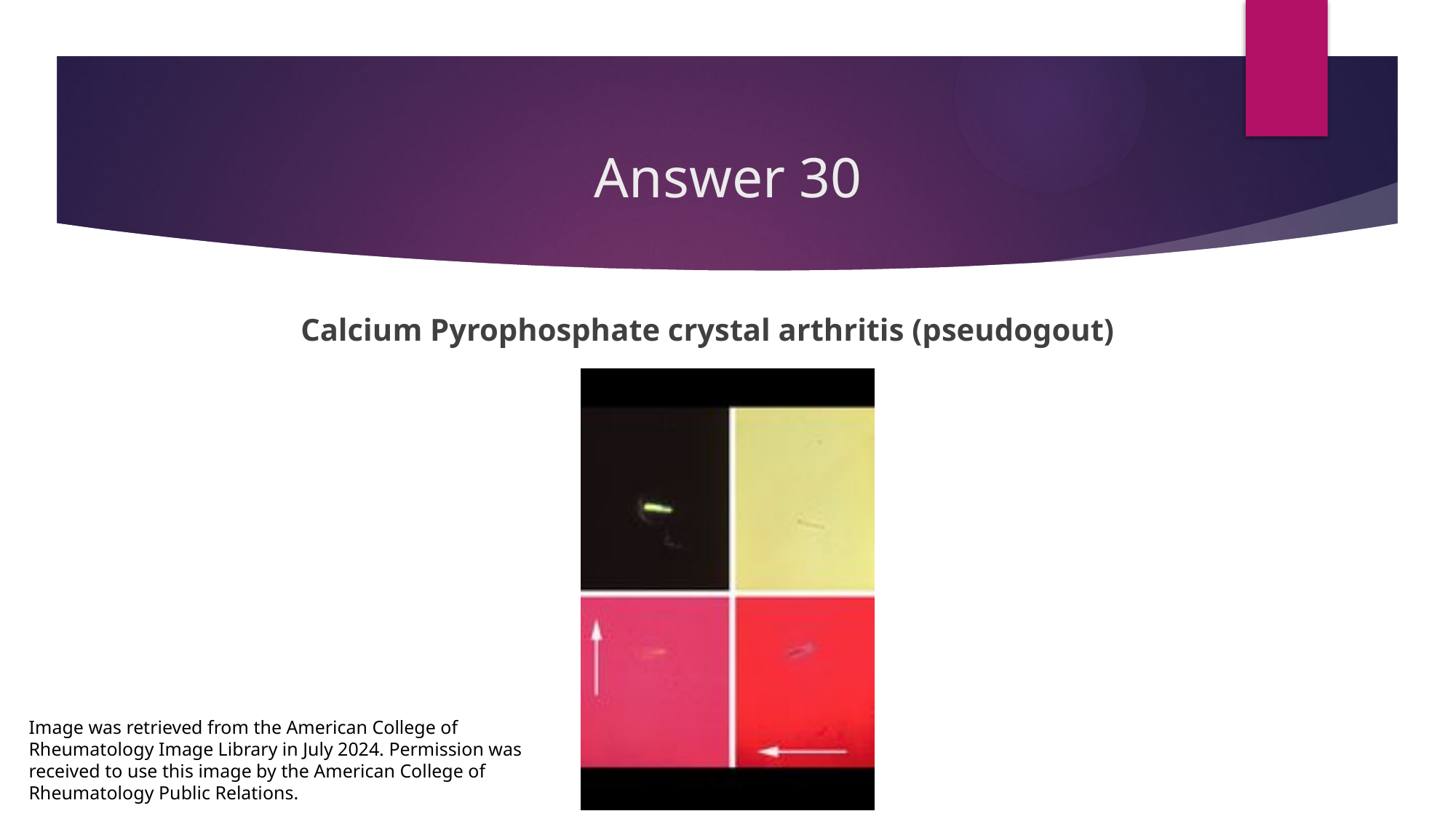

# Answer 30
Calcium Pyrophosphate crystal arthritis (pseudogout)
Image was retrieved from the American College of Rheumatology Image Library in July 2024. Permission was received to use this image by the American College of Rheumatology Public Relations.

## Slide 91
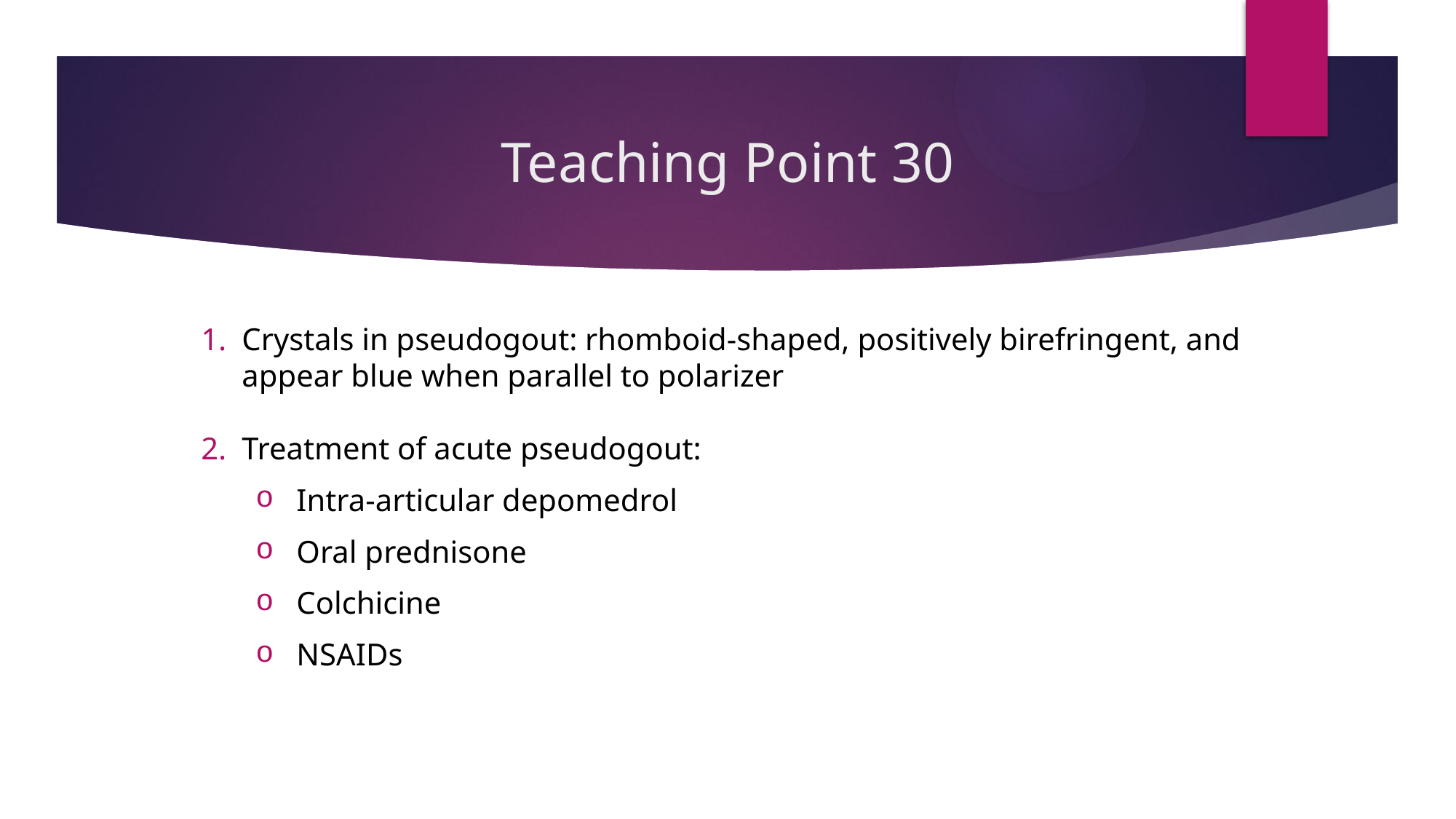

# Teaching Point 30
​
Crystals in pseudogout: rhomboid-shaped, positively birefringent, and appear blue when parallel to polarizer
Treatment of acute pseudogout:
Intra-articular depomedrol
Oral prednisone
Colchicine
NSAIDs

## Slide 92
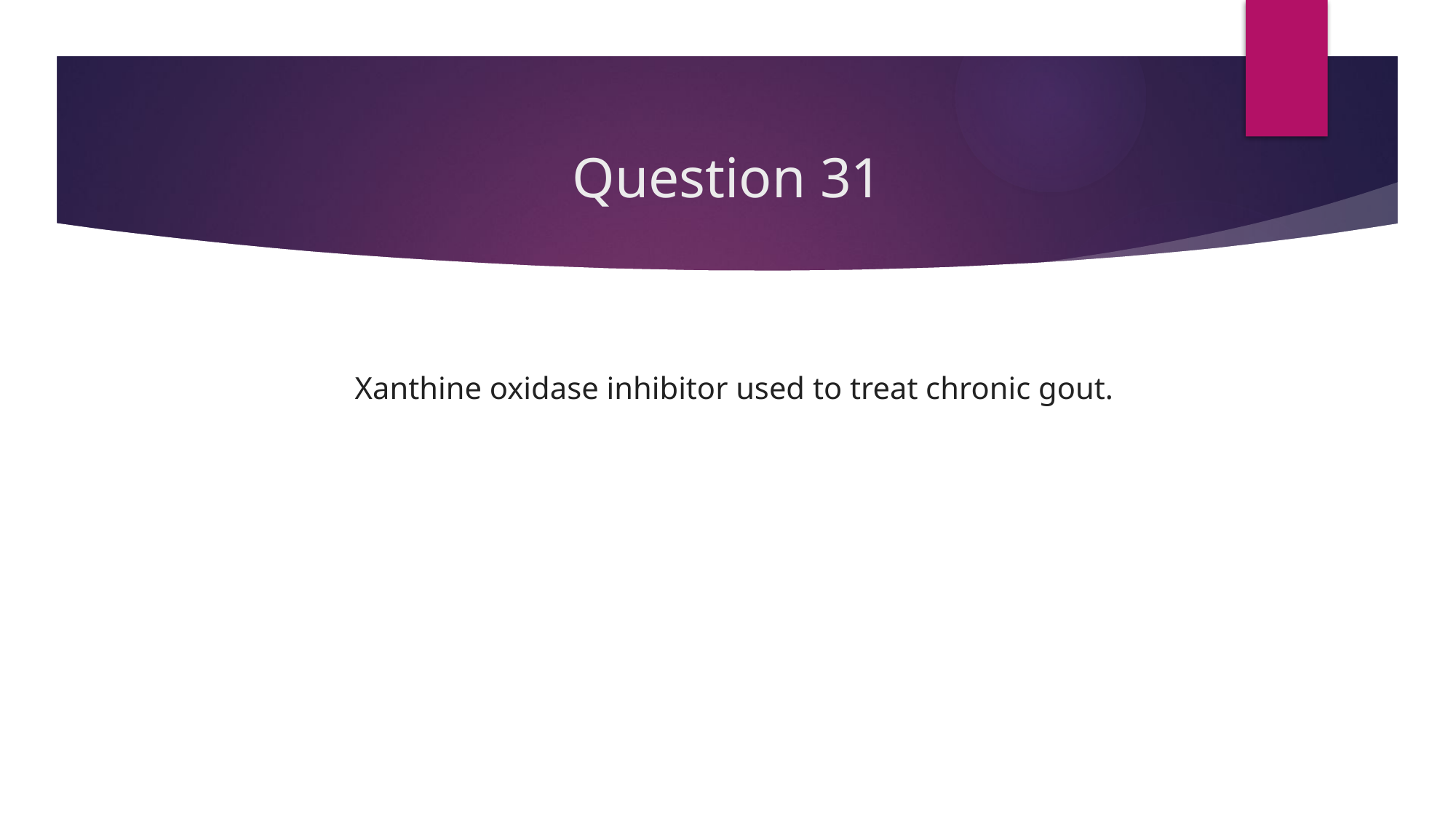

# Question 31
Xanthine oxidase inhibitor used to treat chronic gout.

## Slide 93
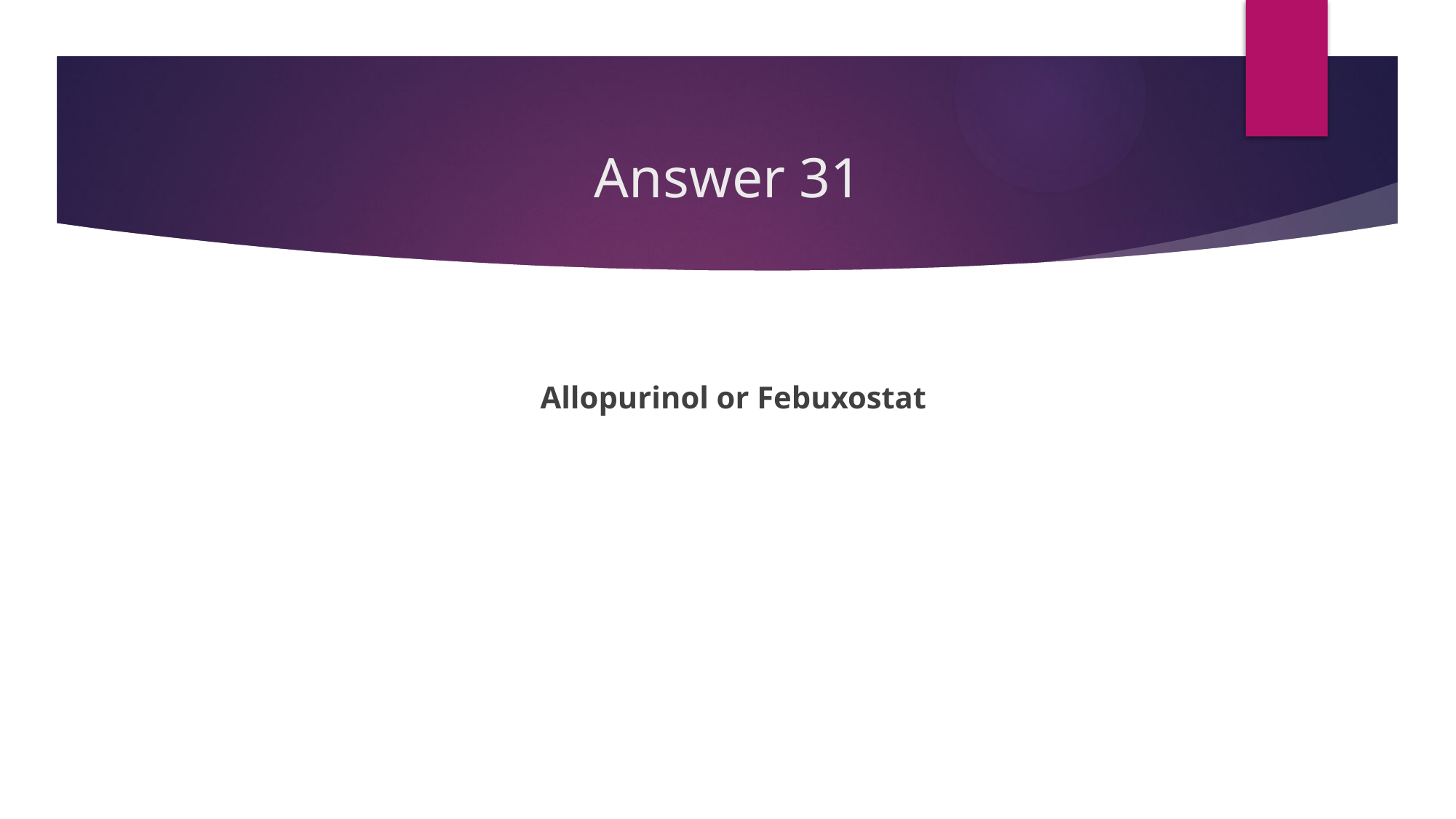

# Answer 31
Allopurinol or Febuxostat

## Slide 94
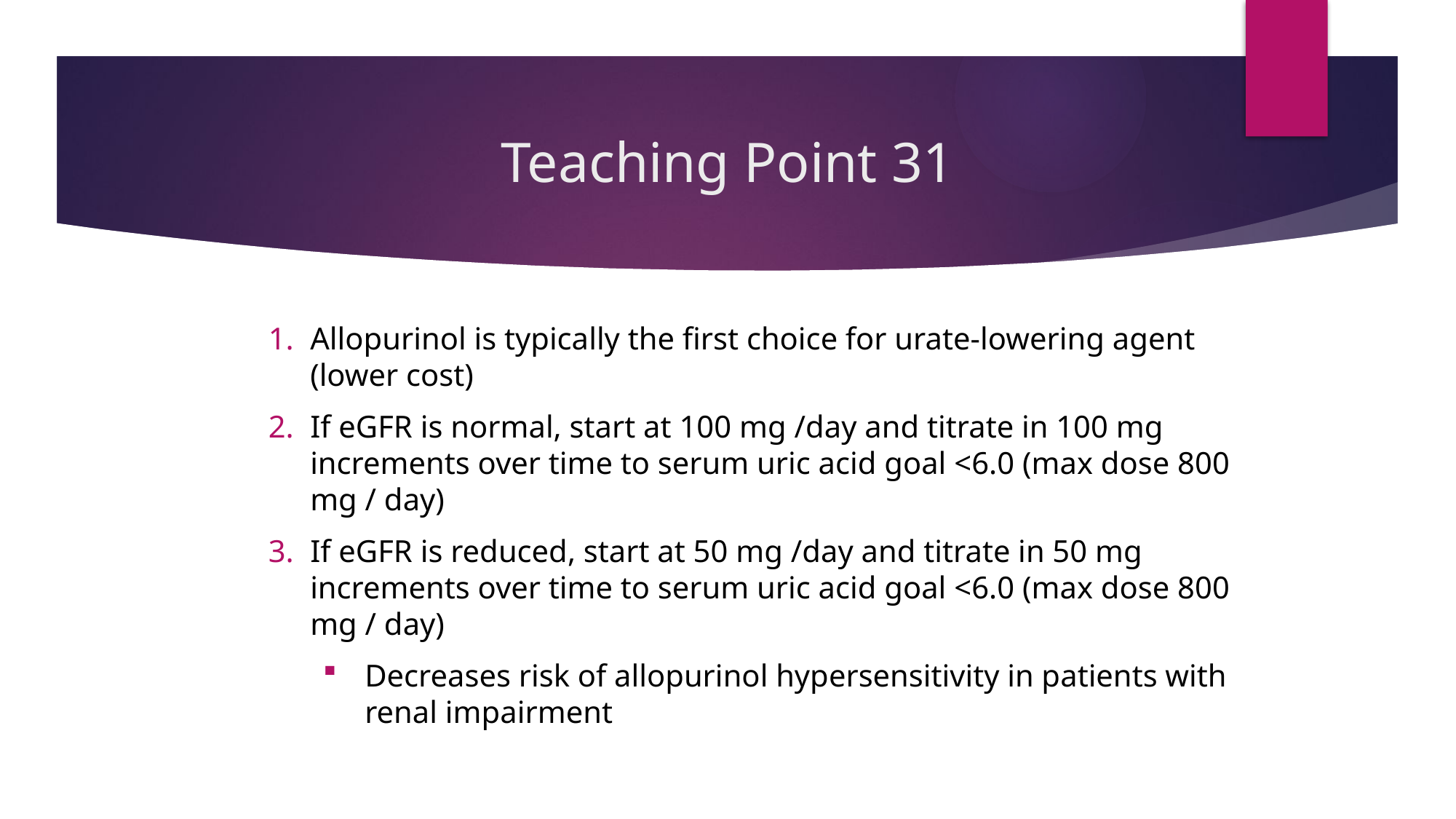

# Teaching Point 31
​
Allopurinol is typically the first choice for urate-lowering agent (lower cost)
If eGFR is normal, start at 100 mg /day and titrate in 100 mg increments over time to serum uric acid goal <6.0 (max dose 800 mg / day)
If eGFR is reduced, start at 50 mg /day and titrate in 50 mg increments over time to serum uric acid goal <6.0 (max dose 800 mg / day)
Decreases risk of allopurinol hypersensitivity in patients with renal impairment

## Slide 95
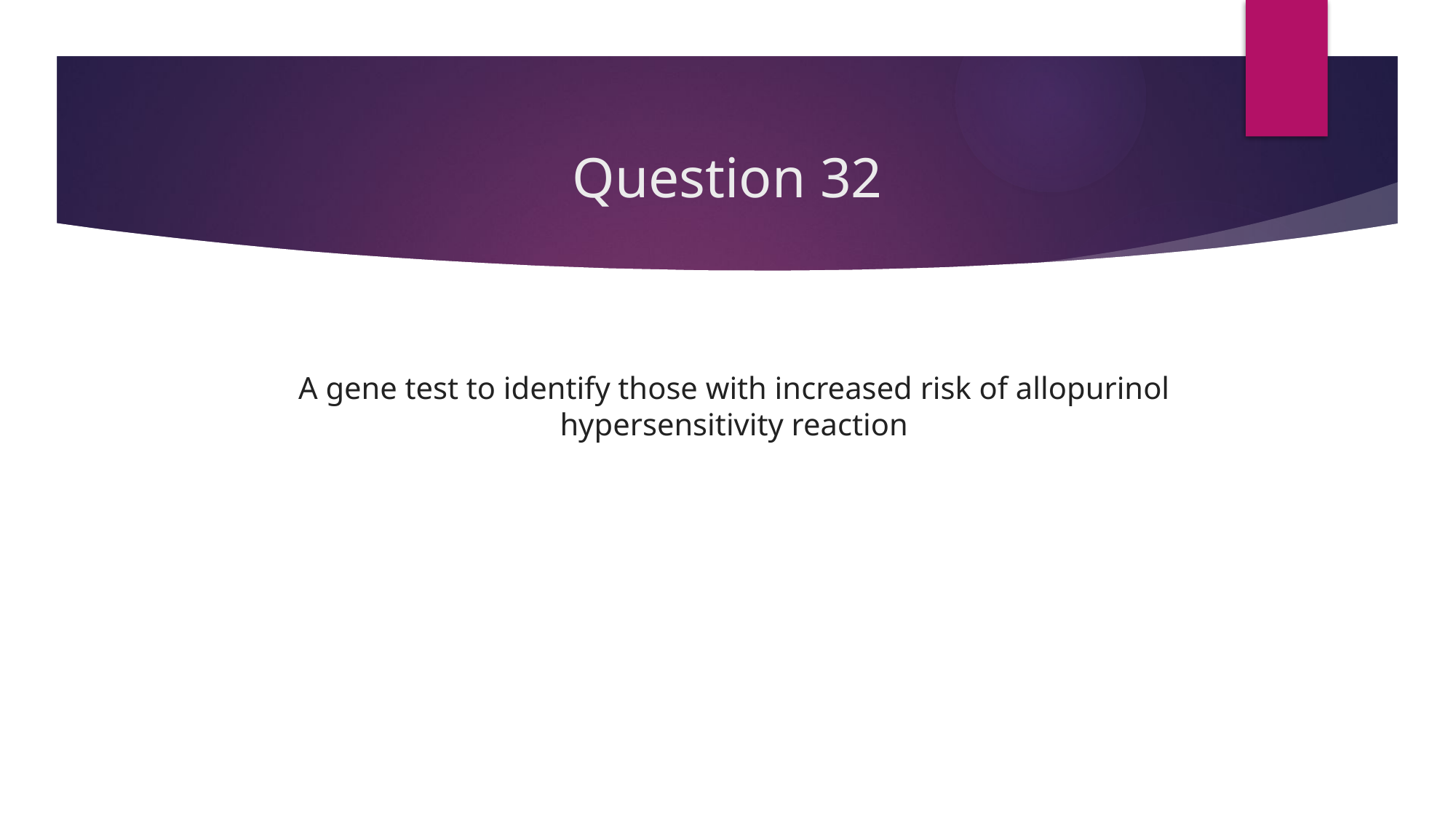

# Question 32
A gene test to identify those with increased risk of allopurinol hypersensitivity reaction

## Slide 96
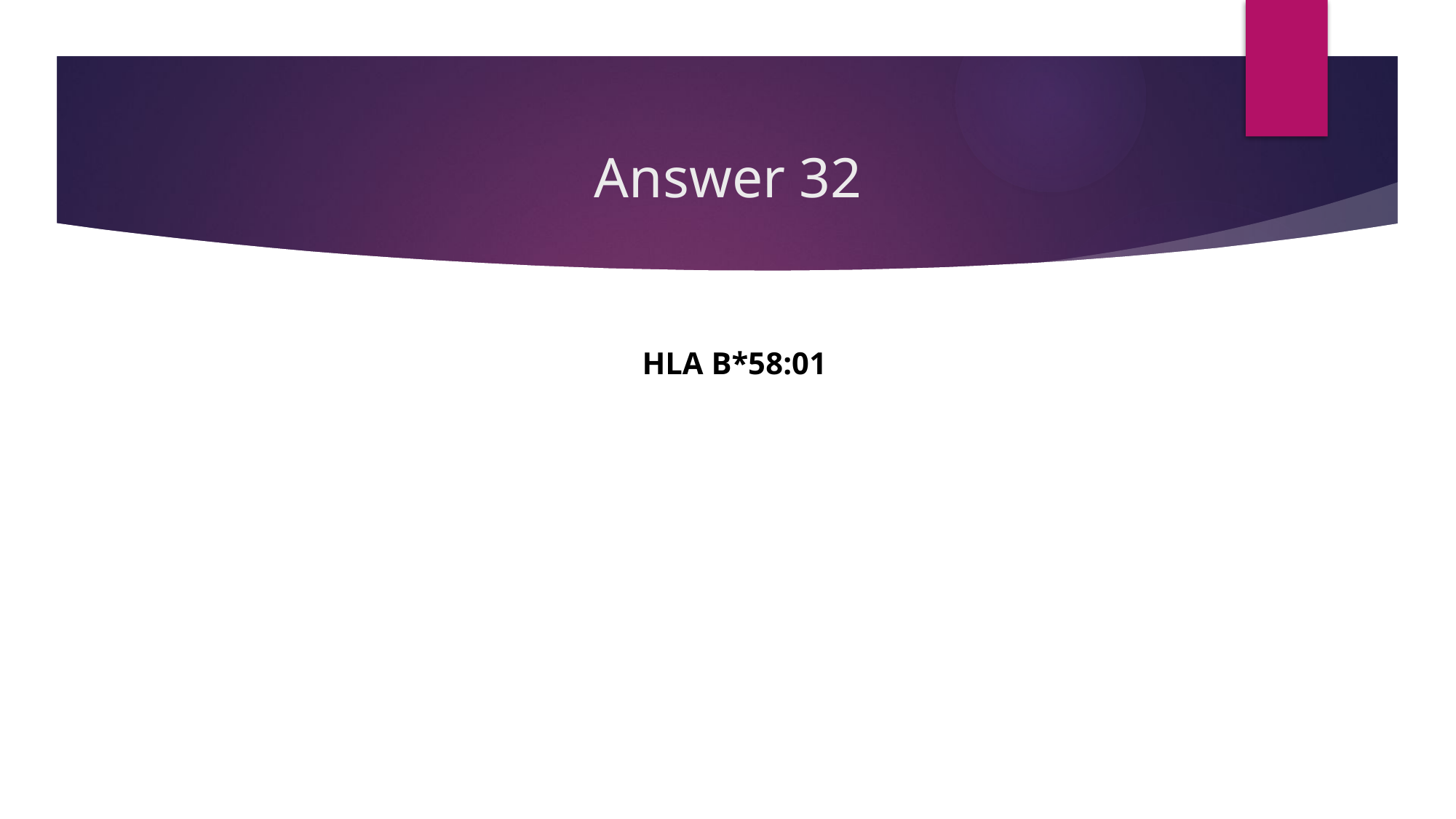

# Answer 32
HLA B*58:01

## Slide 97
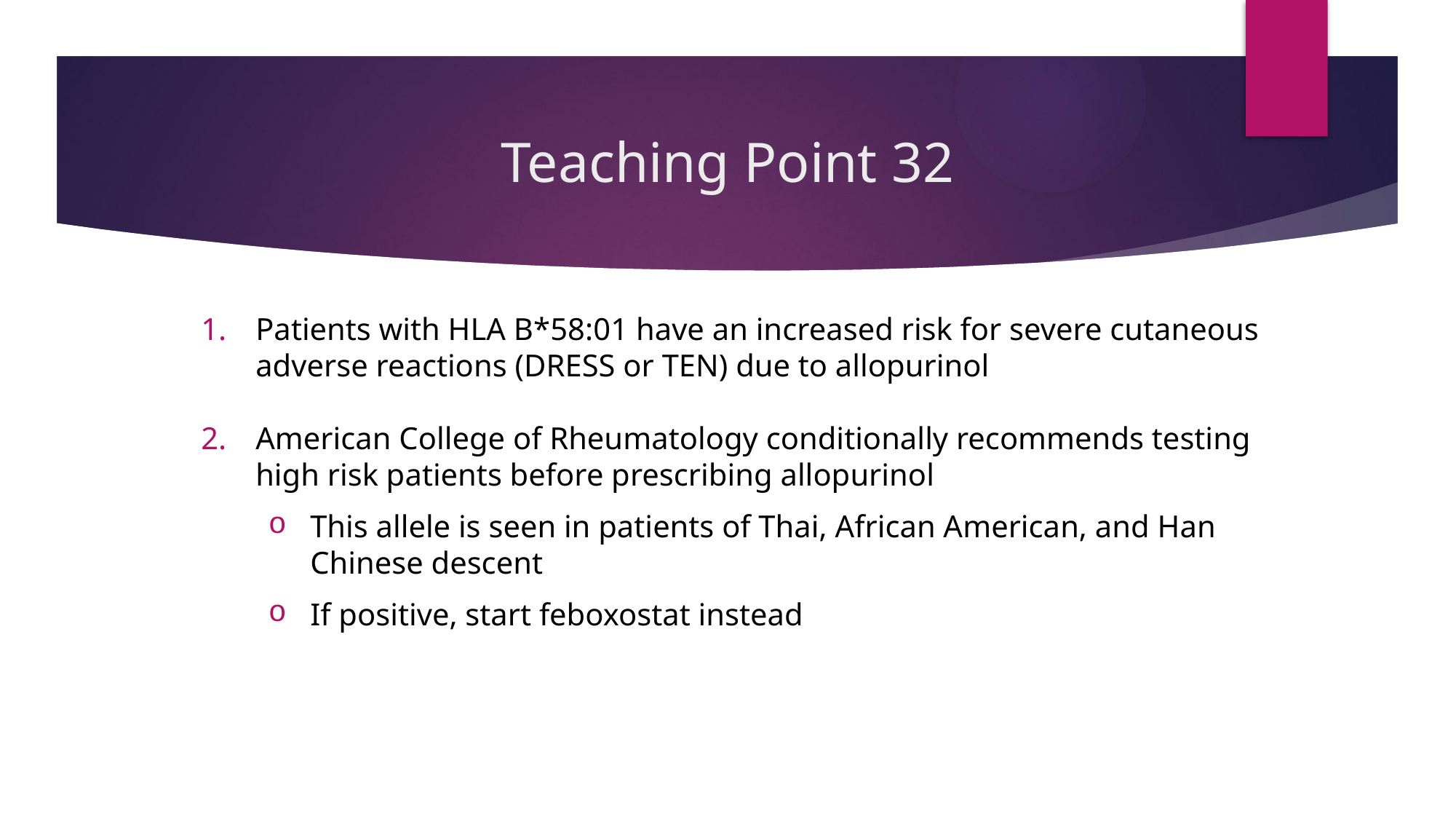

# Teaching Point 32
​
Patients with HLA B*58:01 have an increased risk for severe cutaneous adverse reactions (DRESS or TEN) due to allopurinol
American College of Rheumatology conditionally recommends testing high risk patients before prescribing allopurinol
This allele is seen in patients of Thai, African American, and Han Chinese descent
If positive, start feboxostat instead
