# Supplementary material for: Rheum4Games: A Game-Based Board Review to Enhance Confidence and Knowledge in Rheumatology for Internal Medicine Residents
Source: MedEdPORTAL. 2026 May 1;22:11597. doi: 10.15766/mep_2374-8265.11597 (PMC13133093; doi:10.15766/mep_2374-8265.11597)
Supplement: Supplementary file 1 — Question Bank - Easier.pptxQuestion Bank - Challenging.pptxSurvey.docxGame Rules.pptxBoard Game.docx [file mep_2374-8265.11597-s001.zip › B. Question Bank - Challenging.pptx]

## Slide 1
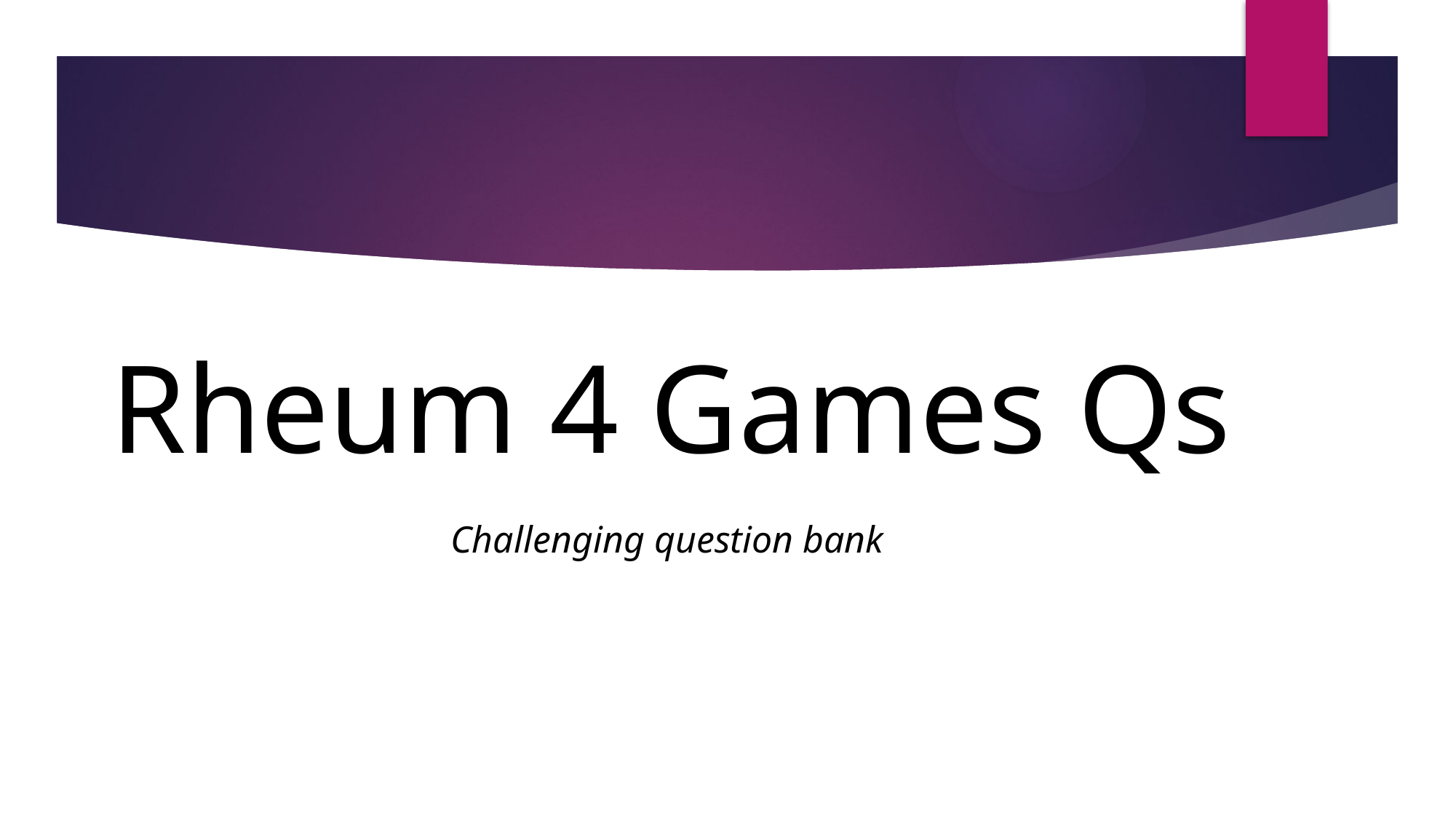

# Rheum 4 Games Qs
Challenging question bank

## Slide 2
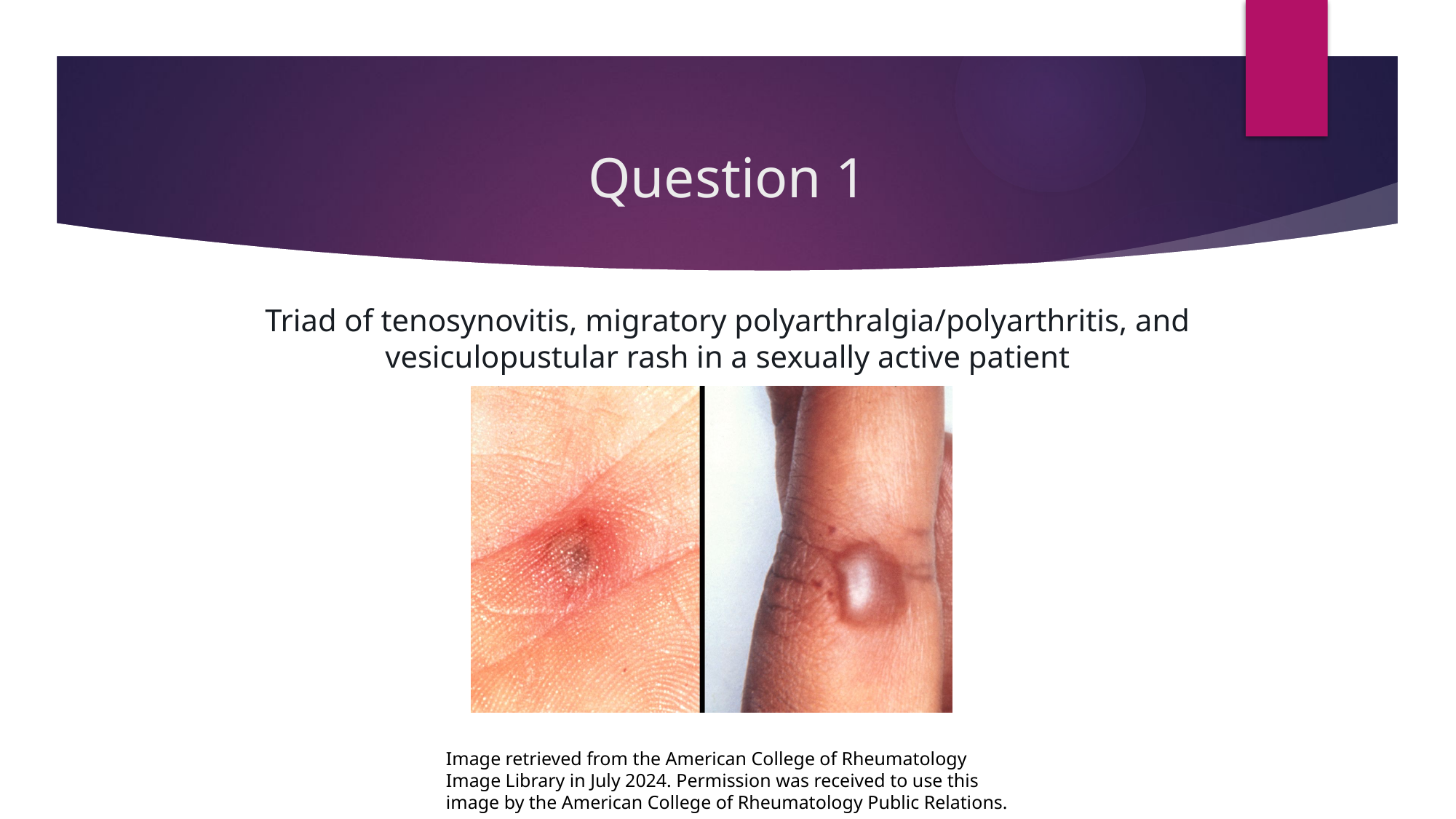

# Question 1
Triad of tenosynovitis, migratory polyarthralgia/polyarthritis, and vesiculopustular rash in a sexually active patient
Image retrieved from the American College of Rheumatology Image Library in July 2024. Permission was received to use this image by the American College of Rheumatology Public Relations.

## Slide 3
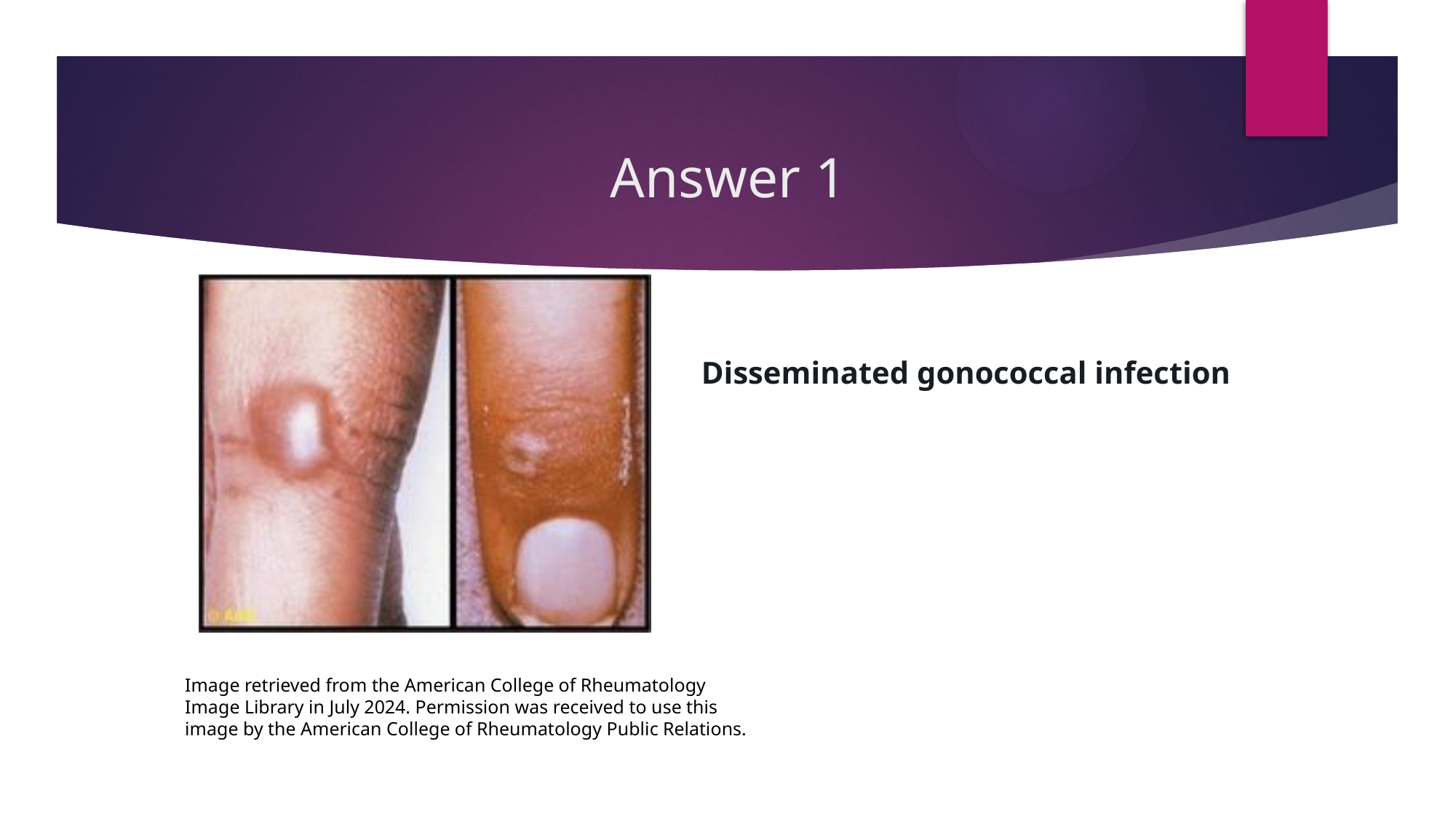

# Answer 1
Disseminated gonococcal infection
Image retrieved from the American College of Rheumatology Image Library in July 2024. Permission was received to use this image by the American College of Rheumatology Public Relations.

## Slide 4
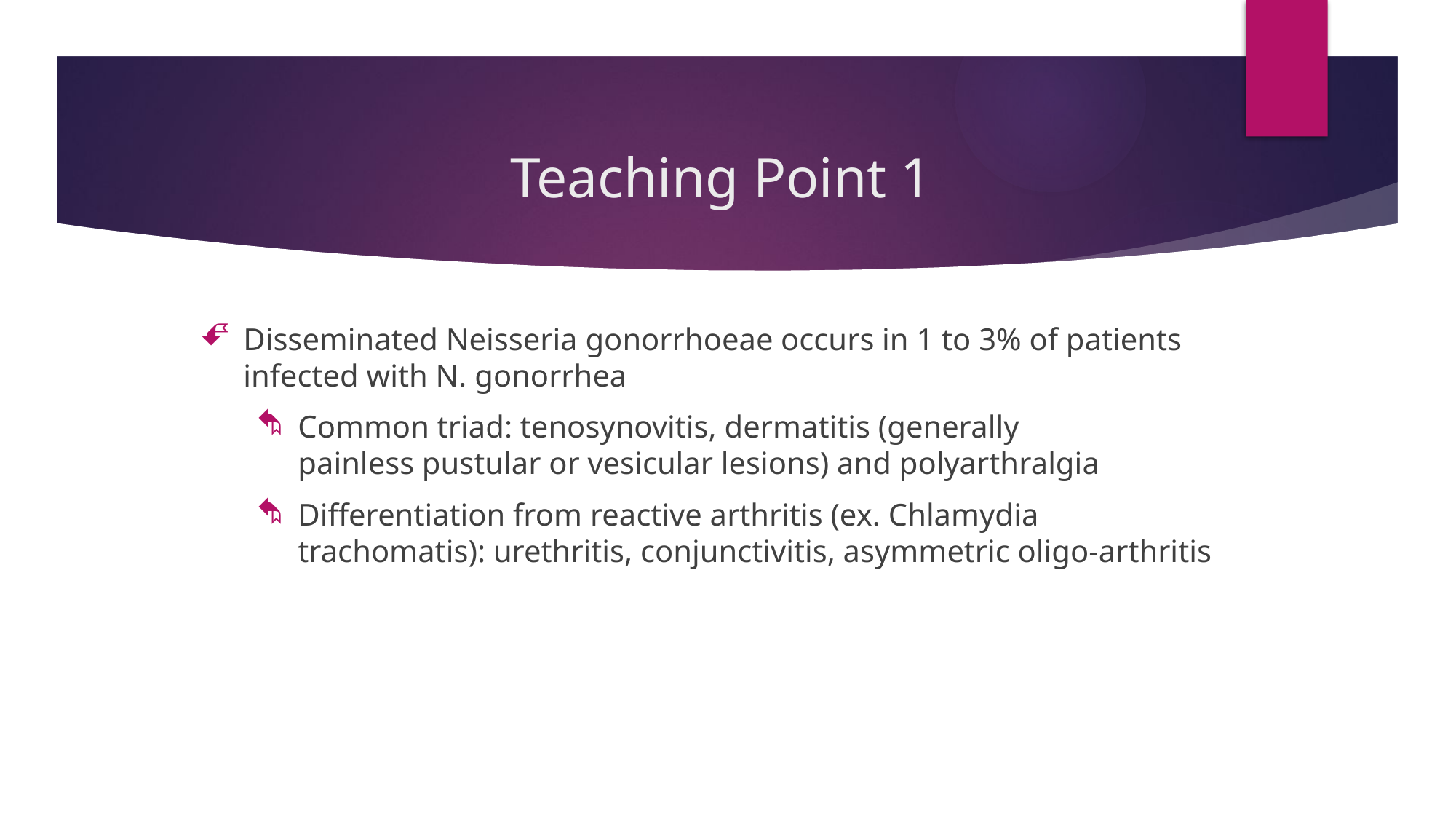

# Teaching Point 1
Disseminated Neisseria gonorrhoeae occurs in 1 to 3% of patients infected with N. gonorrhea
Common triad: tenosynovitis, dermatitis (generally painless pustular or vesicular lesions) and polyarthralgia
Differentiation from reactive arthritis (ex. Chlamydia trachomatis): urethritis, conjunctivitis, asymmetric oligo-arthritis

## Slide 5
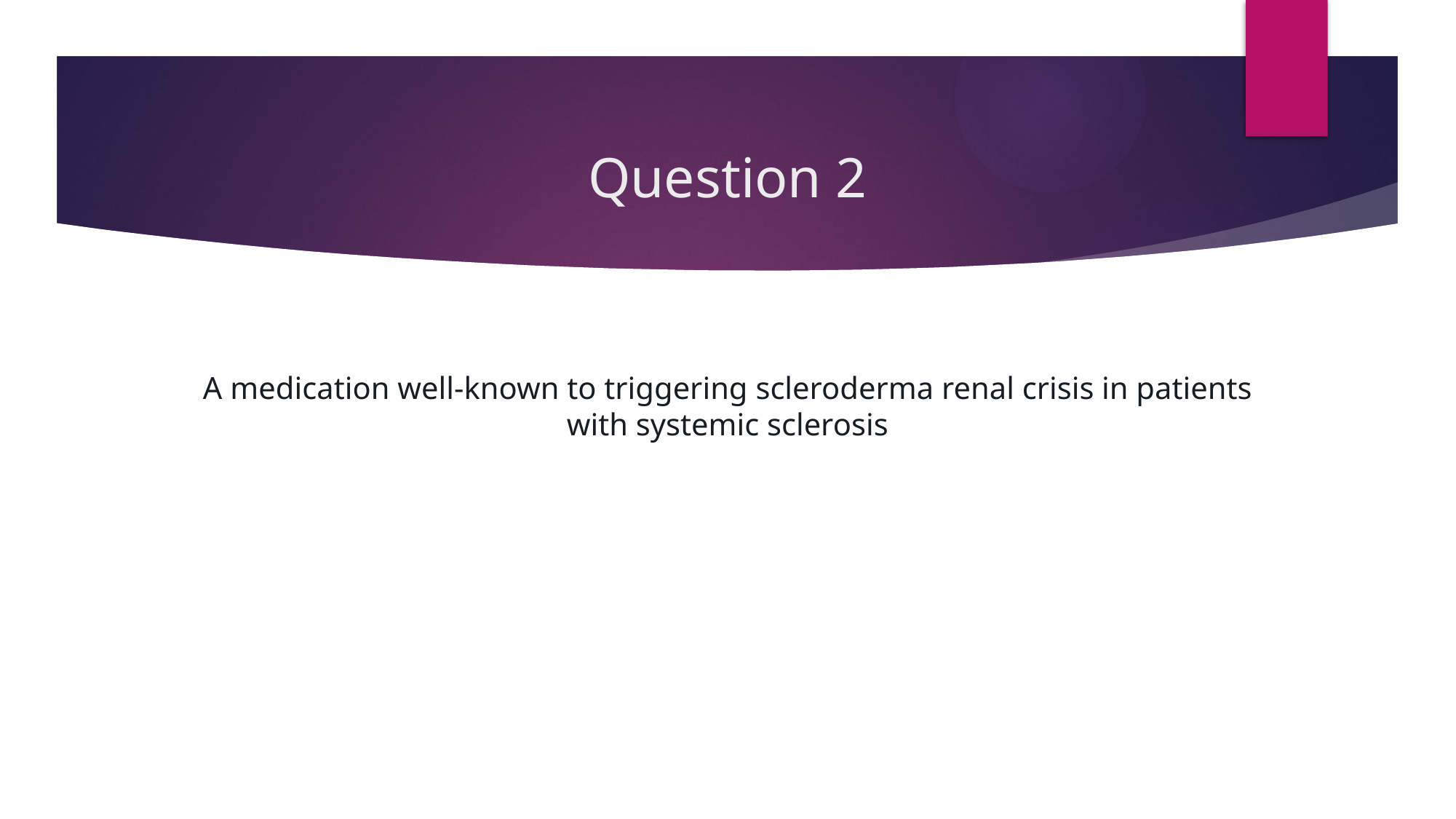

# Question 2
A medication well-known to triggering scleroderma renal crisis in patients with systemic sclerosis

## Slide 6
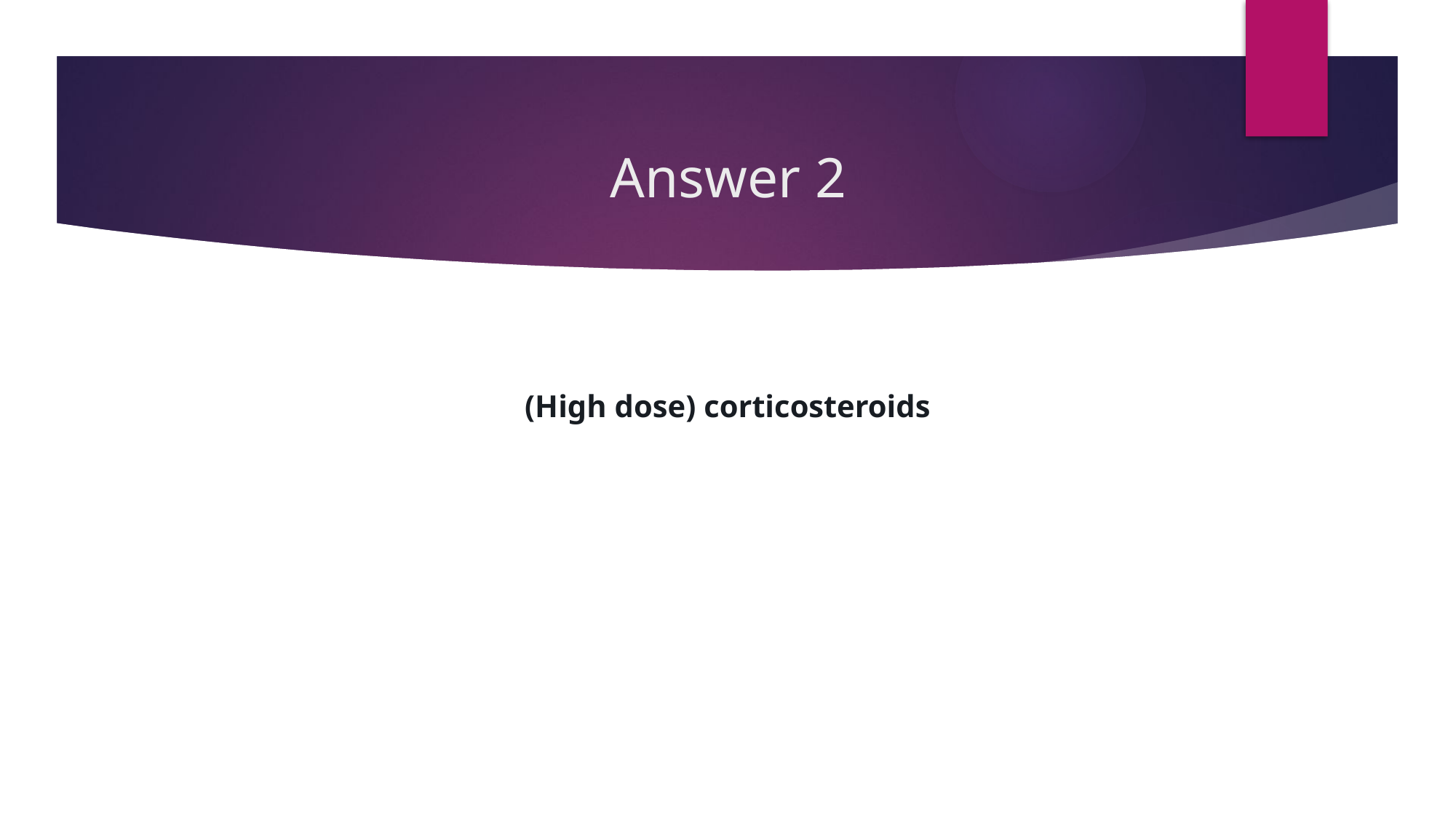

# Answer 2
(High dose) corticosteroids

## Slide 7
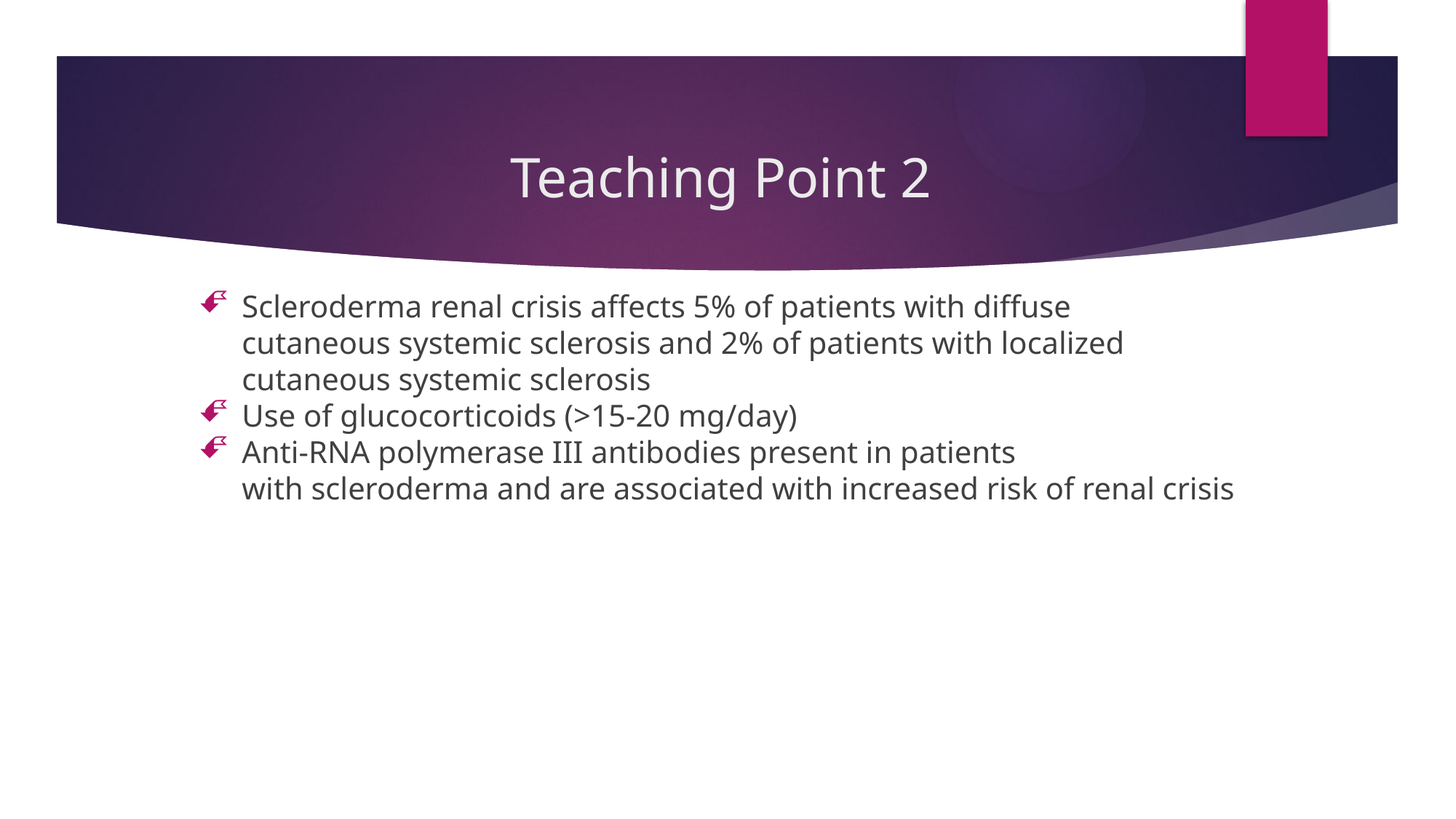

# Teaching Point 2
Scleroderma renal crisis affects 5% of patients with diffuse cutaneous systemic sclerosis and 2% of patients with localized cutaneous systemic sclerosis
Use of glucocorticoids (>15-20 mg/day)
Anti-RNA polymerase III antibodies present in patients with scleroderma and are associated with increased risk of renal crisis

## Slide 8
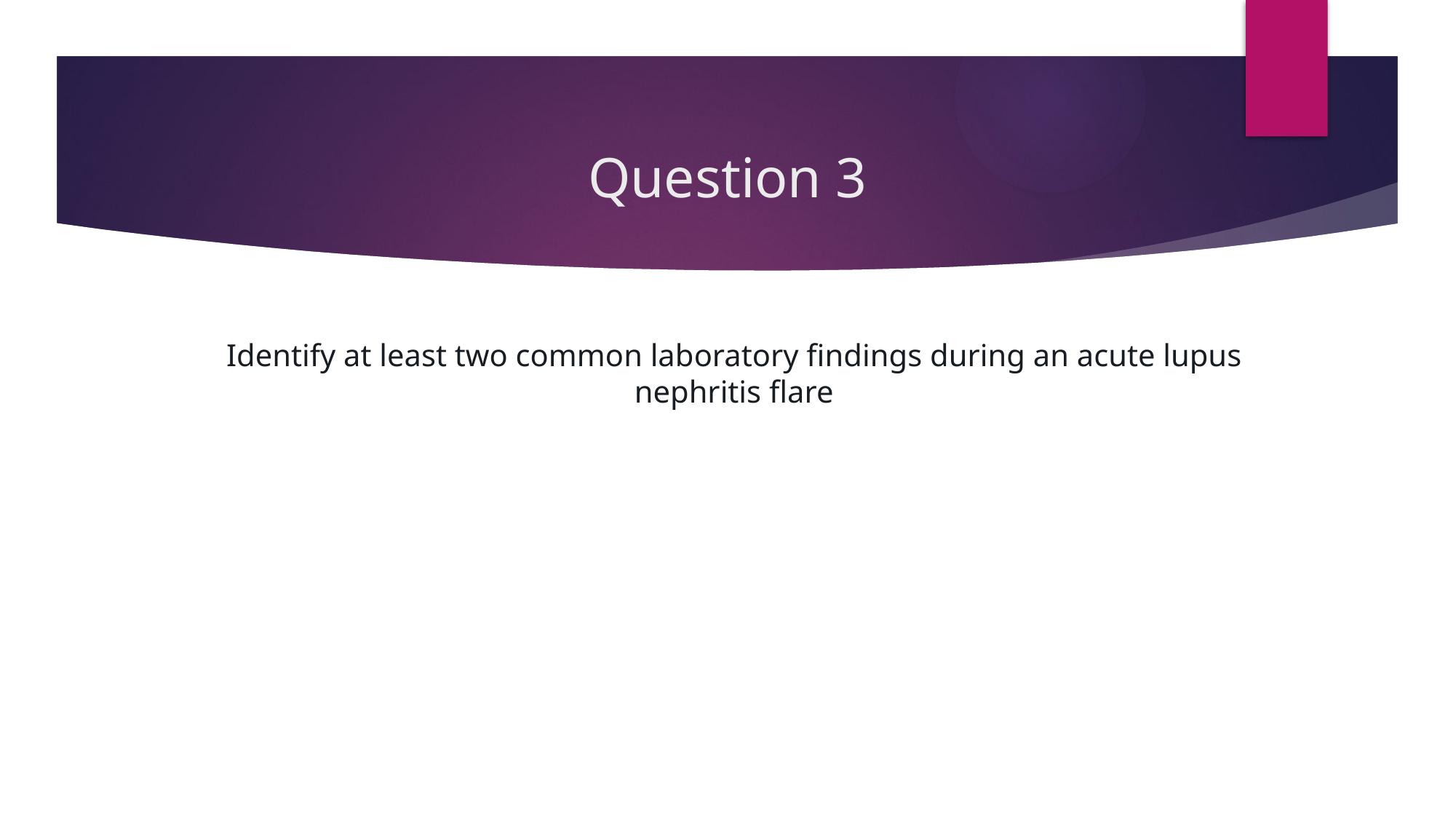

# Question 3
Identify at least two common laboratory findings during an acute lupus nephritis flare

## Slide 9
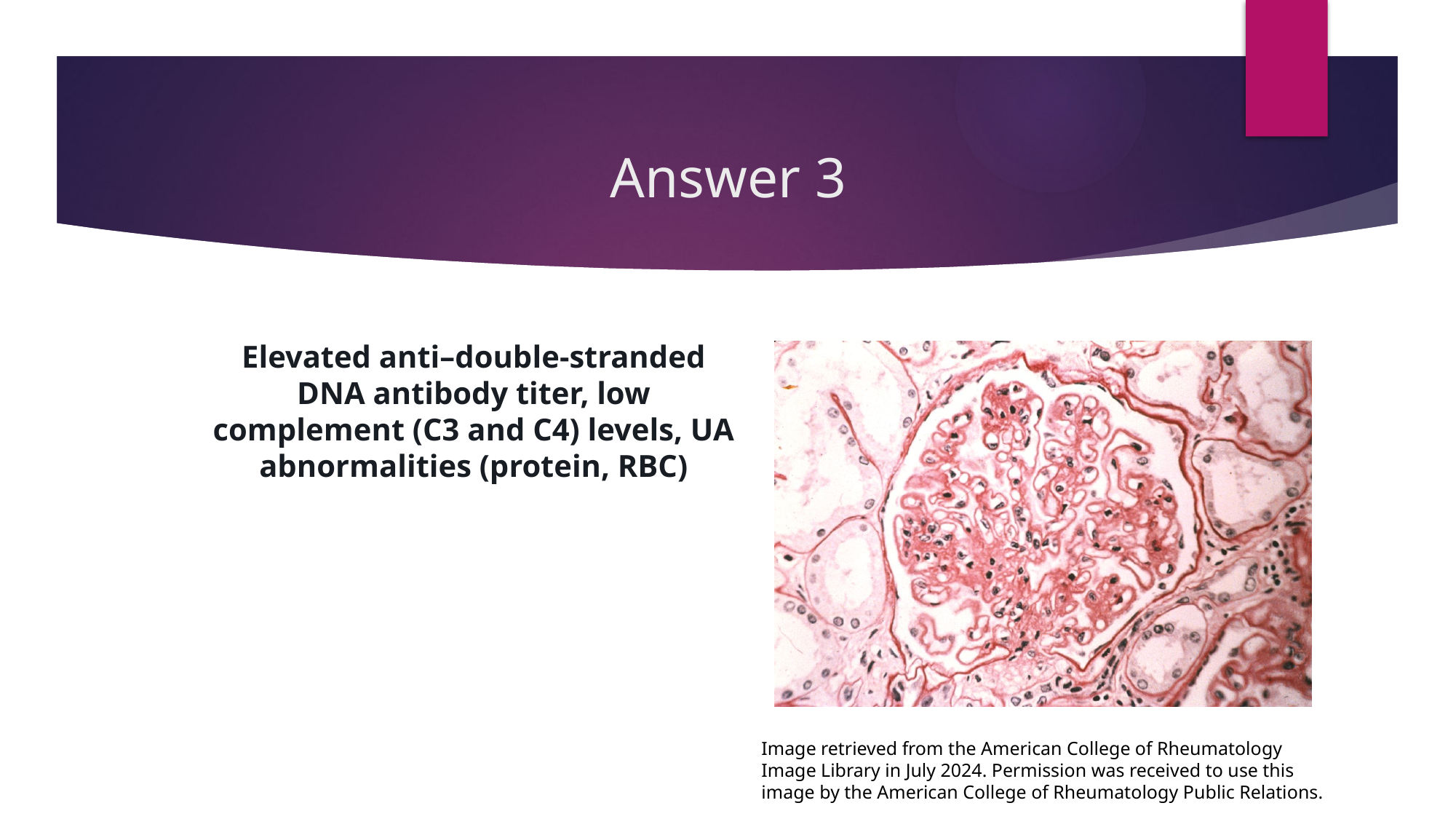

# Answer 3
Elevated anti–double-stranded DNA antibody titer, low complement (C3 and C4) levels, UA abnormalities (protein, RBC)
Image retrieved from the American College of Rheumatology Image Library in July 2024. Permission was received to use this image by the American College of Rheumatology Public Relations.

## Slide 10
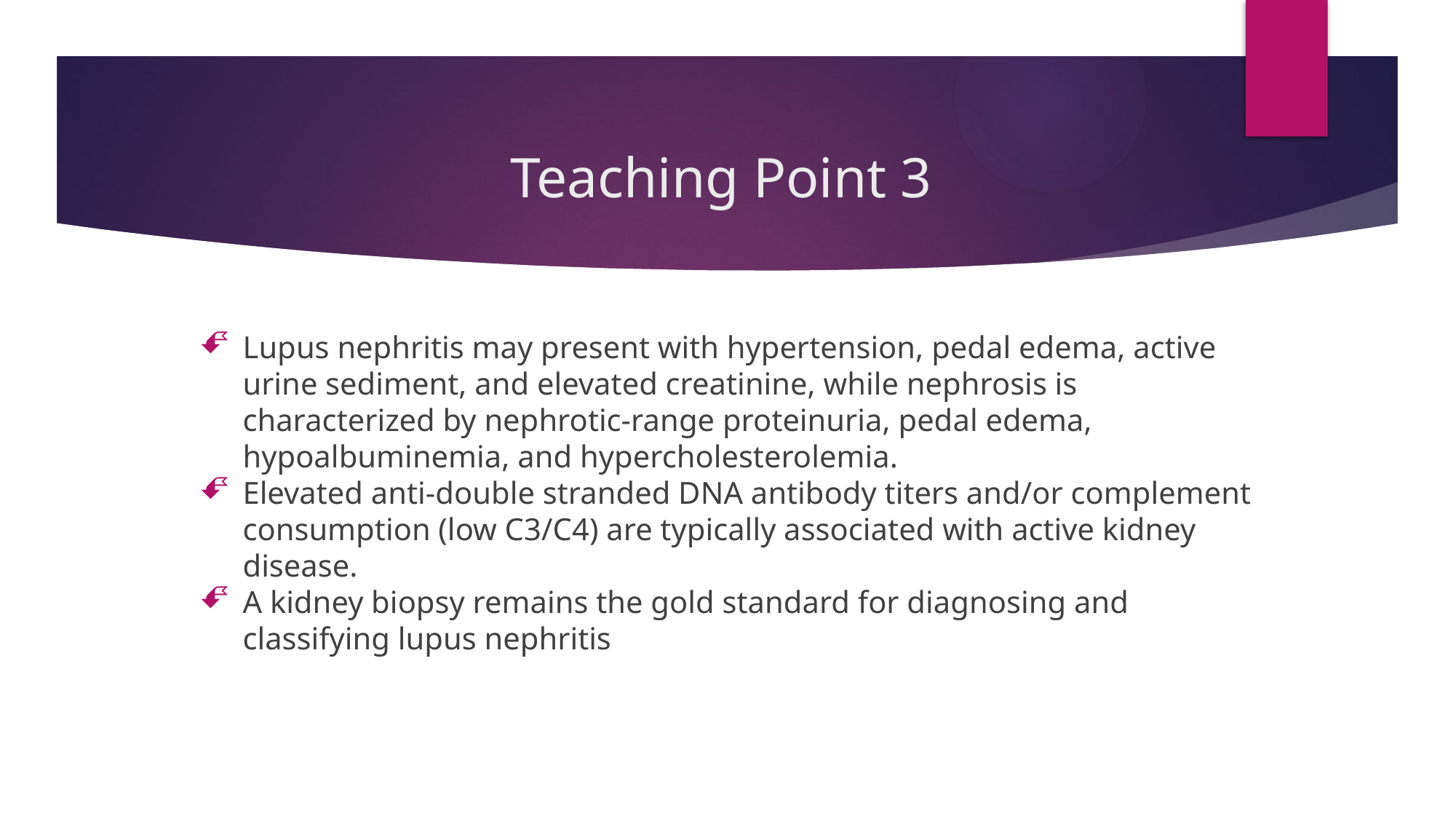

# Teaching Point 3
Lupus nephritis may present with hypertension, pedal edema, active urine sediment, and elevated creatinine, while nephrosis is characterized by nephrotic-range proteinuria, pedal edema, hypoalbuminemia, and hypercholesterolemia.
Elevated anti-double stranded DNA antibody titers and/or complement consumption (low C3/C4) are typically associated with active kidney disease.
A kidney biopsy remains the gold standard for diagnosing and classifying lupus nephritis

## Slide 11
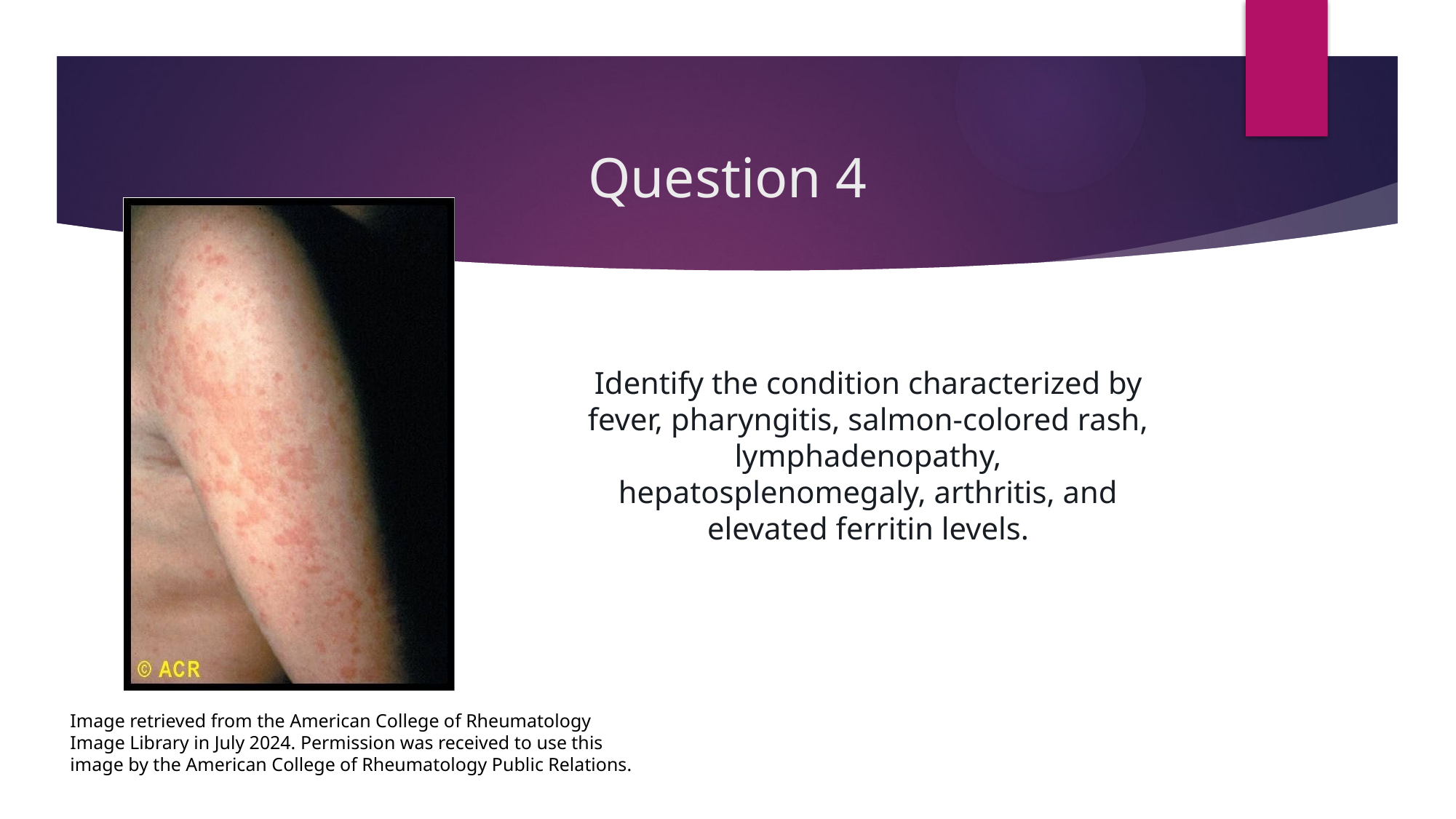

# Question 4
Identify the condition characterized by fever, pharyngitis, salmon-colored rash, lymphadenopathy, hepatosplenomegaly, arthritis, and elevated ferritin levels.
Image retrieved from the American College of Rheumatology Image Library in July 2024. Permission was received to use this image by the American College of Rheumatology Public Relations.

## Slide 12
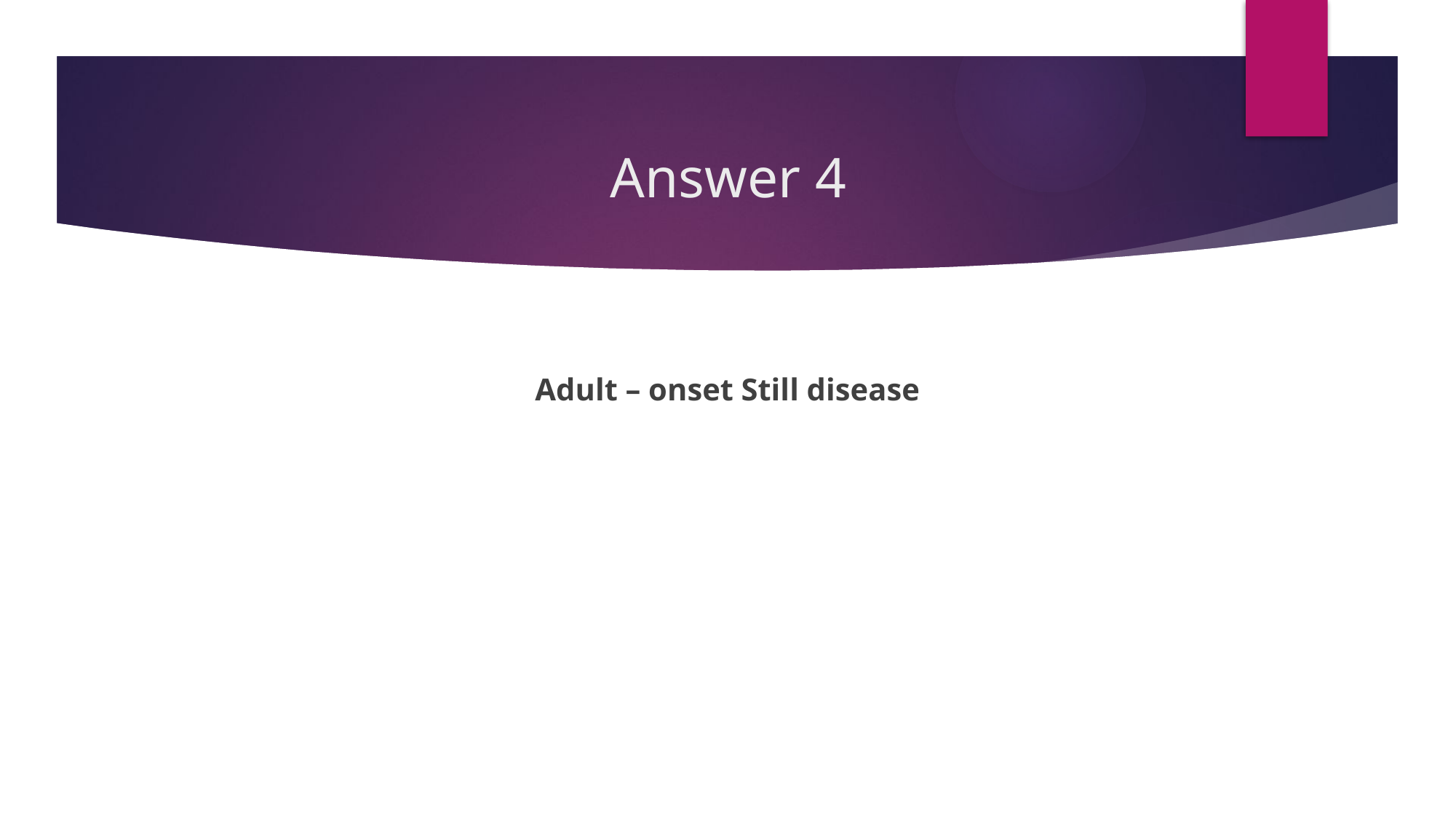

# Answer 4
Adult – onset Still disease

## Slide 13
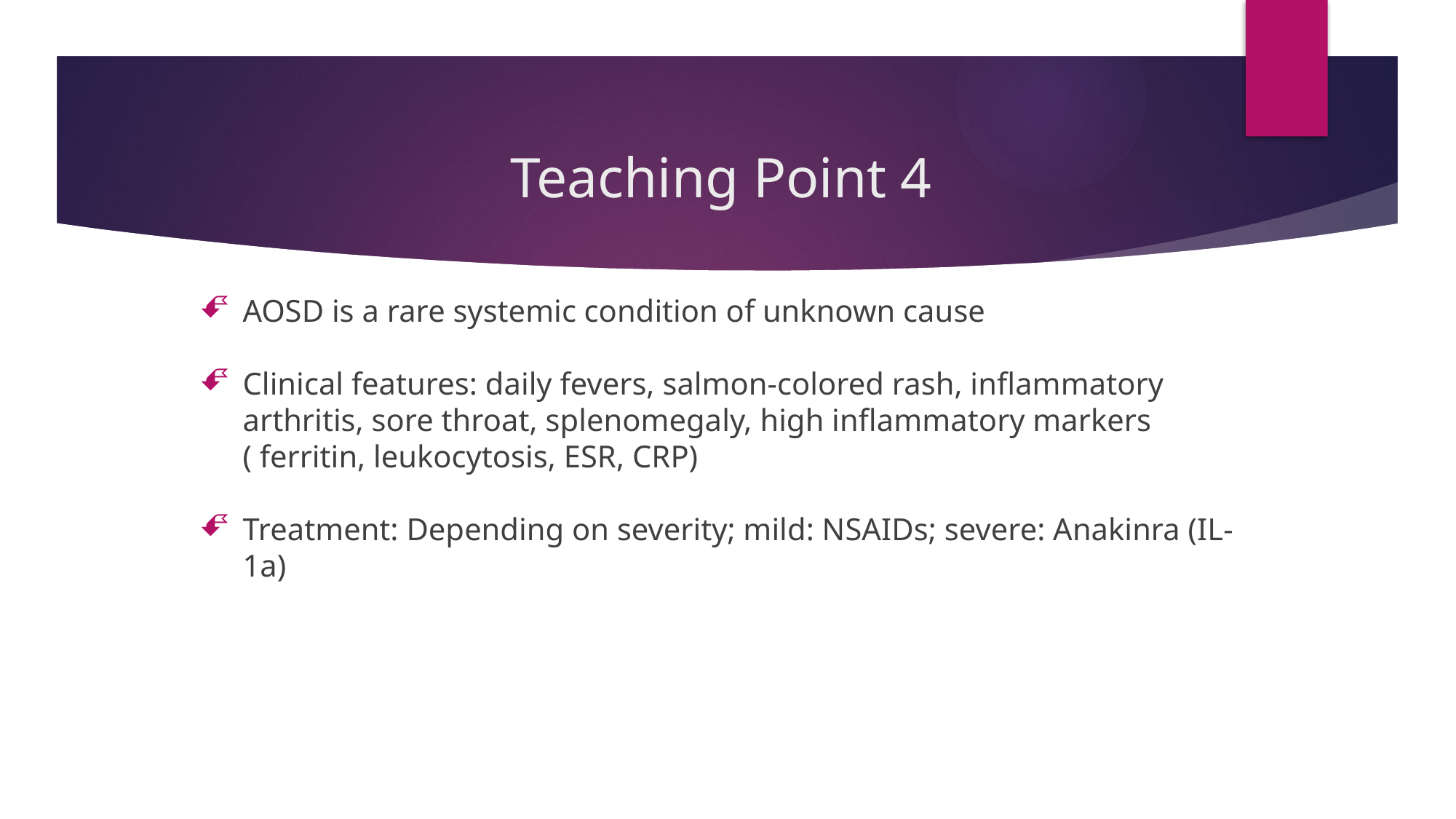

# Teaching Point 4
AOSD is a rare systemic condition of unknown cause
Clinical features: daily fevers, salmon-colored rash, inflammatory arthritis, sore throat, splenomegaly, high inflammatory markers ( ferritin, leukocytosis, ESR, CRP)
Treatment: Depending on severity; mild: NSAIDs; severe: Anakinra (IL-1a)

## Slide 14
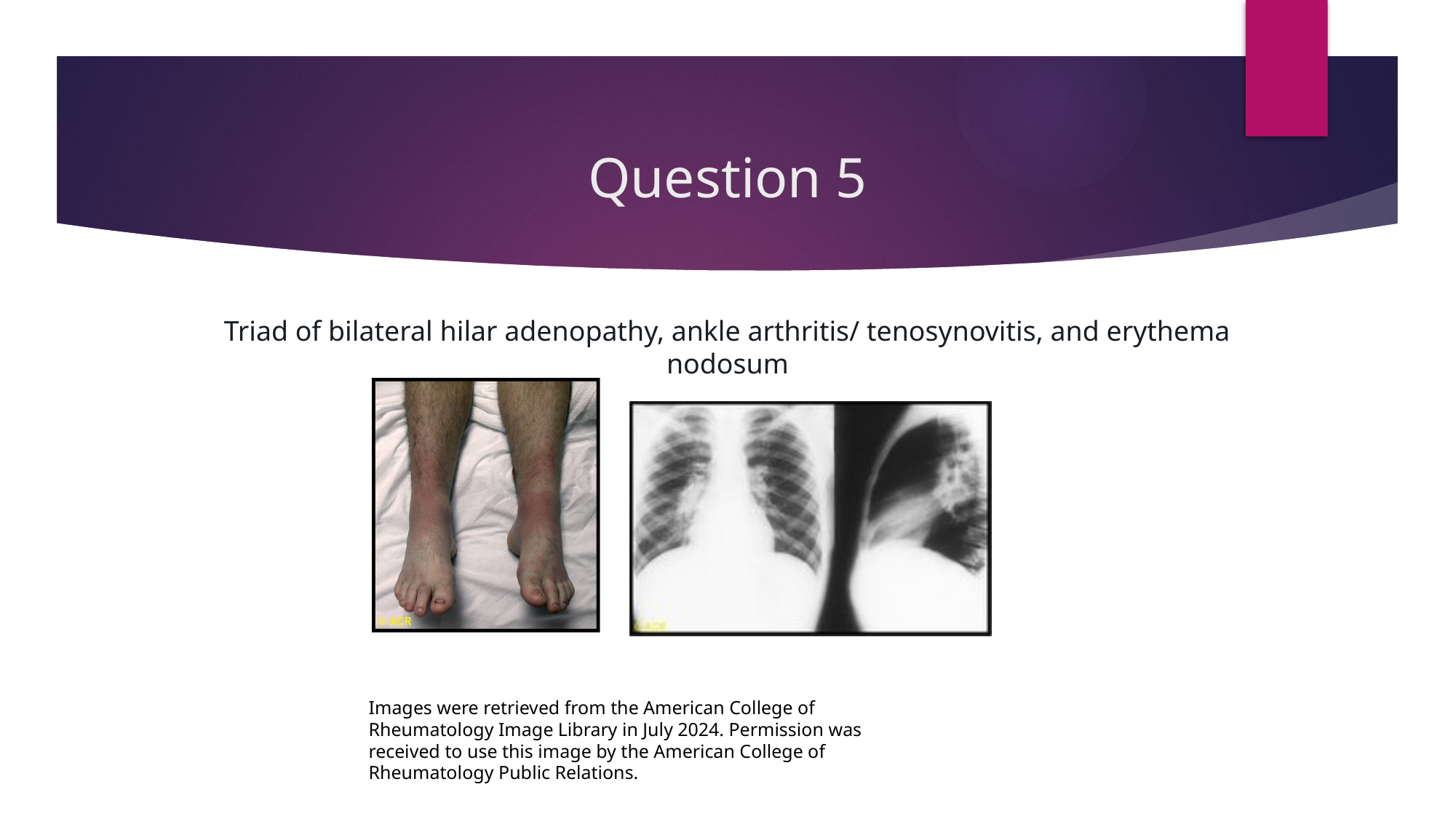

# Question 5
Triad of bilateral hilar adenopathy, ankle arthritis/ tenosynovitis, and erythema nodosum
Images were retrieved from the American College of Rheumatology Image Library in July 2024. Permission was received to use this image by the American College of Rheumatology Public Relations.

## Slide 15
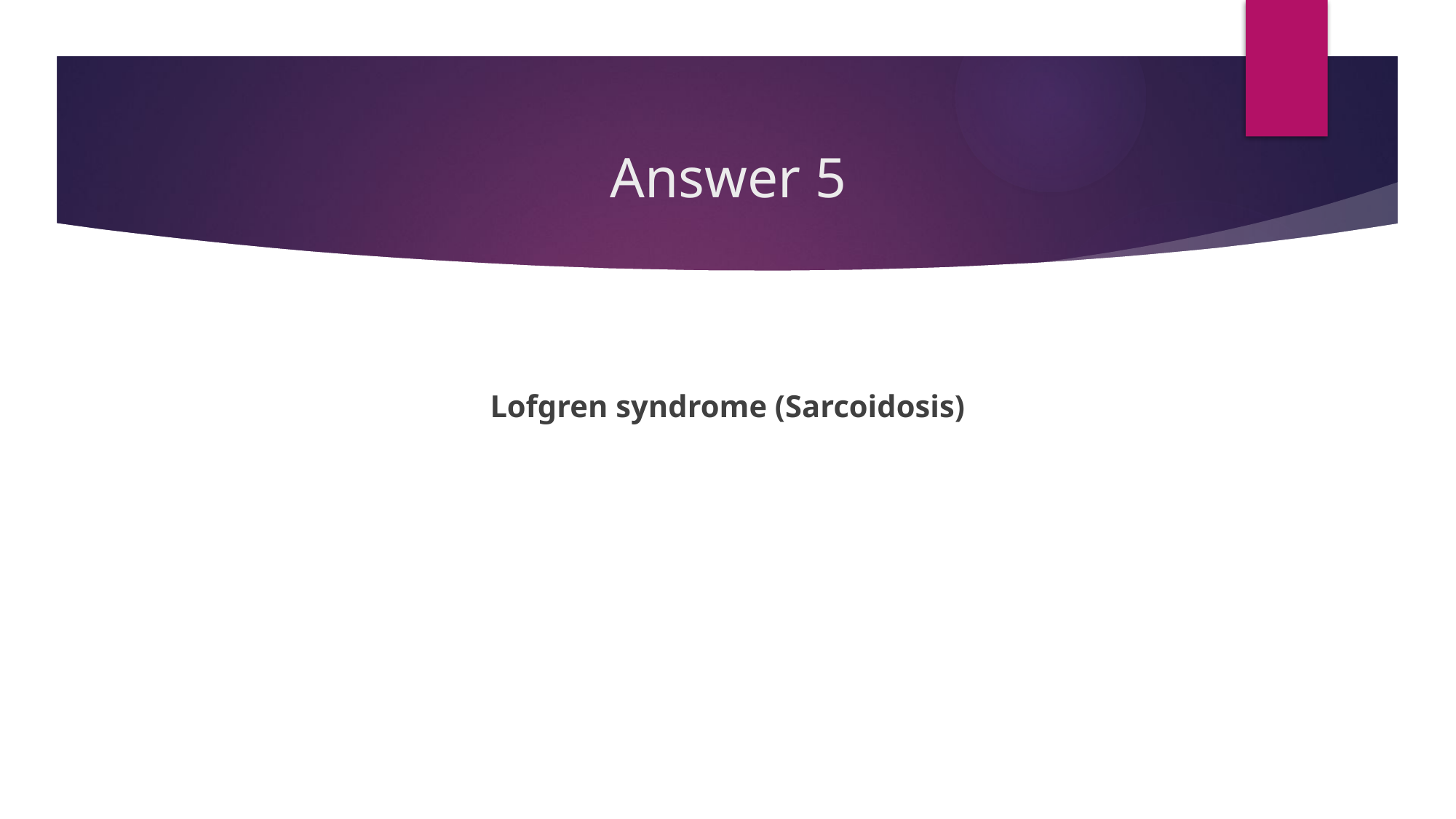

# Answer 5
Lofgren syndrome (Sarcoidosis)

## Slide 16
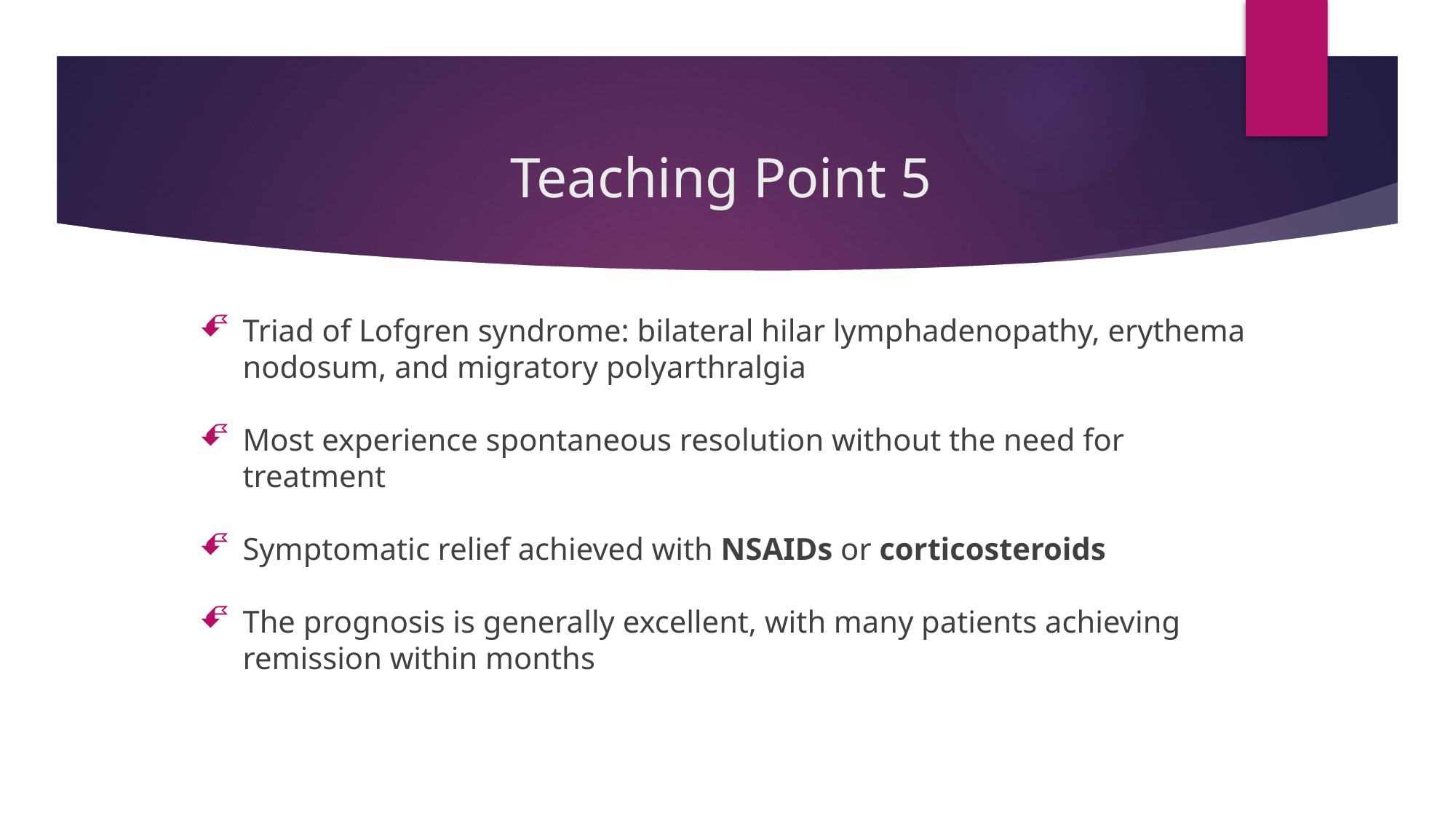

# Teaching Point 5
Triad of Lofgren syndrome: bilateral hilar lymphadenopathy, erythema nodosum, and migratory polyarthralgia
Most experience spontaneous resolution without the need for treatment
Symptomatic relief achieved with NSAIDs or corticosteroids
The prognosis is generally excellent, with many patients achieving remission within months

## Slide 17
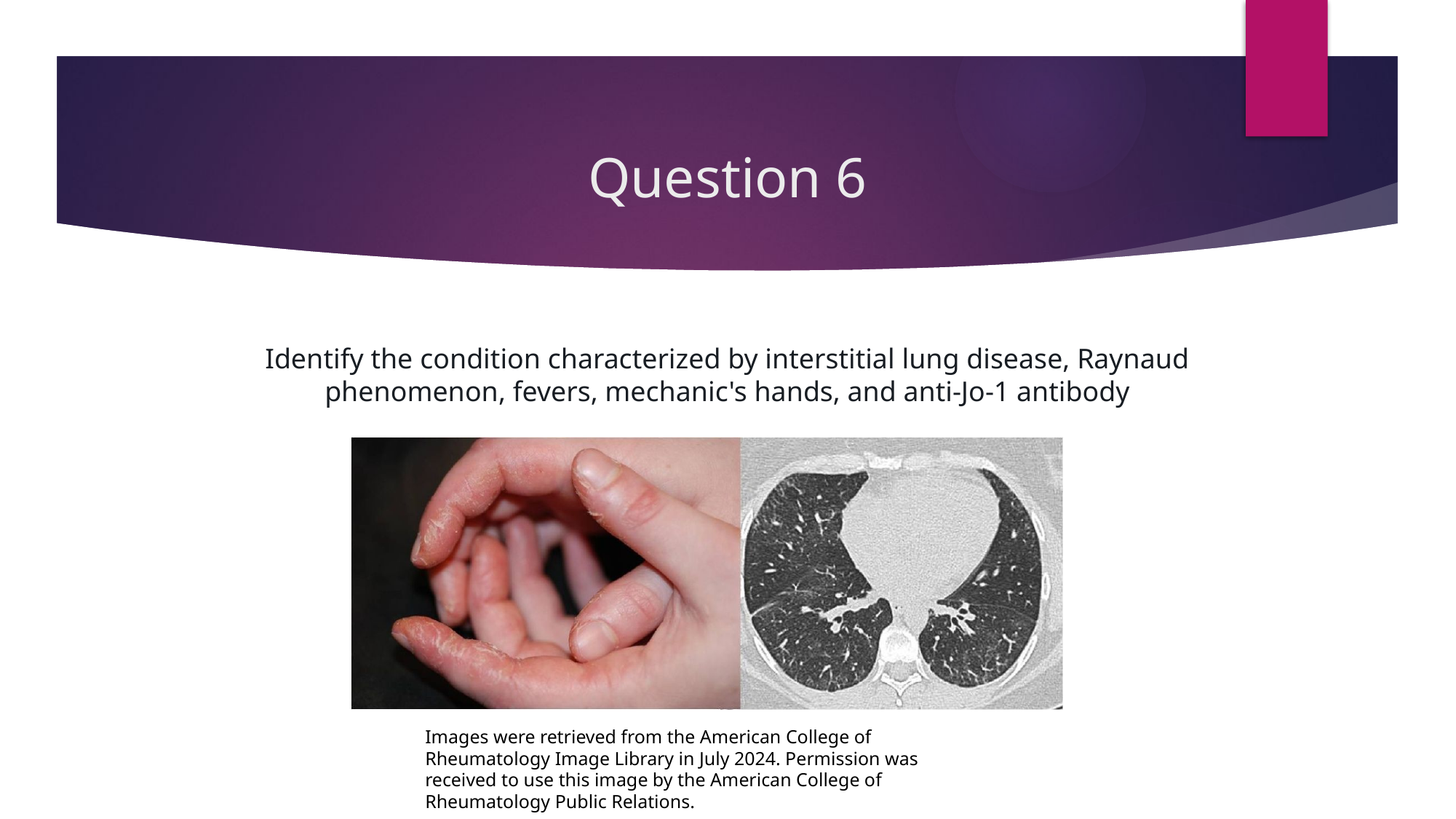

# Question 6
Identify the condition characterized by interstitial lung disease, Raynaud phenomenon, fevers, mechanic's hands, and anti-Jo-1 antibody
Images were retrieved from the American College of Rheumatology Image Library in July 2024. Permission was received to use this image by the American College of Rheumatology Public Relations.

## Slide 18
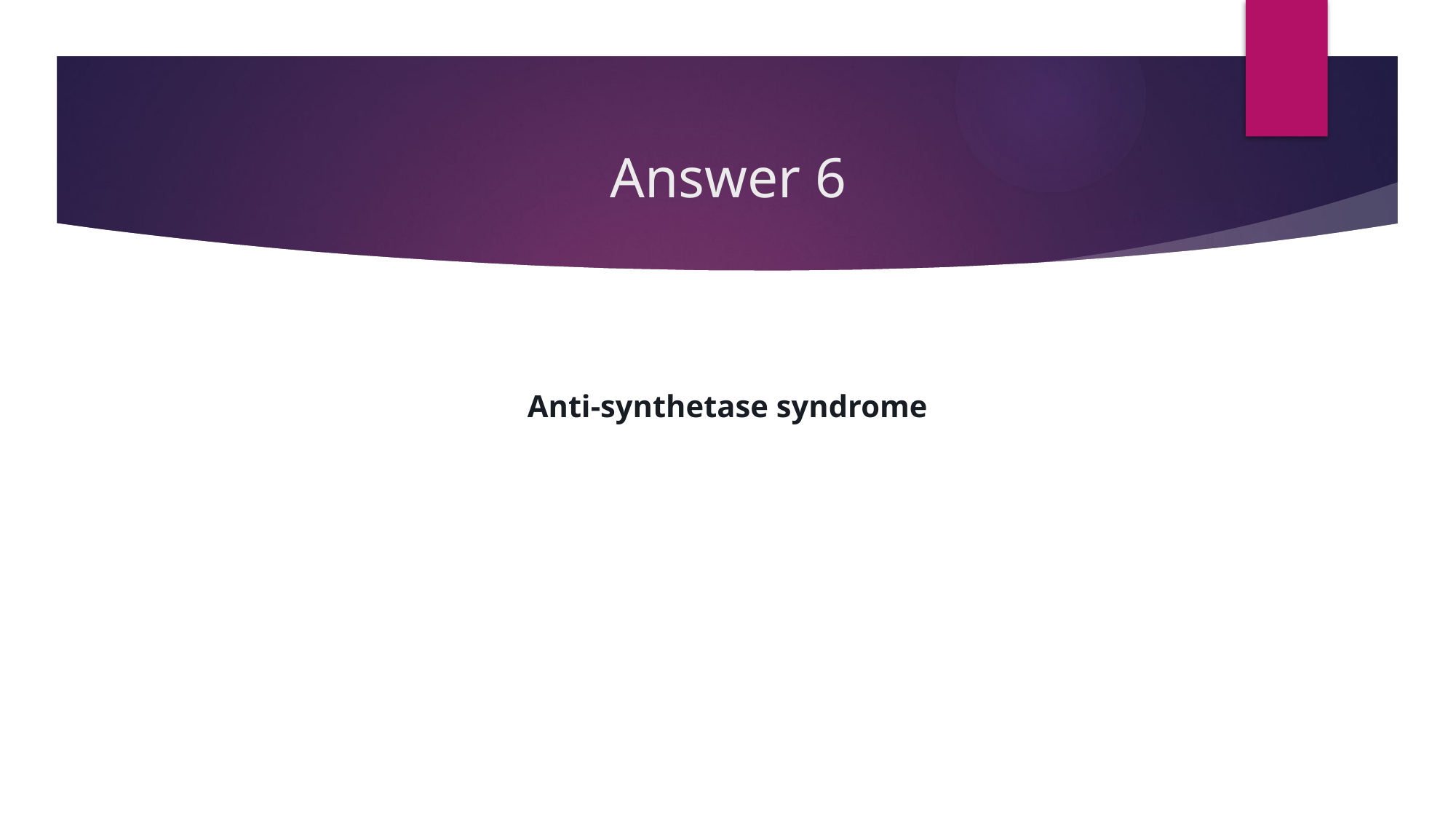

# Answer 6
Anti-synthetase syndrome

## Slide 19
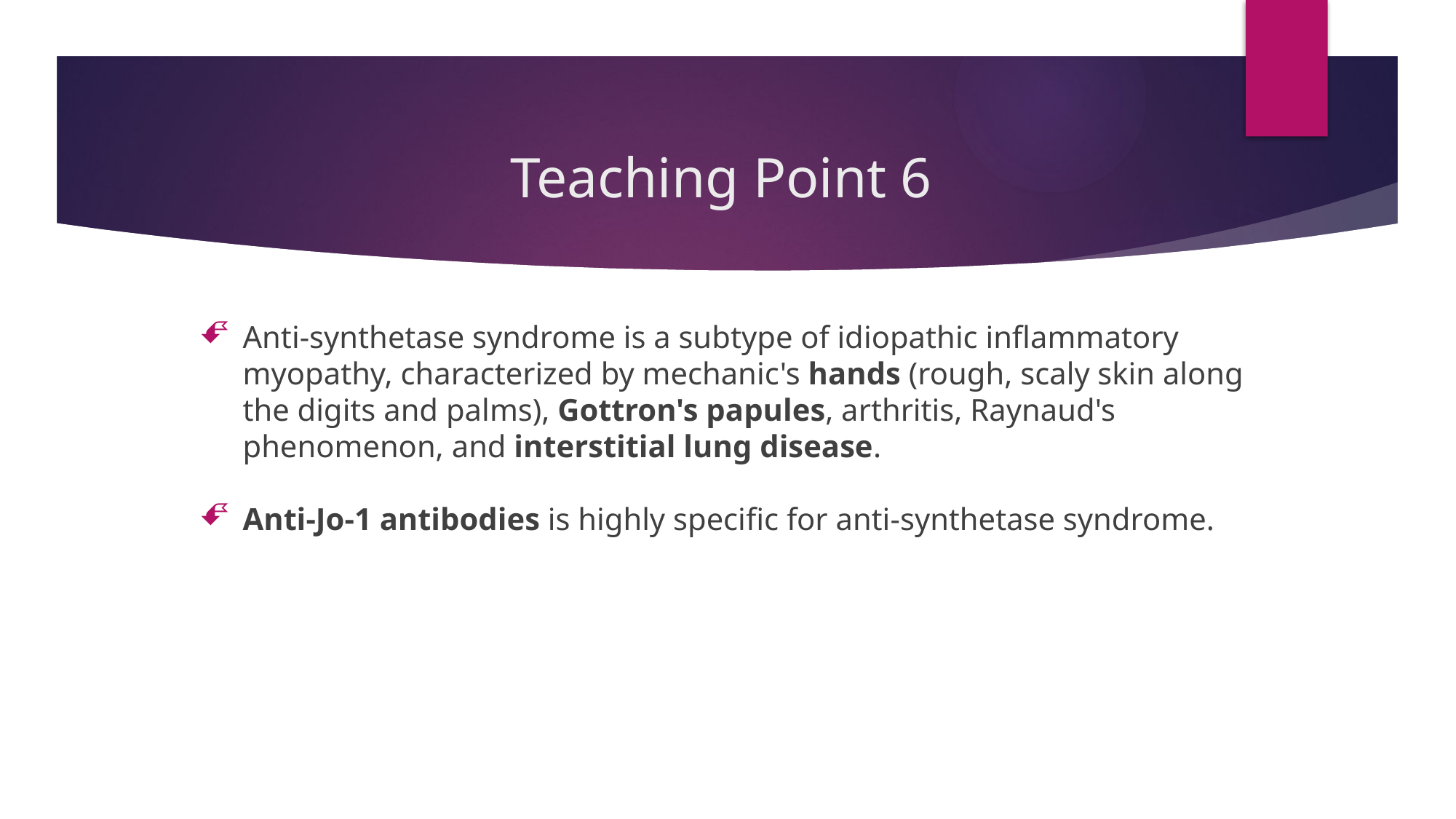

# Teaching Point 6
Anti-synthetase syndrome is a subtype of idiopathic inflammatory myopathy, characterized by mechanic's hands (rough, scaly skin along the digits and palms), Gottron's papules, arthritis, Raynaud's phenomenon, and interstitial lung disease.
Anti-Jo-1 antibodies is highly specific for anti-synthetase syndrome.

## Slide 20
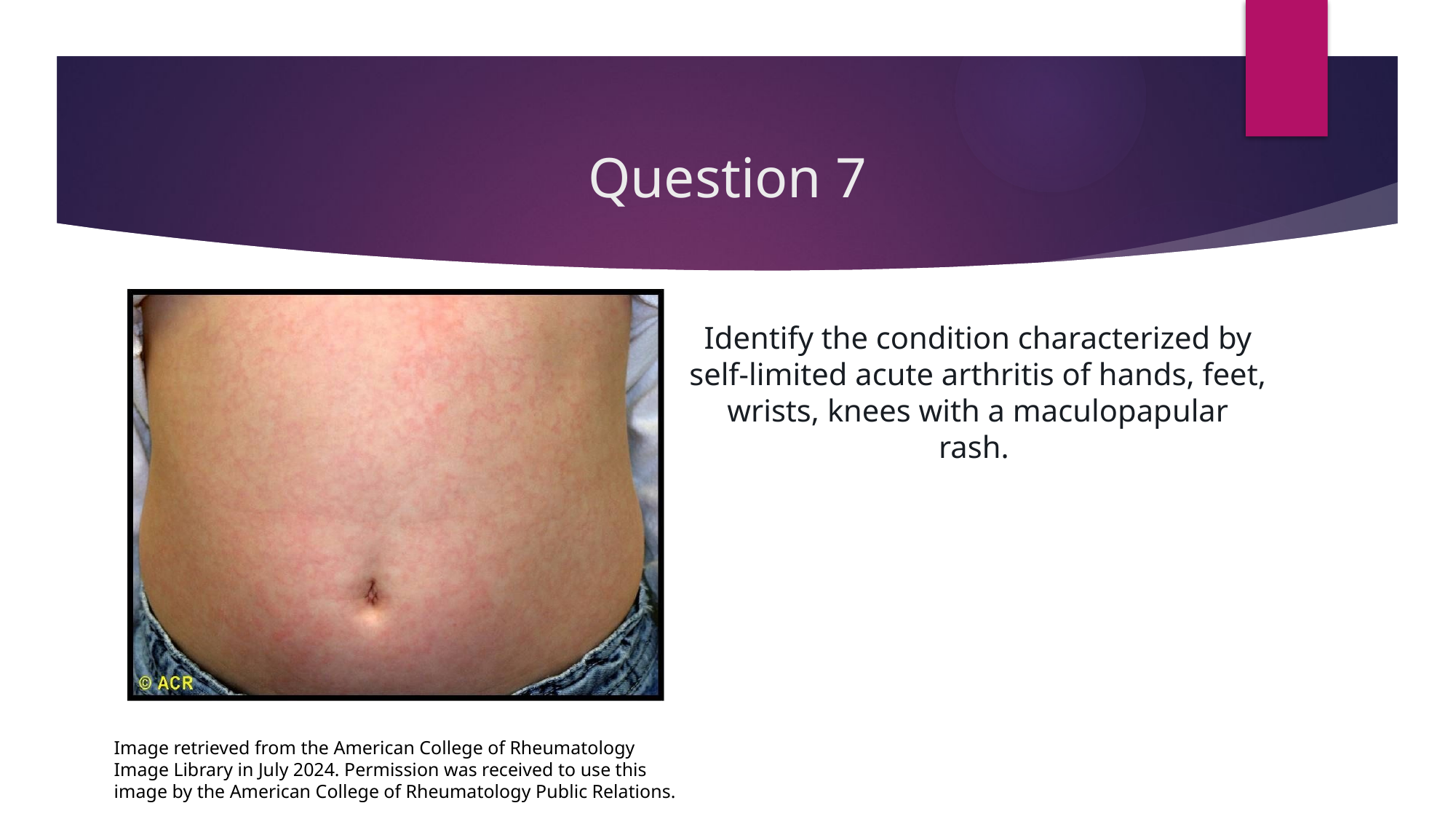

# Question 7
Identify the condition characterized by self-limited acute arthritis of hands, feet, wrists, knees with a maculopapular rash.
Image retrieved from the American College of Rheumatology Image Library in July 2024. Permission was received to use this image by the American College of Rheumatology Public Relations.

## Slide 21
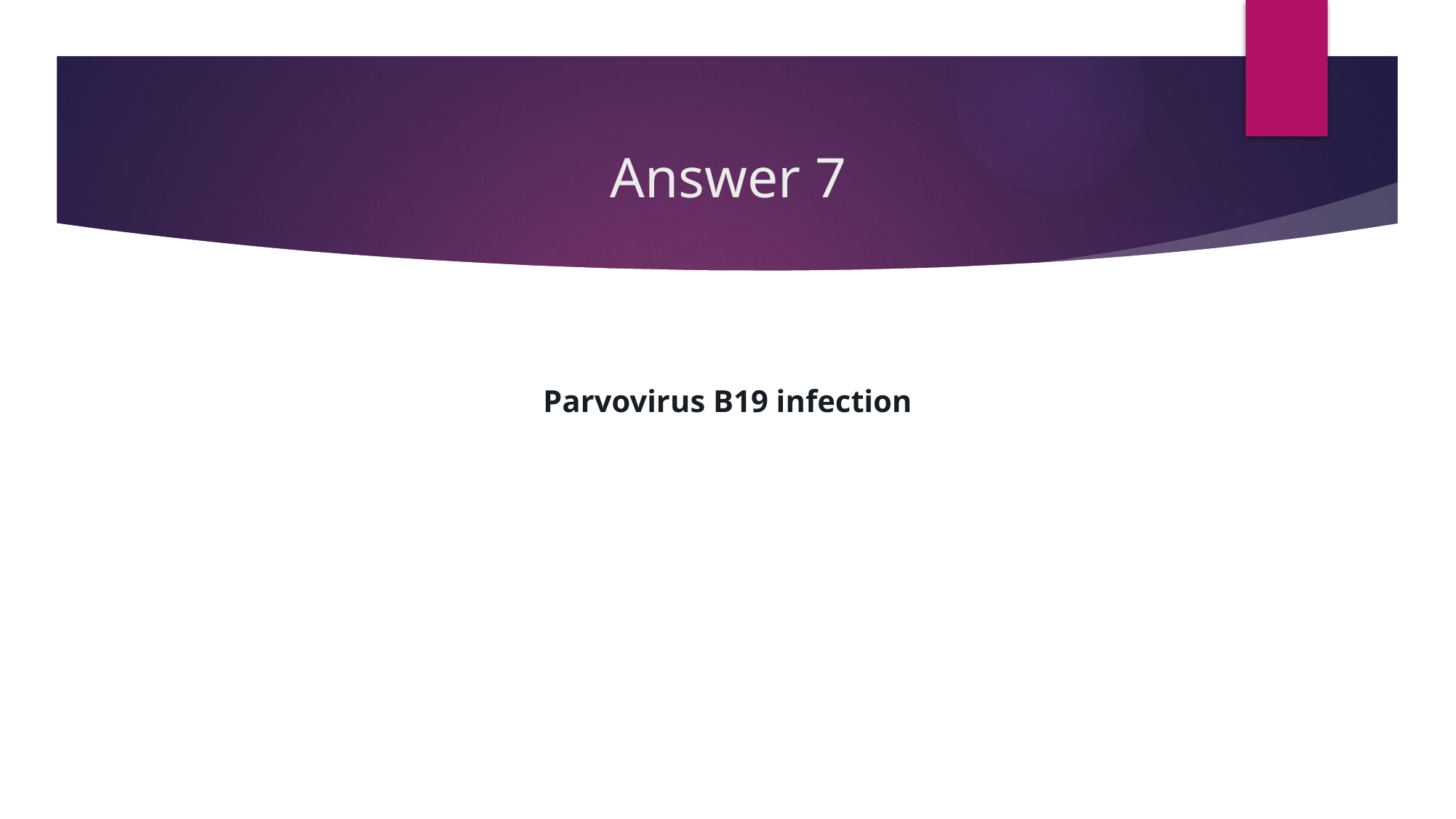

# Answer 7
Parvovirus B19 infection

## Slide 22
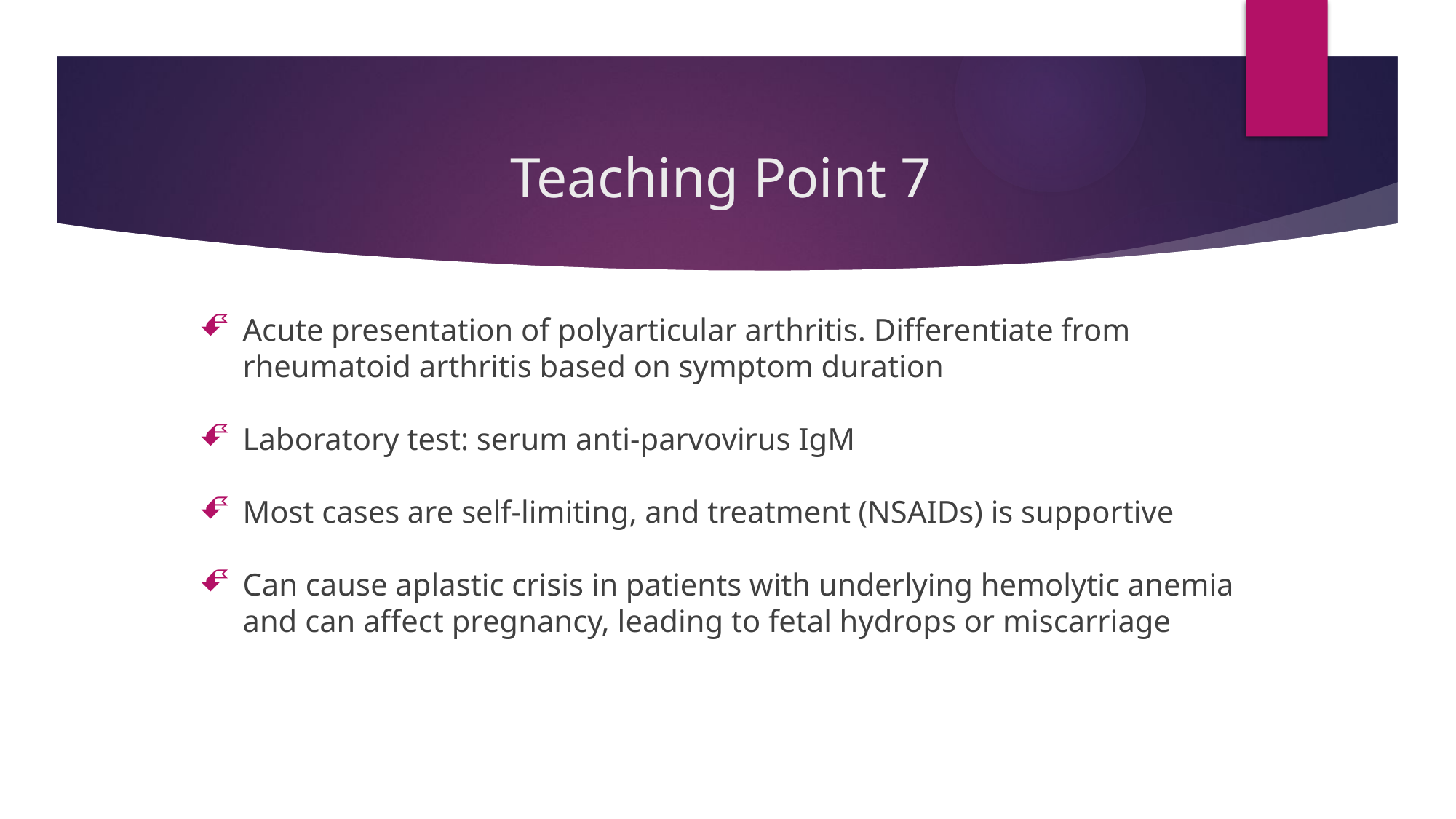

# Teaching Point 7
Acute presentation of polyarticular arthritis. Differentiate from rheumatoid arthritis based on symptom duration
Laboratory test: serum anti-parvovirus IgM
Most cases are self-limiting, and treatment (NSAIDs) is supportive
Can cause aplastic crisis in patients with underlying hemolytic anemia and can affect pregnancy, leading to fetal hydrops or miscarriage

## Slide 23
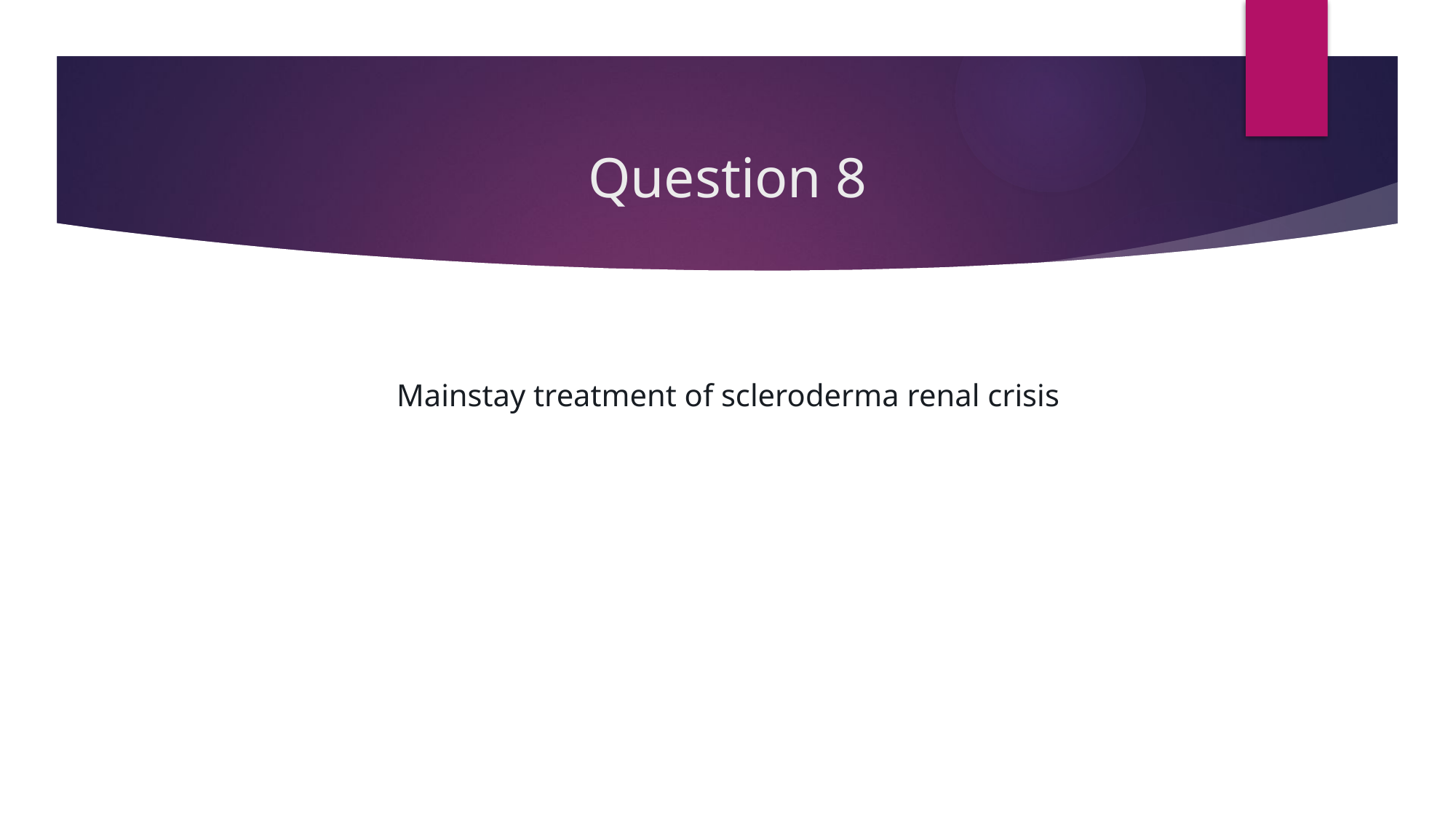

# Question 8
Mainstay treatment of scleroderma renal crisis

## Slide 24
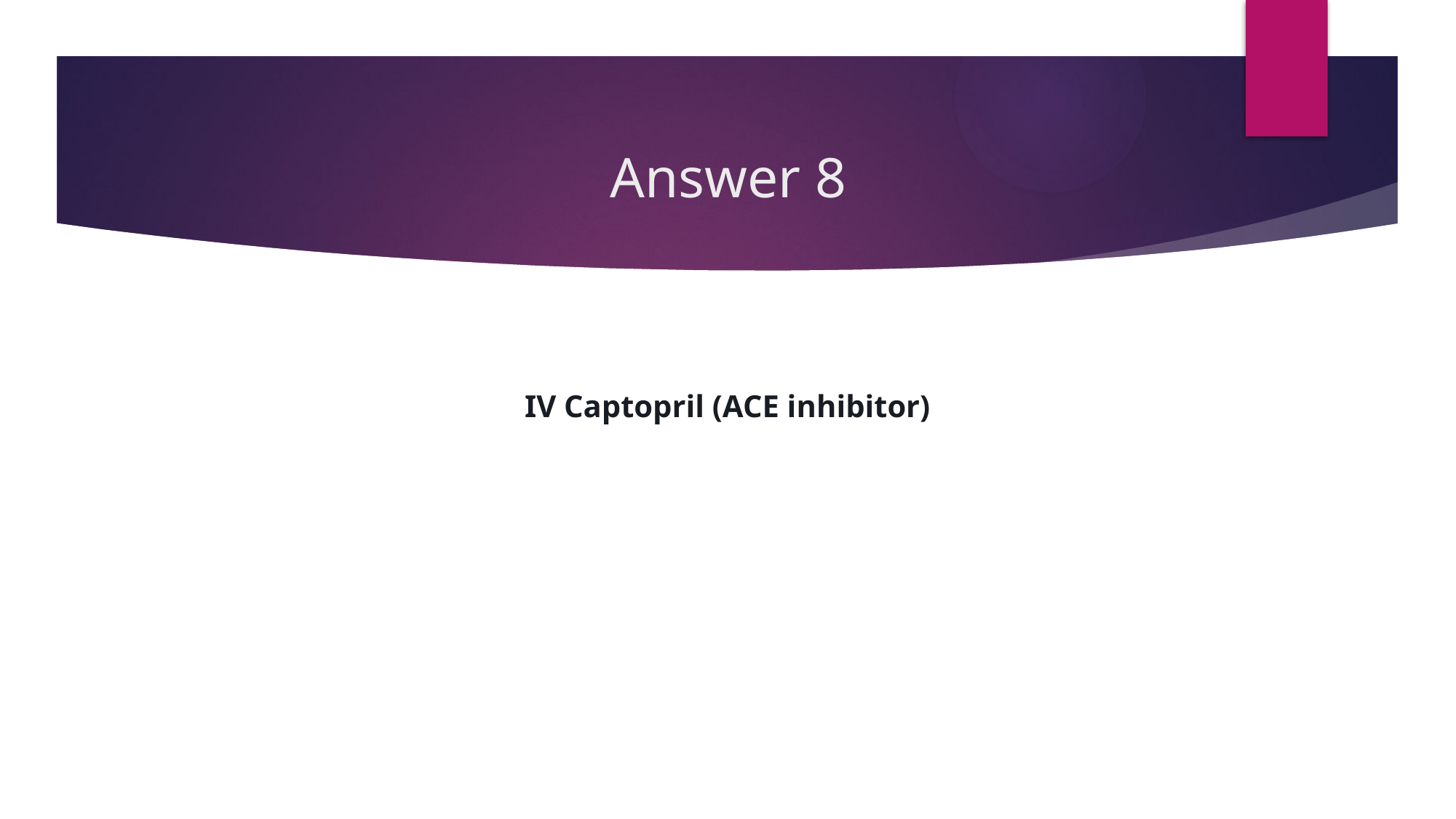

# Answer 8
IV Captopril (ACE inhibitor)

## Slide 25
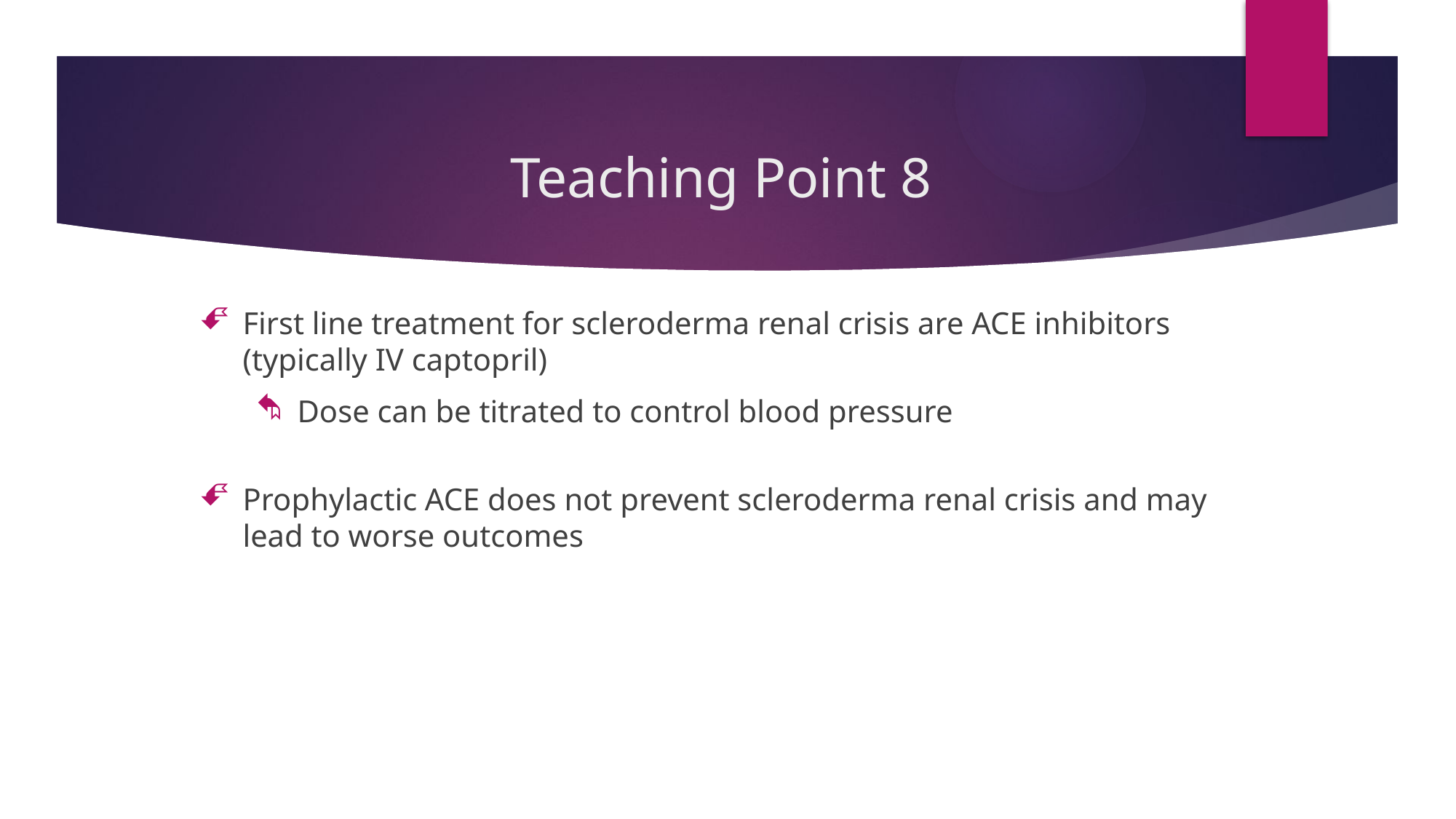

# Teaching Point 8
First line treatment for scleroderma renal crisis are ACE inhibitors (typically IV captopril)
Dose can be titrated to control blood pressure
Prophylactic ACE does not prevent scleroderma renal crisis and may lead to worse outcomes

## Slide 26
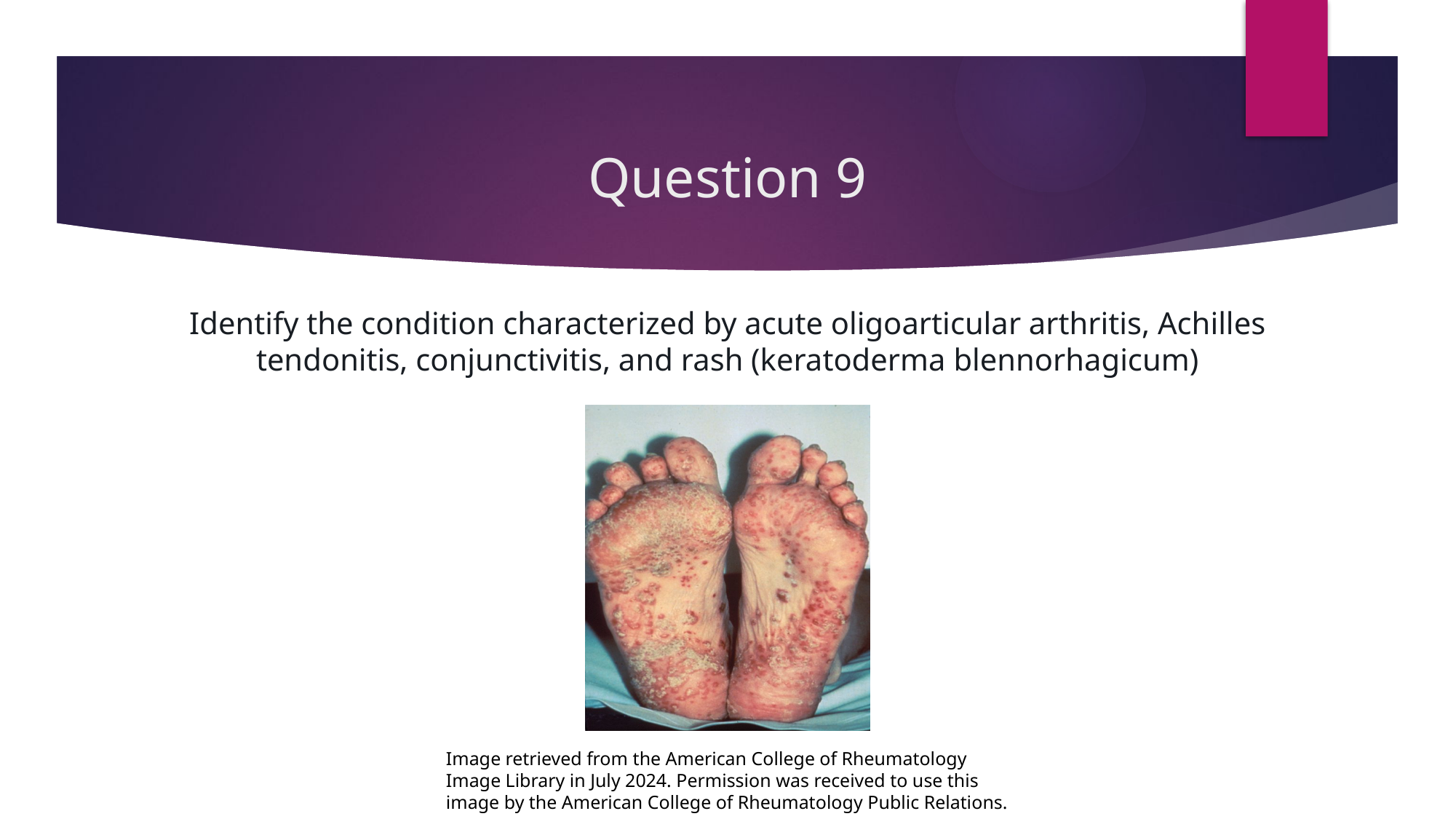

# Question 9
Identify the condition characterized by acute oligoarticular arthritis, Achilles tendonitis, conjunctivitis, and rash (keratoderma blennorhagicum)
Image retrieved from the American College of Rheumatology Image Library in July 2024. Permission was received to use this image by the American College of Rheumatology Public Relations.

## Slide 27
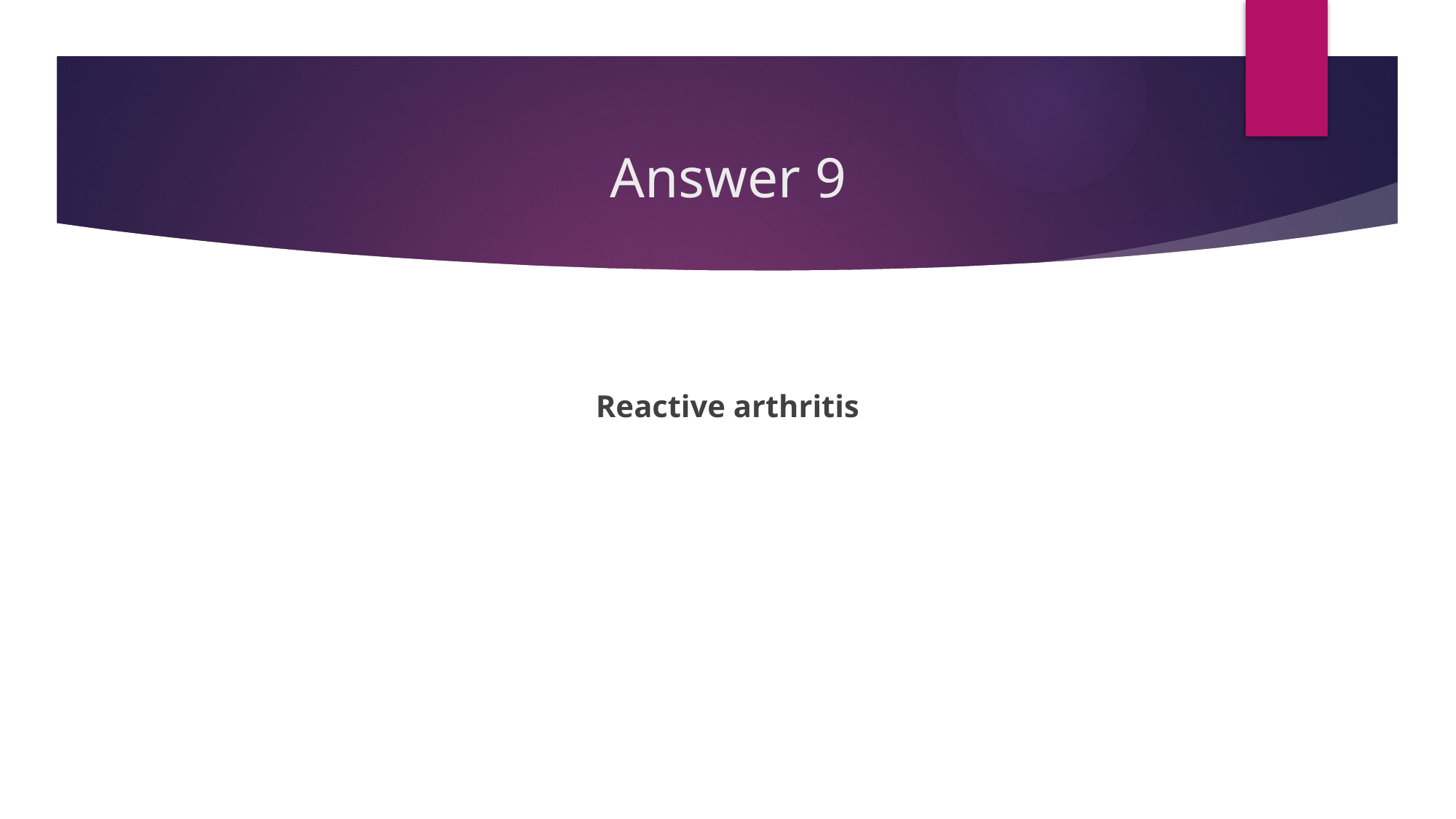

# Answer 9
Reactive arthritis

## Slide 28
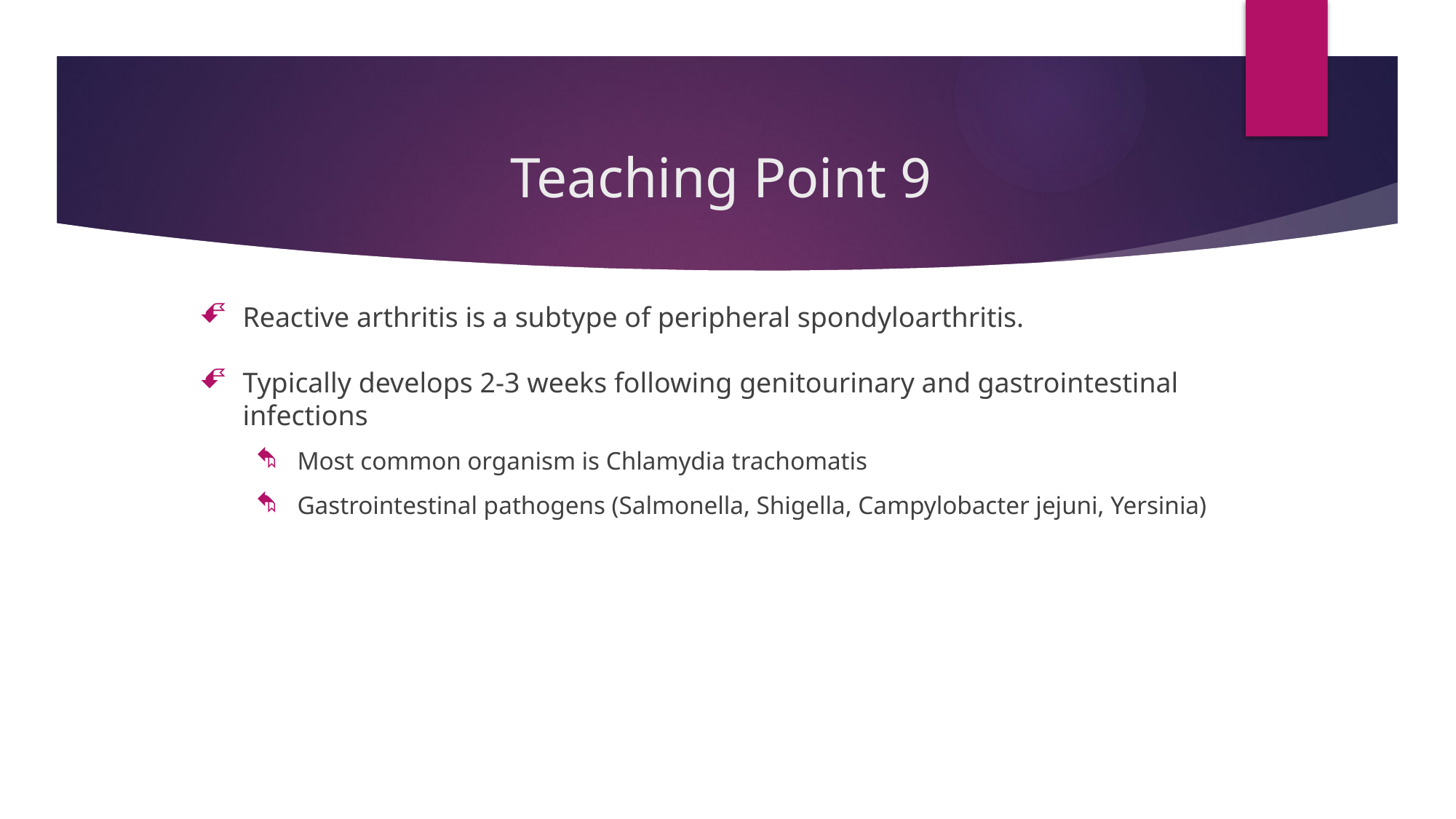

# Teaching Point 9
Reactive arthritis is a subtype of peripheral spondyloarthritis.
Typically develops 2-3 weeks following genitourinary and gastrointestinal infections
Most common organism is Chlamydia trachomatis
Gastrointestinal pathogens (Salmonella, Shigella, Campylobacter jejuni, Yersinia)

## Slide 29
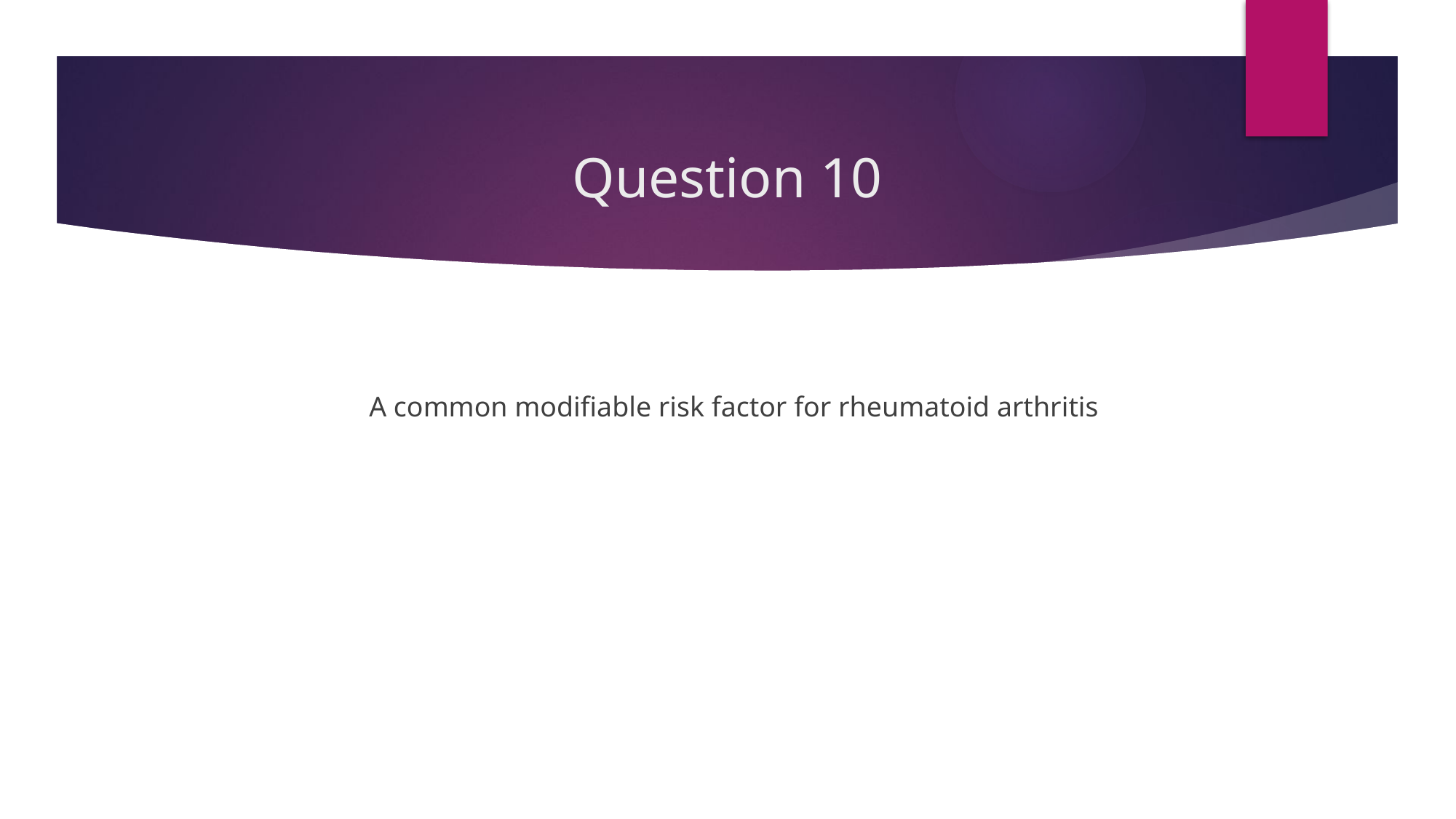

# Question 10
A common modifiable risk factor for rheumatoid arthritis

## Slide 30
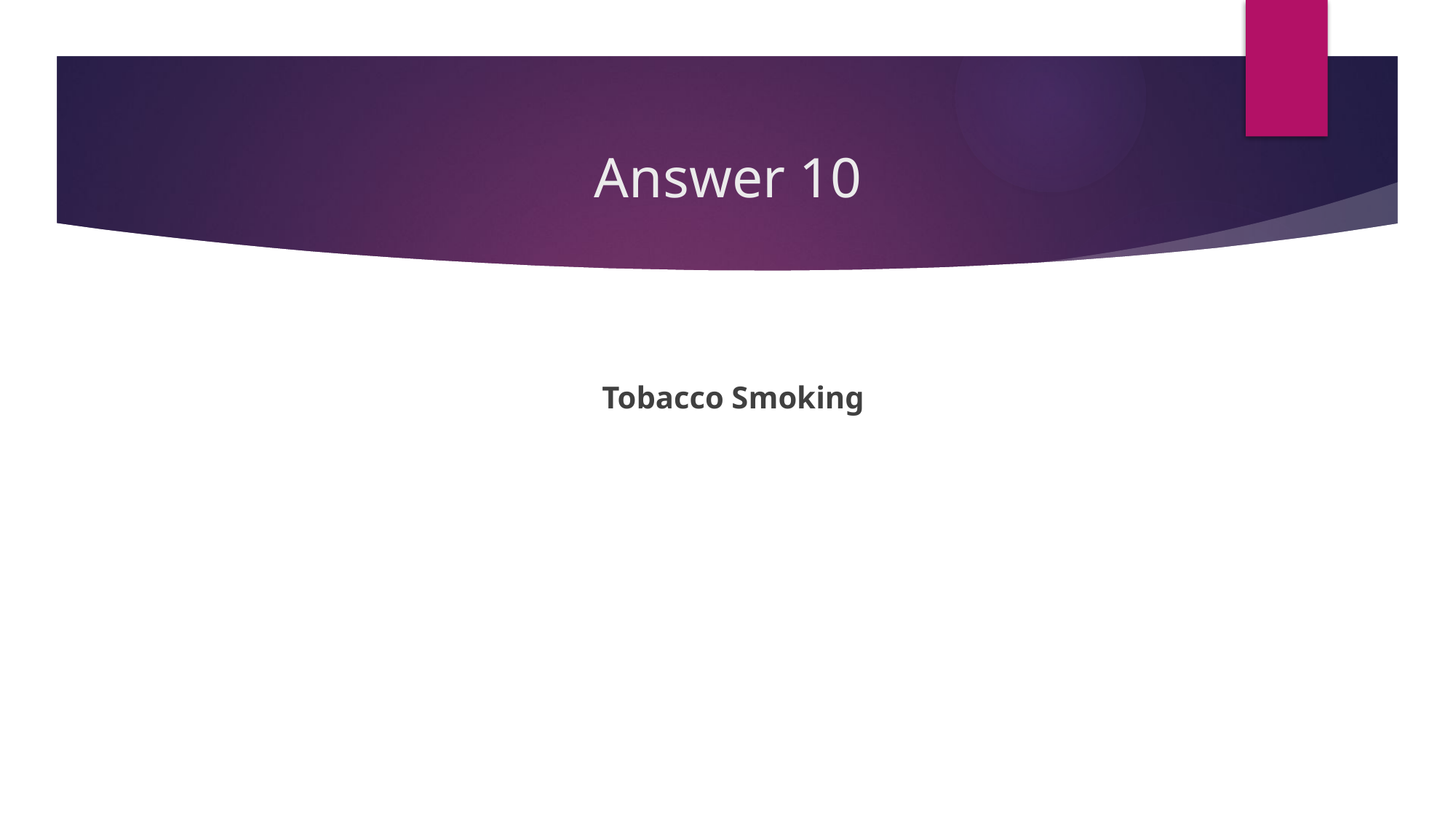

# Answer 10
Tobacco Smoking

## Slide 31
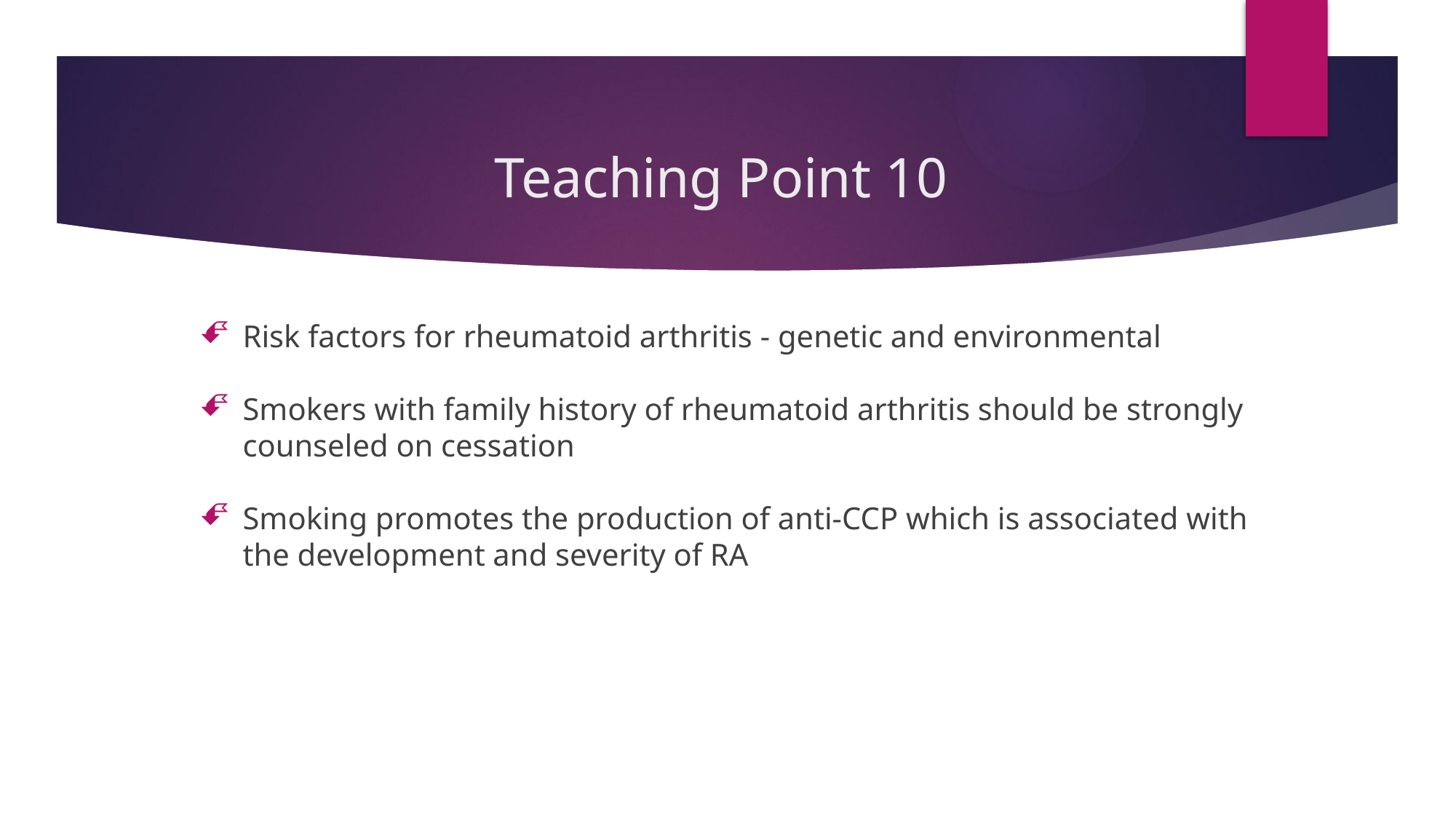

# Teaching Point 10
Risk factors for rheumatoid arthritis - genetic and environmental
Smokers with family history of rheumatoid arthritis should be strongly counseled on cessation
Smoking promotes the production of anti-CCP which is associated with the development and severity of RA

## Slide 32
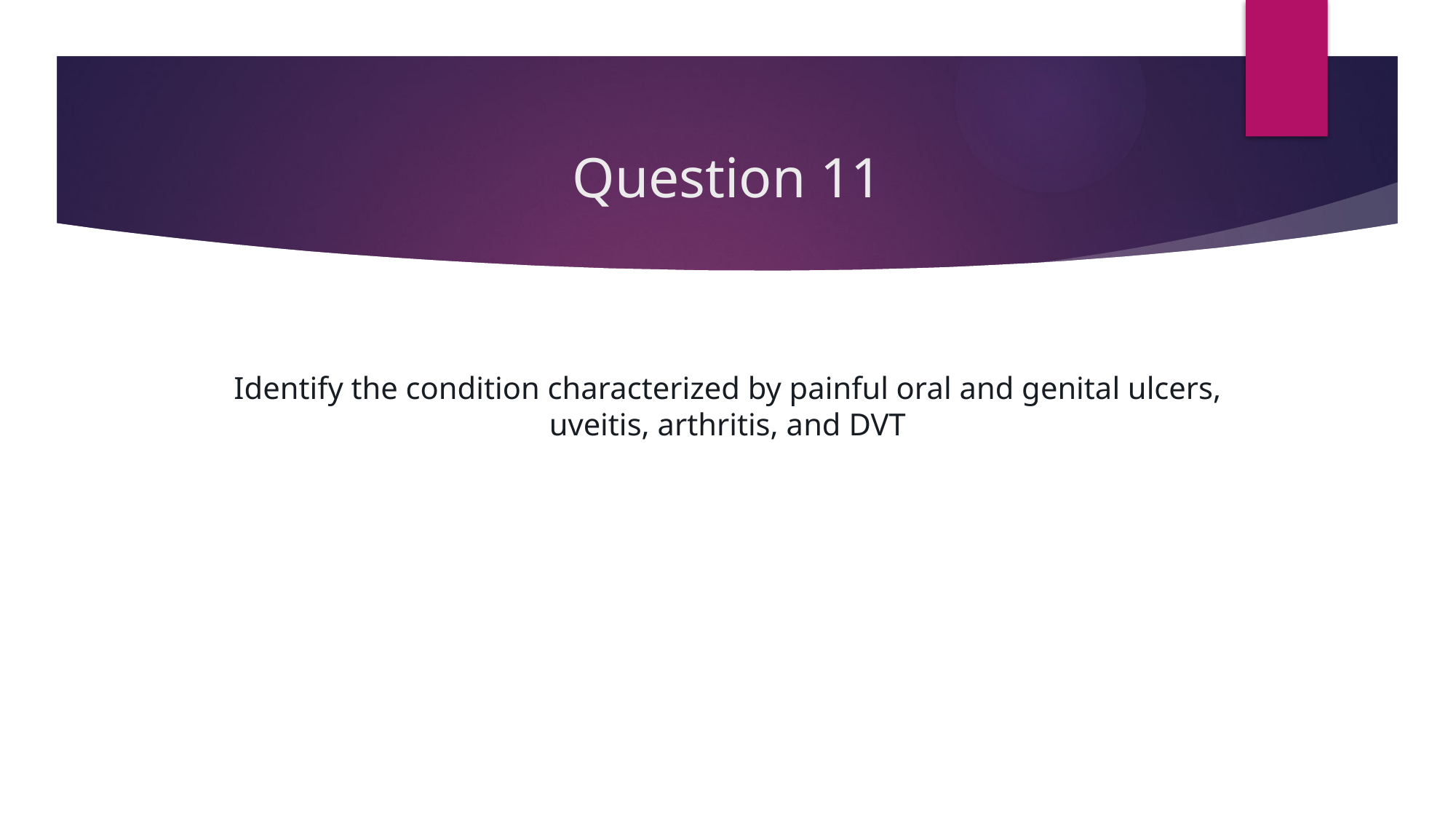

# Question 11
Identify the condition characterized by painful oral and genital ulcers, uveitis, arthritis, and DVT

## Slide 33
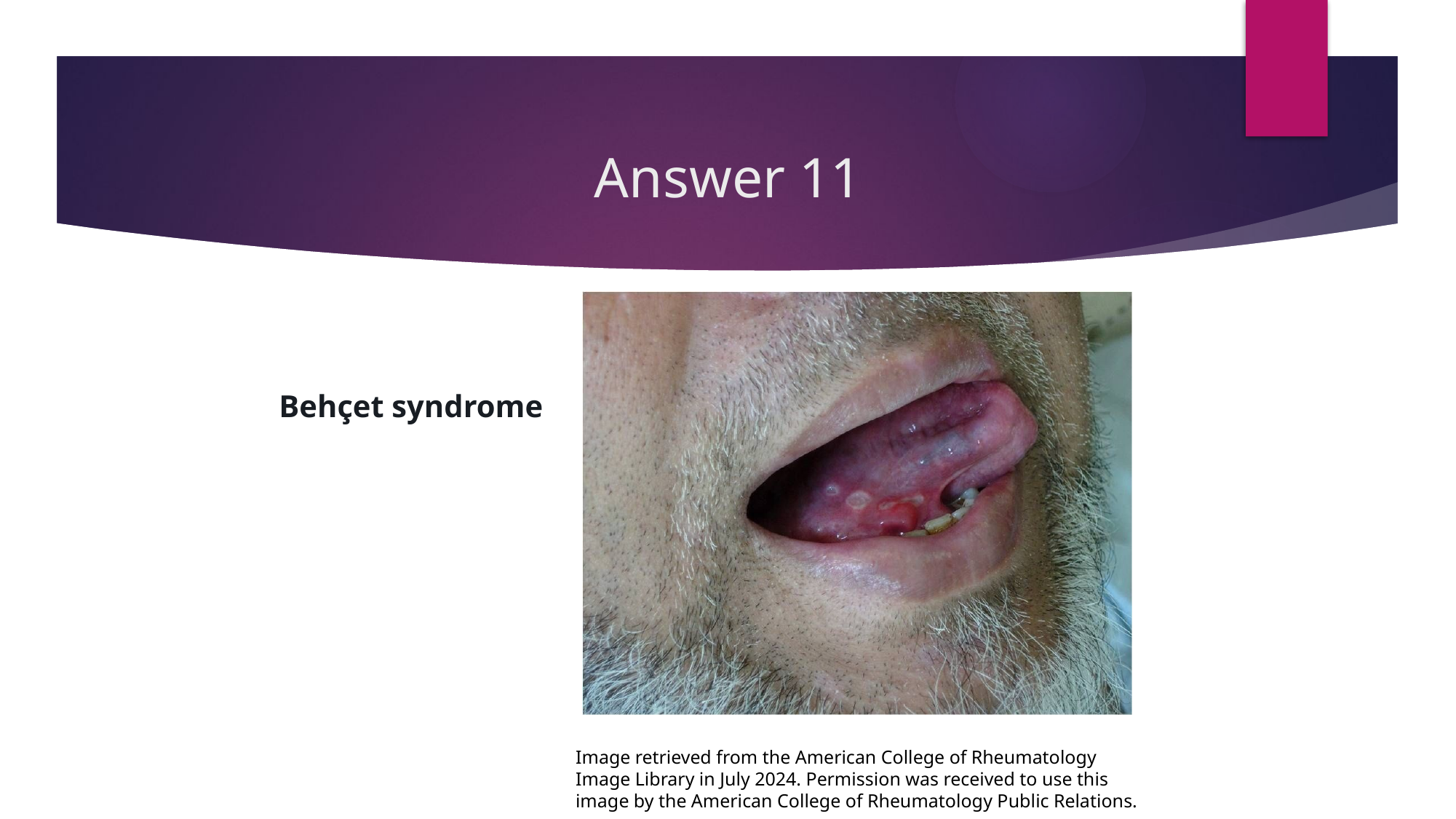

# Answer 11
Behçet syndrome
Image retrieved from the American College of Rheumatology Image Library in July 2024. Permission was received to use this image by the American College of Rheumatology Public Relations.

## Slide 34
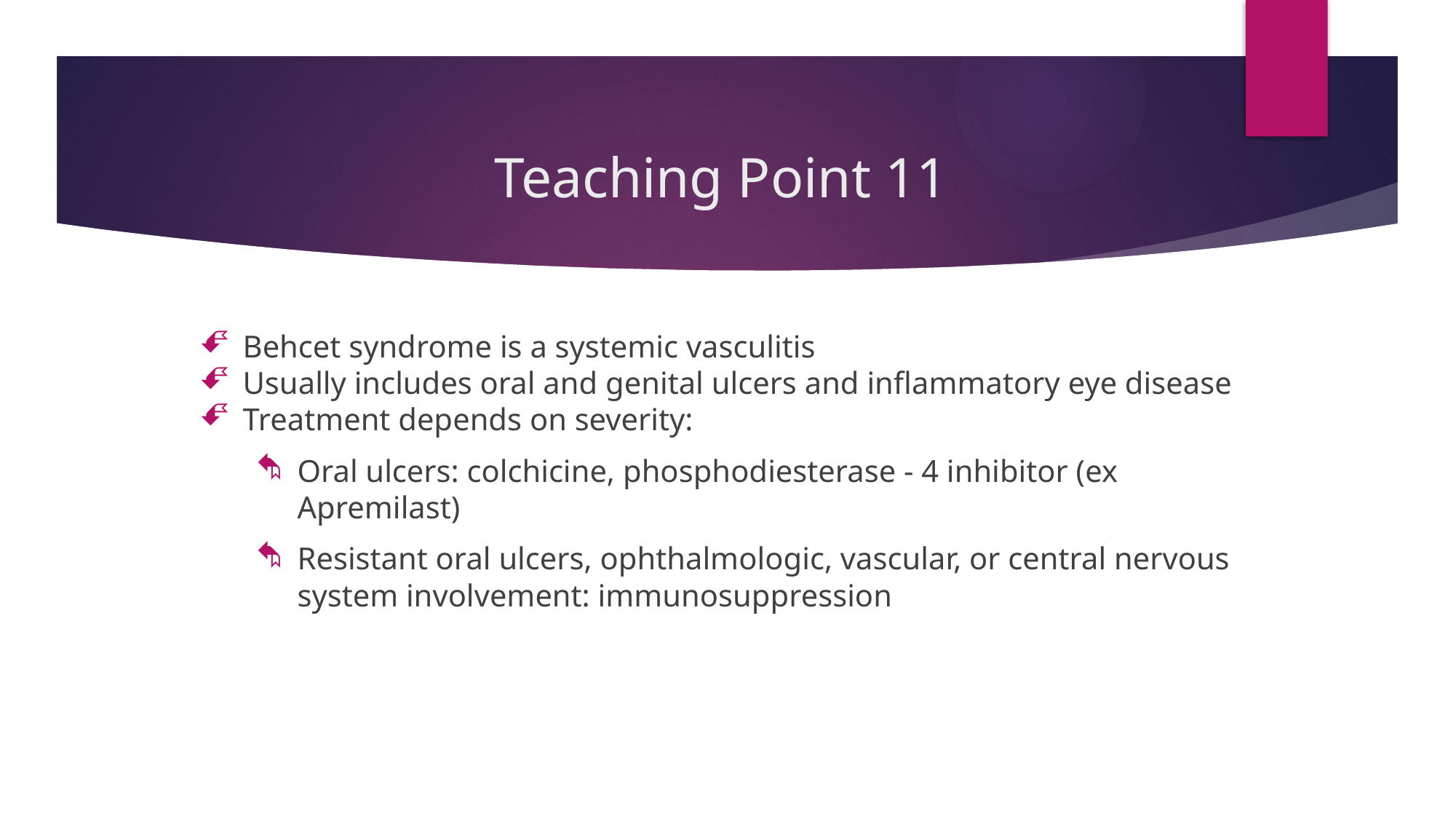

# Teaching Point 11
Behcet syndrome is a systemic vasculitis
Usually includes oral and genital ulcers and inflammatory eye disease
Treatment depends on severity:
Oral ulcers: colchicine, phosphodiesterase - 4 inhibitor (ex Apremilast)
Resistant oral ulcers, ophthalmologic, vascular, or central nervous system involvement: immunosuppression

## Slide 35
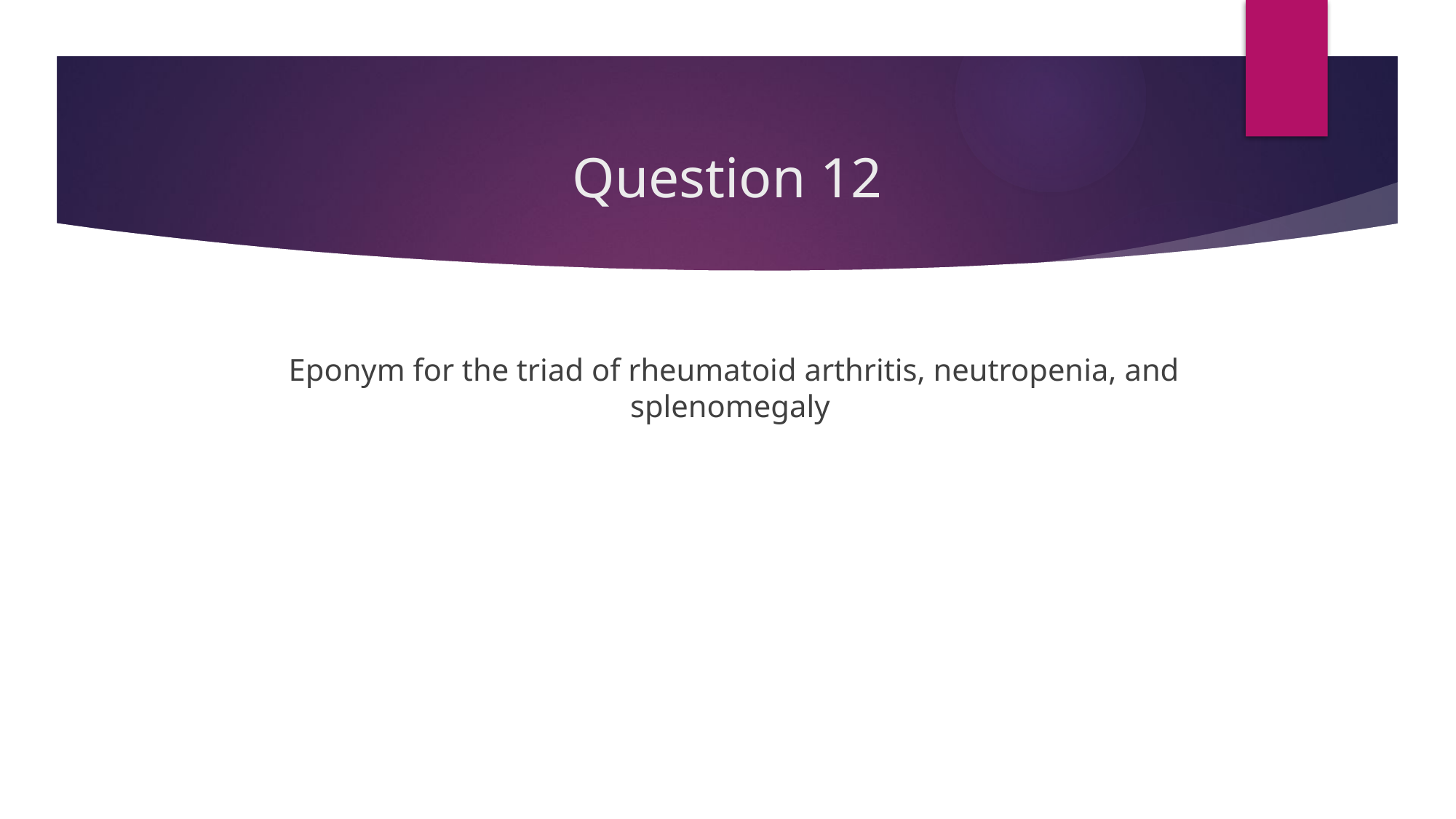

# Question 12
Eponym for the triad of rheumatoid arthritis, neutropenia, and splenomegaly

## Slide 36
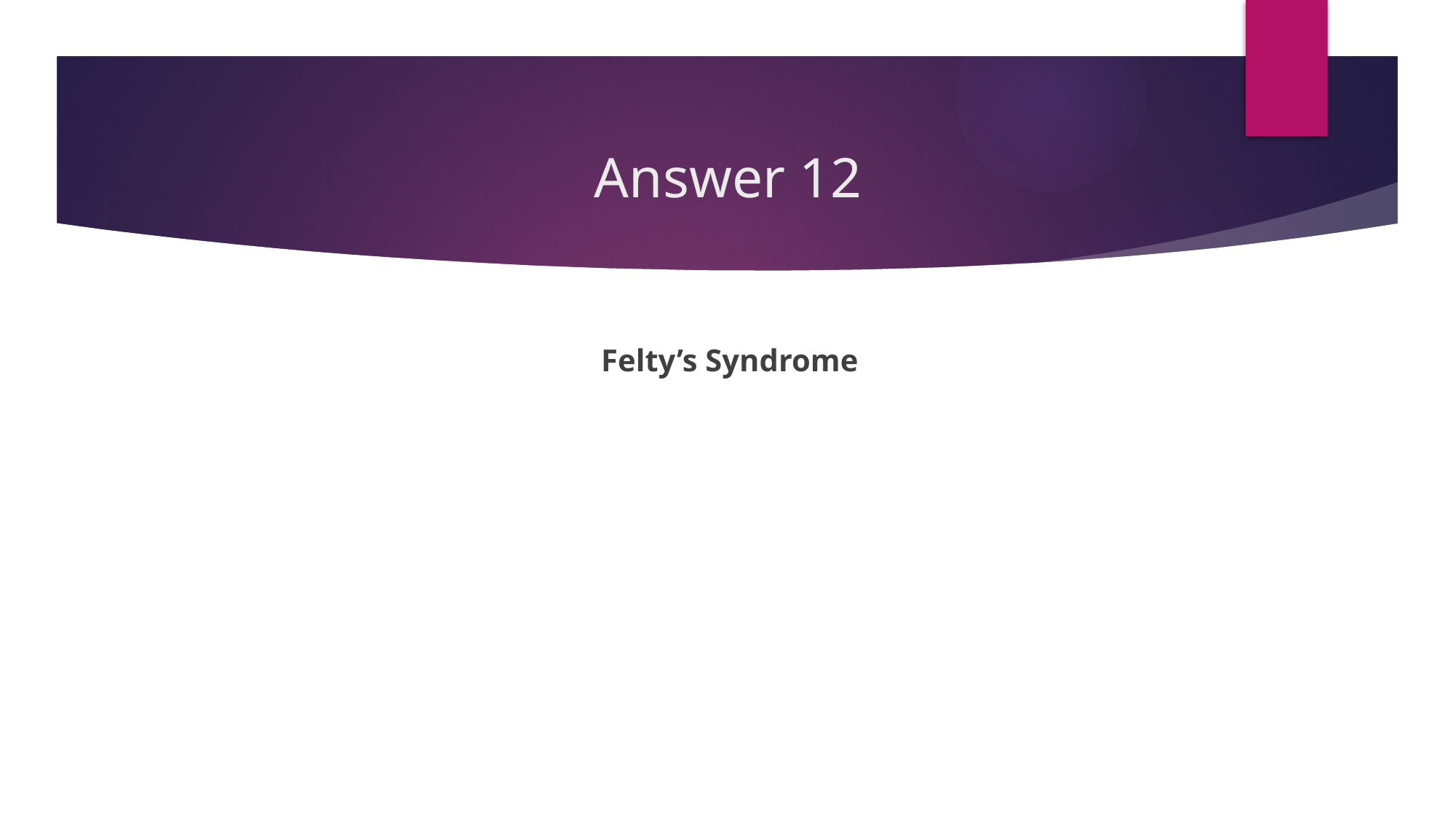

# Answer 12
Felty’s Syndrome

## Slide 37
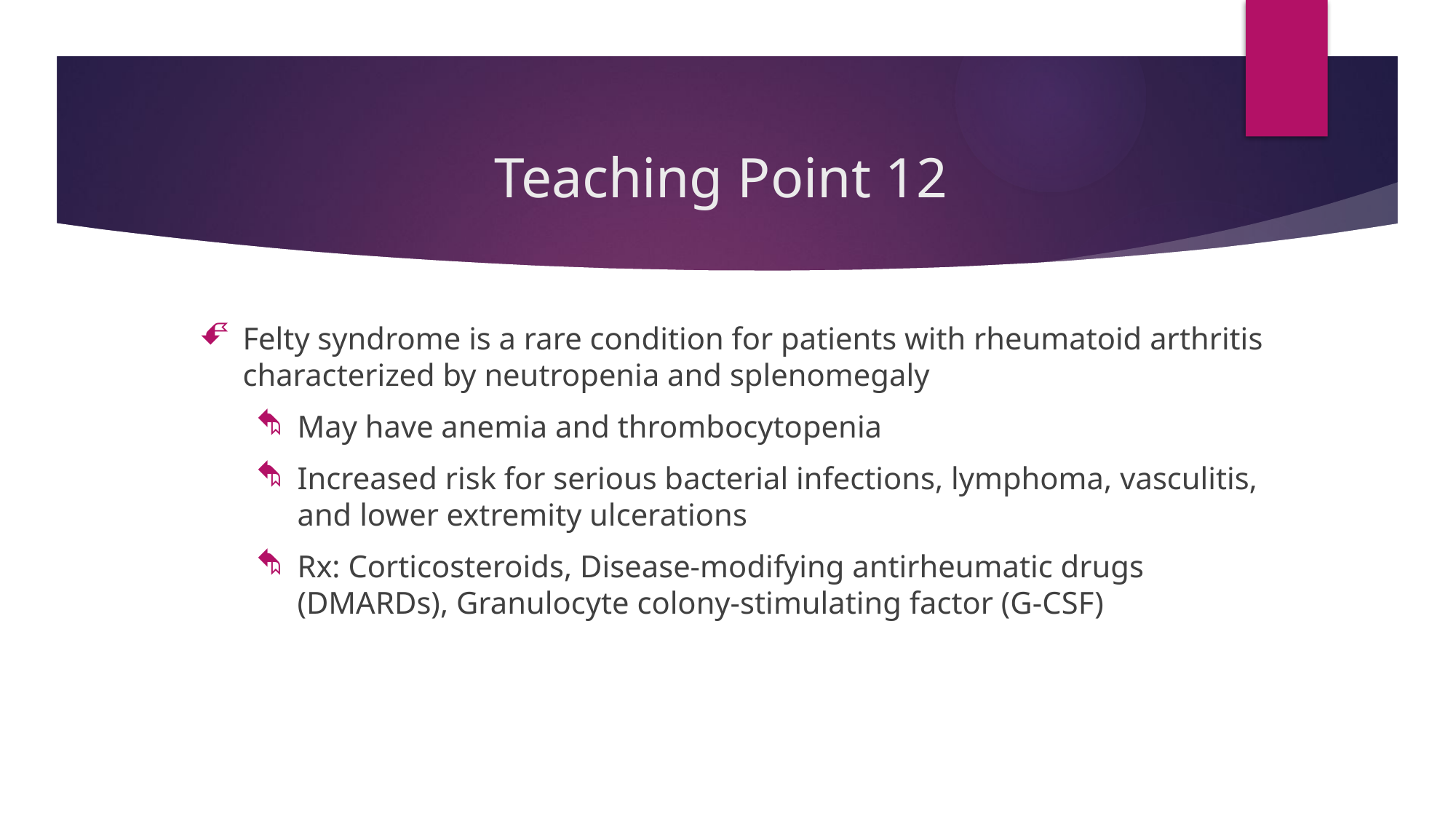

# Teaching Point 12
Felty syndrome is a rare condition for patients with rheumatoid arthritis characterized by neutropenia and splenomegaly
May have anemia and thrombocytopenia
Increased risk for serious bacterial infections, lymphoma, vasculitis, and lower extremity ulcerations
Rx: Corticosteroids, Disease-modifying antirheumatic drugs (DMARDs), Granulocyte colony-stimulating factor (G-CSF)

## Slide 38
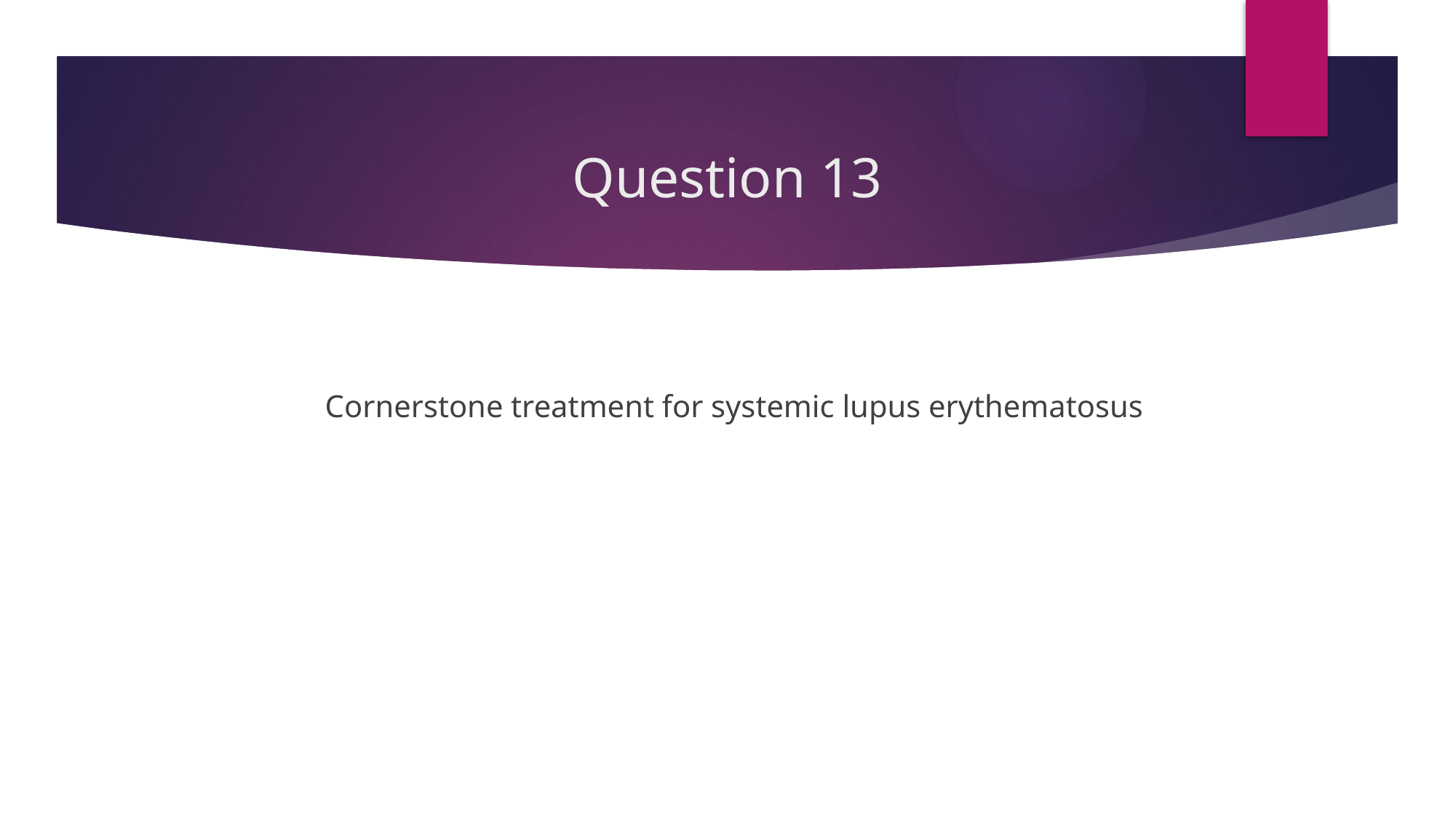

# Question 13
Cornerstone treatment for systemic lupus erythematosus

## Slide 39
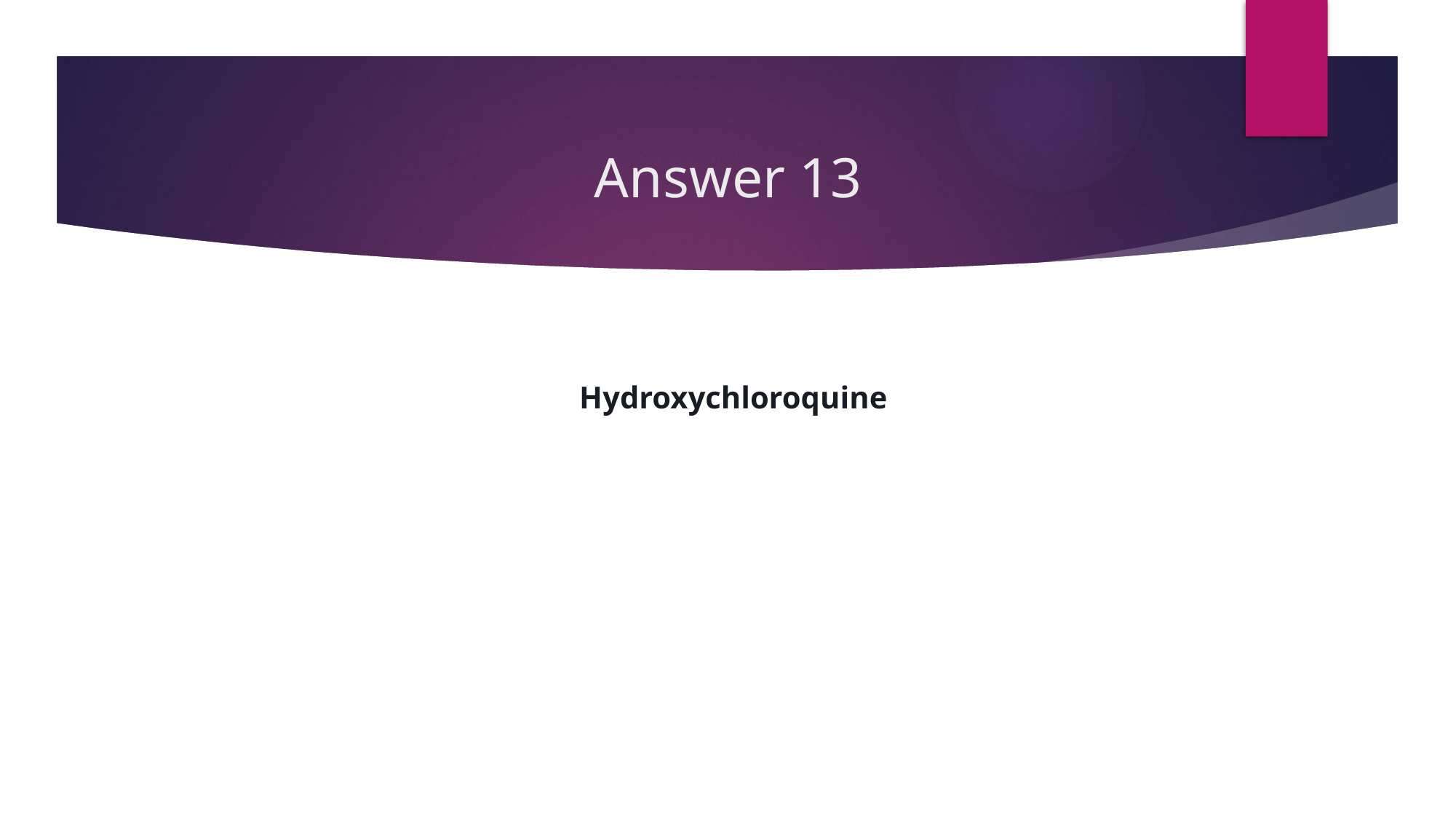

# Answer 13
Hydroxychloroquine

## Slide 40
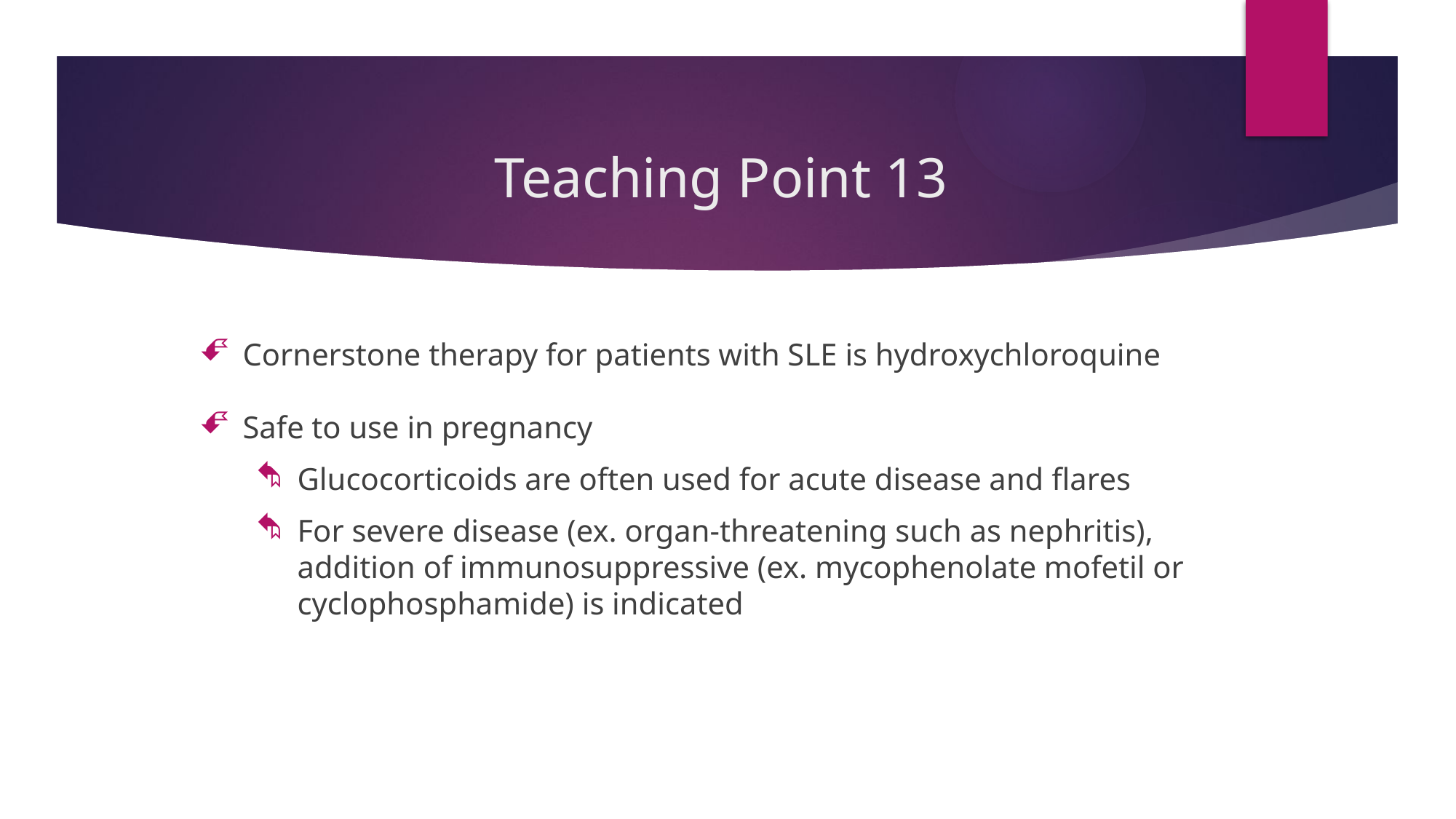

# Teaching Point 13
Cornerstone therapy for patients with SLE is hydroxychloroquine
Safe to use in pregnancy
Glucocorticoids are often used for acute disease and flares
For severe disease (ex. organ-threatening such as nephritis), addition of immunosuppressive (ex. mycophenolate mofetil or cyclophosphamide) is indicated

## Slide 41
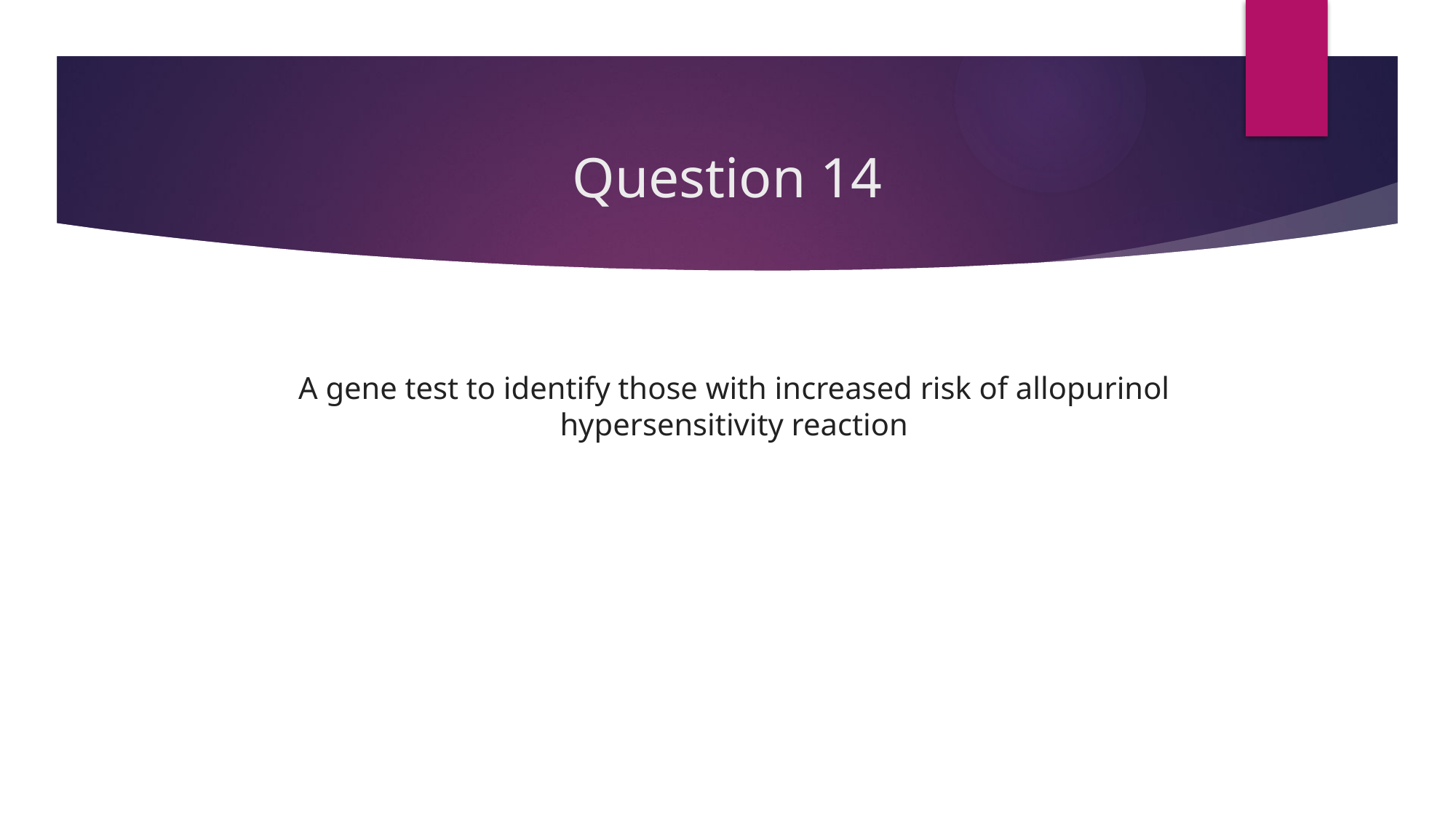

# Question 14
A gene test to identify those with increased risk of allopurinol hypersensitivity reaction

## Slide 42
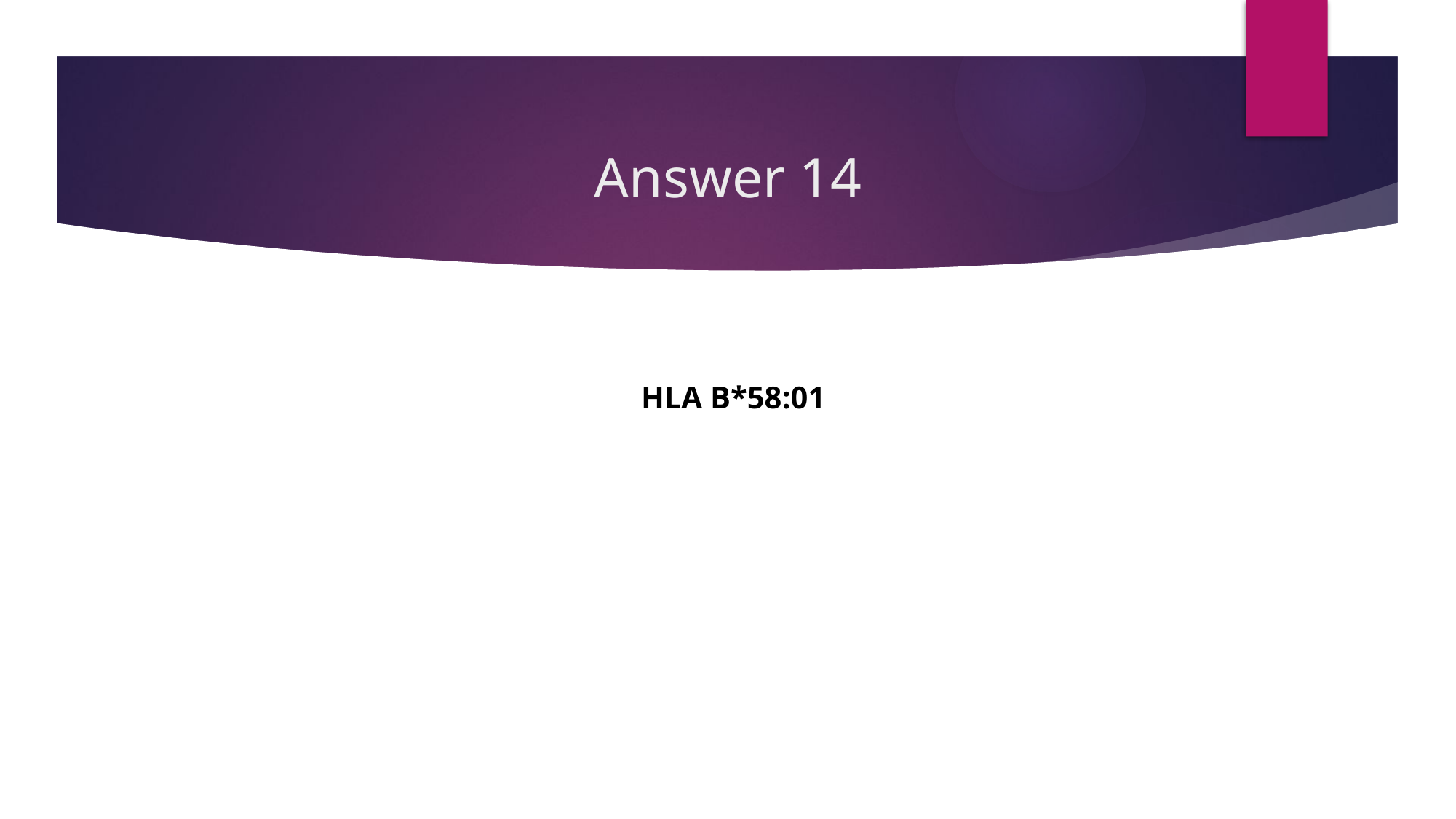

# Answer 14
HLA B*58:01

## Slide 43
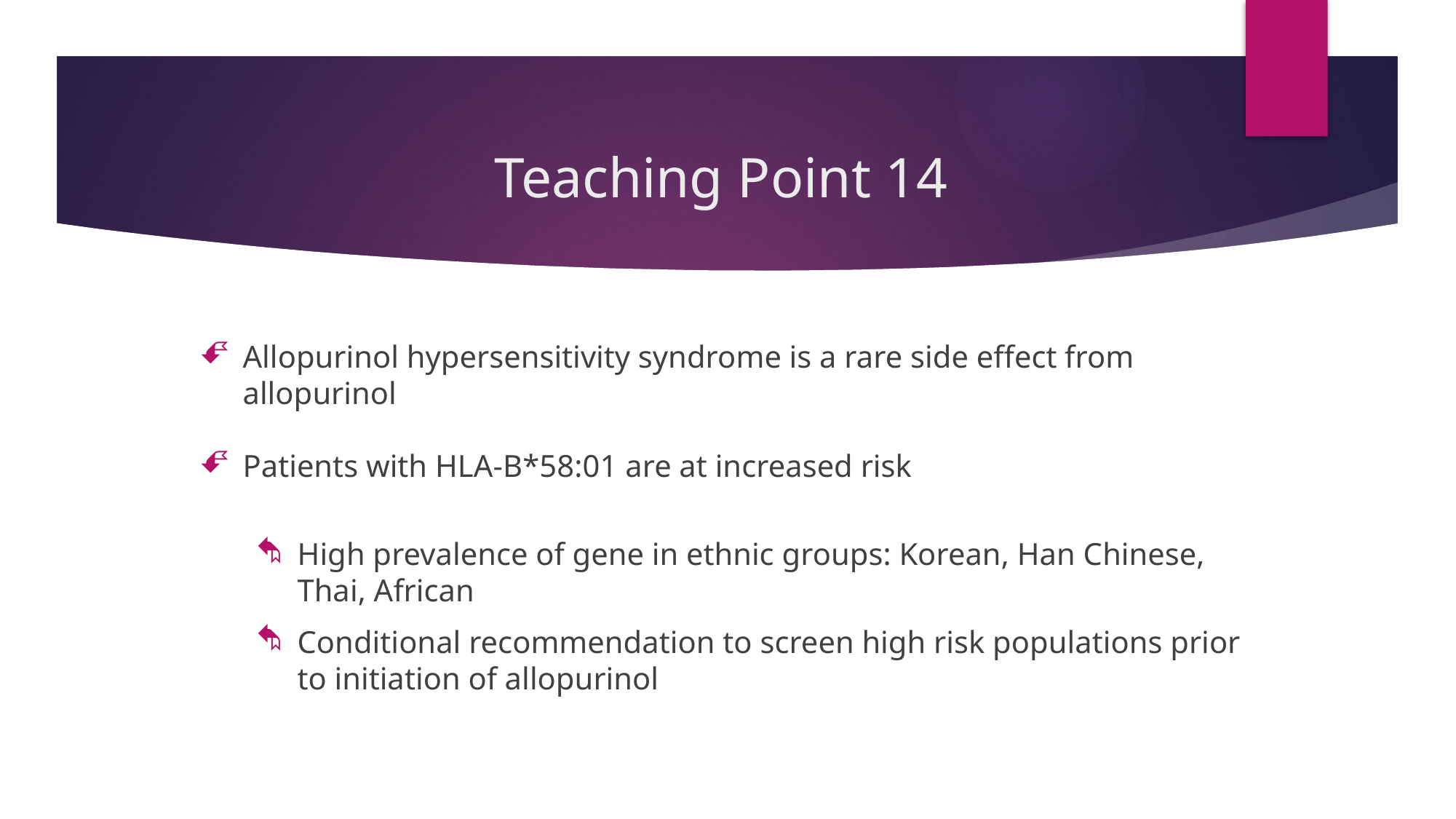

# Teaching Point 14
Allopurinol hypersensitivity syndrome is a rare side effect from allopurinol
Patients with HLA-B*58:01 are at increased risk
High prevalence of gene in ethnic groups: Korean, Han Chinese, Thai, African
Conditional recommendation to screen high risk populations prior to initiation of allopurinol

## Slide 44
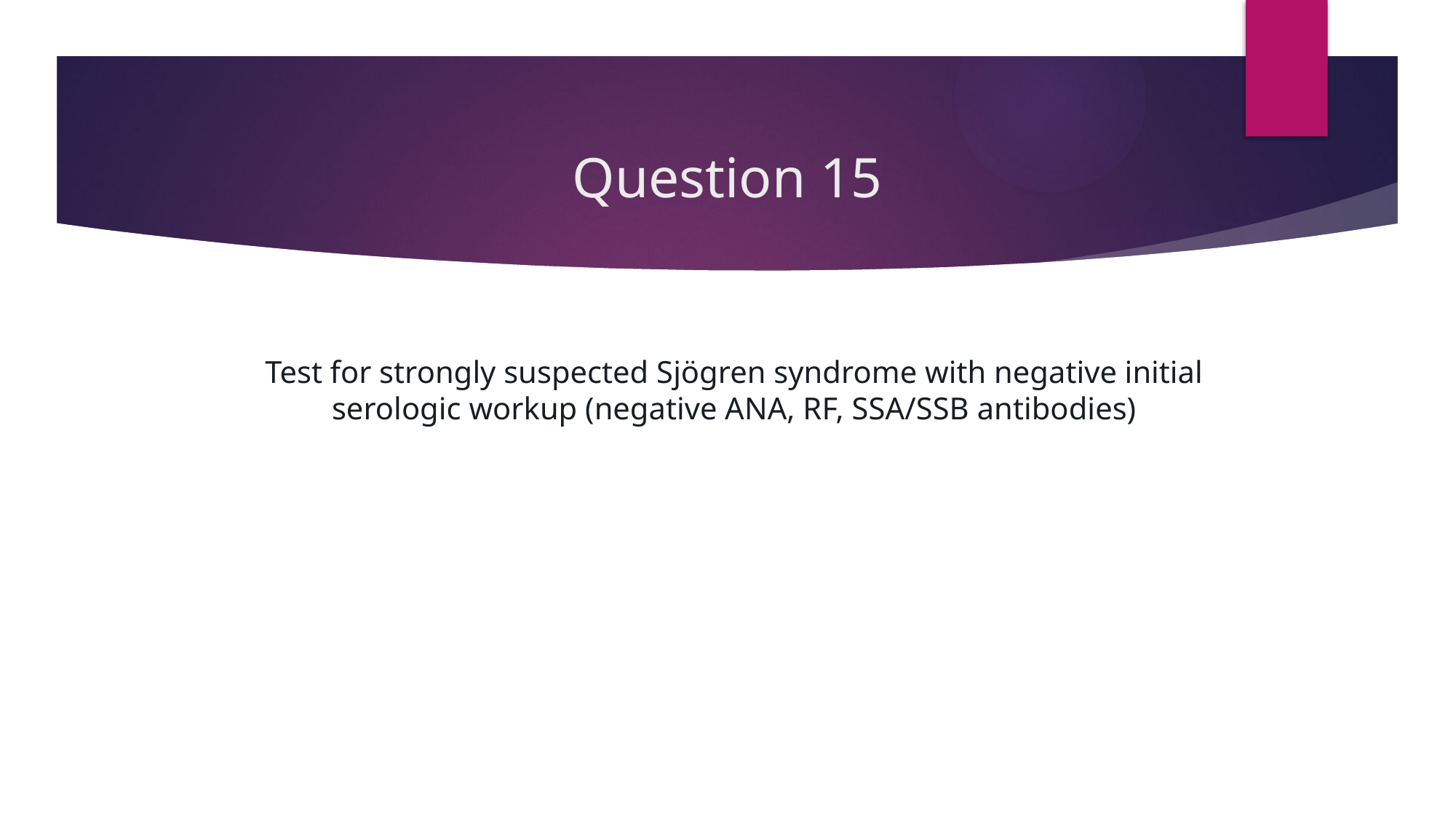

# Question 15
Test for strongly suspected Sjögren syndrome with negative initial serologic workup (negative ANA, RF, SSA/SSB antibodies)

## Slide 45
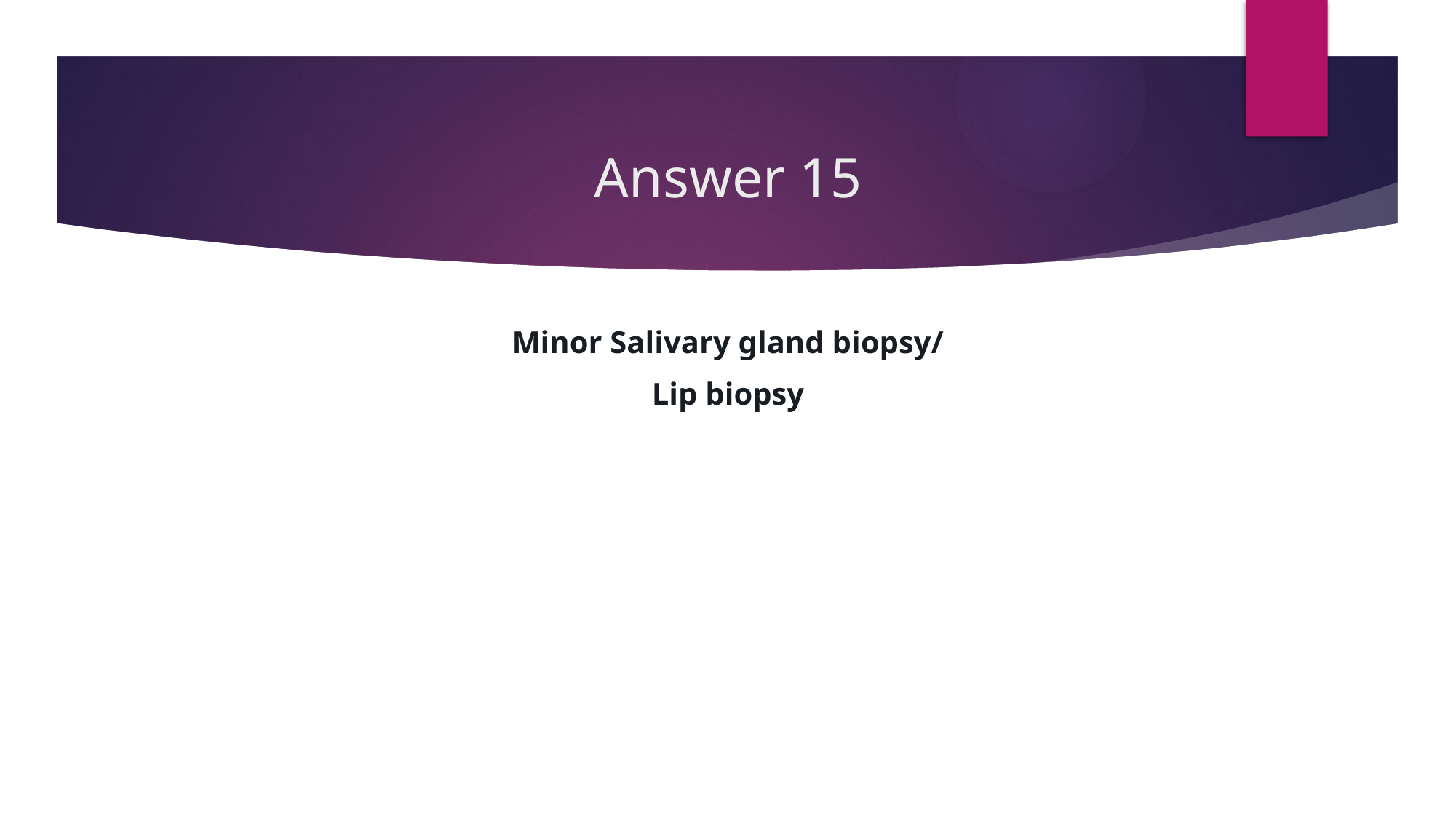

# Answer 15
Minor Salivary gland biopsy/
Lip biopsy

## Slide 46
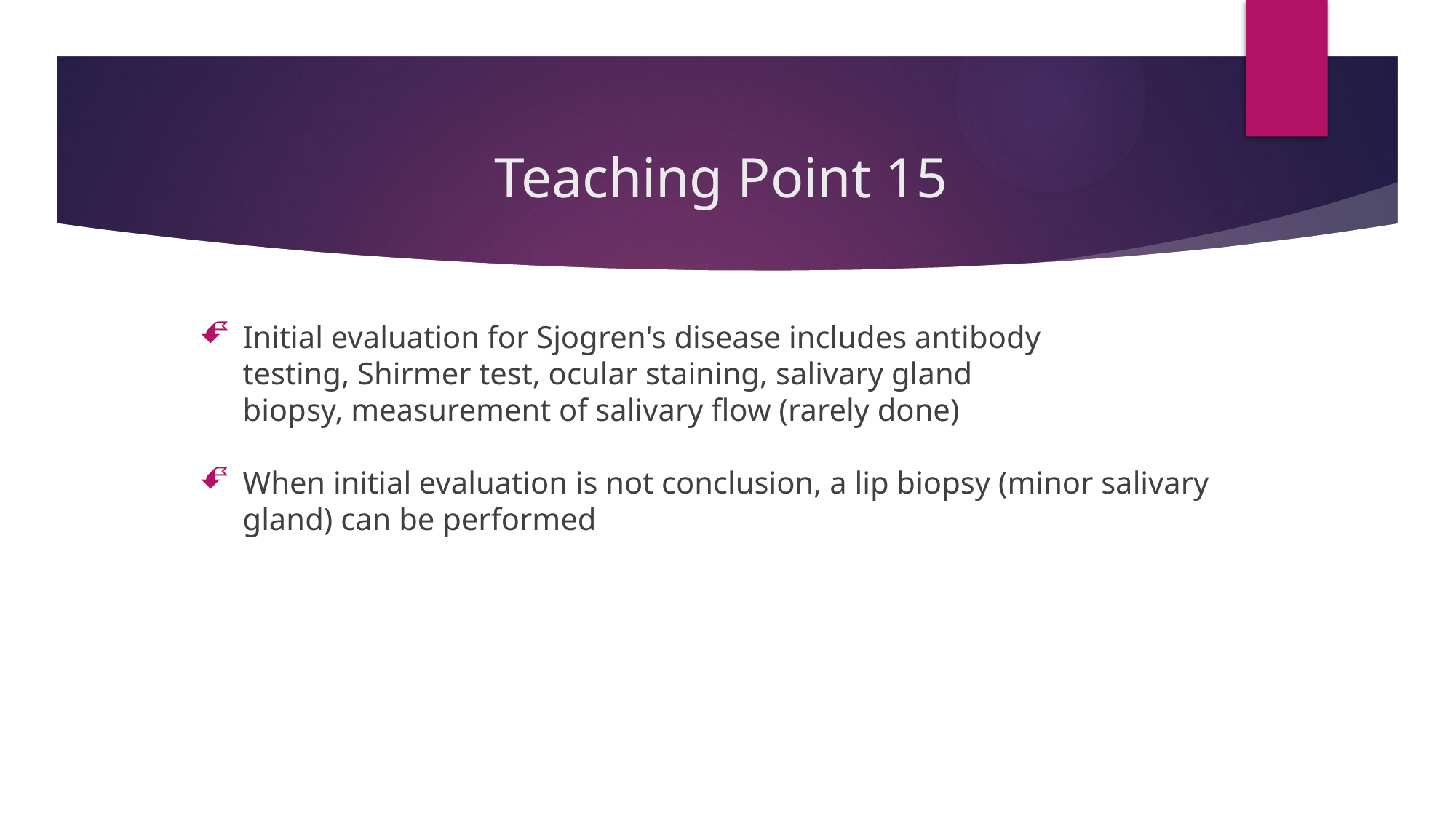

# Teaching Point 15
Initial evaluation for Sjogren's disease includes antibody testing, Shirmer test, ocular staining, salivary gland biopsy, measurement of salivary flow (rarely done)
When initial evaluation is not conclusion, a lip biopsy (minor salivary gland) can be performed
