# Supplementary material for: Rheum4Games: A Game-Based Board Review to Enhance Confidence and Knowledge in Rheumatology for Internal Medicine Residents
Source: MedEdPORTAL. 2026 May 1;22:11597. doi: 10.15766/mep_2374-8265.11597 (PMC13133093; doi:10.15766/mep_2374-8265.11597)
Supplement: Supplementary file 1 — Question Bank - Easier.pptxQuestion Bank - Challenging.pptxSurvey.docxGame Rules.pptxBoard Game.docx [file mep_2374-8265.11597-s001.zip › C. Survey.docx]

Thank you for participating in the “Rheum4Games” board review activity. During this session you will complete a board game activity similar to “Snakes and Ladders.” This activity is meant to improve your understanding of rheumatology. We request you complete a pre-activity and a post-activity survey. Each survey should take less than 5 minutes to complete. We value your privacy and will not share your individual responses with your residency program or others in a way that would let them identify who made the comments or provided specific feedback. Your responses will in no way impact your current or future relationship with your residency program. The information gathered will be used only for this educational research study for evaluation and strategic planning purposes only.

Pre-Activity Survey:

1. Name of your residency program: ___________________________________
2. Select your PGY level:

- PGY 1
- PGY 2
- PGY3
- Other:

1. How many weeks of rheumatology elective have you completed so far during residency?

- None
- 1 week
- 2 weeks
- 3 weeks
- ≥4 weeks

1. Which of the following resources have you utilized to prepare for the rheumatology board review? Please select all that apply

- UWorld Question Bank
- ACP MKSAP
- ACP Board Basics
- Live board review course (in-person/virtual)
- Text books
- Other____________________________________________________
- None

1. Rate the following statement: I feel confident about the rheumatology section of the In-Training Examination (ITE) / American Board of Internal Medicine (ABIM) certification exam.

- Extremely confident
- Quite confident
- Moderately confident
- Slightly confident
- Not confident

Post-Activity Survey:

Please rate the following statements using the scale below:

| **Statements** | **Strongly disagree** | **Disagree** | **Neutral** | **Agree** | **Strongly Agree** |
| --- | --- | --- | --- | --- | --- |
| Overall, I enjoyed the “Rheum4Games” activity. |  |  |  |  |  |
| **This format was stressful.** |  |  |  |  |  |
| I found this game fun to play. |  |  |  |  |  |
| I felt like I was immersed in rheumatology. |  |  |  |  |  |
| **I prefer didactic lectures to the game.** |  |  |  |  |  |
| This activity increased my interest in rheumatology. |  |  |  |  |  |
| The challenges in the game were interesting to me. |  |  |  |  |  |
| The debriefing conducted by the game master improved my understanding in rheumatology. |  |  |  |  |  |
| This activity increased my general knowledge in rheumatology. |  |  |  |  |  |
| This format helped me retain rheumatology information. |  |  |  |  |  |
| This format helped me identify knowledge gaps. |  |  |  |  |  |
| The information I needed to solve questions was similar to information I need to pass my ITE/boards. |  |  |  |  |  |
| This activity sparked my interest to read more about rheumatology. |  |  |  |  |  |
| This activity encouraged the use of communication skills. |  |  |  |  |  |
| This activity encouraged the use of collaboration skills. |  |  |  |  |  |
| This activity encouraged the use of leadership skills |  |  |  |  |  |

Rate the following statement: Now after completing the activity, I feel confident about the rheumatology section of the In-Training Examination (ITE) / American Board of Internal Medicine (ABIM) certification exam.

- Extremely confident
- Quite confident
- Moderately confident
- Slightly confident
- Not confident

Any additional comments/feedback: __________________________________________________________________________________________________________________________________________________________________________
