# Supplementary material for: Rheum4Games: A Game-Based Board Review to Enhance Confidence and Knowledge in Rheumatology for Internal Medicine Residents
Source: MedEdPORTAL. 2026 May 1;22:11597. doi: 10.15766/mep_2374-8265.11597 (PMC13133093; doi:10.15766/mep_2374-8265.11597)
Supplement: Supplementary file 1 — Question Bank - Easier.pptxQuestion Bank - Challenging.pptxSurvey.docxGame Rules.pptxBoard Game.docx [file mep_2374-8265.11597-s001.zip › D. Game Rules.pptx]

## Slide 1
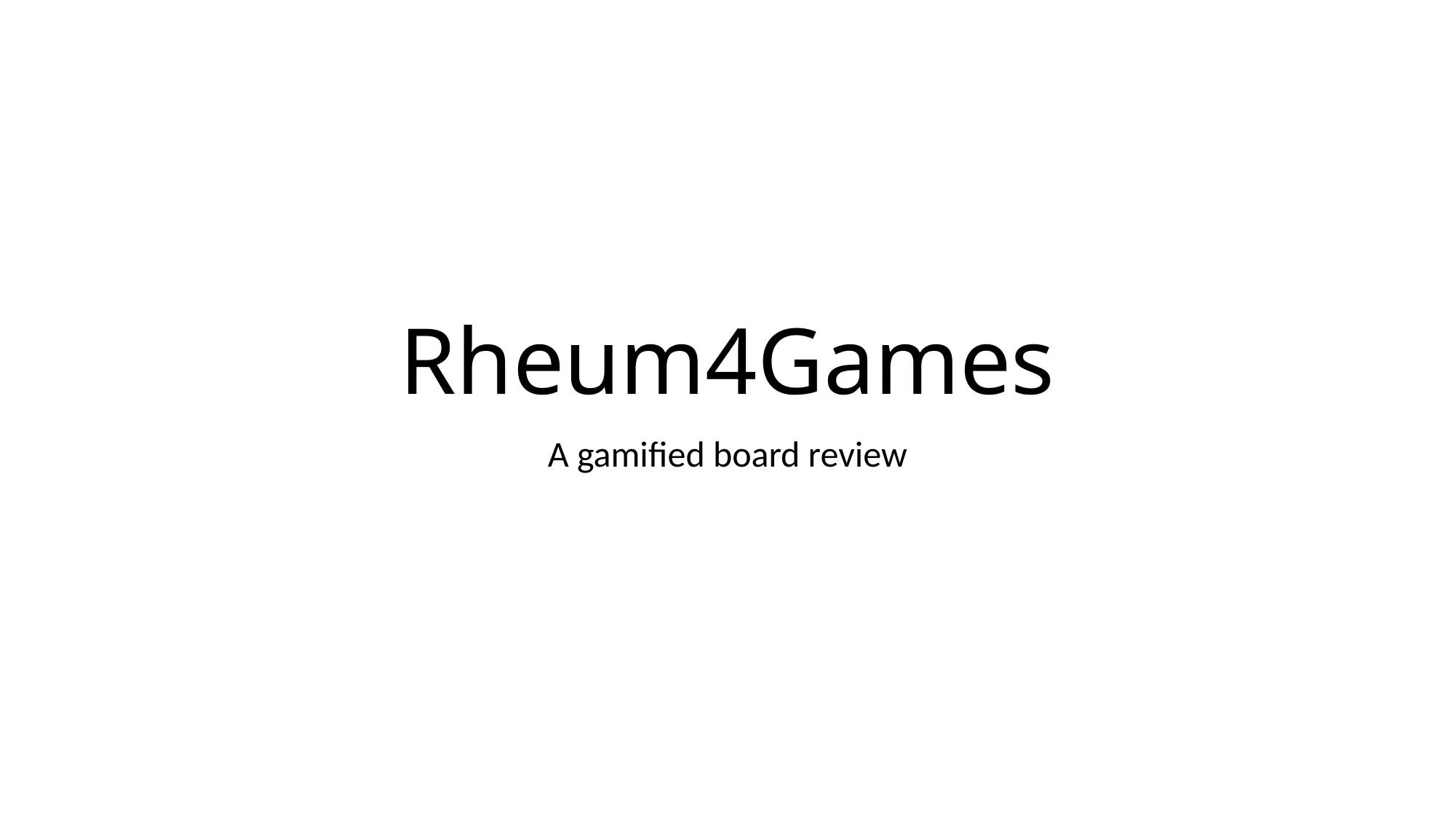

# Rheum4Games
A gamified board review

## Slide 2
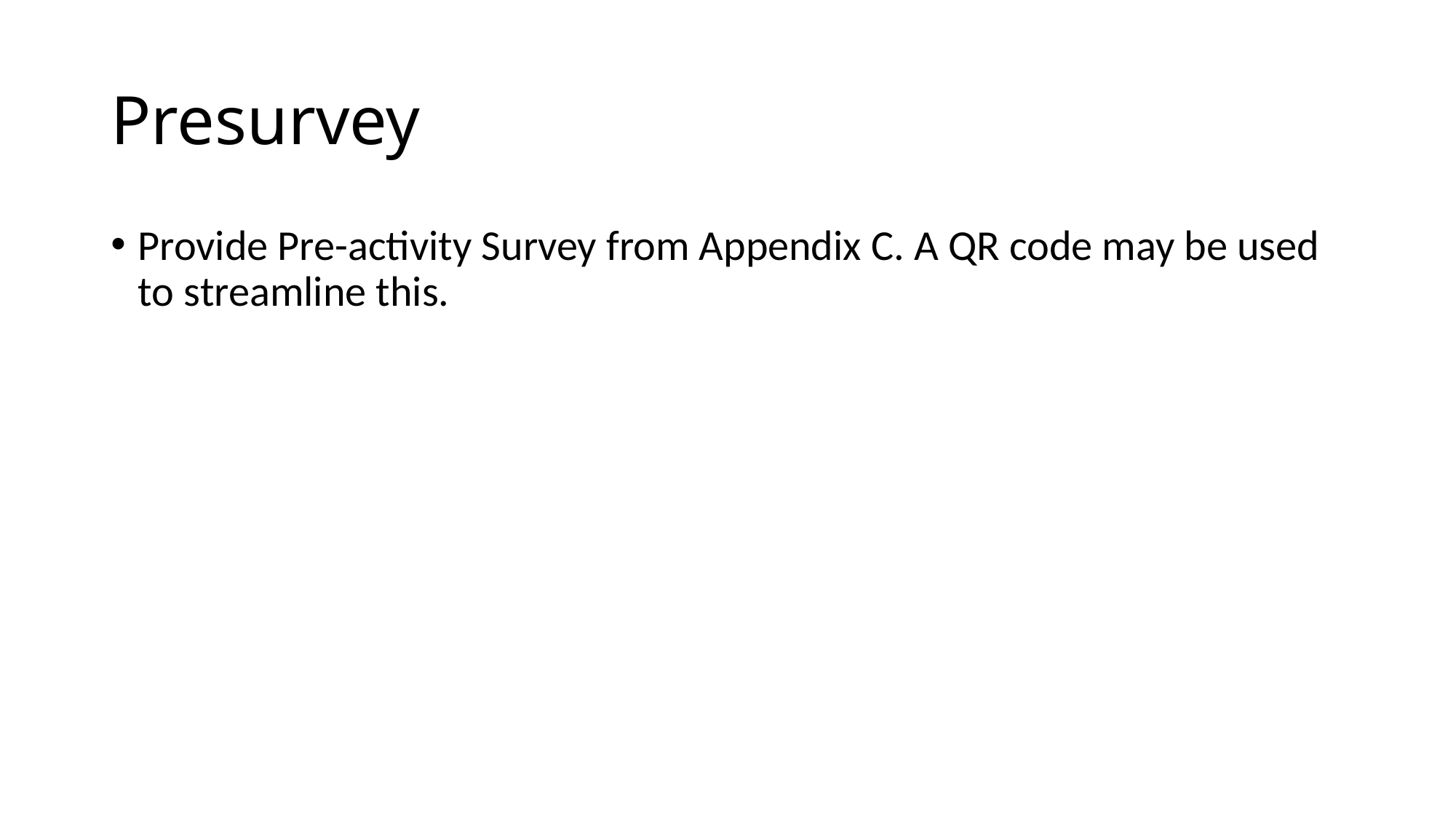

# Presurvey
Provide Pre-activity Survey from Appendix C. A QR code may be used to streamline this.

## Slide 3
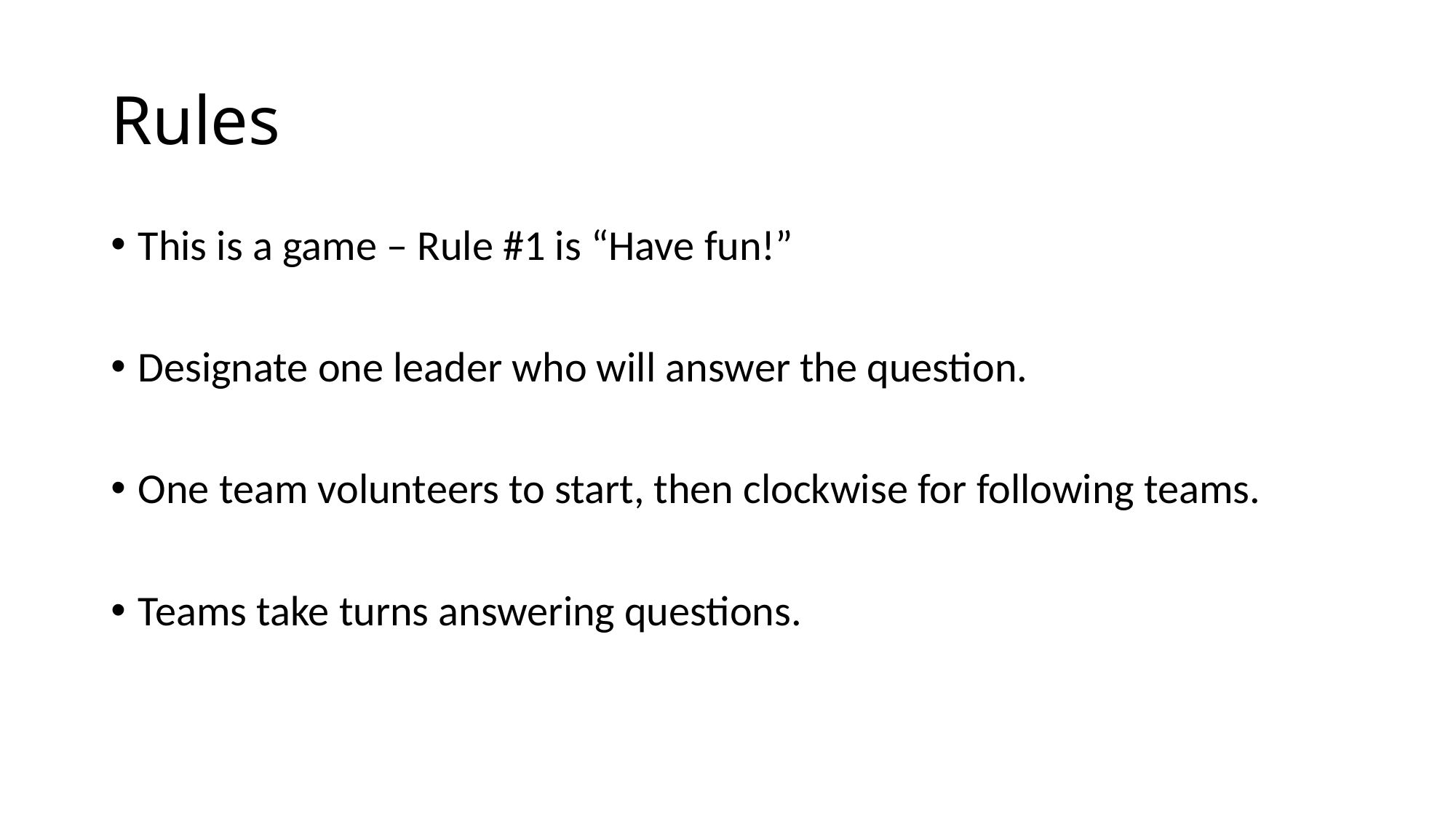

# Rules
This is a game – Rule #1 is “Have fun!”
Designate one leader who will answer the question.
One team volunteers to start, then clockwise for following teams.
Teams take turns answering questions.

## Slide 4
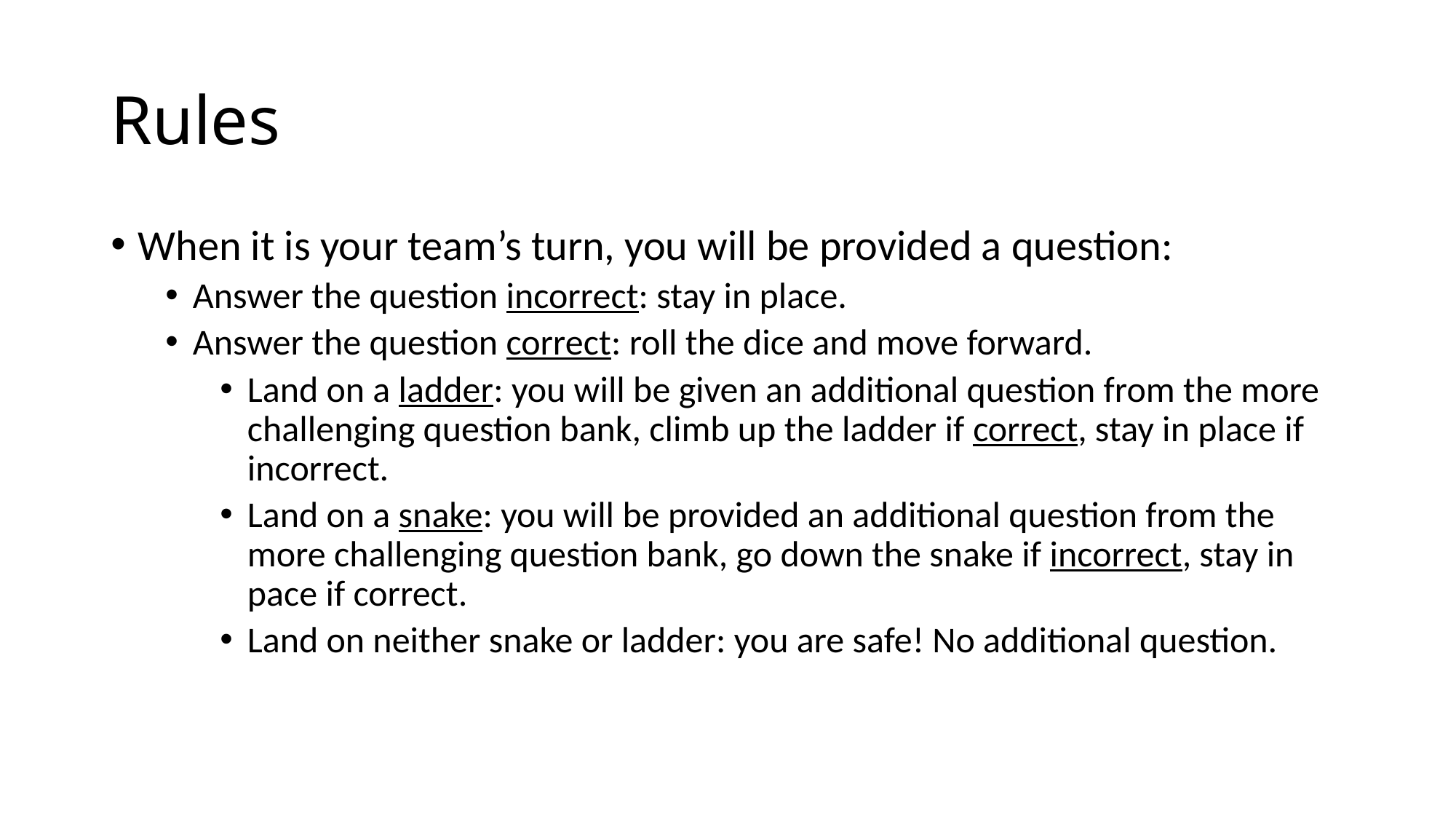

# Rules
When it is your team’s turn, you will be provided a question:
Answer the question incorrect: stay in place.
Answer the question correct: roll the dice and move forward.
Land on a ladder: you will be given an additional question from the more challenging question bank, climb up the ladder if correct, stay in place if incorrect.
Land on a snake: you will be provided an additional question from the more challenging question bank, go down the snake if incorrect, stay in pace if correct.
Land on neither snake or ladder: you are safe! No additional question.

## Slide 5
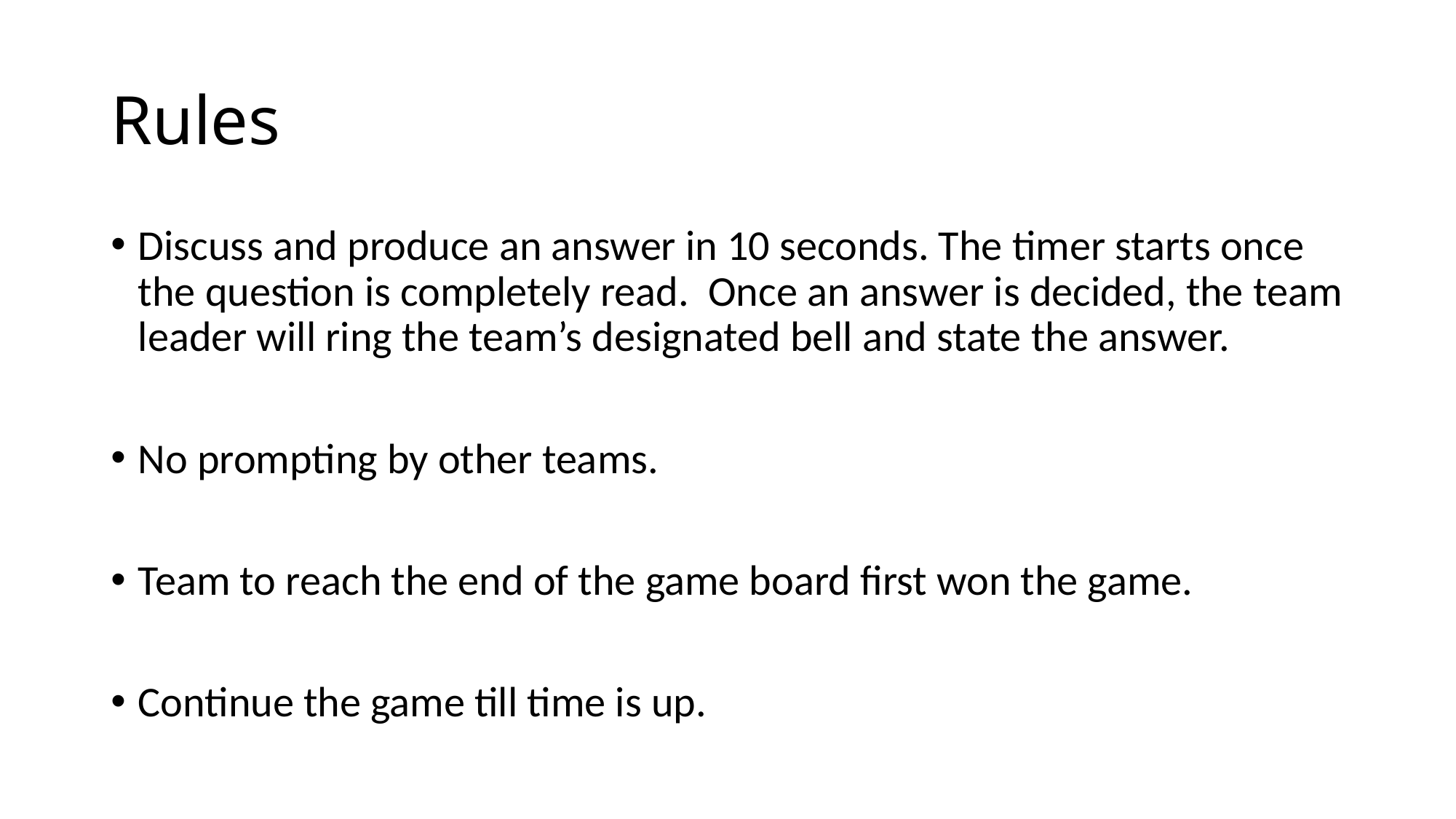

# Rules
Discuss and produce an answer in 10 seconds. The timer starts once the question is completely read. Once an answer is decided, the team leader will ring the team’s designated bell and state the answer.
No prompting by other teams.
Team to reach the end of the game board first won the game.
Continue the game till time is up.

## Slide 6
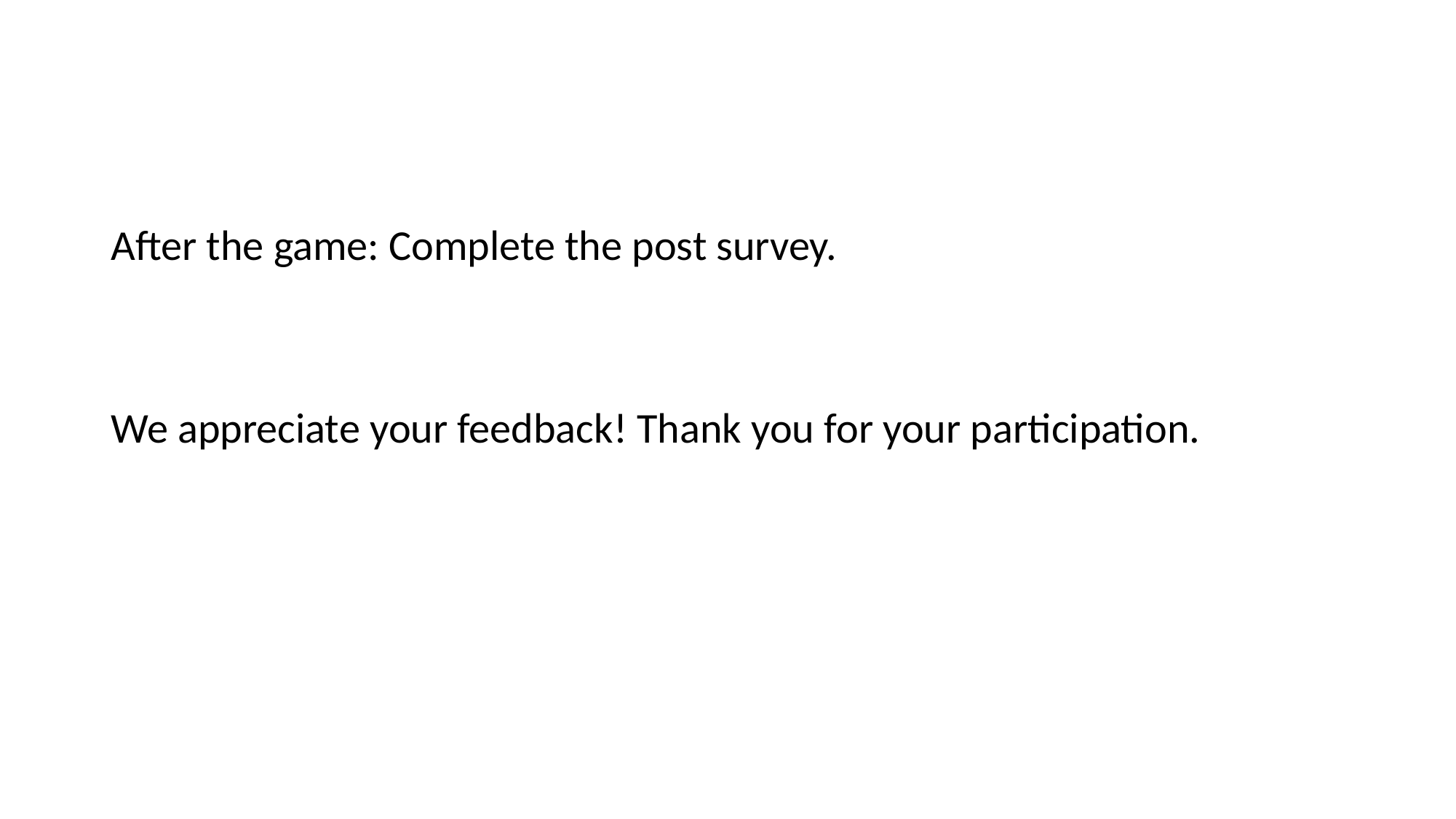

#
After the game: Complete the post survey.
We appreciate your feedback! Thank you for your participation.

## Slide 7
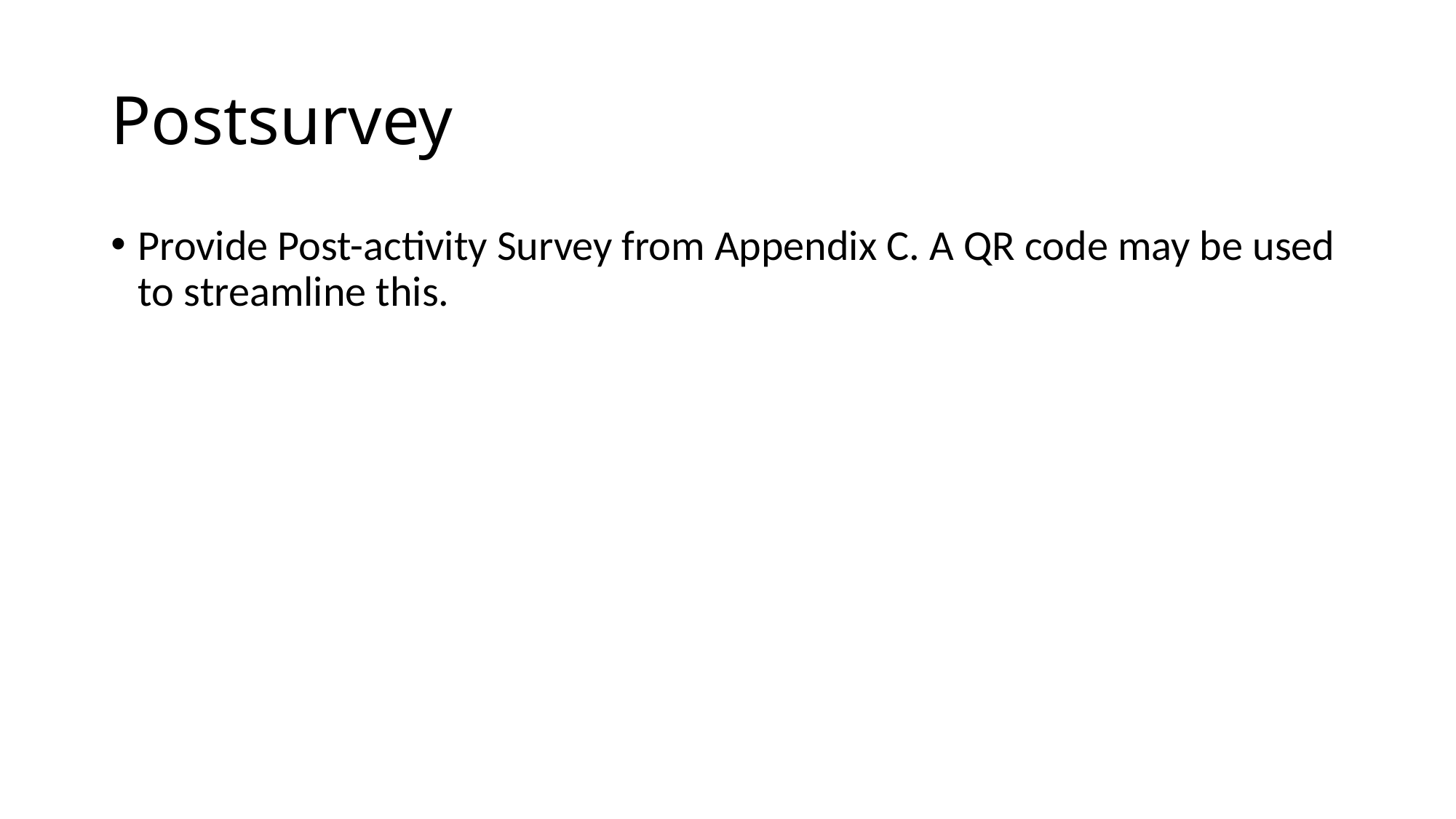

# Postsurvey
Provide Post-activity Survey from Appendix C. A QR code may be used to streamline this.

## Slide 8
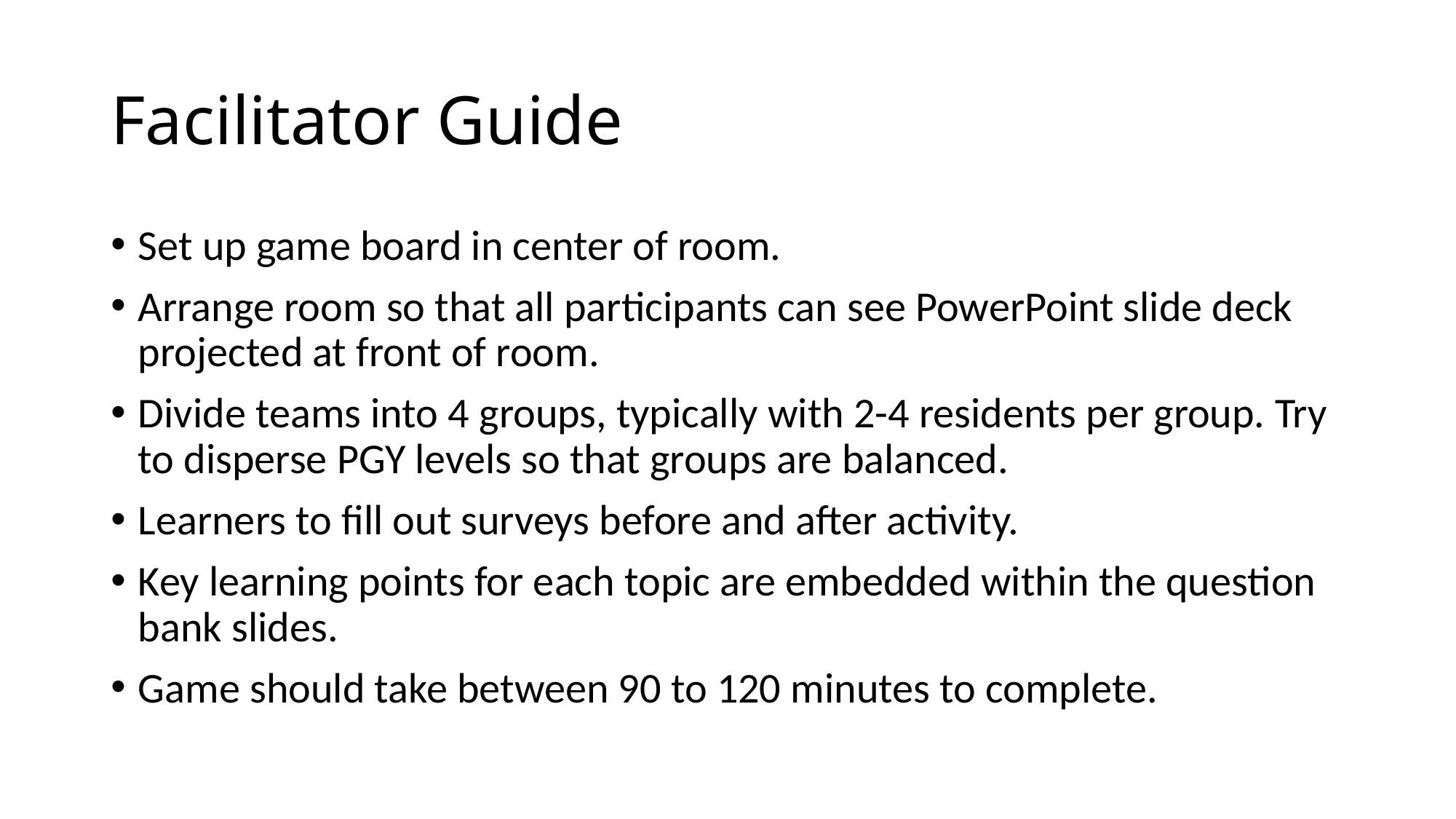

# Facilitator Guide
Set up game board in center of room.
Arrange room so that all participants can see PowerPoint slide deck projected at front of room.
Divide teams into 4 groups, typically with 2-4 residents per group. Try to disperse PGY levels so that groups are balanced.
Learners to fill out surveys before and after activity.
Key learning points for each topic are embedded within the question bank slides.
Game should take between 90 to 120 minutes to complete.
