# Supplementary material for: Rheum4Games: A Game-Based Board Review to Enhance Confidence and Knowledge in Rheumatology for Internal Medicine Residents
Source: MedEdPORTAL. 2026 May 1;22:11597. doi: 10.15766/mep_2374-8265.11597 (PMC13133093; doi:10.15766/mep_2374-8265.11597)
Supplement: Supplementary file 1 — Question Bank - Easier.pptxQuestion Bank - Challenging.pptxSurvey.docxGame Rules.pptxBoard Game.docx [file mep_2374-8265.11597-s001.zip › E. Board Game.docx]

**Appendix E:**

Link to example board game: [Board Game Link](https://www.amazon.com/Worldwide-24601-Jumbo-Snakes-Ladders/dp/B00LNHOFEQ/ref=sr_1_2?crid=37Y6AB4WV5WR8&dib=eyJ2IjoiMSJ9.39_EEZvlr6L4UH96Bh1orqsxDkZLUg1N0C_BCC4N0USC1djV_tnplJIca9n-U1B03H6kjJtoye0GvtGJFi0dIERn2so22fYudBbCTC9R7bnEPG720wkEonjo2jC01m9DPbqbb5Mgwcp0MdKwsp_JpDAjCv3fyhiLJgZsDYv7oyfsd4M6BOiQA1ddrb4c7dAhz39pJZX9xYmjdcvv5uDqrilA2NhESpm4jwPTG0ScpuS9HRKhazQRyqBktZDRFivgo5Ju3aTAf_lVfdlUM-wP_yInZVTm_fyiVyTOMuUPgVw.nl-9P6L5jum2d0pGnfzxNXJ0kUrYLAvE7TFUaOi4sIw&dib_tag=se&keywords=snake+and+ladder+play+mat&qid=1732207401&sprefix=snake+and+ladder+play+ma%2Caps%2C198&sr=8-2)

A board game template based off the childhood board game “Snakes and Ladders”.

*Worldwide 24601 Jumbo Snakes & Ladders [Internet]. Amazon.com; [cited 2026 Feb 23]. Available from:* [*https://www.amazon.com/Worldwide-24601-Jumbo-Snakes-Ladders/dp/B00LNHOFEQ/*](https://www.amazon.com/Worldwide-24601-Jumbo-Snakes-Ladders/dp/B00LNHOFEQ/)


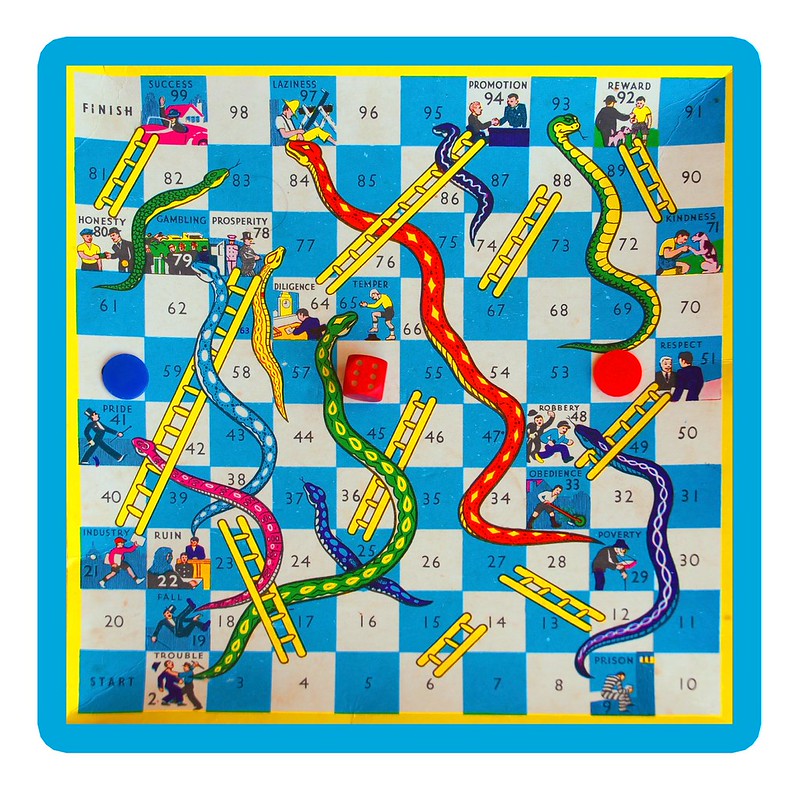
Snakes and Ladders by Leonard J Matthews, retrieved from: <https://www.flickr.com/photos/mythoto/9720925815> on 3/4/2026. Licensed as **CC BY-NC-SA 2.0**: <https://creativecommons.org/licenses/by-nc-sa/2.0/deed.en>
